# A *CCL21* gene-modified dendritic cell vaccine combined with pembrolizumab in advanced non-small cell lung cancer: a phase I trial

**DOI:** 10.1038/s41467-026-78130-7

**Published:** 2026-10-04

**Authors:** Michael S. Oh, Aaron E. Lisberg, Camelia Dumitras, Linh M. Tran, Ramin Salehi-Rad, Kostyantyn Krysan, Raymond J. Lim, Maryam Shabihkhani, Tahmineh Romero, Shuo Li, Weihua Zeng, Zhe Jing, Rui Li, Bitta P. Kahangi, Olga Tsvetkova, Robert D. Suh, Fereidoun Abtin, Scott J. Genshaft, Scott S. Oh, Gregory A. Fishbein, Daniel R. Greenwald, Kanwarpal S. Kahlon, Sidharth R. Anand, Shahryar Ashouri, Jonathan W. Goldman, Jay M. Lee, Xianghong J. Zhou, David A. Elashoff, Edward B. Garon, Bin Liu, Steven M. Dubinett

**Affiliations:** 1 Department of Medicine, David Geffen School of Medicine at UCLA, Los Angeles, California USA; 2 Department of Medicine, VA Greater Los Angeles Healthcare System, Los Angeles, California USA; 3 UCLA Health Jonsson Comprehensive Cancer Center, UCLA, Los Angeles, California USA; 4 Department of Molecular and Medical Pharmacology, David Geffen School of Medicine at UCLA, Los Angeles, California USA; 5 Department of Pathology and Laboratory Medicine, David Geffen School of Medicine at UCLA, Los Angeles, California USA; 6 Molecular Biology Institute, UCLA, Los Angeles, California USA; 7 David Geffen School of Medicine at UCLA, Los Angeles, California USA; 8 Department of Radiological Sciences, David Geffen School of Medicine at UCLA, Los Angeles, California USA; 9 Department of Surgery, David Geffen School of Medicine at UCLA, Los Angeles, California USA

**Keywords:** Tumour immunology, Non-small-cell lung cancer, Tumour vaccines

## Abstract

Immune checkpoint inhibitors (ICIs) have transformed treatment for non-small cell lung cancer (NSCLC), but resistance to these therapies is common. We report here results from a phase I trial combining intratumoral injection of a CCL21-expressing dendritic cell (CCL21-DC) vaccine with pembrolizumab in patients with advanced non-small cell lung cancer (ClinicalTrials.gov identifier: NCT03546361). The primary objectives were to determine the maximum administered dose (MAD) in the dose escalation phase and the objective response rate (ORR) in the dose expansion. Eleven patients were enrolled in the dose escalation cohort, and 12 patients were treated in the dose expansion cohort at the MAD of 3 ×10^7^ cells/injection. The trial did not meet its primary endpoint for ORR as no objective responses were observed, though 36.8% of patients achieved a best response of stable disease. The combination therapy was well tolerated. Post-hoc analyses of longitudinal biopsies revealed that the therapy increased tumor infiltration by CD4+ T cells and novel T cell clones. Disease stability was associated with increased T cell receptor diversity and greater expansion of novel clones following therapy. However, many novel clones failed to persist over time, and immunosuppressive myeloid signaling signatures were identified across time points. Our findings overall demonstrated that CCL21-DC vaccination combined with pembrolizumab can promote T cell infiltration, but this did not translate into meaningful clinical efficacy, potentially due to limited tumor specificity of the T cell response and an inability to fully reprogram the tumor microenvironment.

## Abstract (web summary)

Feasibility and safety profile of intratumoral administration of a DC vaccine overexpressing CCL21 (CCL21-DC) have been previously reported in patients with non-small cell lung cancer (NSCLC). Here the authors report safety, clinical outcomes and immunological correlates of a phase I trial of CCL21-DC in combination with anti-PD1 pembrolizumab in patients with advanced NSCLC.

## Introduction

Effective immunotherapy options are lacking for patients with advanced NSCLC who progress on ICIs, such as those blocking programmed cell death-(ligand)-1 [PD-(L)1]^1,2^. ICIs also have limited efficacy in patients with epidermal growth factor receptor (*EGFR*) or anaplastic lymphoma kinase (*ALK*) genomic alterations^3,4^. There is a pressing need for the development of novel therapies that can overcome both primary and secondary resistance to ICIs.

One approach to improving the efficacy of ICIs is to promote cytolytic T cell infiltration and activation in tumors^5,6^. T cell targeting of tumor-specific neoantigens represents an essential component of antitumor immunity^7^, and increased T cell infiltration has been associated with improved response to ICIs^8^. Enhancement of these immune parameters may be accomplished by bolstering the activity of antigen-presenting cells, such as dendritic cells (DCs), in the tumor microenvironment (TME), which can take advantage of the full repertoire of tumor antigens and promote local and systemic antitumor responses^9,10^. DC function can potentially be augmented by injecting DCs directly into tumors as a therapeutic cancer vaccine^11^. These DC vaccines are typically generated by expanding autologous DCs ex vivo and then modifying them to improve inflammatory functions^11,12^. For example, DCs can be genetically engineered to express cytokines that foster a TME that is conducive to cell-mediated immunity^13,14^. C-C motif chemokine ligand 21 (CCL21) represents a candidate chemokine for therapeutic use due to its ability to induce T cell trafficking and promote co-localization of T cells and DCs^15,16^.

Our prior phase I trial of intratumoral (IT) administration of DCs modified to overexpress CCL21 (CCL21-DC) in patients with advanced-stage NSCLC demonstrated the feasibility and favorable safety profile of in situ administration of the CCL21-DC vaccine^17–21^. Correlative studies revealed augmentation of tumor antigen presentation, resulting in increased T cell infiltration and evidence for systemic antitumor immune responses^19^. However, increased PD-L1 expression was observed in patient tumors in response to the CCL21-DC therapy, suggesting that the PD-1 axis may be utilized by tumor cells to inhibit T cell function and prevent a more robust CCL21-DC-mediated antitumor response. Combination studies of the CCL21-DC vaccine and anti-PD-1 therapy in murine models demonstrated a synergistic effect, leading to improved tumor control^22^.

Here, we report on a phase I trial combining IT CCL21-DC with pembrolizumab in patients with advanced NSCLC. This study was designed to evaluate the safety and antitumor activity of the combination therapy, with the hypothesis that the addition of pembrolizumab to the CCL21-DC vaccine would enhance tumor neoantigen presentation and T cell activation, thereby improving clinical outcomes for patients with limited historical benefit from ICI therapy. Immune monitoring studies accompanied the trial in order to characterize the immunologic effect as well as the underlying mechanisms of the trial therapy.

## Results

### Overview of the clinical study

**Fig. 1:**
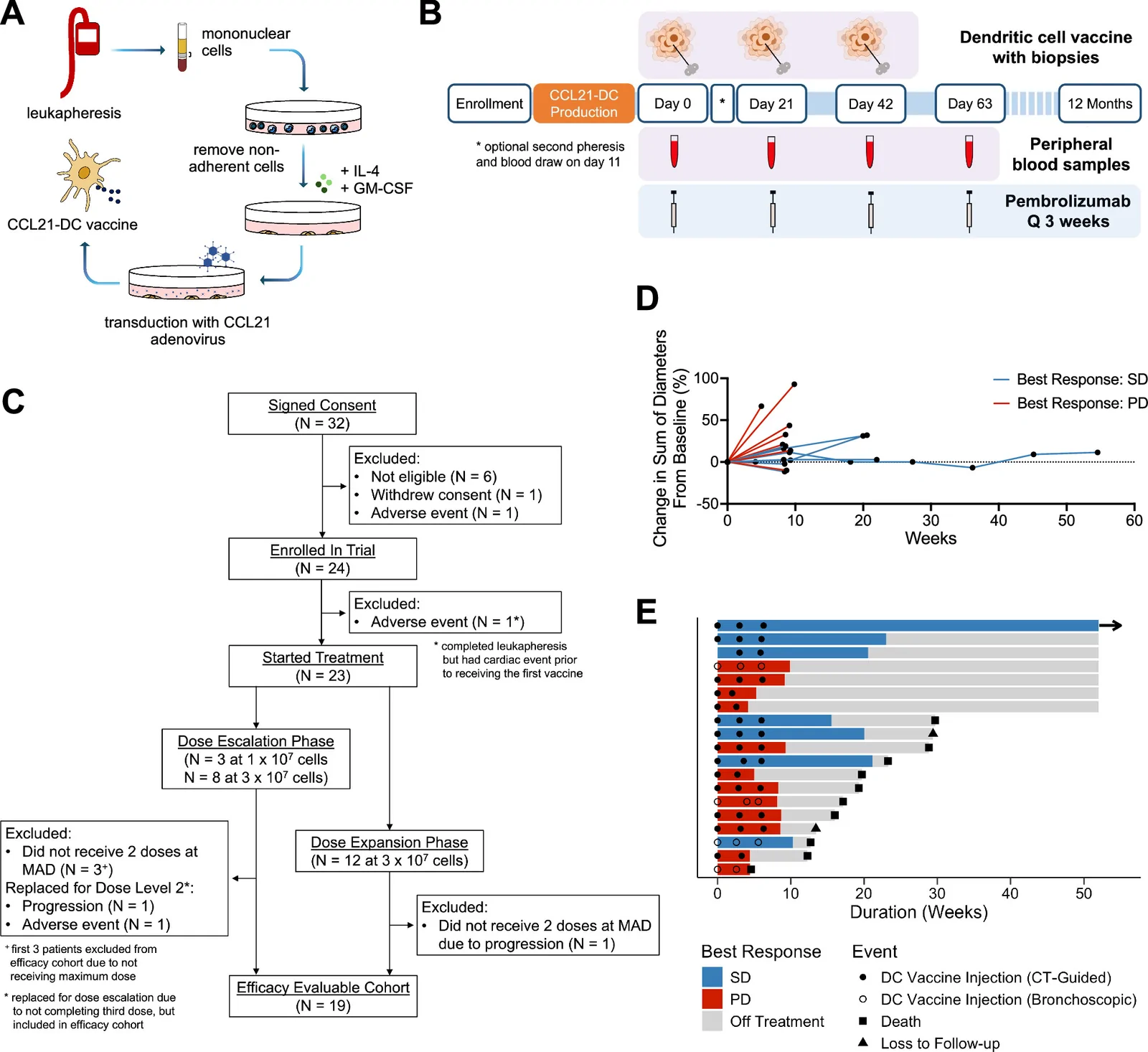
Phase I clinical trial of the CCL21-DC vaccine plus pembrolizumab. **A** Schematic illustrating the production method of the CCL21-DC vaccine. **B** Overview of the clinical trial design and schedule for patient biospecimen collection. **C** CONSORT flow chart showing patient enrollment and reasons for exclusion. MAD – maximum allowed dose, which was 3 × 10^7^ cells per injection of CCL21-DC vaccine. **D** Spider plot illustrating changes in total sum of diameters of target lesions over time, divided by SD (stable disease, *n* = 7) and PD (progressive disease, *n* = 12). **E** Swimmer plot showing time to progression with bars color- coded based on best response. The arrow indicates ongoing disease control. Source data are provided as a Source Data file.

Between July 2019 and January 2024, 23 NSCLC patients were treated with the combination of the CCL21-DC vaccine plus pembrolizumab. The CCL21-DC vaccine was manufactured by generating autologous monocyte-derived DCs (MoDCs) followed by transduction with a *CCL21* gene-expressing adenoviral vector, as previously described^23^ (Fig. 1A). The clinical trial treatment and biospecimen collection schedule is depicted in Fig. 1B. There were 11 patients in the dose escalation cohort (three patients given 1 × 10^7^ cells/injection and eight patients given 3 × 10^7^ cells/injection) and 12 patients in the expansion cohort. The injections were performed in a lung tumor through either a CT-guided percutaneous (*n* = 18) or bronchoscopic (*n* = 5) approach. A total of 19 patients received at least 2 injections at the MAD of 3 × 10^7^ cells/injection and comprised the efficacy evaluable subset (Fig. 1C). Demographic data and baseline characteristics are summarized in Table 1. The median age among all patients was 63 years. Three patients (13%) had an *EGFR*-activating mutation (2 cases with L858R and 1 case with exon 19 deletion) and, per protocol, had not previously received ICI therapy. In the full cohort, 21 of 23 (91%) patients had received chemotherapy.

**Table 1:** Patient demographics and baseline characteristics

Patient characteristic	All patients (*N* = 23)
**Age, years, median** (**range**)	63 (34–90)
**Sex, No**. (**%**)	
Male	10 (43)
Female	13 (57)
**Race, No**. (**%**)	
White	15 (65)
Asian	5 (22)
Other	3 (13)
**Ethnicity, No**. (**%**)	
Non-Hispanic of Latino	20 (86)
Hispanic or Latino	3 (14)
**Current or prior smoking history, No**. (**%**)	14 (61)
**ECOG performance status, No**. (**%**)	
0	13 (57)
1	10 (43)
**Histology, No**. (**%**)	
Squamous	4 (17)
Non-squamous	19 (83)
**PD-L1 tumor positivity, No**. (**%**)	
<1%	10 (43)
1–49%	6 (26)
≥50%	5 (22)
Unknown	2 (9)
**History of brain metastases, No**. (**%**)	8 (35)
**Prior lines of therapy, No**. (**%**)	
1	12 (52)
2	5 (22)
≥3	6 (26)
**Prior systemic treatments, No**. (**%**)	
Chemotherapy	21 (91)
Immune checkpoint inhibitor^a^	20 (87)
Tyrosine kinase inhibitor^b^	5 (22)

*ECOG* Eastern Cooperative Oncology Group.

^a^All received pembrolizumab; one patient also received ipilimumab and nivolumab.

^b^Three patients received osimertinib for *EGFR* mutations, one patient received capmatinib for *MET* exon 14 skipping mutation, and one patient received afatinib for *ERBB2* G600D mutation.

### Clinical outcomes

Dose limiting toxicities (DLTs) did not occur in any patients during dose escalation. Per protocol, six patients needed to be treated in dose escalation cohort 2, and two patients were replaced for MAD determination due to the inability to complete all 3 injections. The MAD of 3 × 10^7^ cells/injection was utilized for the expansion cohort. Adverse events (AEs) of any cause were experienced by 19 patients (83%, Supplementary Table 1). The most common AEs of any grade were dyspnea (35%), fatigue (30%), and cough (17%). Grade ≥3 AEs occurred in five patients (26%), including two grade 4 events and one grade 5 event of pneumonia. Treatment-related AEs (TrAEs) occurred in four patients (17%). One patient reported grade 1 flank pain associated with the injection, and three patients reported trAEs deemed to be immune-mediated, including one grade 3 trAE (4%) of immune-related diabetes mellitus.

Efficacy results are summarized in Supplementary Table 2 and Fig. 1. Among 19 efficacy evaluable patients, there were no confirmed objective responses [95% confidence interval (CI), 0.0–17.6%, Supplementary Table 2]. Disease control rate (DCR) was 36.8% (Fig. 1D, E, and Supplementary Fig. 1A). Stability of tumor size was observed more frequently in lesions injected with the CCL21-DC vaccine compared to other target lesions (Supplementary Fig. 1B, C). However, no tumors experienced 30% or greater reductions in size. After a median follow-up time of 32.3 months, median PFS was 2.1 months [95% CI, 1.2–3.6 months] and the median overall survival (OS) was 6.9 months (95% CI, 4.0 months-not evaluable, Supplementary Fig. 1D, E).

The longest duration of disease control was 12 months, achieved by a patient who completed the full year of protocol-planned treatment and was then censored per protocol. This patient had tumor PD-L1 expression of 80% by immunohistochemistry and a *KRAS* G12D mutation by next-generation sequencing (NGS) of the tumor. He was initially treated with single-agent pembrolizumab for 2 years with good response, but he was then noted to have an increase in size and number of over 20 bilateral pulmonary nodules. This progression included a doubling in the diameter of several lesions over a 6-month period. He was then enrolled in the clinical trial and received 3 injections of the CCL21-DC vaccine into the largest lung tumor (a 2.7-cm right lower lobe lesion), with subsequent stability observed in all pulmonary nodules.

Another patient with a best response of stable disease (SD) had resolution of a brain metastasis that was not treated with local therapy (Supplementary Fig. 1F). This patient had poorly differentiated carcinoma with tumor PD-L1 of 10% and multiple mutations identified on NGS, including loss-of-function variants in *TP53*, *STK11*, *NF1*, and *CDKN2A*. He was treated with carboplatin, pemetrexed, and pembrolizumab with favorable response, and he continued maintenance therapy for 14 months before experiencing progression in a 5.6-cm left lower lobe lung mass and 4.4-cm sacroiliac mass. An MRI of the brain during screening showed a new 4 mm enhancing focus in the superior frontal gyrus with minimal associated FLAIR hyperintensity. Due to the small size and asymptomatic nature of the metastasis, he continued on the trial therapy as permitted per protocol. The brain lesion was not seen on repeat MRI 3 months later. Body imaging at 4 months showed stability of the lung mass that was injected with the CCL21-DC vaccine, but there was progression in the sacroiliac mass.

NGS had been performed on all tumors as part of routine clinical care using either an in-house panel of actionable gene alterations or commercial assays (Supplementary Fig. 1G). The SD group was enriched in *KRAS*-mutant tumors, including those with co-mutations, such as *STK11* or *KEAP1*. The progressive disease (PD) group most commonly harbored *TP53* mutations.

### Disease control after therapy was associated with greater tumor immunoediting

**Fig. 2:**
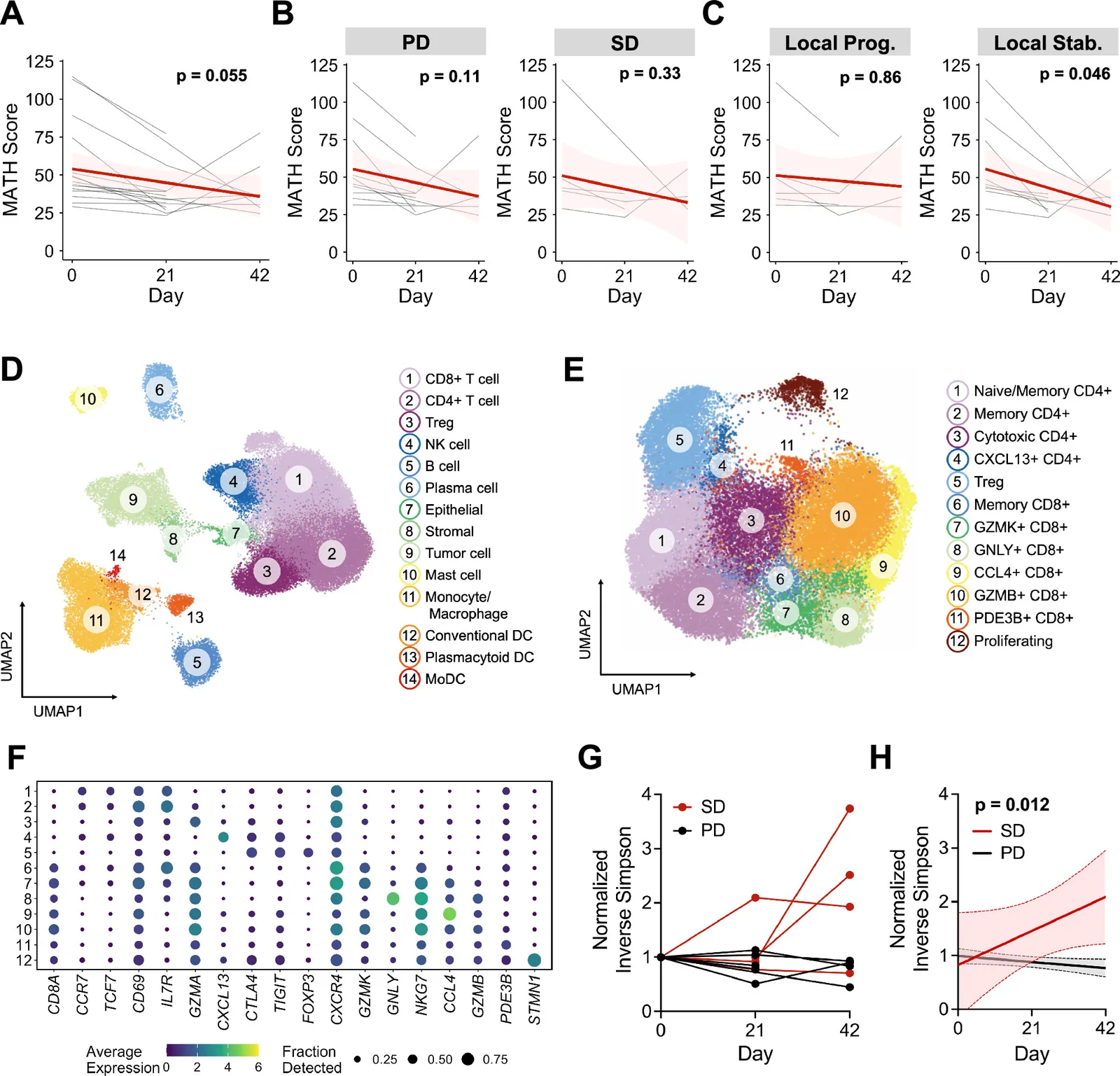
Correlates to clinical outcomes after CCL21-DC vaccine and pembrolizumab. **A**–**C** Mutant-Allele Tumor Heterogeneity (MATH) scores were derived from whole exome sequencing (WES) of tumor samples (*n* = 15 patients). Statistical significance was assessed using linear mixed-effects models; *p*-values were determined by two-sided t-tests. Shading indicates 95% confidence bands. Tumor MATH scores over time are depicted for (**A**) the full cohort over time; **B** the cohort separated between patients with progressive disease (PD, *n* = 10 patients) and those with stable disease (SD, *n* = 5 patients); and **C** the cohort separated between patients with local tumor progression (Prog., *n* = 6 patients) and those with local tumor stability (Stab., *n* = 9 patients). **D** scRNA-seq analysis of samples from 17 patients and 41 samples reveals clusters with T cell predominance. Treg – regulatory T cell, DC – dendritic cell, MoDC – monocyte-derived dendritic cell. **E** Subclusters within the T cell scRNA-seq data annotated based on (**F**) expression of marker genes. **G** TCR diversity within tumors (*n* = 10 patients) over time as measured by the inverse Simpson index, with comparison between SD (*n* = 4 patients) and PD (*n* = 6 patients) samples. **H** Linear mixed effects models demonstrated a significant increase in TCR diversity within SD samples but not PD samples, as in (**G**). Shading indicates 95% confidence bands; *p*-values were determined by two-sided t-tests. Source data are provided as a Source Data file.

Post-hoc immune monitoring studies were performed using longitudinal tumor biopsy and peripheral blood samples. Core biopsies of the DC-injected tumors were obtained on days 0, 21, and 42, with samples pooled from four quadrants of each tumor to account for tumor heterogeneity. To evaluate tumor evolution in response to treatment, whole exome sequencing (WES) was performed on genomic DNA extracted from tumor tissue. Identification of sequencing variants demonstrated temporal changes in the mutational profile of patient tumors. The mutant-allele tumor heterogeneity (MATH) score demonstrated a trend towards decreased heterogeneity over time in the full cohort (Fig. 2A). There was no significant trend in MATH scores among tumor samples from SD or PD patients (Fig. 2B). However, when samples were grouped based on local response in the injected tumor, there was a decrease in MATH scores specifically in those with local disease stability (Fig. 2C). Given that reductions in tumor heterogeneity often reflect effective immunoediting, these findings suggest that the CCL21-DC vaccine may have impacted clinical outcomes by affecting local intratumoral immunologic responses.

### TCR diversity increases after trial therapy in SD tumors

To characterize the intratumoral immune response, we performed combined single-cell RNA with T cell receptor sequencing (scRNA/TCR-seq) on dissociated tumor samples, starting with the sixth patient. One patient had only one biopsy sample and was excluded from the analysis. After quality control, we obtained and processed data from a total of 65,344 cells across 41 total samples from 17 patients and included biopsies from days 0, 21, and 42. Cell clusters were annotated based on the expression of canonical markers (Fig. 2D and Supplementary Fig. 2A). The majority of the recovered cells were immune cells, though tumor and stromal cells were also identified at varying frequencies.

Selecting for T cells resulted in a subset of 40,005 cells that subclustered into 12 groups identified by predominant marker genes (Fig. 2E, F). Five of these clusters were composed of CD4+ cells, including (1) a naive/memory cluster with higher expression of *CCR7*, *SELL*, and *TCF7* and (2) a second memory subset with relatively higher expression of *CD69* and *IL7R*. There was also (3) a cytotoxic CD4+ cluster, (4) a cluster with high *CXCL13* expression, and (5) a cluster with high *FOXP3* and *CTLA4* expression consistent with regulatory T cells (Tregs). CD8+ clusters included (6) a cluster characterized by a memory phenotype and comparatively lower expression of effector genes, (7) a cluster with high *GZMK* and low *GZMB* expression, (8) a cluster with terminal effector phenotype including high *GNLY* and *NKG7* expression, (9) an inflammatory cluster marked by elevated *CCL4* expression, (10) a cytotoxic subset with high *GZMB* expression, (11) a small cluster of activated CD8+ T cells marked by *PDE3B* expression, and (12) a cluster consisting of proliferative CD4+ and CD8+ T cells. Cluster 10 exhibited relative enrichment in exhaustion genes (Supplementary Fig. 2B), such as *PDCD1* (PD-1) and *ENTPD1* (CD39). In addition, gene signatures previously validated to identify neoantigen-specific CD8+ T cells^24–26^ were expressed by a higher proportion of cells in clusters 9 and 10 (Supplementary Fig. 2C). No significant trends in relative T cell cluster proportions were observed over time (Supplementary Fig. 2D).

Among the full T cell subset, 86% had their TCR sequence identified by single-cell TCR-seq (Supplementary Fig. S2E). Quality control measures were implemented to include patients with a baseline sample, at least 1 post-treatment sample, and >50 T cells in each of these samples for a robust evaluation of TCR clonal evolution. There were 10 patients whose samples met these criteria, accounting for 18,707 T cells with 11,433 distinct clonotypes. The diversity of TCR clones was then measured at each timepoint using the inverse Simpson index and fit to linear mixed-effect models. Intratumor TCR repertoires from SD patients exhibited an increase in TCR diversity over time, while those with PD exhibited no change in TCR diversity (Fig. 2G, H). These findings were driven predominantly by three patients who experienced an over 2-fold increase in intratumoral TCR diversity; these subjects included the patient with an intracranial response, the patient with over 1 year of disease control, and a third patient who also achieved SD.

### Tumor infiltration by CD4+ T cells increases after CCL21-DC vaccination

**Fig. 3:**
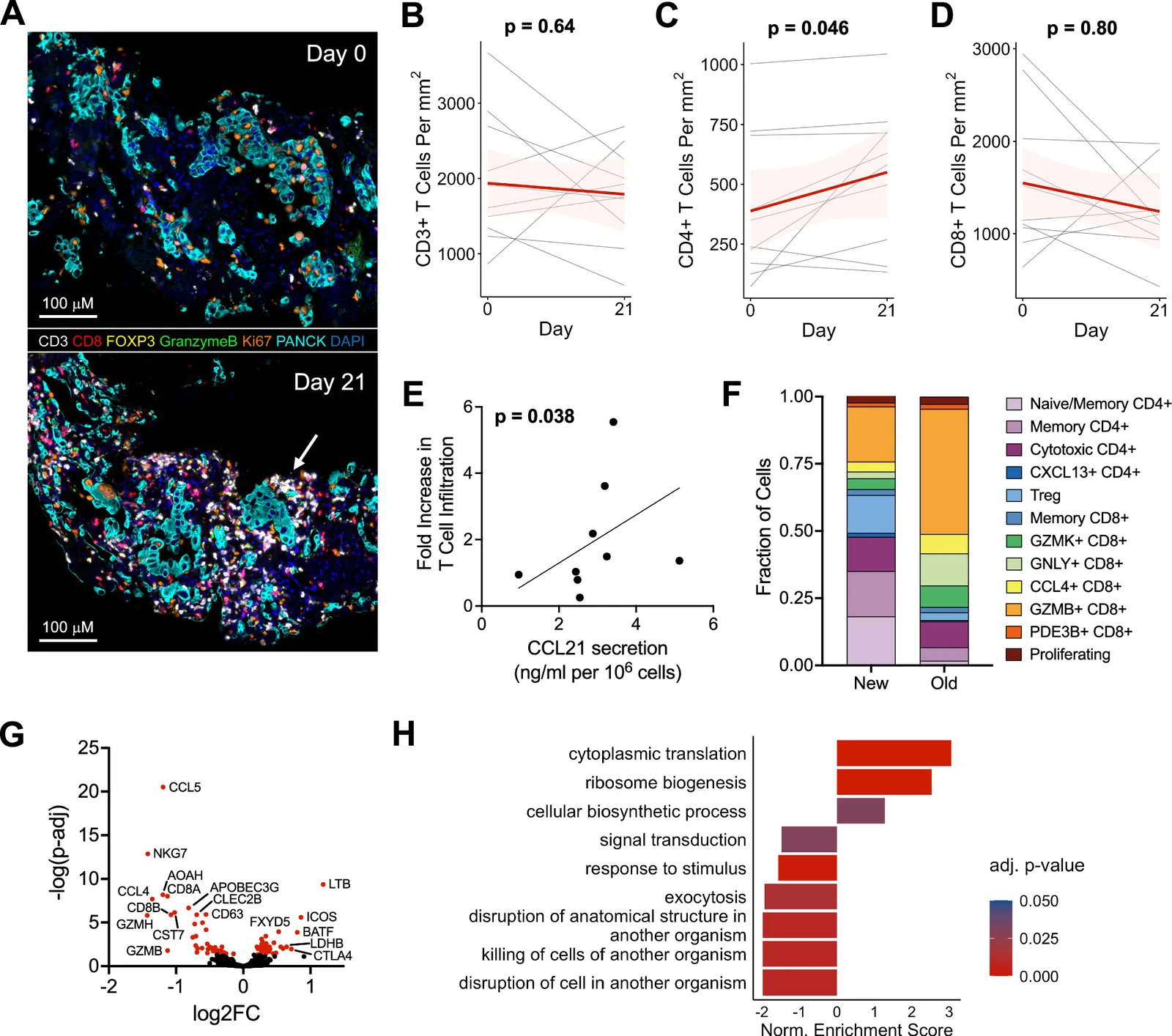
CCL21-DC vaccine and pembrolizumab induce CD4+ T cell tumor infiltration. **A** Representative MIF images at day 0 (above) and day 21 (below) showing T cell infiltration into tumor regions (white arrow). **B**–**D** Quantification of tumor infiltration (*n* = 10 patients) on days 0 and 21 by T cells. Statistical significance was assessed using linear mixed-effects models; *p*-values were determined by two-sided t-tests. Shading indicates 95% confidence bands. Results are shown for **B** all CD3+ T cells, **C** CD4+ T cells, and **D** CD8+ T cells. **E** Spearman correlation between CCL21 secretion by CCL21-DC vaccine batches and the fold increase in T cell infiltration after injection with that batch (*n* = 9). **F** Proportion of novel (new) or pre-existing (old) clones within scRNA-seq T cell clusters (*n* = 10 patients). **G** Volcano plot showing genes with increase in expression over time when compared between novel and pre-existing clones (*n* = 10 patients). **H** Gene ontology terms found to be significant based on the DEGs, with positive scores representing enrichment in novel clones (*n* = 10 patients). Source data are provided as a Source Data file.

An increase in TCR diversity could be driven by an intrinsic change in TCR repertoire composition, infiltration by new T cell clones, or both. We performed multiplex immunofluorescence (MIF) studies to quantify T cell infiltration into the tumor before and after receiving the CCL21-DC vaccine plus pembrolizumab (Fig. 3A, B). Following quality control measures, we analyzed samples from 13 patients who had sufficient biopsy tissue at two or more time points containing at least 10% tumor content, including 10 patients with adequate tissue on day 21. There was an increase in CD4+ (Fig. 3C) but not CD8+ (Fig. 3D) T cells in tumors after the first dose of vaccine. In several patients, an initial increase in T cell infiltration on day 21 abated by day 42, leading to no significant trend when considering all time points (Supplementary Fig. 3A–C). CD8+ T cells increased in tumors from 5 of 13 patients, but there was no significant trend and no difference in these trends between SD and PD tumors (Supplementary Fig. 3D–F). There was also no trend over time in regard to tumor infiltration by Tregs or GZMB+ CD8+ T cells (Supplementary Fig. 3G, H).

Given that the therapy was engineered to secrete CCL21 as a T cell chemoattractant, we sought to determine whether there was a relationship between T cell infiltration and CCL21 secretion. We had previously reported on a characterization of the CCL21-DC vaccine that included ELISA measurements of CCL21 secretion by DC vaccine batches used in the clinical trial^23^. CCL21 production by a vaccine batch correlated positively with the increase in intratumoral T cell infiltration following injection of that batch (Fig. 3E). There was no increase in the proportion of Ki67 positivity in CD4+ or CD8+ T cells over time (Supplementary Fig. 3I, J), implying that the increase in CD4+ T cell infiltration was not driven by greater proliferation.

We next performed neighborhood analysis using pan-cytokeratin-expressing tumor cells as centroids to analyze immune-tumor cell interactions. The proportion of CD4+ T cells, including Tregs, within tumor neighborhoods increased over time, though there was no difference between SD and PD samples (Supplementary Fig. 4A–D). We also evaluated whether there were correlating changes among tumor cells. There were no significant trends in regard to tumor density or Ki67 positivity over time, though SD samples numerically exhibited a relative decrease in both measures (Supplementary Fig. 4E, F). There was no significant correlation between the change in CD4+ T cells and these tumor measures (Supplementary Fig. 4G, H).

We sought to characterize newly infiltrating T cells by focusing on novel clones detected only in post-treatment biopsies (i.e., days 21 and 42) in the scRNA/TCR-seq dataset. Novel clones were predominantly found in CD4+ T cell clusters (Fig. 3F). There was a disproportionately large fraction of naive/memory CD4+ T cells with relatively high expression of *CCR7*, which encodes the receptor for CCL21. Some of the CD4+ skew could potentially be attributed to the greater diversity inherent to CD4+ TCR repertoires, such that repeat sampling even within the same tumor can yield different CD4+ T cell clones. To account for this, we examined only TCRs that were multiplet clones with at least 0.2% clonal proportion within the tumor, representing the 95th percentile of expanded clones. This subset showed greater similarity between novel and pre-existing clones, but there was still relative overrepresentation of naive/memory and cytotoxic CD4+ cells as well as Tregs (Supplementary Fig. 5A).

Differentially expressed gene (DEG) analysis confirmed that novel clones were enriched in memory markers, such as *IL7R* and ribosomal genes, which are typical in scRNA-seq of naive/memory CD4+ cells (Fig. 3G and Supplementary Data 1). There were several genes linked to clonal expansion, including *JUNB* and *FXYD5*, and genes related to CD4+ cell activation and Treg development, such as *ICOS* and *CTLA4*. There was de-enrichment of genes related to cytotoxic and terminal effector T cell phenotypes, including *GZMA*, *GZMB*, *CCL5*, and *NKG7*. Gene ontology analysis confirmed enrichment for protein synthesis processes in novel clones as well as de-enrichment for signaling- and cytotoxicity-related processes (Fig. 3H). When comparing cluster distribution of these clones based on clinical response, SD patients were found to have a greater proportion of pre-existing clones in the GZMB+ CD8+ cluster at baseline (Supplementary Fig. 5B). There were, however, no differences between PD and SD groups in regard to the cluster distribution of novel versus pre-existing clones (Supplementary Fig. 5C).

### Expansion of novel intratumoral clones is greater in SD patients

**Fig. 4:**
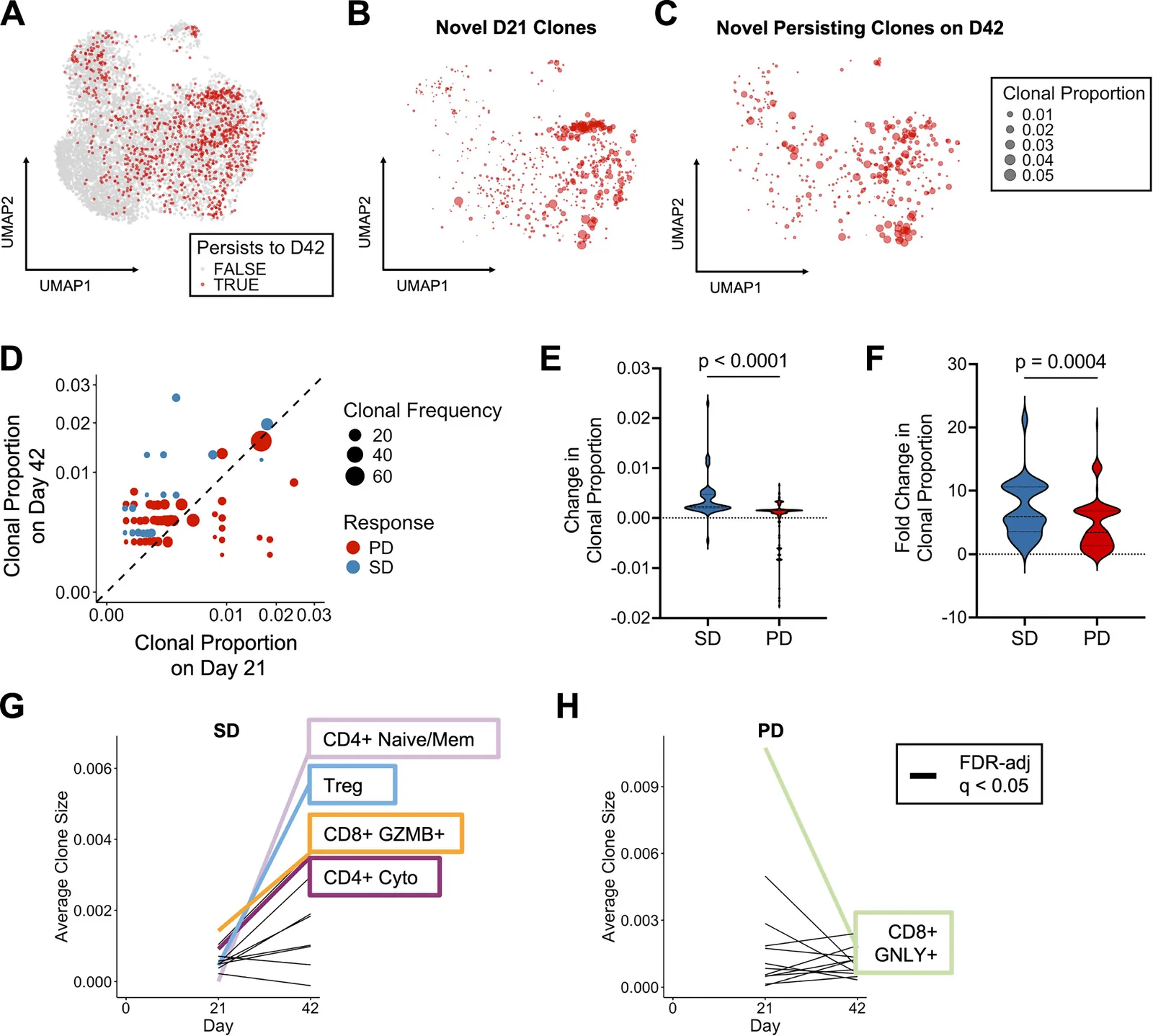
Intratumoral expansion of persistent novel clones. **A** UMAP plot (*n* = 10 patients) highlighting cells that represent post-therapy novel clones on day 21 that persisted to day 42 (red dots). **B** UMAP plot (*n* = 10 patients) of novel persistent clones on day 21 and **C** day 42, with the size of the dot correlating with the clonal proportion. **D** Scatter plot of persistent novel clones (*n* = 10 patients) with comparisons made between patients with progressive disease (PD, *n* = 6 patients) and stable disease (SD, *n* = 4 patients). Clones in the upper left have greater expansion on day 42. **E** Quantification of the absolute change in clonal proportion of persistent novel clones from day 21 to day 42, as well as the **F** fold change. Comparisons made using the two-sided Wilcoxon rank-sum test. **G** Average clone size of novel persistent clones in SD samples (*n* = 4 patients) separated out by cluster. Bold and labeled lines represent clusters with a statistically significant non-zero slope based on false discovery rate (FDR)-adjusted *q*-value < 0.05. **H** Same as above, but with novel persistent clones in PD samples (*n* = 6 patients). Source data are provided as a Source Data file.

We next examined the evolution of novel clones over time. Many clones that emerged on day 21 were not seen on day 42 (Fig. 4A), which could be due in part to sampling and detection variations. However,  novel clones on day 42 were 2.7 times more likely to be found on day 21 than vice versa, suggesting that there may have been clonal loss, such as due to antigen neglect or other immunosuppressive factors. Among clones that persisted through day 42, there was a mean 5.2-fold increase in clonal proportion, indicating robust expansion (Fig. 4B, C). This expansion was more pronounced in SD patients compared to PD patients (7.1-fold expansion vs 4.6-fold expansion, *p* = 0.0001, Fig. 4D–F).

To better characterize these novel and persistent clones, we first cross-referenced the T cells with a database of validated viral clonotypes^27^. This analysis was limited by an overall low number of viral clones detected, but novel T cell clones mapped onto viral clonotypes at low frequencies (Supplementary Fig. 5D). We also applied gene signatures of neoantigen-specific T cells onto novel T cell clones and found that persistent clones were more likely to express these signatures than transient ones (Supplementary Fig. 5E). When separated by clusters, persistent clones expanded specifically within the CD4+ Naive/Mem, CD4+ Cyto, Treg, and CD8+ GZMB+ clusters in SD samples (Fig. 4G). In contrast, there was a trend towards clonal contraction in PD samples, including a strong reduction in the average clone size within the CD8+ GNLY+ cluster (Fig. 4H).

### Tumor samples exhibit immunosuppressive myeloid-T cell interactions

**Fig. 5:**
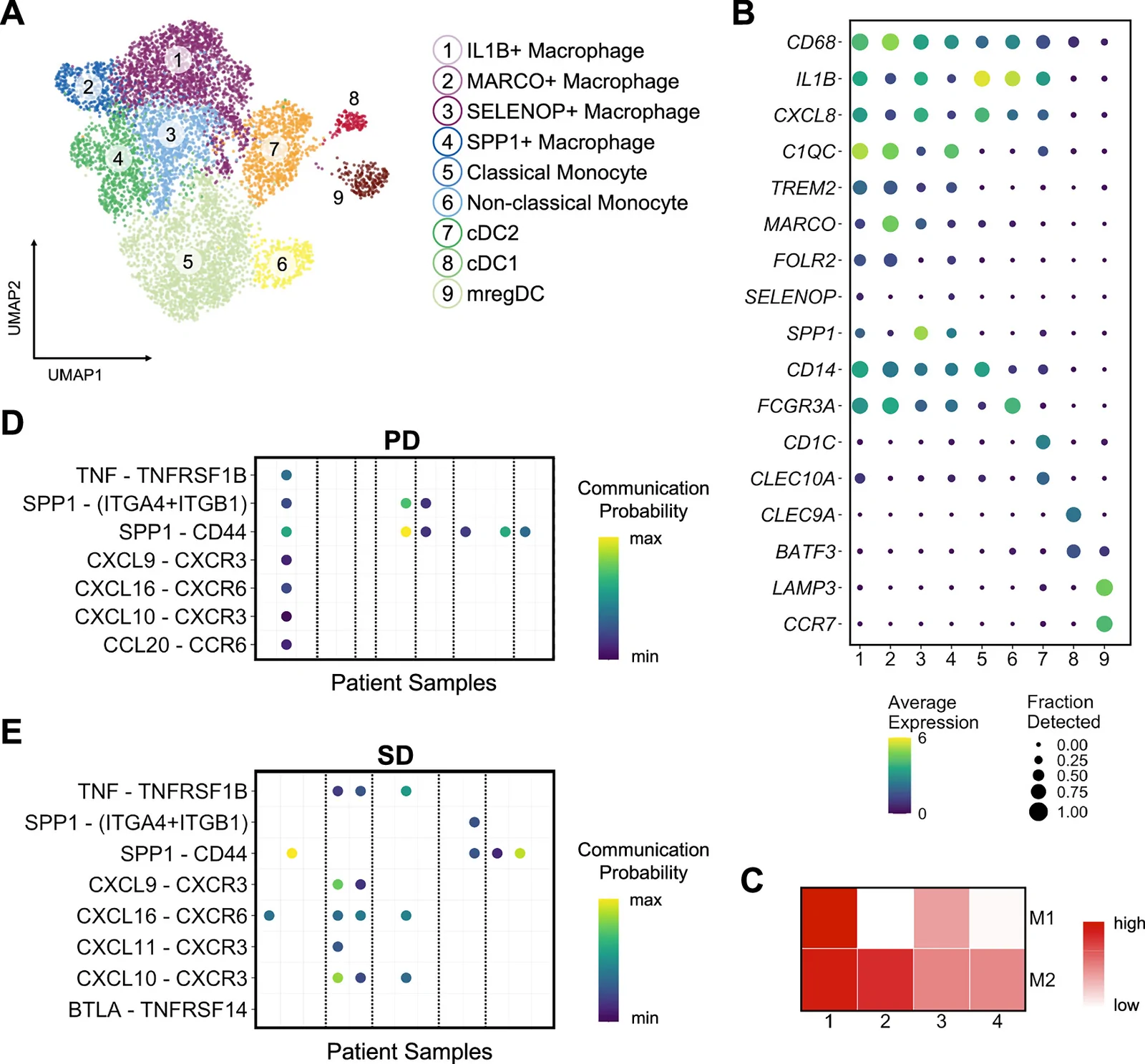
Myeloid compartment of the tumor immune microenvironment. **A** UMAP plot (*n* = 17 patients) showing myeloid cell clusters identified by tumor scRNA-seq analysis. cDC – classical dendritic cell, mregDC – mature dendritic cell enriched in immunoregulatory molecules. **B** Dot plot showing expression of marker genes identifying myeloid clusters. **C** Heatmap of expression of M1 or M2 signatures across the four macrophage clusters. **D**, **E** Ligand–receptor interaction inference plot with dots showing interactions between macrophages and T cells, with each column representing a patient sample and individual patients separated by vertical dotted lines. Each dot represents an interaction with a statistically significant probability. Results are divided between **D** progressive disease (PD) samples (*n* = 7 patients) and **E** stable disease (SD) samples (*n* = 5 patients).

We further evaluated relationships between other cell types and T cells to understand the effect of the TME. Due to variable sample quality, there was inconsistency in the presence of non-T cell components, but there was more uniform capture of the myeloid compartment, with sufficient cells available for 12 patients across 28 samples. A total of 7428 myeloid cells were recovered in these samples, and clustering analysis demonstrated a large proportion of *IL1B*+ and *SPP1+* macrophages, which have been reported to suppress the antitumor immune response^28^ (Fig. 5A, B). The macrophage clusters also had elevated expression of other pro-tumorigenic markers, including *MARCO*^29^ and *TREM2*^30^. Application of M1/M2 gene signatures^31^ showed that the immunosuppressive and pro-tumorigenic M2 phenotype was dominant across macrophage clusters (Fig. 5C). Inference of ligand-receptor interactions revealed that SPP1-related signaling was the most common interaction between macrophages/monocytes and T cells. Evidence for this signaling interaction occurred in tumor samples from 5 of 7 PD patients and 3 of 5 SD patients (Fig. 5D, E). In contrast, the immune-enhancing CXCL9/10-CXCR3 signaling axis, which has been linked to improved outcomes from ICIs^32^, was observed in tumor samples from only three patients in total (Fig. 5D, E).

### Systemic immune signatures associated with clinical outcomes

**Fig. 6:**
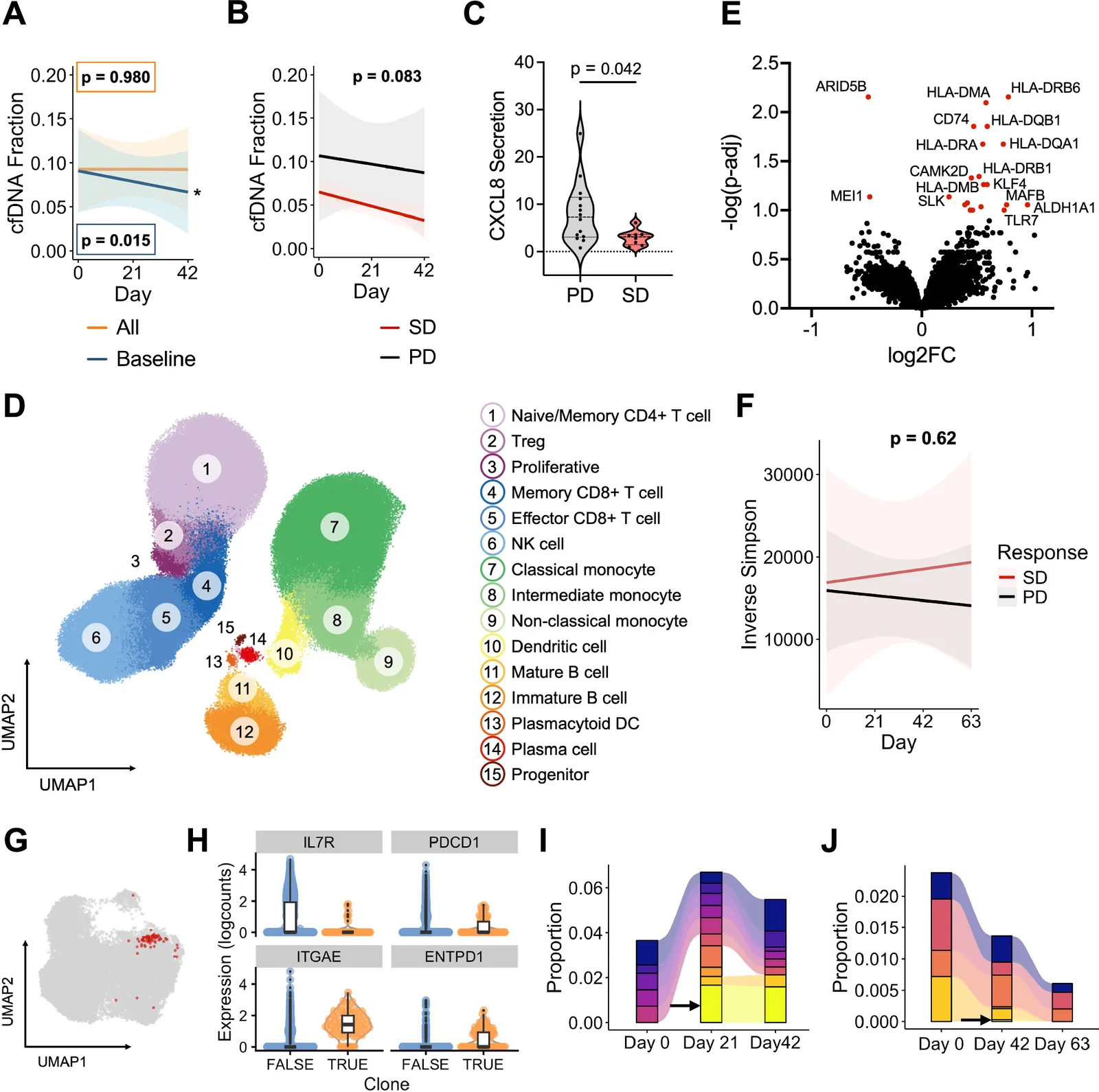
Systemic immune signatures associated with clinical outcomes. **A**, **B** Quantification of tumor fraction within cfDNA (cell-free DNA, *n* = 16 patients). Statistical comparison made using linear mixed-effects models; *p*-values were determined by two-sided t-tests. Shading indicates 95% confidence bands. Results were compared **A** between all or only baseline mutations, and **B** compared between stable disease (SD, *n* = 6 patients) and progressive disease (PD, *n* = 10 patients) groups using baseline mutations. **C** Luminex analysis showed increased baseline CXCL8 secretion in PD (*n* = 15 patients) compared to that in SD (*n* = 8 patients) samples. Statistical comparisons were made using the two-sided Wilcoxon rank-sum test. **D** Clusters identified in scRNA-seq data of patient PBMCs (*n* = 18 patients) on days 0, 21, 42, 63. **E** Volcano plot showing differentially expressed genes over time between SD (*n* = 7 patients) and PD (*n* = 9 patients) samples. Positive fold change represents genes with increased expression over time in SD but not PD samples. **F** There was no difference between SD (*n* = 8 patients) and PD (*n* = 11 patients) samples in regard to TCR diversity as measured by the inverse Simpson index. Statistical comparison made using two-sided t-tests obtained from a linear mixed effects model. Shading indicates 95% confidence bands. **G** ScRNA/TCR-seq of biopsies from a patient revealed an expanded post-therapy novel clone grouped primarily in the CD8+ GZMB+ T cell cluster. **H** Violin plots show significantly higher expression of exhaustion markers, such as *PDCD1* (PD-1) and *ENTPD1* (CD39) and the tumor resident marker *ITGAE* (CD103) in this clone (*n* = 1471 total cells). Box plot indicates median with interquartile range (IQR) with whiskers extending to 1.5 × IQR. **I** Alluvial plots of the top 10 most common clones on day 21, with the arrow indicating this novel expanded clone. **J** Similar analysis of the peripheral TCR repertoire shows that this clone did not exist at baseline, but it was transiently detectable on day 42. Source data are provided as a Source Data file.

To assess tumor evolution at a systemic level, we performed cell-free DNA (cfDNA) sequencing of patient plasma samples and estimated the fraction of tumor DNA based on the frequency of somatic variants. The tumor fraction based on baseline mutations decreased from day 0 to day 42, while the tumor fraction based on all mutations remained stable over time (Fig. 6A). These results are suggestive of systemic immunoediting against pre-existing tumor mutations that are offset by the emergence of de novo mutations. There were no significant differences in tumor fraction trends based on clinical outcomes (Fig. 6B).

To evaluate corresponding systemic immune changes, we measured levels of 54 cytokines in the peripheral blood by Luminex. CXCL8 was identified as the only cytokine that was significantly elevated at baseline in blood samples from PD patients as compared to those from SD patients (Fig. 6C), consistent with the previously reported association between CXCL8 and reduced clinical benefit of ICIs^33,34^. Assessing the rate of change over time from baseline to day 63 revealed significant differences between SD and PD groups in 5 cytokines. We observed significant induction of IL-3 in the SD group and reduction of XCL1 in the PD group. There was a significant relative reduction of CCL20, IL-10, and TGFα in the SD group, with opposing trends in the PD group (Supplementary Fig. 6A–E).

We next performed scRNA-seq on PBMCs obtained on days 0, 21, 42, and 63 of the clinical trial to evaluate systemic immune signatures associated with clinical outcomes (Fig. 6D). Downstream analysis yielded clusters consistent with known peripheral immune cell types. There were no significant differences in cluster frequencies when comparing pre- and post-therapy samples or the SD and PD groups (Supplementary Fig. 6F). DEG analysis revealed that a set of genes almost exclusively related to MHC-II were significantly upregulated over time in SD but not in PD patient samples (Fig. 6E and Supplementary Data 2). Correlations between MHC-II gene expression and ICI response have typically been observed in tumor but not immune cells^35^, while the importance of MHC-II neoantigen presentation to the antitumor immune response has been well described^35^.

### Peripheral TCR repertoire partially reflects changes in intratumoral repertoire

To further explore the impact of the CCL21-DC vaccine and pembrolizumab combination therapy on the systemic immune response, we performed bulk TCR-seq on PBMCs. In contrast to the intratumoral TCR repertoire, there was no significant difference between SD and PD samples when assessing changes in peripheral TCR diversity over time (Fig. 6F). We then assessed the frequency of novel intratumoral clones, defined as those newly detected in the tumor on day 21 or day 42, in the peripheral TCR repertoire. We found that 45.9% of novel clones could be detected in the peripheral compartment and 35.5% could be detected in day 0 blood samples, compared to 51.3% and 41.5% of pre-existing clones, respectively (Supplementary Fig. 6G). These findings are consistent with prior studies showing moderate overlap between the intratumor and peripheral TCR repertoires^36^, and further suggest that at least a portion of novel clones represent T cells that have migrated into the tumor from systemic circulation. We then identified clones that were not present in either the tumor or peripheral blood on day 0 to isolate novel clones that were more likely to have been generated in the TME and tumor-draining lymph nodes. Only 16.1% of these clones were found in the peripheral TCR repertoire at any time point, indicating much lower clonal overlap. These clones exhibited a decrease in CD4+ T cell clonal fractions over time, indicating that these emergent clones were unable to persist in the peripheral blood (Supplementary Fig. 6H–J).

Despite these results, we were able to identify individual instances where the generation of novel intratumoral TCR clones was accompanied by clear corresponding changes in the systemic TCR repertoire. In one patient with PD but stability in the CCL21-DC-injected tumor, day 21 biopsies demonstrated the emergence of an expanded clone with high gene expression levels of PD-1, CD103, and CD39 (Fig. 6G, H), consistent with the phenotype of tumor-specific exhausted T cells^37,38^. This clone was the most expanded clone in the tumor on day 21 and persisted through day 42 (Fig. 6I). In the peripheral TCR-seq data, this clone was not detected on day 0 and became detectable on day 42, but it was no longer detectable on day 63 (Fig. 6J). These findings are consistent with a tumor-specific T cell clone that was generated post-therapy and successfully seeded the peripheral repertoire, but this systemic component was not sustained.

## Discussion

This phase I study evaluated the safety and efficacy of the CCL21-DC vaccine and pembrolizumab in patients after progression on ICIs for advanced-stage NSCLC, or after progression on TKIs for patients with actionable *ALK*/*EGFR* alterations. The treatment demonstrated a favorable safety profile with no DLTs and a low rate of trAEs, and the study supported the feasibility of this combination approach. Patient accrual was relatively slow due to the complexity of the trial procedures as well as the COVID-19 pandemic, but the CCL21-DC vaccine series was successfully manufactured and delivered to patients without major treatment delays. The trial did not meet its primary efficacy endpoint as no objective responses were observed, though there was a 36.8% DCR.

The treatment landscape for advanced NSCLC in the second and later lines of therapy remains limited^6^. Most patients on this trial had previously progressed on ICIs and would have been unlikely to benefit from additional treatment with ICIs, with prior studies suggesting a response rate of up to 8% in this setting^39^. Similarly, the three patients with sensitizing *EGFR* mutations would not be expected to derive benefit from ICIs at any line of therapy^40^. The enrolled cohort thus represented a population typically considered to be refractory to standard immunotherapies, and novel therapeutic strategies are needed to stimulate an effective antitumor response in these patients. This study did not support the hypothesis that the CCL21-DC vaccine would overcome ICI resistance in this setting.

To fully characterize the immunologic effect of this therapy, we performed comprehensive immune monitoring studies on longitudinal biopsies of patient tumors. Samples from patients with local tumor control exhibited a decrease in mutational tumor heterogeneity, suggesting that there was more effective immunoediting and clonal pruning in those tumors. These samples also exhibited an increase in TCR diversity, with MIF studies suggesting that this change was driven at least in part by greater CD4+ T cell infiltration. These findings are consistent with prior studies showing that intratumoral T cells induced by other cancer vaccines are heavily comprised of CD4+ cells^41,42^. Our results also corroborate multiple studies demonstrating an ability of DC vaccines to promote T cell infiltration and, at least initially, alter the tumor immune microenvironment^11,19^. We find it intriguing that the patients with the most compelling clinical responses (such as a patient with over 12 months of SD and another with a response in an untreated intracranial lesion) had the greatest changes in immune metrics, such as TCR diversity. However, it remains unclear whether any associations between clinical outcomes and immunologic findings represent true mechanistic links, since the study did not have a control arm and short-term disease control may instead reflect underlying disease biology.

Many of the novel clones in our study clustered with *CCR7*+ naive/early memory T cells, which potentially could have been recruited to the tumor via a vaccine-induced CCL21 chemokine gradient. The post-therapy increase in T cell infiltration within a given tumor, in fact, correlated with the CCL21 secretion measured in the specific CCL21-DC vaccine batch used to inject that tumor. Due to the short half-life of both DCs and CCL21, post-injection biopsies were unable to provide direct measurements of therapy-induced CCL21 in the tumor. However, our prior pre-clinical studies in murine models demonstrated robust increases in intratumoral CCL21 peaking on day 2 after injection with CCL21-transduced DC vaccine^22^. These studies further showed that this therapy elicited greater T cell infiltration in comparison to a mock-transduced DC vaccine, and that this difference was more pronounced for CD4+ T cells^22^. Overall, our pre-clinical and correlative studies support the chemoattractant role of the CCL21-DC vaccine as an essential component of its immunologic effect.

The increased T cell infiltration observed after CCL21-DC vaccine therapy did not translate to clinical efficacy, potentially due to the transient nature of the immunologic changes induced by the vaccine. Notably, the majority of novel clones on day 21 did not persist on day 42. This result could in part be explained by sampling variation of relatively rare clones, but it is also consistent with the anticipated behavior of clones that are not specific for tumor antigens. Migration of CCR7+ T cells from the peripheral circulation along a CCL21 gradient would likely not select for tumor-specific clones, and these bystander clones, which are known to be pervasive in tumors^38^, would be susceptible to neglect and clonal loss. Indeed, an initial increase in T cell infiltration did not lead to a persistent cytotoxic CD8+ T cell signature in many patients. Meanwhile, novel clones that persisted through day 42 were more likely to undergo clonal expansion, shift towards effector or exhausted phenotypes, and express gene signatures associated with tumor-specific T cells. These persistent clones exhibited greater expansion within SD samples and may represent more tumor-relevant cells, but they were overall low in number. In summary, the CCL21-DC vaccine demonstrated an ability to induce T cell infiltration and perturb clonal dynamics, but these changes were largely transient and likely were predominantly non-specific.

The factors limiting clonal persistence and expansion in response to the CCL21-DC vaccine would represent major resistance mechanisms to this therapy. The SD group included a high proportion of *KRAS-*mutated tumors, including those with *STK11* and *KEAP1* pathogenic variants, which have been reported to be associated with poor outcomes to immunotherapy^43,44^. Resistance mechanisms to the CCL21-DC vaccine, thus, may not align perfectly with mechanisms associated with ICI resistance. However, our data ultimately do not elucidate whether associations with genomic variants reflect tumor biology or an interaction with the trial therapy.

The scRNA-seq data suggested that immunoregulatory signals in the TME may play a role in modulating outcomes. Myeloid cell profiling revealed enrichment of immunosuppressive IL1β+ and SPP1+ macrophages, which have been implicated in T cell exclusion and dysfunction^28,45^. Ligand-receptor interaction inference showed that SPP1 signaling between myeloid and T cells was observed more frequently than pro-inflammatory signaling, such as through the CXCL9/10-CXCR3 axis. Our immune monitoring studies contrast with preclinical data demonstrating that the CCL21-DC vaccine has the ability to reprogram the TME, including the myeloid compartment^22^. We speculate that the DC vaccine in patients may have been limited by the severity of immunosuppressive signaling in advanced, treatment-refractory tumors. Although MoDCs consistent with our vaccine product were identified in scRNA-seq data, they were largely apoptotic and would not be expected to maintain functionality 21 days after injection, and we thus were unable to glean insights regarding their direct interaction with other cellular components. There were few DCs captured by our data in general, but future studies could assess the effect of the CCL21-DC vaccine on other intratumoral DCs, as several DC markers and phenotypes have been linked to immunotherapy efficacy^46,47^.

Given the importance of TME factors, DC vaccines might be deployed most effectively as a personalized therapy tailored to the tumor characteristics of each patient. For example, DCs engineered to secrete CXCL9 and CXCL10 have been shown to initiate a robust immune response in ICI-resistant murine NSCLC models^48^, and we hypothesize that this therapy could be clinically useful in patients with CXCL9/10-deficient tumors, such as those with loss of chromosome 9p^49,50^. The DC vaccine represents a modular platform where the cytokine payload can be changed by modifying the contents of an adenoviral vector. In addition, our evidence for immunosuppressive myeloid signaling in the TME suggests a role for combination therapies. Therapies targeting tumor-associated macrophages, such as those against CSF1R^51^ or TREM2^52^, have been explored in ICI-resistant settings, though are not yet validated in the clinic. We also hypothesize that the DC vaccine may synergize with neoantigen-specific treatments, such as RNA neoantigen vaccines^53^ or cellular therapies^54^, to limit the recruitment of non-specific bystander T cells. These strategies may help address the “gap” in the cancer-immunity cycle^55^ that can arise between vaccine-mediated T cell infiltration and ICI-mediated T cell cytotoxicity.

Because the CCL21-DC vaccine is delivered locally to a primary lung tumor, its ability to affect the systemic immune response remains a critical question. Systemic responses were generally not observed in this clinical trial. Peripheral blood immune analyses revealed post-therapy changes, but these effects were more muted than in the tumor. Despite the presence of substantial T cell clonal dynamics in the tumor, peripheral blood samples showed minimal changes in TCR diversity or transcriptional profiles. These results further support the interpretation that the CCL21-DC vaccine was not able to robustly generate tumor-specific T cell clones, which would be expected to expand and manifest in the systemic TCR repertoire. Preclinical studies have shown that systemic efficacy with DC vaccines relies on effective trafficking to and from the tumor-draining lymph node^22,56,57^, but we were unable to assess this component of the immune response due to tissue constraints.

Several limitations of this study should be acknowledged. First, the relatively small cohort size and variability in tissue quality limited the number and statistical power of sample analysis. This study did not include control arms, and thus, immunologic findings remain correlative rather than mechanistic in nature. Heterogeneity in the DC product, particularly with regard to CCL21 secretion, may have introduced variability in immunologic responses across patients and underscores the need for improved techniques in cellular vaccine manufacturing^23,58^. We also did not directly corroborate the neoantigen-specificity of any T cell clones, limiting our ability to ascertain which immunologic changes are clinically relevant. Future studies may include validation of T cell specificity, such as by assessing stimulation of cloned TCRs by predicted neoantigens^59,60^. Studies with larger cohorts, mechanistic assays, and enhanced spatial and functional resolution will be critical to validate and extend these observations. These additional efforts may illuminate strategies for augmenting DC vaccine therapy to overcome the immunosuppressive features that limit the durability and depth of immunologic response. Optimization of DC vaccine characteristics, such as the cell source and cytokine payload, and an improved understanding of interactions between DC vaccines and the TME will be necessary to unlock the potential benefits of these therapies.

## Methods

### Clinical study design and procedures

This was a phase I, single-center, non-randomized study of the CCL21-DC vaccine in combination with pembrolizumab in patients with advanced NSCLC. The study was conducted in accordance with the protocol, the International Council for Harmonization Good Clinical Practice guidelines, and the Declaration of Helsinki. The study protocol received approval from the UCLA Institutional Review Board, and all patients provided written informed consent. The study was registered at ClinicalTrials.gov on May 23, 2018 (identifier: NCT03546361). Patient enrollment occurred from July 8, 2019, to December 11, 2023.

Eligible patients were aged 18 years and older with pathologically confirmed advanced/metastatic NSCLC. Patients without *EGFR* or *ALK* alterations were required to have progressed after at least one prior PD-1 or PD-L1 inhibitor. Those with sensitizing *EGFR* or *ALK* alterations were required to have progressed on at least one prior tyrosine kinase inhibitor (TKI), including osimertinib for patients with *EGFR* T790M mutations and a second-generation ALK inhibitor for those with *ALK* gene rearrangements. Patients must have had an Eastern Cooperative Oncology Group performance status of 0–1, measurable disease per Response Evaluation Criteria in Solid Tumors (RECIST) v1.1, adequate organ function, and a tumor lesion that could be accessed bronchoscopically or by computed tomography (CT)-guided transthoracic injection. Exclusion criteria included respiratory failure, clinically active brain metastases (defined as untreated and symptomatic, or requiring therapy with steroids or anticonvulsants to control symptoms), and a history of autoimmune disease requiring immunosuppressive agents.

The study design included dose escalation and expansion phases. The dose escalation followed a standard 3 + 3 design with the rationale of monitoring for potentiation of immune-related toxicities arising from the novel combination with an ICI. The CCL21-DC vaccine was administered into a lung tumor by either bronchoscopy or CT-guided transthoracic injection on days 0, 21, and 42, and the same tumor was injected at each time point. Patients received a dose of 1 × 10^7^ cells/injection in the first cohort of dose escalation and 3 × 10^7^ cells/injection in the second cohort, which were the 2 highest dose levels in the prior phase I trial of the CCL21-DC vaccine^19^. The dose of 3 × 10^7^ cells/injection was selected for the expansion phase. Pembrolizumab was administered intravenously at a dose of 200 mg every 3 weeks for up to 1 year or until disease progression or unacceptable toxicity.

### Generation of the CCL21-DC vaccine

The CCL21-DC vaccine was manufactured and tested as detailed previously^18,23^. Briefly, an adenoviral vector for CCL21 (AdCCL21) was manufactured by the Biopharmaceutical Development Program at SAIC-Frederick (Frederick, MD) following FDA Good Manufacturing Practice (GMP) standards. All patients underwent leukapheresis, and autologous blood was processed to isolate peripheral blood mononuclear cells (PBMCs). The PBMCs were cultured in the presence of granulocyte-macrophage colony-stimulating factor (GM-CSF) and interleukin-4 (IL-4) for 6–7 days to promote DC differentiation as previously described. DCs were then harvested and transduced using the AdCCL21 vector at 100:1 multiplicity of infection. For the first four patients, CCL21-DC vaccines were freshly manufactured for each of the three intratumoral injections. After quality control studies demonstrated minimal effect of a freeze-thaw cycle on DC vaccine phenotype^23^, subsequent CCL21-DC vaccines were cryopreserved in aliquots and thawed for each injection. Two patients required a second leukapheresis session to obtain sufficient cells for all three injections, which was allowed per protocol. CCL21 secretion by transduced DCs was confirmed by ELISA as previously reported^23^. Biosafety tests, including sterility and endotoxin assays, were performed prior to administering the product to patients.

### Clinical end points and assessments

The primary objectives of the study were to assess safety, including determination of the MAD, and to evaluate the ORR. The secondary objectives were to assess the AE profile as well as to determine drug target activity by analyzing serial pre- and post-treatment biopsies and blood specimens. AEs were graded according to the National Cancer Institute Common Terminology Criteria for Adverse Events (CTCAE) v5.0. trAEs were defined as AEs possibly related to either the CCL21-DC vaccine and/or pembrolizumab. Tumor response was evaluated according to RECIST v1.1 guidelines. Additional efficacy endpoints included DCR, progression-free survival (PFS), and OS. Local tumor stability was evaluated as a post-hoc outcome, defined as an increase in tumor diameter <10% in the injected lesion at best response.

The data cutoff for clinical results was June 1, 2024. Efficacy was evaluated only for patients who received at least two doses at the MAD of the CCL21-DC vaccine (3 × 10^7^ DCs per injection). The first efficacy assessment was performed at 9 weeks in order to first complete the CCL21-DC vaccine series and allow sufficient time for an immune response, followed by repeat CT imaging every 12 weeks. AEs were evaluated continually and reported for all patients who received at least one dose of the CCL21-DC vaccine. ORR was analyzed using one-sample exact binomial test. PFS and OS were analyzed by the Kaplan–Meier method, and median event time with two-sided 95% CI was calculated using the Brookmeyer and Crowley method. A sample size of 16 patients was estimated to provide 82.9% power to detect an absolute difference of 25% in ORR from the null hypothesis of 8% to the alternative hypothesis of 33%, using a two-sided one-sample exact binomial test at a 10% significance level.

### Patient sample preparation

Tumor biopsies were obtained on days 0, 21, and 42 at the time of each CCL21-DC vaccine injection. For each time point, at least 2 core biopsy samples were obtained from each of four quadrants of the target tumor using a 17–18 G needle.

One set of biopsies from each quadrant was reserved for single-cell dissociation. These samples were minced and then digested in RPMI media containing 2 mg/mL collagenase type I (Worthington), 2 mg/mL elastase (Worthington), and 0.5 mg/mL DNase I (Worthington). Samples were incubated in the digestion mix for 30 min at 37 °C and then strained through a 70 μm cell filter. Cells were then incubated in red blood cell lysis buffer (Invitrogen) for 1–3 min on ice before washing with phosphate-buffered saline (PBS). Cells were counted and resuspended in CryoStor CS10 medium (Biolife Solutions) for cryopreservation and storage in liquid nitrogen. The other sets of biopsy tissue were fixed in formalin for 24 h and then stored in 70% ethanol prior to being embedded into paraffin.

Peripheral blood samples were collected in Vacutainer CPT tubes (BD Biosciences) and processed to obtain PBMCs. Samples were centrifuged at 1800 × *g* for 20 min at room temperature. The plasma fraction was saved, and the remaining buffy coat was transferred to fresh tubes prior to repeat centrifugation. Cells were washed twice in PBS, and after cell counting, samples were frozen in heat-inactivated human AB serum plus 10% DMSO.

### Whole exome sequencing (WES)

Paraffin embedded tissue was cut onto polyethylene napthalate (PEN)-membrane slides at a thickness of 20 μm. Hematoxylin and eosin (H&E)-stained slides were generated in parallel for each sample and reviewed by a pathologist to demarcate areas of tumor. Samples on PEN slides were heated to 55 °C, deparaffinized in two changes of xylene, and washed in decreasing concentrations of ethanol. Slides were then stained with the Arcturus Paradise PLUS (Thermo Fisher) reagent. The LMD7000 Laser Microdissection System (Leica) was used to dissect out tumor tissue from deparaffinized slides based on the pathologist annotations. Genomic DNA was extracted using the AllPrep DNA/RNA Kit (QIAGEN). Library construction was performed by the UCLA Technology Center for Genomics & Bioinformatics (TCGB) core using the KAPA HyperCap Workflow Kit v3 (Roche). Sequencing was completed at a target depth of 200X using a Novaseq X Plus (Illumina) instrument.

Processing and analysis of sequencing was performed as previously described^61,62^. Briefly, sequencing reads were first aligned to the human reference genome GRCh38p13 following the steps recommended by the GATK Best Practice pipeline^63^, and then re-aligned around indel areas using the ABRA algorithm^64^. Quality control assessments were implemented to include only samples with at least 70% target regions with a minimal depth of coverage >20X. A mutation was called if it was identified by two mutation call algorithms, Strelka2 and Mutect2^65,66^. A second round of forced mutation calling was performed, in which a mutation that met statistical criteria for one sample would be called for all other samples from that patient. Mutations were annotated by the Ensembl Variant Effect Predictor^67^. Tumor heterogeneity was calculated using the MATH score^68^.

### cfDNA analysis

The alignment of raw sequencing reads followed the GATK pipeline described above. Tumor mutations were identified in plasma samples using cfSNV^69^. Mutations were excluded if there was at least one variant supporting read in the matched PBMC sample. A mutation was selected as a marker if its variant allele frequency (VAF) was >25% of the average of the five highest VAFs in the sample. At least 10 markers were required from a plasma sample to obtain a robust prediction. The tumor fraction was quantified in the plasma sample using the identified markers following the cfTrack pipeline^70^.

### MIF analysis

MIF was performed with Opal fluorophores (Akoya Biosciences) and data acquired using the Ventana Discovery Ultra (Roche) instrument as previously described^48,71^. Briefly, samples were included for analysis if H&E staining of adjacent tissue sections showed at least 10% tumor content as determined by a pathologist. Tissue sections of 5 μm thickness were deparaffinized using EZ-Prep reagent (Roche), followed by antigen retrieval in CC1 buffer (Roche). Discovery Inhibitor (Roche) was applied to inhibit enzymatic activities, followed by 6 sequential rounds of staining. Each round included the addition of a primary antibody followed by detection using the OmniMap secondary antibody (Roche). The primary antibodies were against pan-cytokeratin (PanCK, 1:100 dilution, Agilent #M351501–2), CD3 (no dilution, Ventana Medical Systems #790-4341), CD8 (1:200, Leica Biosystems #CD8-4B11-L-CE), FOXP3 (1:200, Cell Signaling Technology #98377), granzyme B (1:200, Leica Biosystems #NCL-L-GRAN-B), and Ki67 (1:200, Ventana Medical Systems #790-4286). Heat-induced epitope retrieval was applied each round to remove the primary-secondary-HRP antibody complexes before staining with the subsequent antibody. The primary antibodies and fluorophores were stained with Opal 480, 520, 570, 690, 780, or TSA-DIG (Akoya Biosciences) at a dilution of 1:400, except for Opal 780, which was used at a dilution of 1:50. The slides were then counterstained with Spectral DAPI (Akoya Biosciences) and mounted with ProLong Diamond antifade medium (Thermo Fisher Scientific).

Stained slides were imaged using the Vectra Polaris imaging system (v1.0, Akoya Biosciences). Slide scans were then imported into HALO (v3.6, Indica Labs) for stitching, selecting regions of interest, cell segmentation, and signal quantification. Cells without DAPI expression were filtered out, and individual cells were annotated manually based on marker colocalization to identify populations of CD8+ T cells (CD3+ and CD8+), CD4+ T cells (CD3+ and CD8-), Tregs (CD3+, CD8-, and FOXP3+), and epithelial cells (PanCK+). Cell density was calculated within tissue area (i.e., excluding non-tumor or non-stromal glass area). Neighborhood analysis was performed using on a previously reported approach^72^ by applying a radius of 50 μm around each tumor cell centroid and filtering for tumor cells with at least four neighbor cells.

### scRNA/TCR-seq of tumor samples

Single-cell dissociated tumor samples were thawed and stained using the Zombie NIR Fixable Viability Kit (Biolegend). The KPL mouse cell line^61^ was simultaneously stained with Zombie NIR, and 20,000 live sorted cells were mixed with tumor samples to act as carrier cells. Viable cells were sorted using a FACSAria III flow cytometer (BD Biosciences), centrifuged at 400 × *g* for 5 min, and resuspended in PBS + 5% fetal bovine serum (FBS) at a concentration of ~1500 cells/μL. Single-cell library construction was performed at the UCLA TCGB core using the Next GEM or GEM-X Single Cell 5′ platform with V(D)J Amplification (10× Genomics) to capture a target of 10,000–20,000 cells per sample based on the manufacturer’s protocols. Libraries were sequenced using a Novaseq X Plus (Illumina) instrument with paired-end sequencing at a target depth of 20,000 reads per cell.

### scRNA-seq of PBMCs

PBMCs from days 0, 21, 42, and 63 for each patient were tagged using the CellPlex Kit (10× Genomics) per manufacturer’s protocols. The cells were pooled and then stained with Zombie NIR prior to flow sorting for viability on a FACSAria III flow cytometer. Library construction was performed at the UCLA TCGB using the Next GEM 3′ Single Cell platform with 3′ Feature Barcode kit (10× Genomics) to capture a target of 30,000 cells per sample based on the manufacturer’s protocols. Library sequencing was performed as described above using a Novaseq X Plus (Illumina) instrument.

### Data analysis for scRNA/TCR-seq

CellRanger 9.0 (10× Genomics) was used to align raw reads to the GRCh38 human and GRCm39 mouse combined reference genome provided by 10× Genomics, yielding raw count matrices based on unique molecular identifiers (UMIs). Further processing was performed following published algorithms^73^. UMI matrices were filtered using the emptyDrops function in the DropletUtils (v1.26.0) package^74^ to remove low quality cells by ranking barcodes and predicting empty droplets with a filter of false discovery rate (FDR)-adjusted *p*-value < 0.001. Species deconvolution was performed by obtaining the sum of human and mouse gene counts, and cells with >75% of counts in human genes were deemed to be human cells. Poor quality cells were removed as defined by cells with mitochondrial content >10% and a low number of detected genes. The threshold for detected genes was determined separately for each sample, with 250 genes serving as the default level but adjusted using histogram analysis to select inflection points. Genes expressed in less than five cells were excluded. After quality control steps, samples were integrated into a single dataset for downstream analysis. Patients with only one time point available were not included in the final dataset. TCRs were extracted from V(D)J-amplified sequencing data and integrated with scRNA-seq data using the scRepertoire (v2.2.1) package^75^.

Subsequent processing was performed using the scran (v1.34.0)^76^ and scater (v 1.34.1)^77^ packages. Specifically, data normalization was performed by deconvolution of pool-based size factors in scran. The top 10% of highly variable features were selected using modelGeneVar and getTopHVGs. Principal component analysis (PCA) was performed using runPCA in scater. Data from all samples were then integrated using Harmony (v1.2.4)^78^ to create batch-adjusted principal components. Visualization was performed by uniform manifold approximation and projection (UMAP) using runUMAP. Cells were clustered by creating shared nearest-neighbor graphs with buildSNN and applying an Infomap clustering algorithm. Cell clusters were first annotated based on broad cell type markers (*CD3D/CD8A* for T cells, *NCAM1/FCGR3A* for NK cells, *MS4A1/CD19* for B cells, *MZB1/JCHAIN* for plasma cells, *CD14*/*CD68* for macrophages/monocytes, *BATF3/CD1C* for DCs, *CD209* for MoDCs, *CPA3* for mast cells, *EPCAM*/*CEACAM6* for tumor, and collagen genes, such as *COL1A1* for stromal cells). Clusters strongly expressing lineage markers from more than one cell type were considered to be doublets and removed from further analysis. Subclusters within each cell type were annotated by assessing DEGs identified by scoreMarkers.

Differential gene expression was performed on pseudobulk samples using DESeq2 (v1.46.0), and samples with pseudobulk groups containing fewer than five cells were excluded from the analysis^79^. Gene set enrichment analysis (GSEA) for Gene Ontology (GO) terms was identified using gseGO in the clusterProfiler (v4.14.6) package^80^ using a *p*-value of 0.05 as the cutoff for significance. Cells with high expression of neoantigen-specific T cell gene signatures were identified using the AUCell (v1.28.0) package to set global thresholds for area under the curve expression levels^81^. The gene signature reported by Zeng et al. was assessed using non-imputed MANAscores as described in their manuscript^25^. Viral clonotypes were identified as those with TCR-beta sequences matching those annotated as viral in the VDJdb database^27^. Ligand-receptor interaction reference was performed using the CellChat (v2.2.0) package^82^. Only samples with >10 cells within T cell and macrophage clusters, and with at least 5 clusters represented by >5 cells, were included for analysis. Communication probabilities were calculated for each sample individually and then merged for the full data set. Analysis of the tumor TCR repertoire, including TCR diversity were performed using the scRepertoire package, while the peripheral TCR repertoire was analyzed using the immunarch (v0.10.3)^83^ package.

### Bulk-TCR sequencing

PBMCs were thawed in RPMI + 10% FBS media. Cells were then stained with antibodies against CD3 (Biolegend, clone OKT3), CD4 (Biolegend, clone RPA-T4), and CD8 (Biolegend, clone RPA-T8) as well as Zombie NIR. Sorting was performed on a FACSAria III flow cytometer to isolate CD4+ and CD8+ T cells. Genomic DNA was extracted from the resulting samples using the DNeasy Blood & Tissue Kit (QIAGEN). TCR-β complementarity determining region 3 (CDR3) sequencing was performed with the immunoSEQ platform (Adaptive Biotechnologies).

### Cytokine measurements and analysis

Cytokine and chemokine levels in blood plasma samples were measured with the Milliplex Human Cytokine/Chemokine/Growth Factor Panel A (Millipore) and the Human Cytokine/Chemokine Magnetic Bead Panels II, III, and IV (Millipore), using the manufacturer’s protocols. The 54 analytes in these panels were: soluble CD40L, colony stimulating factor 1, eotaxin, epidermal growth factor, fibroblast growth factor 2, FMS-like tyrosine kinase 3 ligand, G-CSF, GM-CSF, interferon (IFN)-α2, IFN-β, IFN-γ, IL-1α, IL-1β, IL-1RA, IL-2, IL-3, IL-4, IL-5, IL-6, IL-7, IL-8/C-X-C motif chemokine ligand 8 (CXCL8), IL-9, IL-10, IL-12p40, IL-12p70, IL-13, IL-15, IL-17A, IL-17E/IL-25, IL-17F, IL-18, IL-22, IL-27, CCL2, CCL3, CCL4, CCL7, CCL18, CCL19, CCL20, CCL22, CX3CL1, CXCL1, CXCL9, CXCL10, CXCL11, platelet-derived growth factor (PDGF)-AA, PDGF-AB/BB, transforming growth factor (TGF)-α, TGF-β, tumor necrosis factor (TNF)-α, TNF-β, vascular endothelial growth factor, and X-C motif chemokine ligand 1 (XCL1). Differences in baseline levels of each cytokine between the SD and progressive disease (PD) groups were analyzed by the Wilcoxon test. Therapy-induced changes over time were modeled by a linear mixed effects model adjusted for repeated measure, disease status, time in week and the interaction of time and disease status, allowing for different slopes.

### Statistical analysis

Statistical analyses were performed in R (version 4.4.2) for scRNA/TCR-seq and otherwise in Prism 9 (GraphPad). Results are reported as mean ± SD, unless indicated. Linear mixed effects were modeled in R using the lme4 (v1.1.37) package^84^. Correlations were assessed using Spearman’s rank correlation coefficient. Differences between groups were assessed using the Wilcoxon rank-sum test, two-tailed t-tests, or one-way or two-way ANOVA with correction for multiple comparisons as specified in the figure legends. Statistical significance is reported as *, *p* < 0.05; **, *p* < 0.01; ***, *p* < 0.001; and ****, *p* < 0.0001.

### Reporting summary

Further information on research design is available in the Nature Portfolio Reporting Summary linked to this article.

## Supplementary information

Supplementary Information

Description of Additional Supplementary Files

Supplementary Data 1–2

Reporting Summary

Transparent Peer Review File

## Source data

Source data

## Data Availability

The study protocol is available in the Supplementary Information file. Raw single-cell RNA sequencing data is deposited in the Sequence Read Archive under BioProject accession code PRJNA1348488. Processed data, including count matrices, are deposited in the Gene Expression Omnibus under accession codes GSE317309 and GSE345124. Tumor and peripheral blood WES data generated in this study have been deposited in the European Genome-phenome Archive (EGA) under accession code EGAS50000002127. This data can be made available through an approved Data Use Agreement for research purposes only. WES data cannot be made publicly available for three of the patients, as they did not sign a consent that explicitly allowed WES deposition. Additional data, including de-identified participant data, will be shared on reasonable request for non-commercial research purposes. Requests for de-identified individual participant data can be made to the corresponding author, and this data will be shared up to 5 years after publication through an approved Data Use Agreement. The remaining data are available within the Article, Supplementary Information, or Source Data file. Source data are provided with this paper.
