# Supplementary material for: A CCL21 gene-modified dendritic cell vaccine combined with pembrolizumab in advanced non-small cell lung cancer: a phase I trial
Source: Nat Commun. 2026 Oct 4;17:11321. doi: 10.1038/s41467-026-78130-7 (PMC13724721; doi:10.1038/s41467-026-78130-7)
Supplement: Supplementary file 1 [file 41467_2026_78130_MOESM1_ESM.pdf]

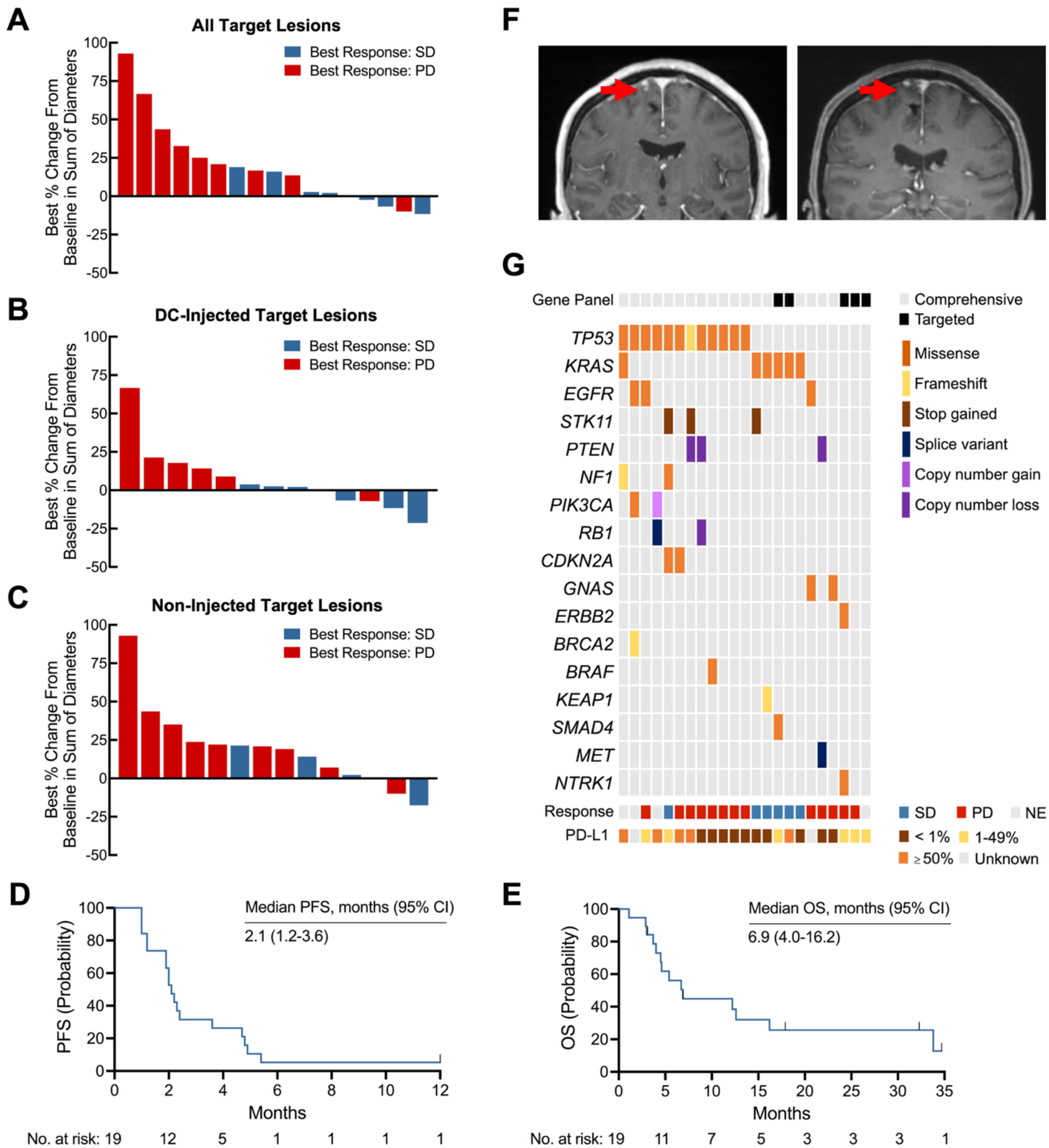

Supplementary Fig. 1. Clinical outcomes in the phase I trial of CCL21-DC vaccine and pembrolizumab combination therapy. (A-C) Waterfall plots showing best percent change in diameters in **(A)** all target lesions (n=17), **(B)** lesions injected with the CCL21-DC vaccine (n=13), and **(C)** target lesions not injected with the DC vaccine (n=14). Not all injected tumors were measurable target lesions. **(D)** Kaplan-Meier curve of progression-free survival (PFS) and **(E)**

overall survival (OS) in the efficacy-evaluable cohort. Number at risk at serial time points is designated along the bottom. Tick markers along the curves indicate censored data. OS censoring events at 3, 7, and 32 months represent loss to follow up. **(F)** Representative image of patient with untreated brain metastasis (left image, red arrow) at baseline, which resolved after starting the trial therapy in the absence of localized therapy such as radiotherapy (right image). **(G)** Oncoplot of somatic variants identified in tumor sequencing (n=23 patients). Genes with variants in at least 2 patients were included, as were those known to act as driver mutations or modulators of immune response. Top row shows gene panel used for sequencing – "targeted" denotes an in-house panel that only includes actionable genomic alterations, while "comprehensive" denotes larger panels performed via commercial assays. The bottom rows depict clinical response denoted by stable disease (SD), progressive disease (PD), or not evaluable (NE), as well as PD-L1 expression by immunohistochemistry. Source data are provided as a Source Data file.

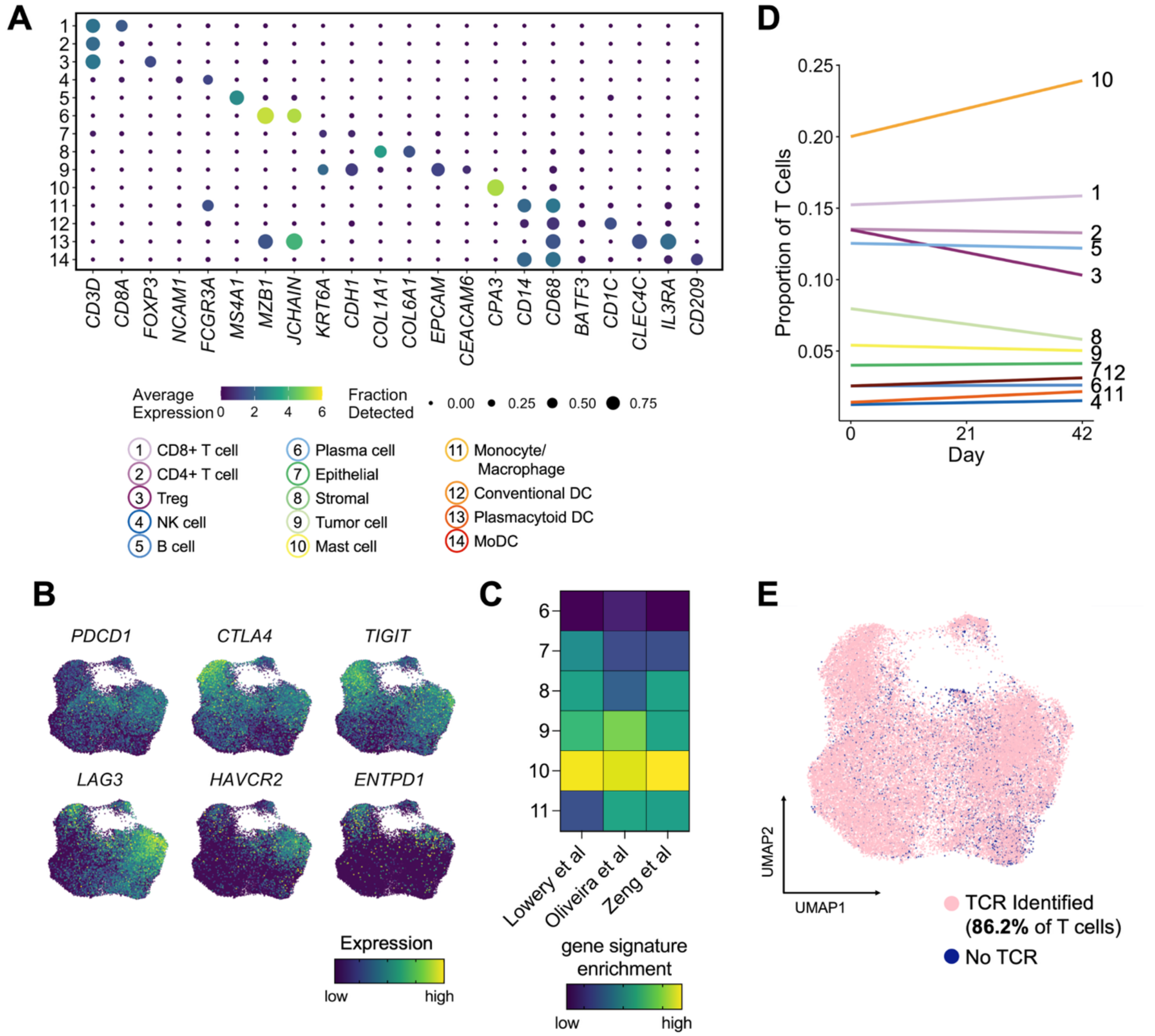

Supplementary Fig. 2. ScRNA/TCR-seq analysis of patient biopsies. (A) Dot plot (n=17 patients) showing expression of key marker genes used to determine cluster identities (as listed on the right). **(B)** Expression of exhaustion-related genes in T cells (n=17 patients) plotted onto UMAP space. **(C)** Heatmap showing relative proportion of cells (n=9 patients) within each CD8+ cluster that exhibit high expression of gene signatures previously validated to identify neoantigen-specific T cells. **(D)** Visualization of linear models of scRNA-seq T cell cluster proportions over time (n=17 patients), with none of the clusters exhibiting a statistically significant non-zero slope. **(E)** UMAP representation of T cells (n=17 patients) with TCRs successfully identified by simultaneous TCR-seq. Source data are provided as a Source Data file.

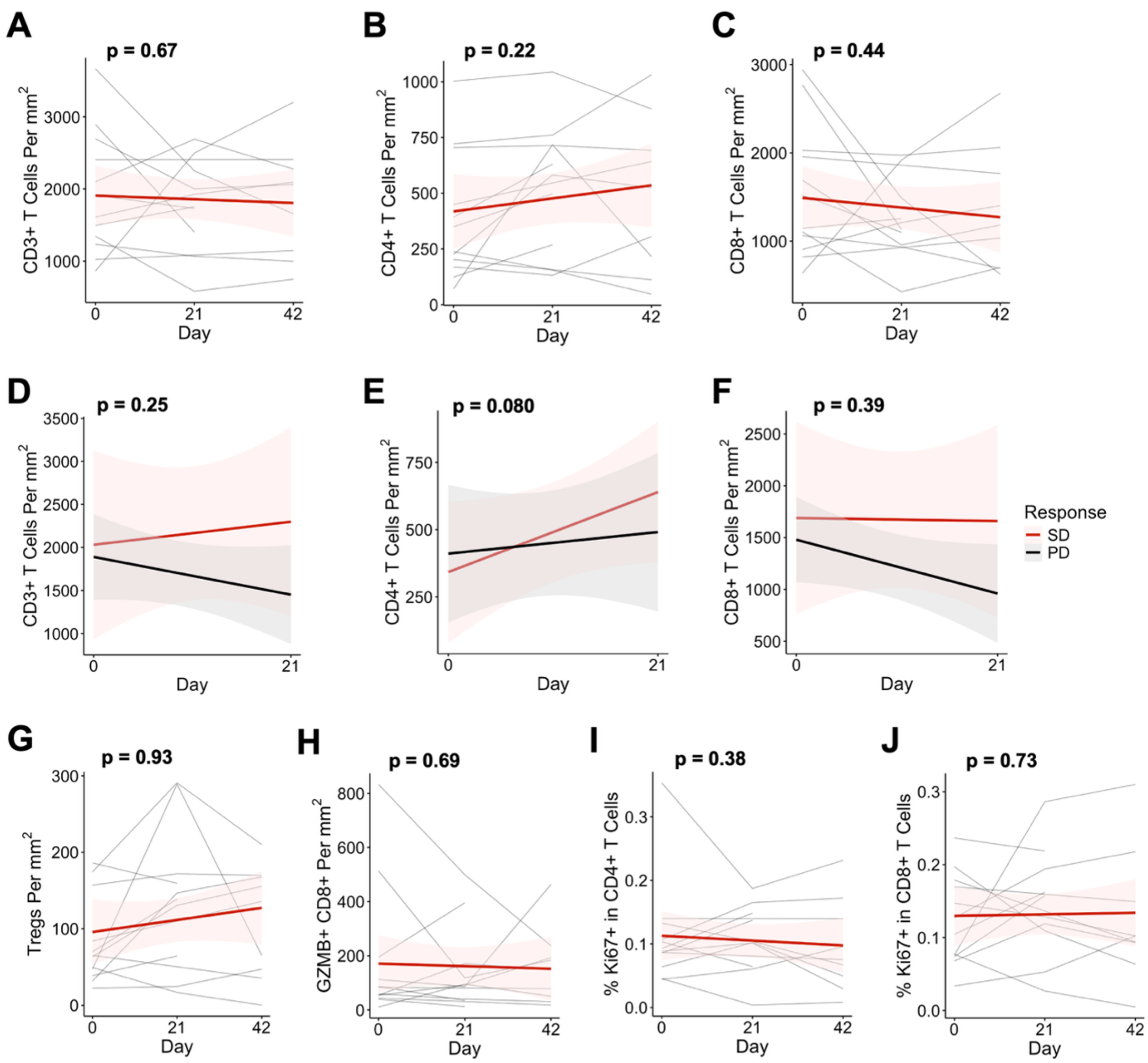

Supplementary Fig. 3. Patterns of post-therapy T cell recruitment into tumors. (A) Tumor infiltration by CD3+ T cells over time as assessed by multiplex immunofluorescence (n=12 patients), illustrated as individual tumors (grey lines) or linear models (red line with 95% confidence bands). **(B-C)** Same as in **A** but for **(B)** CD4+ T cells and **(C)** CD8+ T cells. **(D-F)** Difference in tumor infiltration over time separated by clinical outcomes (SD – stable disease, n=4 patients; progressive disease – PD, n=8 patients) for **(D)** CD3+ T cells, **(E)** CD4+ T cells, and **(F)** CD8+ T cells. **(G-J)** Tumor infiltration (n=12 patients) for **(G)** regulatory T cells (Tregs), **(H)** GZMB+ CD8+ T cells, **(I)** Ki67+ CD4+ T cells, and **(J)** Ki67+ CD8+ T cells. Source data are provided as a Source Data file.

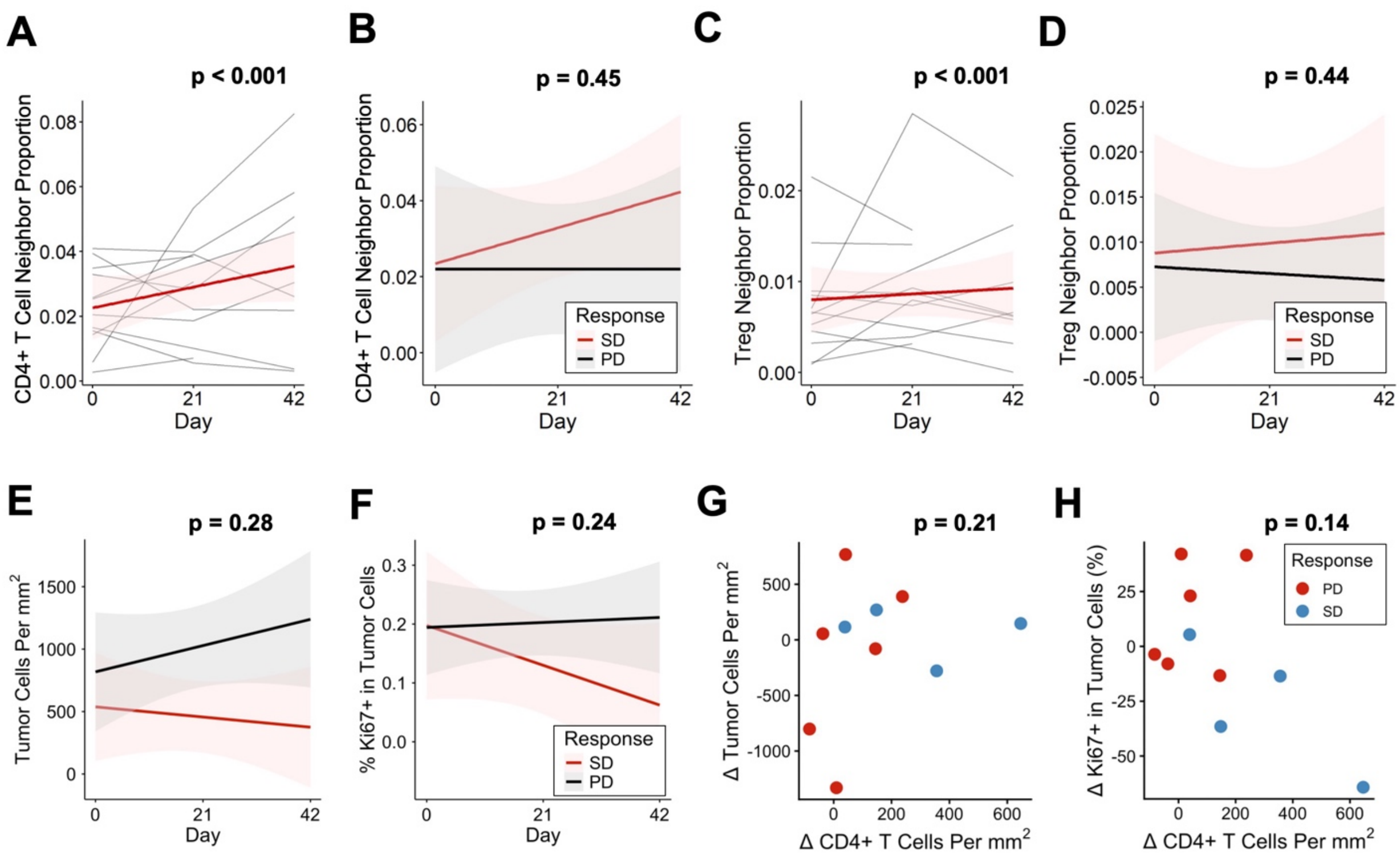

Supplementary Fig. 4. Spatial interactions between CD4+ T cells and tumor cells. (A) Proportion of CD4+ T cells in tumor cell neighborhoods (n=12 patients), defined as a 50 μm radius around each given tumor cell, over time. **(B)** Same data separated between stable disease (SD, n=4 patients) and progressive disease (PD, n=8 patients) groups and represented using linear models, with shading representing 95% confidence intervals. **(C)** Proportion specifically of Tregs in tumor cell neighborhoods (n=12 patients) over time overall and **(D)** separated between SD (n=4 patients) and PD (n=8 patients) groups. **(E)** Difference between trajectories of tumor cell density and **(F)** tumor Ki67 positivity over time (n=12 patients) compared between SD (n=4 patients) and PD (n=8 patients) groups. **(G)** Correlation between change in CD4+ T cell infiltration and either change in tumor cell density at day 21 (n=10 patients with samples at both time points) or **(H)** change in percent of Ki67+ tumor cells. Source data are provided as a Source Data file.

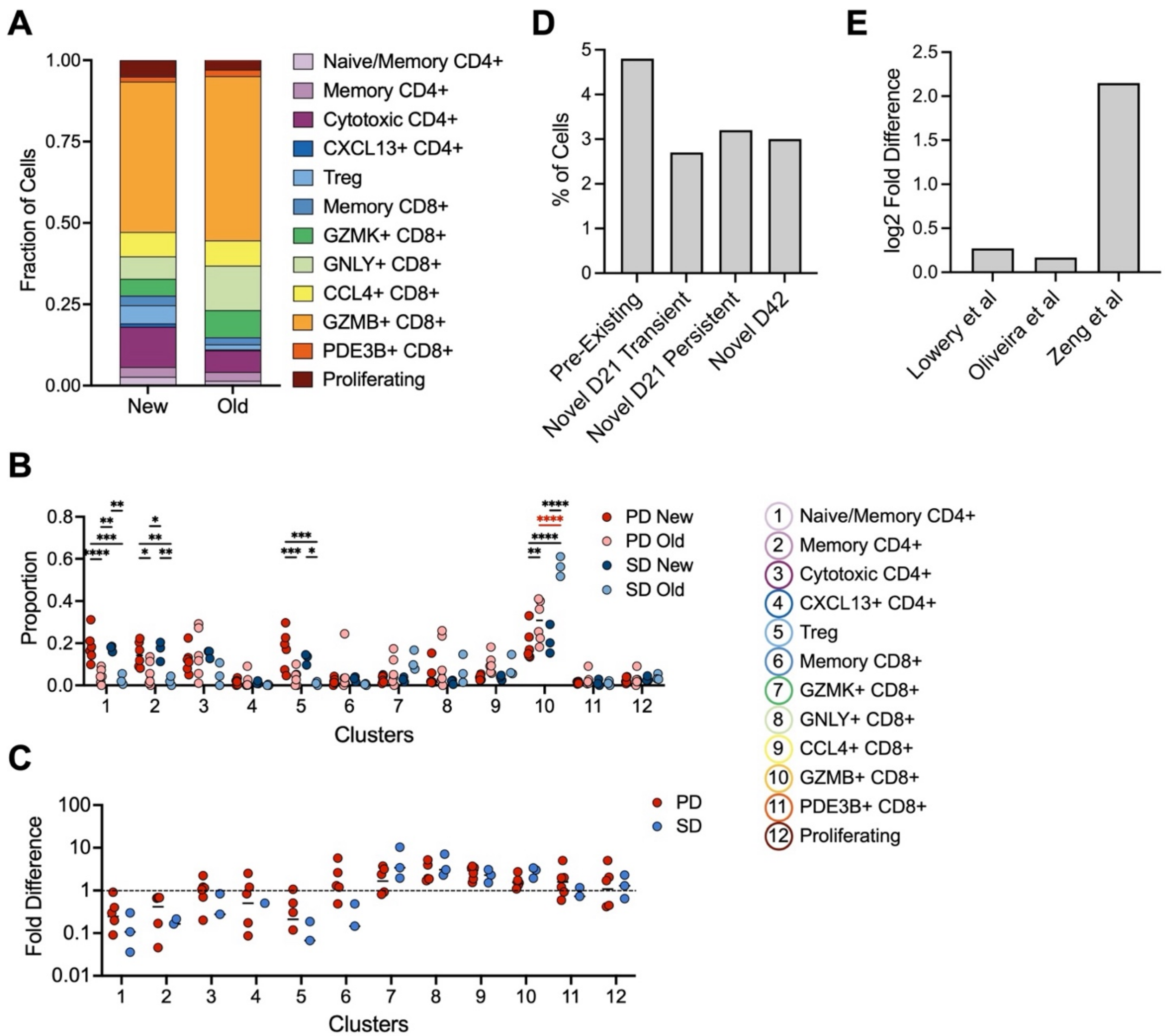

Supplementary Fig. 5. Characteristics of persistent and transient novel clones. **(A)** Proportion of scRNA-seq T cell clusters (n=9 patients) within new (novel) or pre-existing (old) clones after filtering for clones with at least 0.2% clonal proportion. **(B)** Baseline cluster distributions of clones separated based on clinical outcomes. Red-colored stars denote comparisons between SD old (n=3 patients) vs PD old (n=6 patients) clones. **(C)** Fold difference in cluster distribution of these clones, with higher ratios indicating greater prevalence in pre-existing clones. **(D)** Percent of T cells mapping to a database of viral clonotypes (n=9 patients). **(E)** Relative increase in expression of gene signatures associated with neoantigen-specific T cells within novel persistent clones compared to novel transient clones (n=9 patients). Comparisons made using two-way ANOVA with Tukey correction for multiple comparisons, * $p < 0.05$, ** $p < 0.01$, *** $p < 0.001$, **** $p < 0.0001$. Source data are provided as a Source Data file.

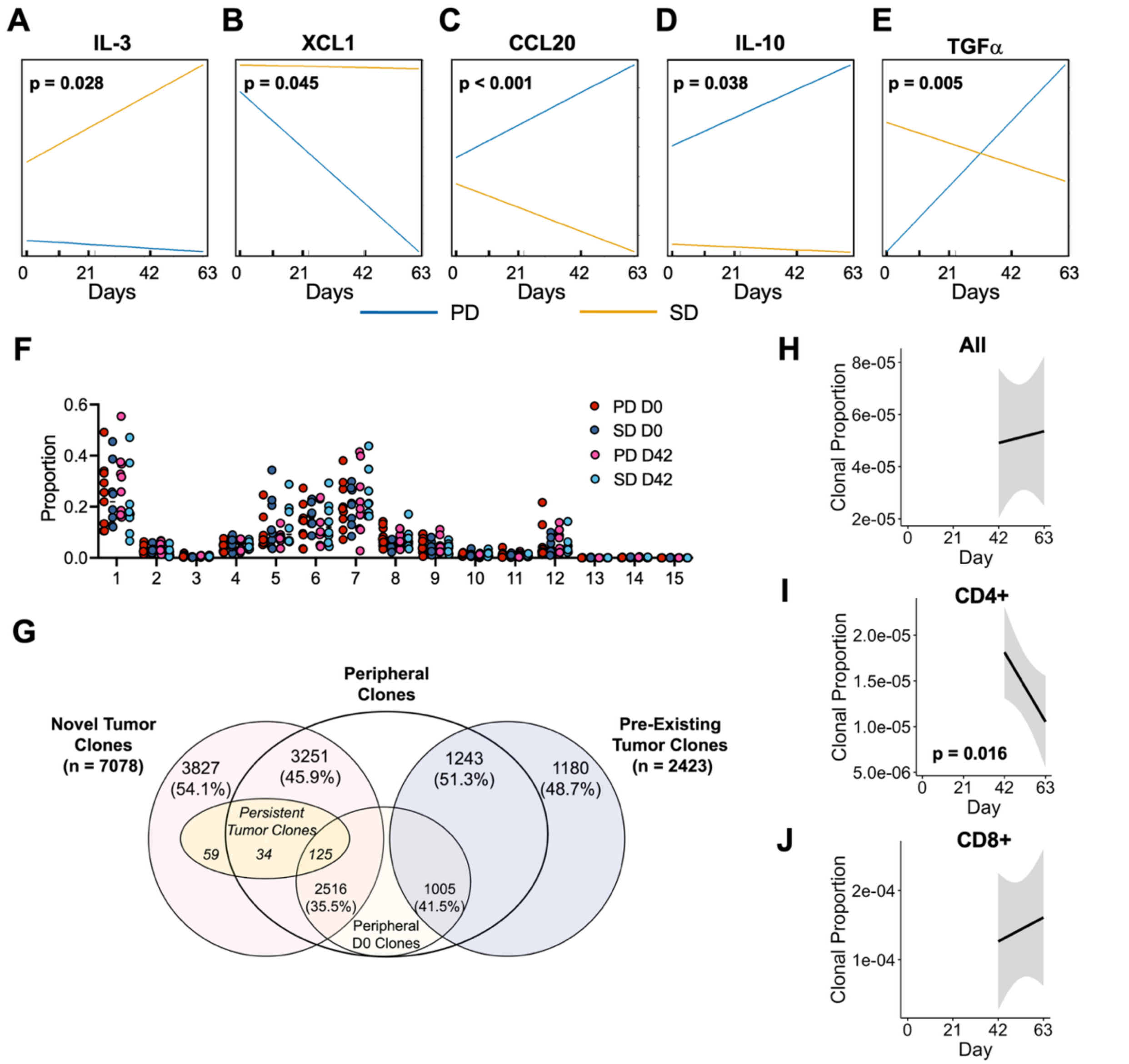

Supplementary Fig. 6. Systemic immunologic changes induced by the CCL21-DC vaccine and pembrolizumab. (A) Slopes of IL-3 levels in peripheral blood samples (n=23 patients) over time as determined by a linear mixed effects model of cytokine measurements by Luminex from progressive disease (PD, n=15 patients) versus stable disease (SD, n=8 patients) samples. **(B-E)** Same as in **A**, but for **(B)** XCL1, **(C)** CCL20, **(D)** IL-10, and **(E)** TGFα. **(F)** Cluster distributions of peripheral immune cells at day 0 and at day 42 (n=17 patients) separated between PD (n=9 patients) and SD (n=8 patients) groups. **(G)** Venn diagram illustrating overlap between novel or pre-existing intratumoral clones with the peripheral TCR repertoire (n=7 patients). **(H)** Trend in peripheral proportion of clones that emerged within the tumor post-therapy (n=7 patients), separated into **(I)** CD4+ and **(J)** CD8+ T cells. Source data are provided as a Source Data file.

Supplementary Table 1. Summary of adverse effects (AEs).

	All Patients (N = 23)			
	**Any grade, No. (%)**	**Grade 3, No. (%)**	**Grade 4, No. (%)**	**Grade 5, No. (%)**
**AEs of any cause**				
Any	19 (83)	2 (9)	2 (9)	1 (4)
Serious	9 (39)	2 (9)	2 (9)	1 (4)
Resulting in discontinuation	0	0	0	0
**Treatment-related AEs**				
Alopecia	1(4)	0	0	0
Diabetes mellitus	1(4)	1(4)	0	0
Fatigue	1(4)	0	0	0
Flank pain (injection-related)	1(4)	0	0	0
**AEs observed in ≥ 10% of patients**				
Dyspnea	8 (35)	0	0	0
Fatigue	7 (30)	0	0	0
Cough	4 (17)	0	0	0
Thromboembolic event	3 (13)	2 (9)	0	0
Flank pain	3 (13)	0	0	0
Abdominal pain	3 (13)	0	0	0
Rash	3 (13)	0	0	0
Pruritus	3 (13)	0	0	0

Supplementary Table 2. Efficacy of the study treatment.

Response	Efficacy-Evaluable Patients (N = 19)
ORR, No. (%)	0
DCR, No. (%)	7 (37)
SD, No. (%)	7 (37)
PD, No. (%)	12 (63)
PFS, months, median (range, 95% CI)	2.1 (1.2-12.0, 1.2-3.6)
OS, months, median (range, 95% CI)	6.9 (1.1-NE, 4.0-NE)

Abbreviations: ORR – objective response rate, DCR – disease control rate, SD – stable disease, PD – progressive disease, PFS – progression-free survival, OS – overall survival, CI – confidence interval, NE – not evaluable

Clinical Protocol. AM 8 Dated May 12, 2022

A phase I trial of intratumoral administration of CCL21-gene modified dendritic cell (Ad-CCL21-DC) combined with intravenous pembrolizumab for advanced NSCLC

Principal Investigator:
Edward Garon, M.D., M.S.
Associate Professor of Medicine
Director, Thoracic Oncology Program
2020 Santa Monica Blvd, Suite 600
Santa Monica, CA 90404
Phone: 310-586-2098
Email: egaron@mednet.ucla.edu

Immune Monitoring Lead:
Steven M. Dubinett, M.D.
Professor of Medicine
Chief, Division of Pulmonary and Critical Care Medicine
Director, UCLA Lung Cancer Research Program
37-131 CHS
Los Angeles, CA 90095
Phone: 310-794-6566
Fax: 310-267-2829
E-mail: sdubinett@mednet.ucla.edu

Pathology Lead:
W. Dean Wallace, M.D.
Associate Professor, Pathology and Laboratory Medicine
Chief, Pulmonary Pathology
13-145B CHS
10833 Le Conte Ave.
Los Angeles, CA 90095
Phone: 310-825-3719
Fax: 310-267-2058
Email: wwallace@mednet.ucla.edu

Radiology Lead:
Robert D. Suh, M.D.
Director of Thoracic Interventional Services
Professor of Radiology
1638 RRUMC
Los Angeles, CA 90095-7437
Phone: 310-267-8708
Fax: 310-267-3635
Email: rsuh@mednet.ucla.edu

Statistical Lead:
David Elashoff, Ph.D.
Professor of Biostatistics & Medicine
914 Broxton Ave., Ste 101

Los Angeles, CA 90024
Phone: 310-794-3346
Fax: 310-794-8149
Email: delashoff@mednet.ucla.edu

<u>Other Co-Investigators</u>:

Jonathan W. Goldman, M.D.
Assistant Professor, UCLA Hematology & Oncology
Associate Director of Drug Development
Director of Clinical Trials in Thoracic Oncology
2020 Santa Monica Blvd, Suite 600
Santa Monica, CA 90404
Phone: 310-829-5471
Fax: 310-829-6192
E-mail: jwgoldman@mednet.ucla.edu

Denise R. Aberle, MD
Vice Chair of Research in Radiological Sciences
UCLA Radiological Sciences
924 Westwood Blvd., #420
Los Angeles, CA
90095-7255
Phone: 310-794-8996
Email: daberle@mednet.ucla.edu

Scott Seung Oh, DO
Co-Director, Interventional Pulmonology
200 UCLA Medical Plaza
Suite 365-B
Los Angeles, CA 90095
Phone: 310-825-8061
E-mail: SSOh@mednet.ucla.edu

Igor Barjaktarevic, MD
Assistant Clinical Professor
200 UCLA Medical Plaza
Suite 365-B
Los Angeles, CA 90095
Phone: 310-825-8061
E-mail: IBarjaktarevic@mednet.ucla.edu

Aaron E. Lisberg, MD
Health Sciences Clinical Instructor
2020 Santa Monica Blvd.
Suite 600
Santa Monica, CA 90404
Phone: 310-829-5471
E-mail: alisberg@mednet.ucla.edu

Fereidoun Abtin, MD
Assistant Professor of Radiology

Ronald Reagan UCLA Medical Center
Suite 1621
Los Angeles, CA 90095
Phone: 310-267-8708
E-mail: fabtin@mednet.ucla.edu

Scott J. Genshaft, MD
Assistant Professor of Radiology
Ronald Reagan UCLA Medical Center
Suite 1638
Los Angeles, CA 90095
Phone: 301-267-8708
E-mail: sgenshaft@mednet.ucla.edu

Gregory A. Fishbein, MD
Assistant Clinical Professor
Ronald Reagan UCLA Medical Center
UCLA Pathology & Lab
CHS 1P-326
10833 Le Conte Ave.
Los Angeles, CA 90095
Phone: 310-206-4473
Email: gfishbein@mednet.ucla.edu

PROTOCOL SYNOPSIS

A phase I trial of intratumoral administration of CCL21-gene modified dendritic cell (Ad-CCL21-DC) combined with intravenous pembrolizumab for advanced NSCLC

<u>Hypothesis</u>:

- Intratumoral injection of autologous CCL21 gene-modified DC (Ad-CCL21-DC) when combined with intravenous pembrolizumab is safe
- Intratumoral injection of autologous CCL21 gene-modified DC (Ad-CCL21-DC) when combined with intravenous pembrolizumab can improve objective response rate in patients with advanced NSCLC
- Intratumoral injection of autologous CCL21 gene-modified DC (Ad-CCL21-DC) will improve tumor-specific immune responses unleashed by PD-1 blockade

<u>Primary Objective</u>

1. Dose Escalation: To determine the safety and maximum tolerated dose (MTD) of intratumoral injection of CCL21 gene-modified DC (Ad-CCL21-DC) when combined with intravenous pembrolizumab in either 1) patients with advanced non-small cell lung cancer (NSCLC) without a sensitizing epidermal growth factor receptor (EGFR) mutation and/or anaplastic lymphoma kinase (ALK) gene rearrangement who have received prior systemic anti-cancer therapy for advanced disease that includes a PD -1 and/or PD-L1 inhibitor or 2) patients who have sensitizing EGFR mutations and/or ALK gene rearrangements and have received prior tyrosine kinase inhibitor therapy.
2. Dose Expansion: To evaluate the objective response rate (ORR) in subjects treated with the dose established during dose escalation (ExD) of intratumoral injection of Ad-CCL21-DC when administered with intravenous pembrolizumab in either 1) patients with advanced non-small cell lung cancer (NSCLC) without a sensitizing epidermal growth factor receptor (EGFR) mutation and/or anaplastic lymphoma kinase (ALK) gene rearrangement who have received prior systemic anti-cancer therapy for advanced disease that includes a PD -1 and/or PD-L1 inhibitor or 2) patients who have sensitizing EGFR mutations and/or ALK gene rearrangements and have received prior tyrosine kinase inhibitor therapy..

<u>Secondary Objective</u>

- To define the adverse event (AE) profile of intratumoral injection of Ad-CCL21-DC (determined during dose escalation) when administered with intravenous pembrolizumab in either 1) patients with advanced non-small cell lung cancer (NSCLC) without a sensitizing epidermal growth factor receptor (EGFR) mutation and/or anaplastic lymphoma kinase (ALK) gene rearrangement who have received prior systemic anti-cancer therapy for advanced disease that includes a PD -1 and/or PD-L1 inhibitor or 2) patients who have sensitizing EGFR mutations and/or ALK gene rearrangements and have received prior tyrosine kinase inhibitor therapy., and to determine relationship of AEs to study treatment.
- To determine drug target activity by analyzing serial pre- and post-treatment biopsies and blood specimens of intratumoral injection of Ad-CCL21-DC (determined during dose escalation) when administered with intravenous pembrolizumab in either 1) patients with advanced non-small cell lung cancer (NSCLC) without a sensitizing epidermal growth factor receptor (EGFR) mutation and/or anaplastic lymphoma kinase (ALK) gene rearrangement who have received prior systemic anti-cancer therapy for advanced disease that includes a PD -1

and/or PD-L1 inhibitor or 2) patients who have sensitizing EGFR mutations and/or ALK gene rearrangements and have received prior tyrosine kinase inhibitor therapy.

Endpoints:

Primary Endpoint:

1. Dose Escalation: Establish maximum tolerated dose (MTD) / maximum administered dose (MAD) – the dose level below the one at which more than 1/6 patients experience dose-limiting toxicities (DLT), or Dose Cohort (2), if there is 0 or 1/6 DLT in the cohort.
2. Dose Expansion: Define ORR by RECIST v1.1 criteria

Secondary Endpoint(s):

1. Define toxicities – Describe the adverse events using CTC AEv5.0 to grade toxicities
2. Determine effects of treatment on drug target activity – Before each treatment/injections, CT or bronchoscope guided biopsies will be collected. Immunohistochemistry (IHC) will be conducted to assess biomarkers including PD-1, PD-L1, CD8, and CD4 expression.

Study Population:
Patients with pathologically confirmed and radiographically measurable stage IV NSCLC with either 1) advanced non-small cell lung cancer (NSCLC) without a sensitizing epidermal growth factor receptor (EGFR) mutation and/or anaplastic lymphoma kinase (ALK) gene rearrangement who have received prior systemic anti-cancer therapy for advanced disease that includes a PD -1 and/or PD-L1 inhibitor or 2) who have sensitizing EGFR mutations and/or ALK gene rearrangements and have received prior tyrosine kinase inhibitor therapy, and who have tumor accessible by CT guided intervention or bronchoscopy.

Inclusion Criteria:

1. Male/female participants who are at least 18 years of age on the day of signing informed consent with histologically confirmed diagnosis of NSCLC will be enrolled in this study.
2. Stage IV pathologically proven NSCLC.
3. The participant (or legally acceptable representative if applicable) provides written informed consent for the trial.
4. Must have received prior systemic anti-cancer therapy for advanced disease that includes either:
   i. a PD-1 and/or PD-L1 inhibitor in patients without a sensitizing EGFR mutation and/or ALK gene rearrangement or,
   ii. Have received prior tyrosine kinase inhibitor therapy in patients with a sensitizing EGFR mutation and/or ALK gene rearrangement. For patients with sensitizing EGFR mutations, prior therapy must include osimertinib if a T790M mutation in the EGFR gene has been documented, and for patients with ALK gene rearrangements, prior therapy must include a second generation ALK inhibitor (e.g. alectinib, brigatinib, ceritinib). Note: For this group, prior cytotoxic chemotherapy is permitted, but prior therapy with a PD-1 or PD-L1 inhibitor is not permitted.
5. Measurable disease by RECIST v1.1 Guidelines (see Appendix B).
6. ECOG performance status of 0, 1(see Appendix A).
7. Have adequate organ function as defined in the following table (Table 1).

Table 1 Adequate Organ Function Laboratory Values

System	Laboratory Value
Hematological	
Absolute neutrophil count (ANC)	≥1500/µL
Platelets	≥100 000/µL
Hemoglobin	≥9.0 g/dL or ≥5.6 mmol/L[a]
Renal	
Creatinine OR<br>Measured or calculated[b] creatinine clearance<br>(GFR can also be used in place of creatinine or CrCl)	≤1.5 × ULN OR<br>≥30 mL/min for participant with creatinine levels >1.5 × institutional ULN
Hepatic	
Total bilirubin	≤1.5 ×ULN OR direct bilirubin ≤ULN for participants with total bilirubin levels >1.5 × ULN
AST (SGOT) and ALT (SGPT)	≤2.5 × ULN (≤5 × ULN for participants with liver metastases)
Coagulation	
International normalized ratio (INR) OR prothrombin time (PT)<br>Activated partial thromboplastin time (aPTT)	≤1.5 × ULN unless participant is receiving anticoagulant therapy as long as PT or aPTT is within therapeutic range of intended use of anticoagulants

ALT (SGPT)=alanine aminotransferase (serum glutamic pyruvic transaminase); AST (SGOT)=aspartate aminotransferase (serum glutamic oxaloacetic transaminase); GFR=glomerular filtration rate; ULN=upper limit of normal.

[a] Criteria must be met without erythropoietin dependency and without packed red blood cell (pRBC) transfusion within last 2 weeks.

[b] Creatinine clearance (CrCl) should be calculated per institutional standard.

Note: This table includes eligibility-defining laboratory value requirements for treatment; laboratory value requirements should be adapted according to local regulations and guidelines for the administration of specific chemotherapies.

8. A lesion that either:
    i. Is intended to be accessed bronchoscopically.
    OR
    ii. Is intended to be accessed with CT guided transthoracic injection and in the estimation of the radiologist performing the procedure will not require transversing a bullae that significantly increases the risk of pneumothorax.

9. Criteria for known Hepatitis B and C positive subjects

    Hepatitis B and C screening tests are not required unless:

    •Known history of HBV or HCV infection

•As mandated by local health authority

9.1 Hepatitis B positive subjects

Participants who are HBsAg positive are eligible if they have received HBV antiviral therapy for at least 4 weeks and have undetectable HBV viral load prior to randomization.

Participants should remain on anti-viral therapy throughout study intervention and follow local guidelines for HBV anti-viral therapy post completion of study intervention.

9.2 Participants with history of HCV infection are eligible if HCV viral load is undetectable at screening.

Participants must have completed curative anti-viral therapy at least 4 weeks prior to randomization.

10. Male participants:
    i. A male participant must agree to use a contraception as detailed in Appendix C of this protocol during the treatment period and for at least 4 months after the last dose of study treatment and refrain from donating sperm during this period.
11. Female participants:
    i. A female participant is eligible to participate if she is not pregnant (see Appendix C), not breastfeeding, and at least one of the following conditions applies:
        a. Not a woman of childbearing potential (WOCBP) as defined in Appendix 3.

        OR

        b. A WOCBP who agrees to follow the contraceptive guidance in Appendix C during the treatment period and for at least 4 months after the last dose of study treatment.

Exclusion Criteria:

1. Comorbid disease or a medical or psychiatric condition that would impair the ability of the patient to receive or comply with the study protocol.
2. Any use of systemic corticosteroids within 10 days of treatment initiation.
3. Evidence of respiratory failure or inability to tolerate biopsy procedure in the investigator's opinion.
4. Acute viral, bacterial, or fungal infection, which requires specific therapy. Acute therapy must have been completed at least 7 days prior to study treatment.
5. HIV infected patients (defined as HIV-1/HIV-2 Antibody positive).
6. Concurrent active Hepatitis B (defined as HBsAg positive and/or detectable HBV DNA) and Hepatitis C virus (defined as anti-HCV Ab positive and detectable HCV RNA) infection.

   Note: Hepatitis B and C screening tests are not required unless:
   --Known history of HBV and HCV infection
   --As mandated by local health authority
7. Has a known history of active tuberculosis.
8. Hypersensitivity to any reagents used in the study.

9. Pregnancy or inadequate contraception.
10. Lactating females.
11. Clinically active brain metastases, defined as untreated and symptomatic, or requiring therapy with steroids or anticonvulsants to control associated symptoms.. In patients with brain metastases that have been treated with radiation therapy, treatment must end at least 14 days prior to starting study treatment. History of leptomeningeal disease is exclusionary regardless of prior therapy.
12. Has had an allogenic tissue/solid organ transplant.
13. Previous or concurrent evidence of autoimmune disease requiring systemic steroids or immunosuppressive agents. Subjects with vitiligo or resolved childhood asthma/atopy would be an exception to this rule. Subjects that require inhaled steroid or local steroid injections will not be excluded from the study. Subjects with hypothyroidism who are managed by hormone replacement will not be excluded from the study.
14. Patients with a history or current evidence of any condition, therapy, or laboratory abnormality that might confound the results of the trial, interfere with the subject's participation for the full duration of the trial, or is not in the best interest of the subject to participate, in the opinion of the treating investigator.

Sample Size:

Up to 24 patients. During dose escalation, up to 12 patients will be evaluated, 3 or 6 patients at each dose level, depending on the presence or absence of a dose-limiting toxicity (DLT). To confirm safety of the dose established during dose escalation (ExD), if less than 6 patients were evaluated at this dose during dose escalation, the cohort will be expanded to a total of six patients. During dose expansion, 12 patients will be evaluated at ExD. From enrollment of the first patient onto the study until the last dose administered to the last subject, this study is anticipated to take approximately 4 years to complete.

Study Design:

A phase I, non-randomized, dose escalating, multi-cohort trial followed by a dose expansion portion at the dose established during dose escalation (ExD) will be conducted. During dose escalation, a modified 3+3 design will be used in the first dose escalation cohort and six patients will be enrolled in the second dose escalation cohort. Patients enrolled into a given cohort will receive the same Ad-CCL21-DC dose by CT-guided or bronchoscopic intratumoral injection preceded by intravenous pembrolizumab 200 mg on days 0, 21, and 42, and intravenous pembrolizumab 200 mg every three weeks thereafter for up to a year. The Ad-CCL21-DC dose is 1×10^7 cells/injection in the first cohort (1), and will be increased to 3×10^7 cells/injection (2) pending tolerability in the earlier cohort. Dose escalation may proceed only if all 3 patients enrolled in the lower dose cohort experience no DLT or 1 of 6 patients in a cohort has a DLT. If a patient dies within 30 days of receiving investigational treatment, the study will be held until further evaluation, and potential risks vs. benefits of continuing the study will be discussed with the UCLA JCCC DSMB. If the dose regimen in cohort 1 (Ad-CCL21-DC 1×10^7 cells/injection) is not well tolerated, de-escalation to Ad-CCL21-DC 5×10^6 cells/injection will be allowed (-1). If the dose regimen specified for Cohort (2) (Ad-CCL21-DC 3×10^7 cells/injection) is tolerable (0 or 1 DLT out of six patients), no further dose escalation will be conducted, and this dose level will be defined as maximum administered dose (MAD).

Following the completion of the dose-escalation phase, a dose expansion cohort of 12 patients will be enrolled and treated at ExD for up to a year (note: only intravenous pembrolizumab is given

after day 42). All enrolled patients will continue to be followed by a physician and undergo a history and physical examination every 3 months until progressive disease (PD) or withdrawal from the study. Eligible patients will be assigned to a cohort and will receive intratumoral injections of autologous Ad-CCL21-DC and intravenous pembrolizumab in conjunction with tumor sampling and patient monitoring on the days as detailed in **Table 2**.

Table 2

Table 2. Drug delivery and immune-monitoring schedule.

**Cohort (# pts)**	Anti-PD-1 Dose & Route	**Anti-PD-1 Injection Days**	Ad-CCL21-DC Dose & Route	**Ad-CCL21-DC Injection Days**	**Biopsy Days**	**Safety Monitoring Days**	**Immune Monitoring Days**	**Clinical Response Evaluation Days ****
-1 (6)	200 mg iv	0, 21, 42, then q3wk	5×10^6 IT	0, 21, 42	0, 21, 42	0, 63	0, 11, 21, 42, 63, 126, 189, 252, 315	S, 63, Q3mo
1 (3 or 6)	200 mg iv	0, 21, 42, then q3wk	1×10^7 IT	0, 21, 42	0, 21, 42	0, 63	0, 11, 21, 42, 63, 126, 189, 252, 315	S, 63, Q3mo
2 (6)	200 mg iv	0, 21, 42, then q3wk	3×10^7 IT	0, 21, 42	0, 21, 42	0, 63	0, 11, 21, 42, 63, 126, 189, 252, 315	S, 63, Q3mo
ExD (12)	200 mg iv	0, 21, 42, then q3wk	ExD IT	0, 21, 42	0, 21, 42	0, 63	0, 11, 21, 42, 63, 126, 189, 252, 315	S, 63, Q3mo

** CT imaging to assess tumor burden at patient screening, day 63, and every 3 months following DC injection until progression of disease or withdrawal from study.
S: at time of patient screening. Q3wk: every 3 weeks. Q3mo: every 3 months. ExD: Dose established during dose escalation
All event days are ±7 days

DLT is defined as grade 3 or greater toxicity as defined by the NCI Common Toxicity Criteria version 5.0. In the first dose escalation cohort, if 0 of 3 patients have a DLT, then we will escalate to the next dose by enrolling patients into the next cohort. Dose escalation may proceed only if all 3 patients have been enrolled into a lower dose cohort and no DLT is seen over a 28-day period. If 2 or 3 of the 3 have a DLT, the dose will be de-escalated to dose level -1. In the second dose escalation cohort, 6 patients will be enrolled and receive the same Ad-CCL21-DC dose. If 2 or more of the 6 patients in dose escalation cohort two have a DLT, then the dose is de-escalated to the previous dose. If we select a dose where only 3 patients have been treated, then an additional 3 patients will be treated at that same dose for a total of six patients at the ExD. In any case, if a patient dies within 30 days of receiving investigational treatment, the study will be held until further evaluation, and potential risks vs. benefits of continuing the study will be discussed with the UCLA JCCC DSMB. In determining the MTD/MAD, no more than 6 patients will be treated at a given dose. The MTD/MAD is defined as the dose level at which fewer than 2 of 6 patients experience a DLT. In the event that 2 or 3 of the 3 have a DLT in the first cohort (A), study accrual will be held. In the unlikely event that dose level 2 requires 6 patients, and de-escalation to a dose level 1 that had enrolled only 3 patients finds 2 DLTs in the additional 3 patients enrolled, we would discuss with the UCLA JCCC DSMB the appropriateness of enrolling at dose level -1. Serious adverse events (SAE) that are definitively not related to study drug (for example disease progression, biopsy complications, or other events not attributed to administration of or reaction to study drug) will not be counted as DLT. If a patient has a complication such as pneumothorax or bleeding after the pre-treatment biopsy, the Ad-CCL21-DC will still be injected unless the risk is considered inappropriate by the study investigator and/or proceduralist. If the risk is determined to be too high for the patient to receive the injection of Ad-CCL21-DC, the patient will remain on pembrolizumab alone. Patients who cannot complete the entire Ad-CCL21-DC dose schedule will be excluded from the MTD/MAD determination, and will be replaced with another participant. Patients withdrawing therapy due to prolonged treatment delays or for reasons other than DLT

will be replaced with other subjects until at least 3 evaluable patients have completed treatment in the first dose escalation cohort and at least 6 evaluable patients have completed treatment in the ExD during dose escalation.

Biologic and Clinical Responses: All patients will be monitored to assess the clinical efficacy of the treatment and to define potential determinants of the response. Clinical response will be evaluated by CT scans at screening, on day 63, and every 3 months thereafter until progression of disease or patient withdrawal from the study, as detailed in **Table 3** in the clinical protocol. Immune monitoring will be performed using tumor biopsies and peripheral blood samples. We will also utilize tumor biopsies to determine the mutational load and tumor associated neoantigens

Table 3: Schedule of Events

Schedule of Events (all events have a ±7 day window)									
Day	Screen	-6	0	11	21	42	Starting Day 63, and Q 3 weeks f/u until treatment discontinuation (up to 1 year)	Discontinua tion Visit	Follow up (Q3 months)
Informed Consent	X								
Disease Status									X
Physician Visit	X	X	X	X	X	X	X	X	
History and Physical	X	X	X	X	X	X	X	X	
ECOG	X		X	X	X	X	X	X	
Placement of catheter (if clinically indicated on a case by case basis)	X								
ECG (repeated if symptoms suggest)	X		X		X	X			
Pulse oximetry	X		X		X	X			
Leukapheresis[3]		X[2]		X[2]					
Blood for Safety Testing (post-injections)			X				X[1]		
Blood for CBC	X		X	X	X	X	X	X	
Blood for Chemistries/Creatinine	X		X	X	X	X	X	X	
Blood for LFTs	X		X	X	X	X	X	X	
Blood for PT, PTT	X		X	X	X	X	X	X	
Blood for thyroid testing	X		X	X	X	X	X	X	
Pregnancy test (female	X								

only)									
Urinalysis	X								
HIV, Hepatitis B and Hepatitis C sAg	X								
Blood for biologic/ immunologic tests			X	X	X	X	X[1]		
Tumor aspirate or biopsy			X		X	X			
Imaging to assess tumor burden & response	X						X[1]		
CT or MRI of brain (if symptoms suggestive of CNS metastasis)	X								
DC treatment			X		X	X			
Pembrolizumab treatment			X		X	X	X		

[1]Imaging done every 3 months to assess tumor burden and response. Safety Monitoring blood draws done on Day 63; and Immune Monitoring blood draws done on Day 63, 126, 189, 252, 315.
[2]The first leukapheresis will be performed prior to day -9. A second leukapheresis is to be done only if it is anticipated that not enough cells have been generated from the first leukapheresis procedure or if a patient receives IT Ad-CCL21-DC at lower than the cohort dose on Day 0. Of note, the second leukapheresis can occur on Day 11 or 32.
[3]100mL blood will be drawn at the time of leukapheresis for autologous serum preparation.

<u>**Tumor Biopsy Samples: days 0, 21, 42 (Flow chart 1)**</u>

<u>These are listed in order of priority.</u>

1. Multiplex immunofluorescence (MIF) will be utilized to characterize the immune phenotype of the tumor microenvironment (TME). **Table 4** of the clinical protocol lists surrogate markers of immune phenotypes (Parra, Uraoka et al. 2017). Priority will be given to panel 1, with the specific emphasis on CD8 and PD-L1 staining. These assays will be performed with biopsy samples collected on day 0, 21 and 42.

2. Whole Exome Sequencing (WES) will be performed using genomic DNA (gDNA) from pre- and post-treatment tumor samples to identify somatic mutational load and tumor-associated neoantigens. Patient's diagnostic tissue block (or paraffin-embedded day 0 biopsy if such block is not available) and paraffin-embedded day 42 biopsy will be utilized as pre- and post-treatment samples, respectively. Tumor cells will be enriched by laser capture microdissection, followed by gDNA isolation. Patient's gDNA from PBMC will be served as a germline reference in the mutational analyses. These studies will elucidate the evolution of neoantigen landscape following the combination therapy.

Flow Chart 1

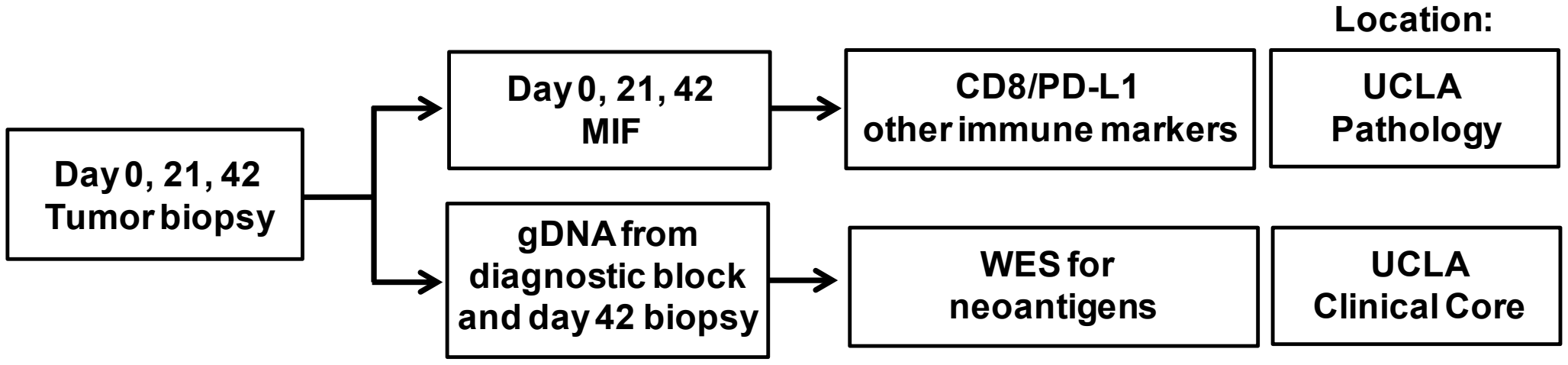

Table 4: Markers of immune phenotypes

Panel 1	Panel 2
AE1/AE3	AE1/AE3
PD-L1	PD-1
CD4	Granzyme B
CD8	CD57
CD3	CD45RO
CD68	FOXP3
DAPI	DAPI

Peripheral Blood Samples: days 0, 11, 21, 42, 63, 126, 189, 252, 315 (Flow chart 2)

1. CD8 T cells will be sorted from PMBC (collected on day 0, 21, 63, 126, 252) by flow cytometry and their DNA will be isolated using a DNeasy kit (Qiagen). TCR-β chain CDR3 regions will be amplified and deep sequencing will be performed with survey ImmunoSeq assay (Adaptive Biotechnologies) to determine clonality.
2. Mass cytometry (CyTOF) will be used to determine comprehensive immunophenotyping of immune cell subsets from PBMCs (collected on day 0, 11, 42, 189, 315) and elucidate the evolution of immune responses through the course of treatment.
3. As a complementary approach to MIF and CyTOF, Nanostring PanCancer Immune Profiling Panel will be utilized to monitor the anti-tumor immune responses in the study patients. This panel consists of 770 genes and includes 109 genes related to cell surface markers for 24 different immune cell types, as well as over 500 genes that represent all categories of immune response, including key checkpoint blockade genes. It also includes 40 reference genes for data normalization in the expression analysis. To identify leukocyte subpopulations based on gene expression, CYBERSORT method will be utilized(Newman, Liu et al. 2015). Briefly, RNA will be isolated from PBMCs (day 0, 11, 42, 189, 315), using an miRNeasy RNA Isolation Kit (Qiagen). RNA quality and integrity will be assessed by running on an Agilent Bioanalyzer. Two hundred nanograms of RNA will be used for the Nanostring analysis at the UCLA Center for Systems Biomedicine.
4. Quantification of anti-Ad antibody by ELISA, using plasma from day 0, 63 blood samples.
5. Antigen-specific ELISPOT assays to monitor immune responses to HLA-matched TAAs commonly seen in NSCLC, using PBMC from day 0, 21, 63, 126, 252.

Flow Chart 2

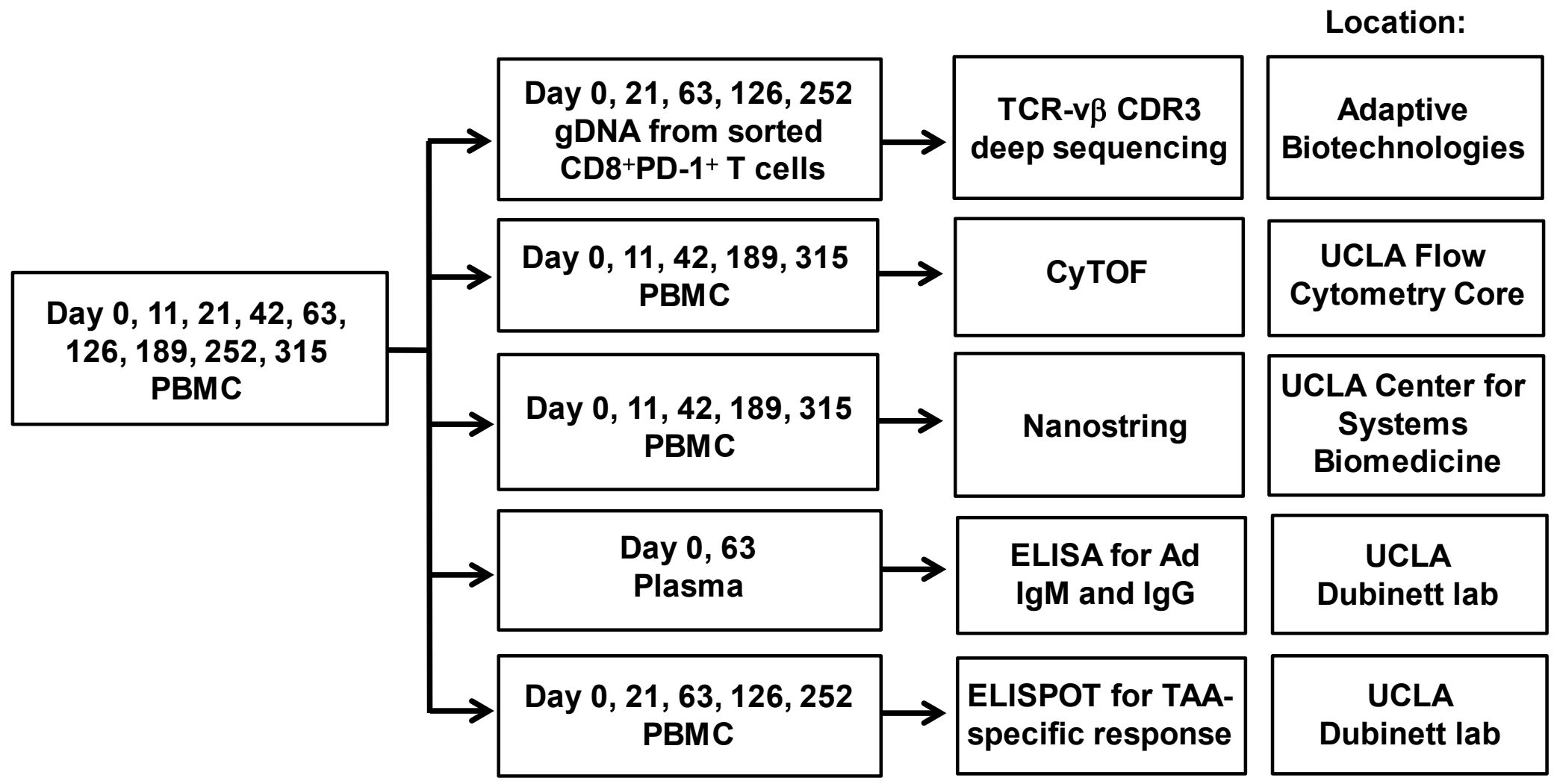

<u>Clinical Responses: Screening, day 63, Q3mo until progression of disease or withdrawal from study</u>

1. CT scans for evaluation of tumor burden (irRECIST Criteria for patient management, RECIST v1.1 for final analysis).
2. Toxicity by NCI Common Toxicity Criteria v 4.0 to define adverse event (AE) associated with the combination therapy of Ad-CCL21-DC and pembrolizumab.

<u>Biologic Reagents</u>:

1. Autologous monocyte-derived DC prepared under GMP conditions from the patient's peripheral blood and *in vitro* culture with 800 U/ml GM-CSF (both are manufactured and tested for clinical use under the Current Good Manufacturing Practices (cGMP) guidelines available from R&D SYSTEMS, Inc. (Minneapolis, MN)).
2. Anti-PD-1 immunotherapy pembrolizumab (Keytruda)

<u>Statistical Analysis</u>

- Sample Size Justification:
  A total sample size of up to 24 (up to 12 in Dose-Escalation Phase, 12 in Expansion Phase) patients is proposed. In the Dose-Escalation Phase, it is estimated to include approximately 9-12 patients to evaluate safety and tolerability. In the Expansion Phase, 12 additional patients will be evaluated at the dose established during dose escalation (ExD). Therefore, a total of 16 evaluable patients (6 in the dose escalation cohort +12 in the expansion cohort, with an assumed 10% dropout rate) will be used to further assess toxicity, ORR, immunologic and clinical responses. A sample size of 16 evaluable patients achieves 82.9% power to detect a 25% difference in ORR between the null hypothesis: ORR=8% and the alternative hypothesis: ORR=33%, using a two-sided one-sample exact Binomial test, at a 0.10 significance level. The rationale for combining these two patient populations for statistical analyses is that both have similar ORRs to single agent PD-1 inhibitor therapy, with the ORR for treatment with a PD-1 or PD-L1 inhibitor beyond progression is 8% and the ORR of pembrolizumab in patients with sensitizing EGFR mutations on the KEYNOTE-001 trial was 7.7% (Kazandjian et al. 2017; Hellmann 2015).

- Safety Analysis:

All safety analyses will be performed by dose level, treatment cycle and week, where appropriate. The AEs will be reported and categorized using a CTCAE v5.0 terminology and severity. Deaths and SAEs will be tabulated on a per-patient basis. Laboratory data will be presented by dose cohort and visit. Abnormal laboratory values will be assessed according to NCI CTCAE v5.0 where possible.

- Efficacy Analysis:
  The primary efficacy endpoint, objective response rate (ORR), will be summarized using descriptive statistics by dose level. The ORR will be analyzed using one-sample exact Binomial test for the patients treated at MTD or MAD in both the dose escalation phase and dose expansion phase. The 95% exact confidence interval (CI) will be provided.

- Analysis of Immunologic Data:
  Analysis of immunologic parameters are exploratory. All immunologic parameters will be summarized by time and dose level and presented graphically. The pre/post- treatment values of the tumor tissue immune parameters will be compared by paired t-test or Wilcoxon signed-rank test. For the repeatedly measured peripheral blood immune parameters, a spline regression model will be developed to characterize the change of immune parameter over time. Comparison between immune parameters will be carried out using the permutation method. Dose level will be included as a covariate.

Protocol Summary:

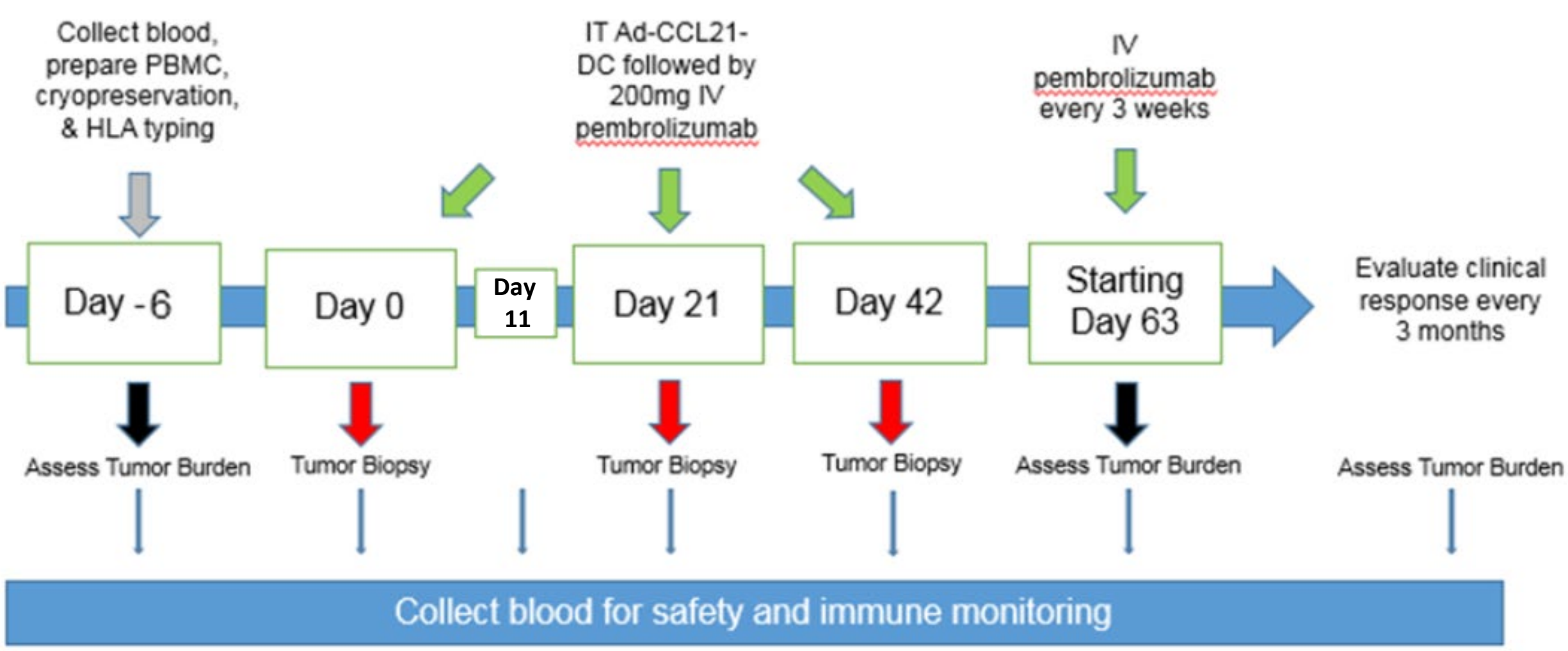

1 BACKGROUND AND RATIONALE

Lung cancer is the leading cause of cancer-related death in the United States and is responsible for more than 1 million deaths worldwide annually (Fong, Sekido et al. 1999; Bunn, Soriano et al. 2000). With the existing therapeutic efforts, patients with lung cancer have a poor prognosis. Approximately 17% of all patients live 5 years (and close to 1% in the setting of metastatic disease). This statistic has changed minimally in the last 20 years; therefore, there is a profound need for more effective therapeutic strategies.

Over 85% of patients with lung cancer have non-small cell lung cancer (NSCLC), including adenocarcinoma, squamous cell carcinoma and large cell carcinoma. The standard of care for advanced NSCLC includes surgery, radiation therapy, chemotherapy and targeted therapy (for activating mutations in EGFR or ALK fusion), while these therapies are often associated with undesirable toxicity and relapse of the disease. Recent studies reveal clinical responses in approximately 20% of Non-Small Cell Lung Cancer (NSCLC) patients treated with inhibitors of the programmed death-1 (PD-1)/PD-1 ligand (PD-L1) immune checkpoint. This includes robust and durable responses in previously treated patients with progressive locally advanced or metastatic NSCLC (Brahmer, Rizvi et al. ; Anagnostou and Brahmer 2015; Garon, Rizvi et al. 2015; Gettinger, Horn et al. 2015), as well as significant antitumor responses in treatment-naive advanced diseases. However, most patients do not respond to checkpoint inhibitors as single agents. Studies in melanoma and NSCLC patient-derived tumor specimens reveal that responses to PD-1/PD-L1 blockade are more likely in the setting of tumor PD-L1 expression and a pre-existing T lymphocyte response at baseline (Herbst, Soria et al. 2014; Tumeh, Harview et al. 2014). While lung cancers express tumor antigens (Yoshino, Goedegebuure et al. 1994; Dubinett, Batra et al. 2000), they do not function well as antigen presenting cells (APCs) (Restifo, Esquivel et al. 1993). In fact, the tumor's lack of co-stimulatory molecules, in combination with its production of inhibitory factors, promotes a state of suppressed cell-mediated immunity (Sharma, Stolina et al. 1999; Zhu, Sharma et al. 2003; Sharma, Yang et al. 2005; Baratelli, Lee et al. 2010; Srivastava, Andersson et al. 2012). Therefore, our efforts are focused on methods to restore tumor T lymphocyte infiltration, tumor antigen presentation, and T lymphocyte responsiveness to improve the effectiveness of checkpoint inhibitors in NSCLC patients.

One potential approach to enhance T lymphocyte responses is by *in situ* vaccination that takes advantage of the full repertoire of available tumor antigens (Dubinett, Lee et al. 2010). Our previous studies revealed that C-C Motif Chemokine Ligand 21 (CCL21)-modified dendritic cell (DC)-based intratumoral (IT) vaccination augments antigen presentation, resulting in effective T lymphocyte responses (Miller, Sharma et al. 2000; Sharma, Yang et al. 2003; Yang, Hillinger et al. 2004; Yang, Batra et al. 2006; Zeng, Aldridge et al. 2006; Baratelli, Takedatsu et al. 2008; Dubinett, Lee et al. 2010). The creation of chemokine gradients that favor lymphocyte entry into the tumor also facilitates *in situ* vaccination (Dubinett, Kurnick et al. 1989; Dubinett, Callahan et al. 1991; McBride, Thacker et al. 1992; Kim, Modlin et al. 1995; Hagenbaugh, Sharma et al. 1997; Huang, Stolina et al. 1998; Sharma, Stolina et al. 1999; Miller, Sharma et al. 2000; Sharma, Stolina et al. 2000; Sharma, Stolina et al. 2001; Hillinger, Yang et al. 2003; Sharma, Yang et al. 2003; Yang, Hillinger et al. 2004; Pan, Burdick et al. 2006; Yang, Batra et al. 2006; Baratelli, Takedatsu et al. 2008; Andersson, Yang et al. 2009; Dubinett, Lee et al. 2010). CCL21 has the ability to strongly attract T lymphocytes and DCs into T lymphocyte zones of secondary lymphoid organs to promote T lymphocyte activation (Chan, Kothakota et al. 1999; Cyster 1999). Our previous studies demonstrate that *in situ* vaccination with adenoviral CCL21-transduced DCs promotes systemic tumor immunity in murine lung cancer models (Yang, Hillinger et al. 2004; Yang, Batra et al. 2006). Our recent first-in-man phase I clinical trial with IT administration of autologous DCs expressing CCL21 (Ad-CCL21-DC) in patients with advanced

NSCLC further revealed effector T lymphocyte infiltration and systemic immune responses in these patients (Lee, Garon et al. 2014; Lee, Lee et al. 2017). However, our observation of increased PD-L1 expression in the tumor microenvironment (TME) of these patients suggests that tumor-mediated impairment of T lymphocyte function may be forestalling a more robust CCL21-mediated antitumor response. Similarly, the lack of efficacy for PD-1/PD-L1 inhibitors could potentially be overcome by enhanced T lymphocyte infiltration and augmented antigen presenting cell function. Therefore, we propose to combine Ad-CCL21-DC therapy with PD-1 inhibition to amplify host antitumor immunity in NSCLC patients who have previously received a PD-1 and/or PD-L1 inhibitor or have sensitizing EGFR mutations and/or ALK gene rearrangements and have received prior tyrosine kinase inhibitor therapy, who may also demonstrate minimal tumor T lymphocyte infiltration and most often do not respond to PD-1 inhibition alone.

We hypothesize that CCL21-dependent induction of $CD8^+$ effector T lymphocyte and DC infiltration of the tumor will lead to augmentation of systemic antitumor immune responses and thus increase the clinical benefit of anti-PD-1 therapy for patients who have previously progressed on anti-PD-(L)1 therapy or have sensitizing EGFR mutations and/or ALK gene rearrangements and have received prior tyrosine kinase inhibitor therapy**.** Our overall goal is to determine the potential of Ad-CCL21-DC to stimulate tumor-specific immune responses that enhance the antitumor activity of PD-1 blockade in NSCLC patients. Based on our extensive preclinical studies and recent phase I trial of Ad-CCL21-DC, along with its favorable toxicity profile, IT administration of Ad-CCL21-DC may prove to be an effective and innovative approach to improve the efficacy of PD-1 blockade in advanced NSCLC patients.

1.1 PD-1 inhibition and Pembrolizumab

1.1.1 Mechanism of the PD-1/PD-L1 Pathway

PD-1, encoded by the *PDCD1* gene, is an immunoglobulin in the CD28 family. PD-1 is a type I transmembrane glycoprotein containing an extracellular Ig variable-type (V-type) domain involved in ligand binding and a cytoplasmic tail involved in intracellular signaling. The cytoplasmic tail contains an immunoreceptor tyrosine-based inhibition motif (ITIM) and an immunoreceptor tyrosine-based switch motif (ITSM) (Okazaki, Maeda et al. 2001). Ligand binding to PD-1 induces recruitment of SHP-1 and SHP-2 to the ITSM motif, resulting in de-phosphorylation of CD3ζ, PKCθ, and ZAP70, which are involved in the CD3 T cell-signaling cascade (Sheppard, Fitz et al. 2004; Riley 2009). Binding of PD-1 with PD-L1 (or its other ligand, PD-L2) conveys a signal to the T lymphocyte that down-regulates its activation, preventing destruction of the antigen-presenting cell. Under healthy conditions, PD-L1 attenuates unwanted immune responses, such as autoimmunity (Keir, Butte et al. 2008).

1.1.2 Pembrolizumab Background

Pembrolizumab is a humanized anti-PD-1 antibody used in cancer immunotherapy. The detailed information on pembrolizumab is described in the Investigator's Brochure and approved labelling.

1.1.3 Efficacy of Pembrolizumab in Patients with Non-Small Cell Lung Cancer

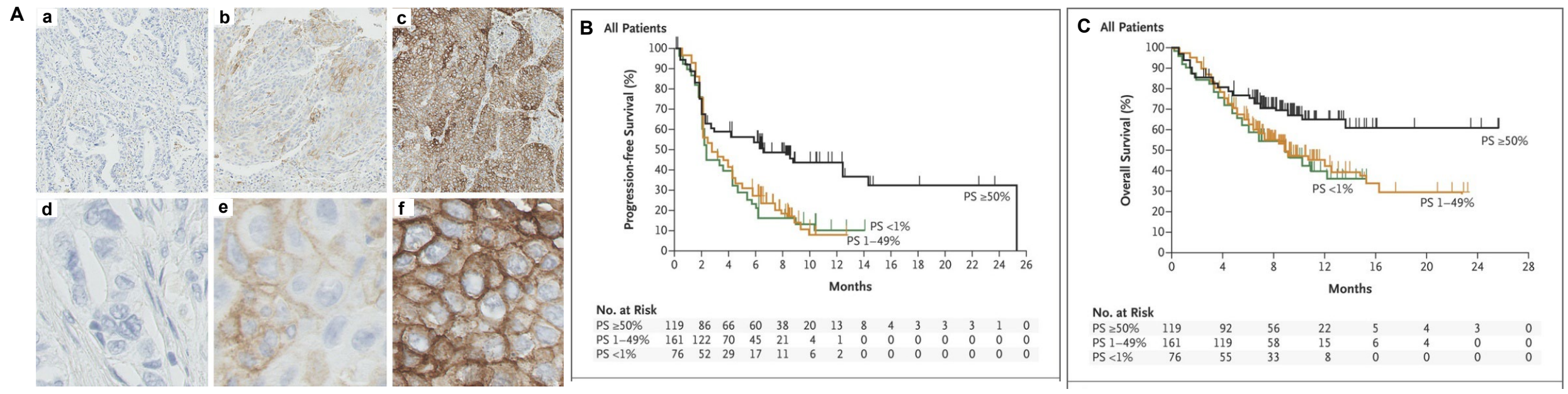

Fig 1. *KEYNOTE 001 trial revealed that NSCLC patients with >50% baseline tumor PD-L1 staining experienced greater benefit from anti-PD-1 pembrolizumab treatment than patients with <50% PD-L1 baseline expression.* ***A****) Baseline tumor PD-L1 expression. Shown are tumor samples from patients with a proportion score (PS) of less than 1% (a), 1 to 49% (b), or at least 50% (c). (d-f) higher magnification.* ***B****) Kaplan–Meier estimates of progression-free survival (PFS) according to the PS of 356 patients.* ***C****) Kaplan–Meier estimates of overall survival (OS) according to the PS of the same 356 patients as in* ***B****.*

Clinical trials testing the safety and efficacy of the anti-PD-1 antibody pembrolizumab in treating NSCLC patients have led to approval of the agent for this disease. The KEYNOTE 001 study revealed responses in approximately 20% of the patients with a modest side-effect profile (Garon, Rizvi et al. 2015). This includes robust and durable responses in previously treated patients with progressive locally advanced or metastatic NSCLC. In brief, 73, 103, and 28 patients were evaluated in the ≥50%, 1-49%, and <1% PD-L1 tumor staining groups, respectively. Patients with ≥50% PD-L1 staining in tumor cells at baseline experienced greater benefit from anti-PD-1 therapy than those with <50% tumor PD-L1 expression, with the Objective Response Rate (ORR) defined by Response Evaluation *Criteria* in Solid Tumors (RECIST) criteria of 45.2% in patients with ≥50% PD-L1 staining, versus 16.5% in patients with 1-49% PD-L1 staining and 10.7% in patients with <1% PD-L1 staining (**Fig.1**). The progression-free survival (PFS) outcomes of 6.4 months were seen in patients with ≥50% PD-L1 staining, compared to 4.1 months in the 1-49% PD-L1 staining group and 4.0 months in the <1% group. The overall survival (OS) was measured at 10.6 months and 10.4 months for patients with 1-49% and <1% PD-L1 tumor staining, respectively; while those having ≥50% staining did not reach the data cutoff point.

In the KEYNOTE 010 phase II/III study, 2 mg/kg and 10 mg/kg q3w of pembrolizumab showed significant benefit over 75 mg/m^2 q3w docetaxel in randomized, stage IV pre-treated patients with ≥1% PD-L1 staining, leading to approval in previously treated patients with any positive PD-L1 staining (Herbst, Baas et al. 2016). Among patients with ≥50% PD-L1 staining, the benefits of pembrolizumab over docetaxel was more pronounced, with median PFS of 5.0 months (2 mg/kg) and 5.2 months (10 mg/kg) vs 4.1 months with docetaxel, and the median OS of 14.9 months (2 mg/kg) and 17.3 months vs 8.2 months with docetaxel.

In the KEYNOTE 024 study, 200 mg of pembrolizumab showed significant benefit over investigators' choice of standard of care chemotherapy in treatment-naïve patients with ≥50% PD-L1 staining (Reck, Rodriguez-Abreu et al.). Median PFS was 10.3 months the pembrolizumab group vs 6.0 months in the chemotherapy group, and the estimated rate of OS at 6 months was 80.2% in the pembrolizumab group vs 72.4% in the chemotherapy group, despite the ability of patients on the chemotherapy arm to crossover to pembrolizumab. The population evaluated (those that exhibit ≥50% tumor PD-L1 staining at baseline) includes approximately 30% of total NSCLC lung cancer patients. Although pembrolizumab has not been extensively evaluated in treatment-naïve patients without ≥50% PD-L1 staining, the CheckMate 026 study of similar design evaluated a less restrictive population with respect to

PD-L1 staining (Carbone, Reck et al.). PFS was numerically greater with chemotherapy, while survival did not show a significant difference. Therefore, frontline PD-1 inhibition is restricted to patients with ≥50% PD-L1 staining.

In addition, anti-PD-(L)1 monotherapy has been shown to have minimal activity in patients with epidermal growth factor receptor (EGFR) mutations and anaplastic lymphoma kinase (ALK) rearrangements. In pretreated EGFR-mutant and ALK positive patients, ample evidence suggests that anti-PD-(L)1 therapy is inferior to docetaxel (Gainor, Shaw et al. 2016; Lee, Man et al. 2018). Specifically, the ORR for patients with sensitizing EGFR mutations with pembrolizumab on the KEYNOTE-001 trial was 7.7% (Hellmann MD 2015). Although, there is limited evidence regarding the efficacy of anti-PD-(L)1 prior to an EGFR tyrosine kinase inhibitor, we led a phase II study that evaluated pembrolizumab, prior to an EGFR TKI in 10 EGFR-mutant, tyrosine kinase inhibitor naïve patients, which was closed early due to futility as the objective response rate observed in the 10 EGFR-mutant patients on trial was 0% (Lisberg, Cummings et al. 2018).

As a result of the limited benefit of PD-1 inhibition in the majority of patients with advanced NSCLC, novel approaches that incorporate immunotherapy have been sought for the initial treatment of metastatic disease. One such approach is the combination of platinum-based chemotherapy plus pembrolizumab which has been shown to be superior to platinum-based chemotherapy alone in patients that are EGFR/ALK wild-type as front-line treatment in NSCLC patients with both non-squamous and squamous disease (Gandhi, Rodríguez-Abreu et al. 2018; Paz-Ares, Luft et al. 2018). Similarly, atezolizumab, a PD-L1 inhibitor, is approved in combination with platinum based chemotherapy and an angiogenesis inhibitor, bevacizumab, as first-line treatment of advanced NSCLC (Socinski, Jotte et al. 2018). Although results from an EGFR-mutant subgroup analysis on the atezolizumab trial are encouraging, the FDA approval for atezolizumab plus platinum based chemotherapy plus bevacizumab, as well as pembrolizumab plus platinum-doublet chemotherapy, is limited to EGFR/ALK wild-type patients.

The approval of chemo-immunotherapy combinations has altered the treatment landscape in advanced NSCLC, such that all eligible patients receive immunotherapy in the front-line setting, either in combination with chemotherapy, an approach approved irrespective of PD-L1 level, or monotherapy in select patients with PD-L1>50% (Lisberg and Garon 2019). This shift has led to improved patient outcomes in the front-line setting, but created a significant area of unmet need, namely effective treatment options for patients after progression on a PD -1 and/or PD-L1 inhibitor.

After progression on a PD -1 and/or PD-L1 inhibitor administered in combination with platinum based chemotherapy, a treatment regimen that is now standard of care for more than 70% of patients with advanced NSCLC (all with PD-L1 < 50% and many with PD-L1 > 50%), effective options are very limited. Second-line chemotherapy is an option for these patients but is associated with significant toxicity and limited efficacy (Shepherd 2003). As a result, treatment with a PD -1 or PD-L1 inhibitor beyond progression has been evaluated, but has not led to meaningful clinical benefit (Kazandjian, Keegan et al. 2017).Taken together, despite robust and durable responses to anti-PD-1 therapy in a subgroup of patients with locally advanced and metastatic NSCLC, most patients do not experience durable responses with checkpoint inhibitors. As the standard of care for initial therapy in patients is now chemotherapy plus a PD -1 and/or PD-L1 inhibitor or PD-1 inhibitor monotherapy, there is an urgent need for effective treatment options for patients after progression on a PD-1 and/or PD-L1 inhibitor. In addition, patients with EGFR mutations and ALK rearrangements are also in need of an effective immunotherapy approach. Therefore, rational and effective combination strategies with PD-1 inhibitors are needed to enhance the efficacy of anti-

PD1 therapy in advanced NSCLC patients .

1.2 CCL21-modified DC as a therapeutic agent in lung cancer immunotherapy

DCs are bone marrow-derived leukocytes characterized by the high expression of human leukocyte antigen (HLA) and co-stimulatory molecules. DCs have the capacity to take up, process and present antigens to facilitate activation and expansion of antigen-specific T lymphocytes (Banchereau and Steinman 1998; Steinman 2012). Recent studies demonstrate that tumor-residing Batf3-driven CD103^{+} (mouse)/CD141^{+} (human) DCs are required for tumor antigen trafficking, effector T lymphocyte infiltration and T lymphocyte antitumor immunity (Roberts, Broz et al. 2016; Spranger, Dai et al. 2017). In addition, CXCL10 produced by CD103^{+} DCs is required for effector T lymphocyte migration (Spranger, Dai et al. 2017). Because of the importance of DC in tumor immunity, a variety of strategies has been utilized to exploit activated DC in cancer immunotherapy (Cerundolo, Hermans et al. 2004; Sabado, Balan et al. 2016; Constantino, Gomes et al. 2017; Garg, Vara Perez et al. 2017).

We have demonstrated that Ad-CCL21-DC-based IT vaccination augments antigen presentation, resulting in effective T lymphocyte responses (Sharma, Batra et al. 2003; Yang, Hillinger et al. 2004; Yang, Batra et al. 2006; Zeng, Aldridge et al. 2006; Baratelli, Takedatsu et al. 2008; Dubinett, Lee et al. 2010; Lee, Garon et al. 2014; Lee, Lee et al. 2017). Chemokines are a group of homologous, yet functionally divergent proteins that directly mediate leukocyte migration and activation, as well as angiogenesis (Nagarsheth, Wicha et al. 2017). CCL21 is a C-C chemokine expressed in high endothelial venules and in T lymphocyte zones of spleen and lymph nodes. CCL21 strongly attracts T lymphocytes and DC to T lymphocyte zones by interacting with chemokine receptors CXCR3 and CCR7 (Chan, Kothakota et al. 1999; Cyster 1999), co-localizing these early immune response constituents and culminating in cognate T lymphocyte activation (Cyster 1999). In addition, CCL21 has been shown to have potent angiostatic effects (Vicari, Ait-Yahia et al. 2000), thus adding further support for its use in cancer therapy.

1.2.1 Intratumoral CCL21 administration induces antitumor responses in vivo in murine lung cancer models

The development of IT therapies to effectively augment local and systemic antitumor immunity in lung cancer could lead to a paradigm shift in the current forms of therapy. Congruent with this overall concept, the IT administration of the recombinant CCL21 mediated T lymphocyte-dependent antitumor responses in 2 independent syngeneic murine lung cancer models, L1C2 and 3LL (**Fig. 2A**) (Sharma, Stolina et al. 2000). Consistently, IT injection of CCL21 significantly increased the infiltration of CD4^{+} and CD8^{+} T lymphocytes and DC into both the tumor and draining lymph nodes. Accompanying these cell infiltrates were increases in IFNγ, CXCL9, CXCL10, GM-CSF and IL-12, while a concomitant decrease in the immunosuppressive molecules PGE-2 and TGFβ. The efficacy of CCL21-mediated antitumor responses required induction of IFNγ, CXCL9 and CXCL10, as *in vivo* depletion of CXCL9, CXCL10 or IFNγ significantly reduced the antitumor efficacy of CCL21 in a syngeneic murine lung cancer model 3LL (Sharma, Yang et al. 2003). In addition, lymphocytes from CCL21-treated tumor-bearing mice demonstrated enhanced specific responses against autologous tumors, indicating the generation of systemic immune responses (Sharma, Stolina et al. 2000). Similarly, the antitumor activity of CCL21 was demonstrated in a spontaneous murine bronchoalveolar cell carcinoma model in transgenic mice, in which the SV40 large T Ag was expressed under the control of the murine Clara cell-specific promoter CC-10 (**Fig. 2B**) (Sharma, Stolina et al. 2001).

Following our initial description of the antitumor activities of CCL21, several groups have reported

that CCL21 has potent antitumor properties in a variety of model systems, including B16 melanoma, murine hepatocellular carcinoma, and a murine prostate cancer model (Kirk, Hartigan-O'Connor et al. 2001; Liang, Zhong et al. 2007; Novak, Igoucheva et al. 2007; Wu, Xing et al. 2008; Yousefieh, Hahto et al. 2009). In all models, CCL21 demonstrated potent regression of tumors, which was dependent on host T lymphocyte immunity. These studies reaffirmed the antitumor efficacy of CCL21, further supporting the rationale to proceed with clinical investigations of this chemokine.

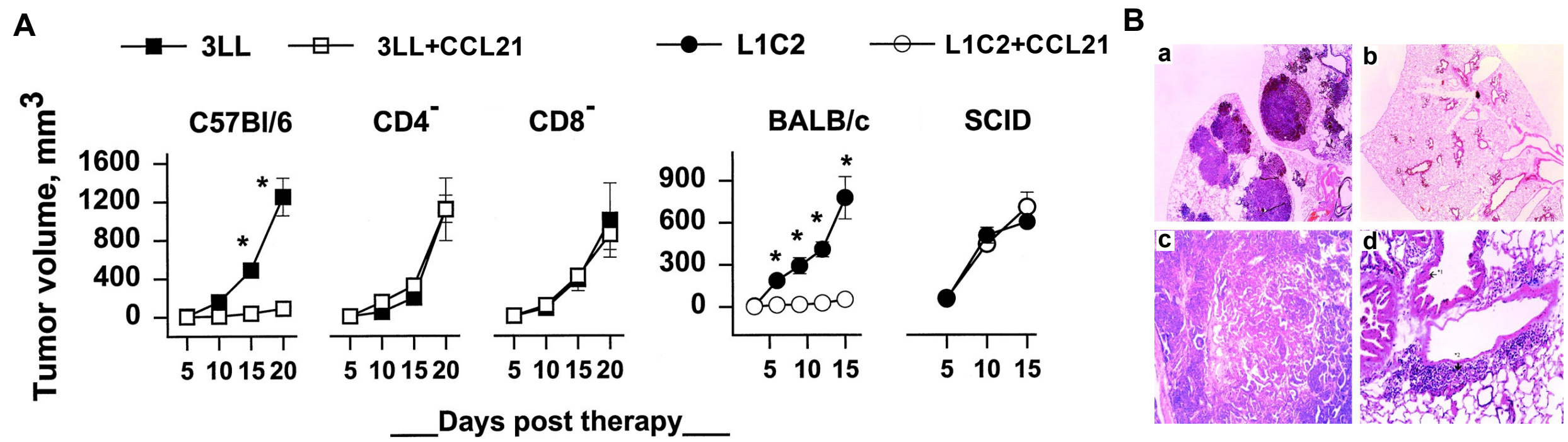

Fig 2. *<u>CCL21 mediates antitumor responses in immune competent mice, which requires CD4+ and CD8+ T lymphocytes.</u>* ***A****) 3LL (H-2^{d}) or L1C2 (H-2^{b}) cells (10^{5}) were inoculated s.c. into the right suprascapular area in C57BL/6 and BALB/c mice, respectively. Five days after tumor establishment, murine recombinant CCL21 (0.5 □g/injection) or 1x PBS diluent was IT administered 3 times per week for 2 weeks (n = 10–12 mice/group). *, P < 0.01. Similar studies were performed in CD4- and CD8-knockout mice (3LL); or SCID mice (L1C2).* ***B****) CCL21 mediates potent antitumor responses in a murine spontaneous bronchogenic carcinoma model in transgenic mice, in which the SV40 large T Ag is expressed under control of the murine Clara cell-specific promoter CC-10. Mice expressing the transgene develop diffuse bilateral bronchoalveolar carcinoma and have an average life span of 4 months. CCL21 (0.5 µg/injection) or murine serum albumin (control) was injected in the axillary lymph node region of 4-week-old transgenic mice 3 times a week for 8 weeks. At 4 months, mice in all groups were euthanized, and their lungs were embedded in paraffin and subjected to H&E staining. (a and c) Control mice; (b and d) CCL21-treated mice. (a and b, ×32; c and d, ×320). Median survival was 18 ± 2 weeks for control mice, while 34 ± 3 weeks for CCL21-treated mice (n = 10 mice/group).*

1.2.2 IT administration of Ad-CCL21-DC results in tumor regression in murine lung cancer models *in vivo.*

To enhance the efficacy and duration of CCL21-mediated antitumor immunity, we performed additional preclinical studies exploiting IT injections of adenoviral-encoded CCL21-modified DCs (Ad-CCL21-DC) into established tumors in a syngeneic murine L1C2 lung cancer model (Yang, Hillinger et al. 2004). Ad-CCL21-DC administration resulted in tumor regression and enhanced protective immunity compared to mice treated with the control vector-transduced DC (CV-DC) or unmodified DC. The complete tumor eradication occurred in 63% of Ad-CCL21-DC treated mice, while only 12% of the mice treated with either the unmodified DC or CV-DC responded. In addition, IT administration of Ad-CCL21-DC induced systemic antitumor immune responses. When mice were inoculated with tumor cells in both flanks, and only the right flank tumor was treated with IT injection, 33% of mice treated with Ad-CCL21-DC had complete tumor eradication of both flank tumors (**Fig. 3A**). Consistently, Ad-CCL21-DC administration increased tumor infiltration of the CD4^{+}, CD8^{+} and CD3^{+}CXCR3^{+} T lymphocytes, as well as DC expressing CD14c^{+} DEC205^{+}, while markedly reduced tumor infiltration of the CD4^{+}CD25^{+} regulatory T lymphocytes (**Fig. 3B**). Ad-CCL21-DC therapy induced specific T-cell response, as evident by the increased frequency of tumor-specific IFNγ-producing cells (**Fig. 3C**). Furthermore, *in vivo* depletion of CXCL10, CXCL9, or IFNγ significantly reduced the antitumor efficacy of Ad-CCL21-DC (**Fig. 3D**).

Similarly, Ad-CCL21-DC elicited potent antitumor immunity in a murine model of spontaneous bronchoalveolar cell carcinoma in transgenic mice expressing the SV40 large T antigen under the Clara cell promoter (CC-10 TAg) (Yang, Batra et al. 2006). A single intra-tracheal instillation of Ad-CCL21-DC resulted in a marked reduction in tumor burden with extensive lymphocytic infiltration into the tumors, which is accompanied by the enhanced elaboration of the type 1 cytokines IFNγ, IL-12, GM-CSF, the anti-angiogenic chemokines CXCL9, CXCL10, while a concomitant decrease in the immunosuppressive molecules IL-10, TGFβ and PGE-2; as well as a significantly greater frequency of tumor specific T lymphocytes releasing IFNγ. Taken together, these data demonstrate that *in situ* administration of Ad-CCL21-DCs can elicit a potent antitumor immunity in murine lung cancer models, and warrant further investigation of using Ad-CCL21-DC in cancer therapy.

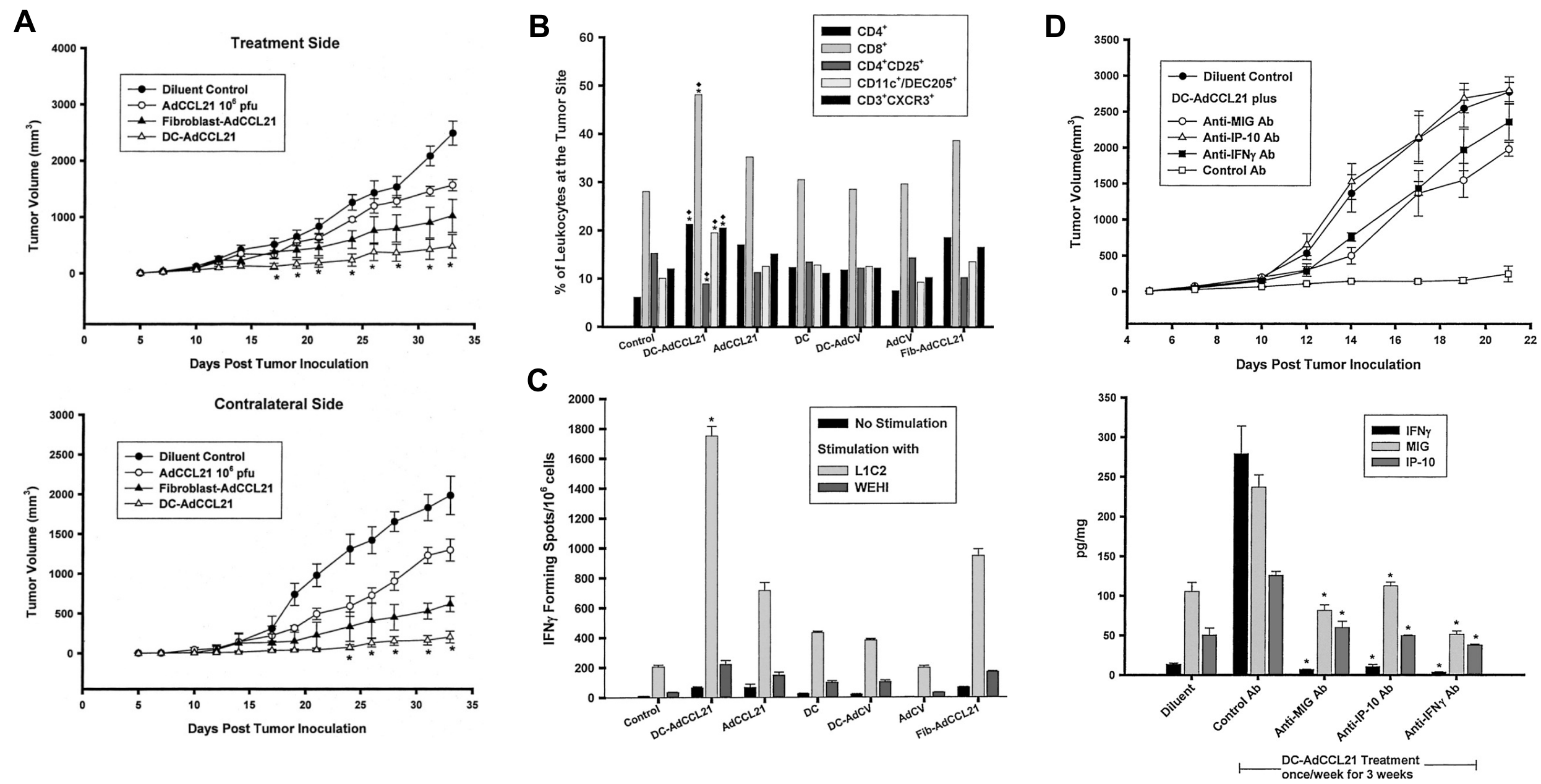

***Fig 3**. <u>IT injection of Ad-CCL21-DC confers tumor immunity in a syngeneic murine L1C2 lung cancer model.</u>* ***A**) IT administration of Ad-CCL21-DC leads to the suppression of bilateral tumors. L1C2 tumor cells (1.5 × 10^5) were inoculated on the right suprascapular area and 2 × 10^4 L1C2 tumor cells on the left in BALB/c mice. Five days after tumors were established, mice were IT injected on the right side only with diluent, Ad-CCL21-DC (10^6), AdCCL21 (10^6 PFUs), or fibroblast-AdCCL21 (10^6) once a week for 3 weeks. Top panel: tumor growth on the treatment side; Bottom panel: tumor growth on the contralateral side. * P < 0.01, compared with diluent treated control and * P < 0.05 compared with fibroblast-AdCCl21 treatment group; n = 8 mice/group. **B**) Ad-CCL21-DC enhances influx of T lymphocyte subsets and DC at the tumor site. Five-day-old established tumors were treated once a week for 2 weeks as in **A**, before tumors were harvested for flow cytometric analysis. *, P < 0.001 compared with diluent control; ♦ P < 0.05 compared with other treatment groups. **C**) Ad-CCL21-DC therapy induces specific T lymphocyte response. Five-day-old established tumors were treated once a week for 3 weeks as in **A**, and splenocytes were harvested and restimulated overnight with irradiated L1C2 cells at a ratio of 10:1. Mouse IFNγ-specific enzyme-linked immunospot (ELISPOT) was performed and spots quantified with an Immunospot Image Analyzer. There were minimal responses to the syngeneic control tumor WEHI. *, P < 0.001; n = 8 mice/group. **D**) Ad-CCL21-DC-mediated antitumor responses require IFN γ, CXCL9, and CXCL10. **Top**, L1C2 (1.5 × 10^5) were implanted in BALB/c mice. Five days after tumor implantation, mice were IT injected with Ad-CCL21-DC once a week for 3 weeks. One day before Ad-CCL21-DC administration, mice were given the respective cytokine antibody by i.p. injection. The antibodies were administered 3 times per week. P < 0.01 for anti-IFNγ and anti-CXCL9; P < 0.001 for anti-CXCL10 compared with the control group; n = 8 mice/group. **Bottom**, Ad-CCL21-DC-treated mice had a significant induction in IFN γ, CXCL9, and CXCL10 at the tumor*

*site compared with diluent-treated tumor-bearing mice. *, P < 0.01; n = 8 mice/group.*

1.2.3 <u>IT administration of Ad-CCL21-DC induces specific systemic antitumor responses in advanced NSCLC patients.</u>

We have a long-term interest in understanding the nature of the immune-suppressive TME (Restifo, Esquivel et al. 1993; Yoshino, Goedegebuure et al. 1994; Sharma, Stolina et al. 1999; Zhu, Sharma et al. 2003; Sharma, Yang et al. 2005; Baratelli, Lee et al. 2010; Dubinett, Lee et al. 2010; Srivastava, Andersson et al. 2012; Tumeh, Harview et al. 2014) and in developing immune-based strategies to augment systemic antitumor immunity in lung cancer (Dubinett, Kurnick et al. 1989; Dubinett, Callahan et al. 1991; McBride, Thacker et al. 1992; Dubinett, Patrone et al. 1993; Dubinett, Huang et al. 1995; Kim, Modlin et al. 1995; Hagenbaugh, Sharma et al. 1997; Huang, Stolina et al. 1998; Chan, Kothakota et al. 1999; Cyster 1999; Sharma, Stolina et al. 2000; Sharma, Stolina et al. 2001; Hillinger, Yang et al. 2003; Lee, Garon et al. 2014; Carmi, Spitzer et al. 2015; Politi and Herbst 2015; Rizvi, Hellmann et al. 2015). Based on our extensive investigation in murine models demonstrating the benefit of intratumoral Ad-CCL21-DC vaccination, we developed and completed an NCI-supported first-in-man phase I, non-randomized, dose escalating, multi-cohort clinical trial assessing IT injection of autologous Ad-CCL21-DC in patients with advanced NSCLC (Lee, Lee et al. 2017). Following peer-review and approval, the RAID program provided a GMP-grade replication-deficient adenovirus expressing CCL21 for our therapy-related work. Sixteen patients received 2 vaccinations at various doses of Ad-CCL21-DC (cohorts A, B, C, or D received 1, 5, 10, or 30 million cells/injection, respectively) by IT injection (days 0 and 7). Peripheral blood was collected for antigen-specific enzyme-linked immunospot (ELISPOT) assays, and CT guided needle biopsies of the primary lung cancer were obtained at the baseline and following the second IT injection for evaluation of cellular infiltrates by immunohistochemistry (IHC). PD-L1 RNA and protein levels were also evaluated by quantitative real time PCR (Q-PCR) and IHC analyses, respectively. Immune response was defined as tumor associated antigen (TAA)-specific and vaccine-dependent IFNγ production that showed more than a 2-fold increase in spot numbers compared to the background (no peptide) with the absolute number of more than 20 spots per 2.5 x 10^5 PBMC. Subjects that had a high response with TAA-non-specific and vaccine-independent IFNγ production at the baseline, which declined after vaccination but met the criteria for TAA-specific and vaccine-dependent immune responses, were included as immune responders in the analysis. The IFNγ ELISPOT assay revealed 6 out of 16 total immune responders. Of these, 3 responders had a high response with TAA non-specific and vaccine-independent IFNγ production at the baseline that declined after vaccination yet met criteria for TAA-specific and vaccine-dependent immune responses on subsequent monitoring.

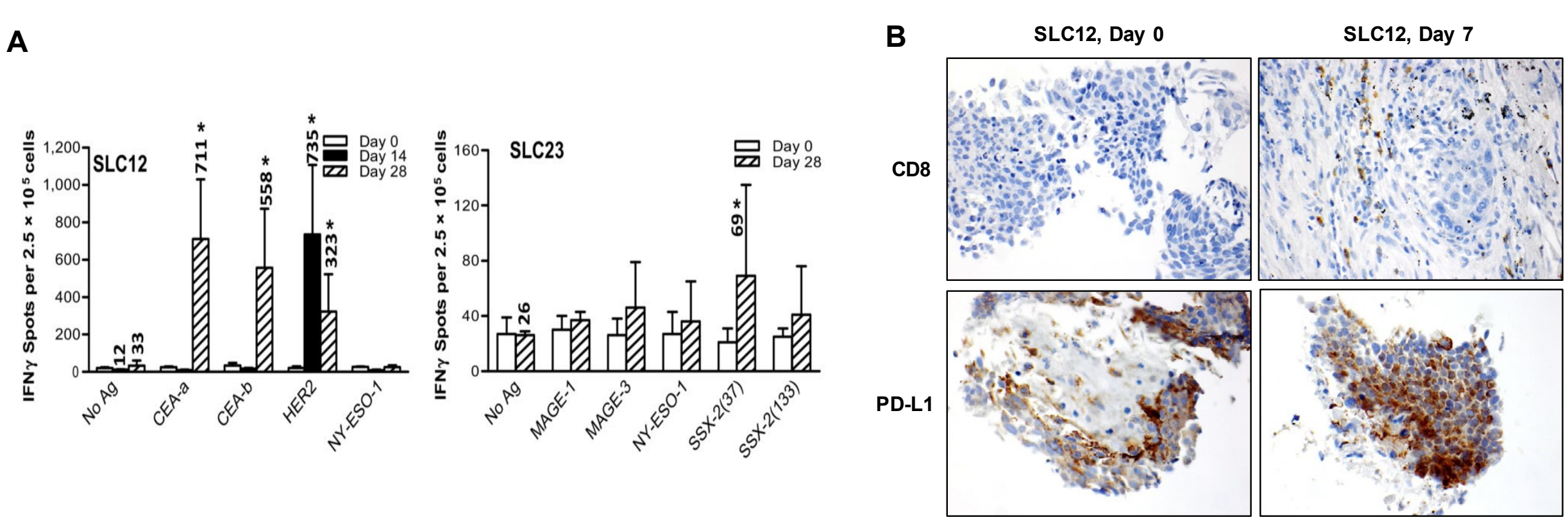

Fig 4. *<u>IT administration of Ad-CCL21-DC in a phase I trial induced positive immune responses (PIR) to</u>*

autologous tumor-associated antigens (TAA), CD8+ T lymphocyte tumor infiltration and increased tumor PD-L1 expression. ***A****) PBLs were collected pre- and post-treatment and were co-cultured in an antigen presentation assay including the patient's HLA-matched peptides for 24 hrs to determine specific immune responses by IFNχ ELISPOT assay. Six out of 16 patients had a treatment-dependent PIR; IFNχ ELISPOT of two representative patients (SLC12 and SLC23) with PIRs are shown.* ***B****) Formalin-fixed paraffin-embedded (FFPE) IHC photomicrographs from patient SLC12 indicate that CD8+ T lymphocytes are recruited into the tumor on day 7 relative to day 0 (top row); however, tumor PD-L1 expression is also upregulated after treatment (bottom row).*

While the tumors of these 16 advanced NSCLC patients did not regress within the time frame of our trial, 6 out of 16 patients had an immune response, as determined by IFNγ release from patients' peripheral blood lymphocytes (PBLs) in response to defined peptide epitopes of TAA (**Fig. 4A**). In addition, tumor $CD8^+$ T lymphocyte infiltration was induced in 7 out of 13 subjects; however, the benefit from these tumor-infiltrating lymphocytes (TILs) may have been limited by the upregulation of tumor PD-L1 expression, which increased significantly accompanying the increased $CD8^+$ T lymphocyte infiltration (**Fig. 4B**). No treatment-related serious adverse events (AEs) were observed (Lee, Lee et al. 2017). Thus, we concluded that IT administration of Ad-CCL21-DC: 1) is safe; 2) induces specific systemic antitumor immune responses in a significant subset of patients; 3) induces $CD8^+$ T lymphocyte tumor infiltration; and 4) is accompanied by induction of tumor PD-L1 expression in a subset of patients (Lee, Lee et al. 2017).

1.3 Rationale for the combination of Ad-CCL21-DC and pembrolizumab

Cancer immunotherapy employing PD-1/PD-L1 immune checkpoint blockade can induce robust and durable responses in patients with metastatic cancers, including melanoma and NSCLC; however, the clinical benefit is limited to a subgroup of patients, while the majority of the patients do not respond to PD-1 inhibitors as single agents due to primary, adaptive or acquired resistance to cancer immunotherapy (Sharma, Hu-Lieskovan et al.). A better understanding of the molecular mechanisms underlining the resistance to cancer immunotherapy and the rational design of the combinational therapy to overcome the resistance will facilitate a better selection of patients and improve the efficacy of the current anti-PD-1 therapy.

1.3.1 The efficacy of PD-1/PD-L1 checkpoint inhibition may be correlated with tumor PD-L1 expression, $CD8^+$ T lymphocyte tumor infiltration, tumor mutational load, and TCR clonality.

Previous studies of PD-1 blockade in melanoma patients revealed that the response rates were correlated with the tumor PD-1/PD-L1 expression, the pre-existing $CD8^+$ TILs and TCR clonality (Tumeh, Harview et al. 2014). The KEYNOTE 001 clinical trial with anti-PD-1 in NSCLC patients also revealed a higher ORR of approximately 45% among patients with tumor PD-L1 expression of at least 50%, as compared to the overall responses of approximately 20% in all patients evaluated (Garon, Rizvi et al. 2015). In addition, it was shown that higher nonsynonymous mutation burden in tumors was associated with improved objective response, durable clinical benefit, and progression-free survival in NSCLC patients (Rizvi, Hellmann et al. 2015). A more recent study found a link between clonal mutations and response to treatment in primary lung adenocarcinomas (McGranahan, Furness et al.). Interestingly, NSCLC patients with high clonal neoantigens showed favorable response to PD-1 checkpoint inhibition. These studies suggest that the efficacy of PD-1/PD-L1 checkpoint inhibition may be determined by multiple factors, including tumor PD-L1 expression, $CD8^+$ T lymphocyte tumor infiltration, tumor mutational load and TCR clonality (Zou, Wolchok et al. 2016). The current hypothesis is that TAAs and neoantigens resulting from nonsynonymous somatic mutations are recognized by

activated CD8+ T lymphocytes infiltrating the tumor, which may in turn upregulate the expression of immune checkpoints, such as PD-L1, via IFNγ-dependent induction by tumor cells and host tumor-infiltrating immune cells, resulting in an adaptive immune resistance. In the presence of high tumor PD-1/PD-L1 expression and a pre-existing CD8+ TILs, immune checkpoint blockade can then unleash the patient's own immune system to elicit an antitumor response.

1.3.2 IT Ad-CCL21-DC treatment may work in synergy with PD-1 inhibition to improve the anti-PD-1 therapy in NSCLC patients.

Strategies to enhance T lymphocyte infiltration into the tumor and to restore tumor antigen presentation are likely to improve the efficacy of anti-PD-1 inhibitors in NSCLC patients. One potential approach to enhance T lymphocyte responses is by *in situ* vaccination that takes advantage of the full repertoire of available tumor antigens (Dubinett, Lee et al. 2010). Our phase I clinical trial with IT administration of Ad-CCL21-DC in advanced NSCLC patients revealed the induction of specific systemic antitumor immune responses and CD8+ T lymphocyte infiltration in a significant subset of patients (Lee, Lee et al. 2017). However, we observed increased PD-L1 expression in the TME of IT Ad-CCL21-DC-treated patients, suggesting that tumor-mediated impairment of T lymphocyte function may be forestalling a more robust CCL21-mediated antitumor response. Similarly, the lack of efficacy for PD-1/PD-L1 inhibitors could potentially be combated by enhanced T lymphocyte infiltration and augmented antigen presenting cell function. Therefore, we propose to combine IT Ad-CCL21-DC therapy with PD-1 inhibition to amplify host antitumor immunity in NSCLC patients. Therefore, we propose to select patients with NSCLC who have received prior systemic anti-cancer therapy for advanced disease that includes a PD -1 and/or PD -L1 inhibitor or patients that have sensitizing EGFR mutations and/or ALK gene rearrangements and have received prior tyrosine kinase inhibitor therapy for the current proposal of the combination therapy of Ad-CCL21-DC and pembrolizumab.

We also plan to perform systematic immune monitoring studies using tumor biopsies and peripheral blood samples collected from these patients. As stated above, we expect that IT administration of Ad-CCL21-DC should result in enhanced T lymphocyte infiltration and a concurrent increase in tumor PD-L1 expression, which often are indications that patient is likely to respond to anti-PD-1 therapy. Therefore, we will assess tumor PD-L1 expression and CD8+ T lymphocyte infiltration by Multiplex immunofluorescence (MIF) using tumor biopsies. It has been implicated that higher tumor nonsynonymous mutation burden and TCR clonality are associated with improved objective response and durable clinical benefit in cancer patients [10, 67, 71]. To evaluate whether tumor somatic mutational load/neoantigens and TCR clonality correlate with the clinical outcome in the proposed study, we will perform Whole Exome Sequencing (WES) analyses to identify tumor mutational load and tumor-associated neoantigens using tumor biopsies. In addition, TCR clonality will be assessed by deep sequencing of the TCR-β chain CDR3 region. Furthermore, patients' immunophenotyping and antitumor immune responses will be examined by Mass cytometry (CyTOF), Nanostring PanCancer Immune Profiling, and TAA-specific ELISPOT using PBMC from patients' peripheral blood. These approaches are complementary to one another. CyTOF will be used to determine the comprehensive immunophenotyping of immune cell subsets using cell surface immune markers, and elucidate the evolution of immune responses through the course of treatment. Nanostring is to assess the immune response by profiling a panel of 770 immune-related genes at the RNA level, including 109 cell surface markers for 24 different immune cell types, as well as over 500 genes that represent all categories of immune response, including key checkpoint blockade genes. Lastly, TAA-specific ELISPOT is a direct functional assay to examine the systemic anti-tumor immune

response. These comprehensive studies could potentially reveal the molecular factors that dictate the responsiveness to the combination therapy of Ad-CCL21-DC and pembrolizumab, and facilitate the improvement of the therapies that could benefit the majority of the NSCLC patients.

In summary, our goal is to determine the potential of IT administration of Ad-CCL21-DC to stimulate tumor-specific immune responses that enhance the antitumor activity of PD-1 blockade in NSCLC patients. Therefore, we propose a phase I clinical trial to determine if the addition of IT injection of Ad-CCL21-DC to the anti-PD-1 treatment regimen: 1) is safe; 2) improves ORRs to anti-PD-1 in those patients with previous progression on anti-PD-(L)1 therapy or those who have sensitizing EGFR mutations and/or ALK gene rearrangement and have received prior tyrosine kinase inhibitor therapy and 3) augments tumor-specific immune responses unleashed by anti-PD-1. Results from this trial could warrant further clinical investigation to establish the efficacy of the Ad-CCL21-DC/pembrolizumab combination therapy. This combination therapy is anticipated to benefit a large population of patients with advanced NSCLC.

2 STUDY OBJECTIVES AND ENDPOINTS

Hypothesis:

- Intratumoral injection of autologous CCL21 gene-modified DC (Ad-CCL21-DC) when combined with pembrolizumab is safe
- Intratumoral injection of autologous CCL21 gene-modified DC (Ad-CCL21-DC) when combined with pembrolizumab can improve objective response rate in patients with advanced NSCLC
- Intratumoral injection of autologous CCL21 gene-modified DC (Ad-CCL21-DC) will improve tumor-specific immune responses unleashed by PD-1 blockade

Primary Objective

1. Dose escalation: To determine the safety and maximum tolerated dose (MTD) of intratumoral injection of CCL21 gene-modified DC (Ad-CCL21-DC) when combined with intravenous pembrolizumab in 1) patients with advanced non-small cell lung cancer (NSCLC) without a sensitizing epidermal growth factor receptor (EGFR) mutation and/or anaplastic lymphoma kinase (ALK) gene rearrangement who have received prior systemic anti-cancer therapy for advanced disease that includes a PD -1 and/or PD-L1 inhibitor or 2) patients who have sensitizing EGFR mutations and/or ALK gene rearrangements and have received prior tyrosine kinase inhibitor therapy.
2. Dose expansion: To evaluate the objective response rate (ORR) in subjects treated with the dose established during dose escalation (ExD) of intratumoral injection of Ad-CCL21-DC when administered with intravenous pembrolizumab in 1) patients with advanced non-small cell lung cancer (NSCLC) without a sensitizing epidermal growth factor receptor (EGFR) mutation and/or anaplastic lymphoma kinase (ALK) gene rearrangement who have received prior systemic anti-cancer therapy for advanced disease that includes a PD -1 and/or PD-L1 inhibitor or 2) patients who have sensitizing EGFR mutations and/or ALK gene rearrangements and have received prior tyrosine kinase inhibitor therapy.

Secondary Objective

- To define the adverse event (AE) profile of intratumoral injection of Ad-CCL21-DC (determined during dose escalation) when administered with intravenous pembrolizumab in 1) patients with advanced non-small cell lung cancer (NSCLC) without a sensitizing epidermal growth factor

receptor (EGFR) mutation and/or anaplastic lymphoma kinase (ALK) gene rearrangement who have received prior systemic anti-cancer therapy for advanced disease that includes a PD -1 and/or PD-L1 inhibitor or 2) patients who have sensitizing EGFR mutations and/or ALK gene rearrangements and have received prior tyrosine kinase inhibitor therapy.

- To determine drug target activity by analyzing serial pre- and post-treatment biopsies and blood specimens of intratumoral injection of Ad-CCL21-DC (determined during dose escalation) when administered with intravenous pembrolizumab in 1) patients with advanced non-small cell lung cancer (NSCLC) without a sensitizing epidermal growth factor receptor (EGFR) mutation and/or anaplastic lymphoma kinase (ALK) gene rearrangement who have received prior systemic anti-cancer therapy for advanced disease that includes a PD -1 and/or PD-L1 inhibitor or 2) patients who have sensitizing EGFR mutations and/or ALK gene rearrangements and have received prior tyrosine kinase inhibitor therapy.

Primary Endpoints

1. Dose escalation: Establish maximum tolerated dose (MTD) / maximum administered dose (MAD) – the dose level below the one at which more than 1/6 patients experience dose-limiting toxicities (DLT), or Dose Cohort (2), if there is 0 or 1/6 DLT in the cohort.
2. Dose expansion: Define ORR by RECIST v1.1 criteria

Secondary Endpoints

1. Define toxicities – Describe the adverse events using CTC AEv5.0 to grade toxicities
2. Determine effects of treatment on drug target activity – Before each treatment/injections, CT or bronchoscope guided biopsies will be collected. Immunohistochemistry (IHC) will be conducted to assess biomarkers including PD-1, PD-L1, CD8, and CD4 (DC) expression.

3 STUDY DESIGN

A phase I trial of intratumoral administration of CCL21-gene modified dendritic cell (Ad-CCL21-DC) combined with intravenous pembrolizumab for 1) patients with advanced non-small cell lung cancer (NSCLC) without a sensitizing epidermal growth factor receptor (EGFR) mutation and/or anaplastic lymphoma kinase (ALK) gene rearrangement who have received prior systemic anti-cancer therapy for advanced disease that includes a PD -1 and/or PD-L1 inhibitor or 2) patients who have sensitizing EGFR mutations and/or ALK gene rearrangements and have received prior tyrosine kinase inhibitor therapy.. Patients will be enrolled at UCLA Medical Center.

3.1 <u>Safety and Dosing</u>: Patients will be enrolled into two cohorts in the dose escalation phase, each cohort receiving sequentially higher doses of intratumoral injection with Ad-CCL21-DC, and during dose expansion, the patients will be treated at the dose established during dose escalation.

A phase I, non-randomized, dose escalating, multi-cohort trial followed by dose expansion at the dose established during dose escalation (ExD) will be conducted. During dose escalation, a modified 3+3 design will be used in the first dose escalation cohort and six patients will be enrolled in the second dose escalation cohort. Patients enrolled into a given cohort will receive the same Ad-CCL21-DC dose by CT-guided or bronchoscopic intratumoral injection preceded by intravenous pembrolizumab 200 mg on days 0, 21, and 42, and intravenous pembrolizumab 200 mg every three weeks thereafter for up to a year. The Ad-CCL21-DC dose is 1×10^7 cells/injection in the first cohort (1), and will be increased to 3×10^7 cells/injection (2) pending tolerability in the earlier cohort. Dose escalation may proceed only if all 3 patients enrolled in the lower dose cohort experience no DLT or 1 of 6 patients in a cohort has a DLT. If a patient dies within 30 days of

receiving investigational treatment, the study will be held until further evaluation, and potential risks vs. benefits of continuing the study will be discussed with the UCLA JCCC DSMB. If the dose regimen in cohort 1 (Ad-CCL21-DC 1 x 10^7 cells/injection) is not well tolerated, de-escalation to Ad-CCL21-DC 5 x 10^6 cells/injection will be allowed (-1). If the dose regimen specified for Cohort 2 (Ad-CCL21-DC 3 x 10^7 cells/injection) is tolerable (0 or 1 DLT out of six patients), no further dose escalation will be conducted, and this dose level will be defined as maximum administered dose (MAD).

Following the completion of the dose-escalation phase, a dose expansion cohort of 12 patients (D) will be enrolled and treated at ExD for up to a year (note: only intravenous pembrolizumab is given after day 42). All enrolled patients will continue to be followed by a physician, and undergo a history and physical examination every 3 months until progressive disease (PD) or withdrawal from the study. Eligible patients will be assigned to a cohort and will receive intratumoral injections of autologous Ad-CCL21-DC and intravenous pembrolizumab in conjunction with tumor sampling and patient monitoring on the days as detailed in **Table 2**.

DLT is defined as grade 3 or greater toxicity as defined by the NCI Common Toxicity Criteria version 5.0. In the first dose escalation cohort, if 0 of 3 patients have a DLT, then we will escalate to the next dose by enrolling patients into the next cohort. Dose escalation may proceed only if all 3 patients have been enrolled into a lower dose cohort and no DLT is seen over a 28-day period. If 2 or 3 of the 3 have a DLT, the dose will be de-escalated to dose level -1. In the second dose escalation cohort 6 patients will be enrolled and receive the same Ad-CCL21-DC dose. If 2 or more of the 6 patients in dose escalation cohort two have a DLT, then the dose is de-escalated to the previous dose. If we select a dose where only 3 patients have been treated, then an additional 3 patients will be treated at that same dose for a total of six patients at the ExD. In any case, if a patient dies within 30 days of receiving investigational treatment, the study will be held until further evaluation, and potential risks vs. benefits of continuing the study will be discussed with the UCLA JCCC DSMB. In determining the MTD/MAD, no more than 6 patients will be treated at a given dose. The MTD/MAD is defined as the dose level at which fewer than 2 of 6 patients experience a DLT. In the event that 2 or 3 of the 3 have a DLT in the first cohort (A), study accrual will be held. In the unlikely event that dose level 2 requires 6 patients, and de-escalation to a dose level 1 that had enrolled only 3 patients finds 2 DLTs in the additional 3 patients enrolled, we would discuss with the UCLA JCCC DSMB the appropriateness of enrolling at dose level -1. Serious adverse events (SAE) that are not related to study drug (for example disease progression, biopsy complications, or other events not attributed to administration of or reaction to study drug) will not be counted as DLT. If a patient has a complication such as pneumothorax or bleeding after the pre-treatment biopsy, the Ad-CCL21-DC will still be injected unless the risk is considered inappropriate by the study investigator and/or proceduralist. If the risk is determined to be too high for the patient to receive the injection of Ad-CCL21-DC, the patient will remain on pembrolizumab alone. Patients who cannot complete the entire Ad-CCL21-DC dose schedule will be excluded from the MTD/MAD determination, and will be replaced with another participant. Patients withdrawing therapy due to prolonged treatment delays or for reasons other than DLT will be replaced with other subjects until at least 3 evaluable patients have completed treatment in the first dose escalation cohort and at least 6 evaluable patients have completed treatment in the ExD during dose escalation.

Table 2

**Table 1. Drug delivery and immune-monitoring schedule.**

Cohort (# pts)	Anti-PD-1 Dose & Route	**Anti-PD-1 Injection Days**	Ad-CCL21-DC Dose & Route	**Ad-CCL21-DC Injection Days**	**Biopsy Days**	**Safety Monitoring Days**	**Immune Monitoring Days**	**Clinical Response Evaluation Days ****
-1 (6)	200 mg iv	0, 21, 42, then q3wk	5×10^6 IT	0, 21, 42	0, 21, 42	0, 63	0, 11, 21, 42, 63, 126, 189, 252, 315	S, 63, Q3mo
1 (3 or 6)	200 mg iv	0, 21, 42, then q3wk	1×10^7 IT	0, 21, 42	0, 21, 42	0, 63	0, 11, 21, 42, 63, 126, 189, 252, 315	S, 63, Q3mo
2 (6)	200 mg iv	0, 21, 42, then q3wk	3×10^7 IT	0, 21, 42	0, 21, 42	0, 63	0, 11, 21, 42, 63, 126, 189, 252, 315	S, 63, Q3mo
ExD (D) (12)	200 mg iv	0, 21, 42, then q3wk	ExD IT	0, 21, 42	0, 21, 42	0, 63	0, 11, 21, 42, 63, 126, 189, 252, 315	S, 63, Q3mo

** CT imaging to assess tumor burden at patient screening, day 63, and every 3 months following DC injection until progression of disease or withdrawal from study.
S: at time of patient screening. Q3wk: every 3 weeks. Q3mo: every 3 months. ExD: Dose established during dose escalation
All event days are ±7 days

3.2 <u>Biologic and Clinical Responses</u>: All patients will be monitored to assess the clinical efficacy of the treatment and to define potential determinants of the response. Clinical response will be evaluated by CT scans at screening, on day 63, and every 3 months thereafter until progression of disease or patient withdrawal from the study, as detailed in **Table 3** in the clinical protocol. Immune monitoring will be performed using tumor biopsies and peripheral blood samples. We will also utilize tumor biopsies to determine the mutational load and tumor associated neoantigens.

3.2.1 Tumor Biopsy Samples: days 0, 21, 42 (Flow chart 1)

These are listed in order of priority.

1. Multiplex immunofluorescence (MIF) will be utilized to characterize the immune phenotype of the tumor microenvironment (TME). **Table 4** of the clinical protocol lists surrogate markers of immune phenotypes (Parra, Uraoka et al. 2017). Priority will be given to panel 1, with the specific emphasis on CD8 and PD-L1 staining. These assays will be performed with biopsy samples collected on day 0, 21 and 42.

2. Whole Exome Sequencing (WES) will be performed using genomic DNA (gDNA) from pre- and post-treatment tumor samples to identify somatic mutational load and tumor-associated neoantigens. Patient's diagnostic tissue block (or paraffin-embedded day 0 biopsy if such block is not available) and paraffin-embedded day 42 biopsy will be utilized as pre- and post-treatment samples, respectively. Tumor cells will be enriched by laser capture microdissection, followed by gDNA isolation. Patient's gDNA from PBMC will be served as a germline reference in the mutational analyses. These studies will elucidate the evolution of neoantigen landscape following the combination therapy.

Flow Chart 1

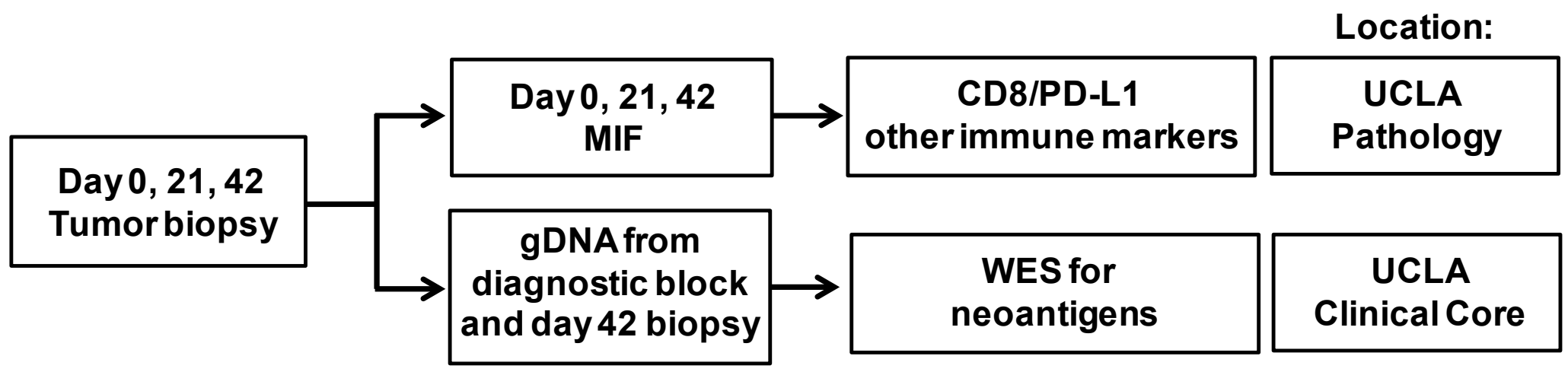

Table 4: Markers of immune phenotypes

Panel 1	Panel 2
AE1/AE3	AE1/AE3
PD-L1	PD-1
CD4	Granzyme B
CD8	CD57
CD3	CD45RO
CD68	FOXP3
DAPI	DAPI

3.2.2 <u>Peripheral Blood Samples: days 0, 11, 21, 42, 63, 126, 189, 252, 315 (Flow chart 2)</u>

1. $CD8^+$ T cells will be sorted from PMBC (collected on day 0, 21, 63, 126, 252) by flow cytometry and their DNA will be isolated using a DNeasy kit (Qiagen). TCR-β chain CDR3 regions will be amplified and deep sequencing will be performed with survey ImmunoSeq assay (Adaptive Biotechnologies) to determine clonality.
2. Mass cytometry (CyTOF) will be used to perform comprehensive immunophenotyping of immune cell subsets from PBMCs (collected on day 0, 11, 42, 189, 315) and elucidate the evolution of immune responses through the course of treatment.
3. As a complementary approach to MIF and CyTOF, Nanostring PanCancer Immune Profiling Panel will be utilized to monitor the anti-tumor immune responses in the study patients. This panel consists of 770 genes and includes 109 genes related to cell surface markers for 24 different immune cell types, as well as over 500 genes that represent all categories of immune response, including key checkpoint blockade genes. It also includes 40 reference genes for data normalization in the expression analysis. To identify leukocyte subpopulations based on gene expression, CYBERSORT method will be utilized(Newman, Liu et al. 2015). Briefly, RNA will be isolated from PBMCs (day 0, 11, 42, 189, 315), using an miRNeasy RNA Isolation Kit (Qiagen). RNA quality and integrity will be assessed by running on an Agilent Bioanalyzer. Two hundred nanograms of RNA will be used for the Nanostring analysis at the UCLA Center for Systems Biomedicine.
4. Quantification of anti-Ad antibody by ELISA, using plasma from day 0, 63 blood samples.
5. Antigen-specific ELISPOT assays to monitor immune responses to HLA-matched TAAs commonly seen in NSCLC patients, using PBMC from day 0, 21, 63, 126, 252.

Flow Chart 2

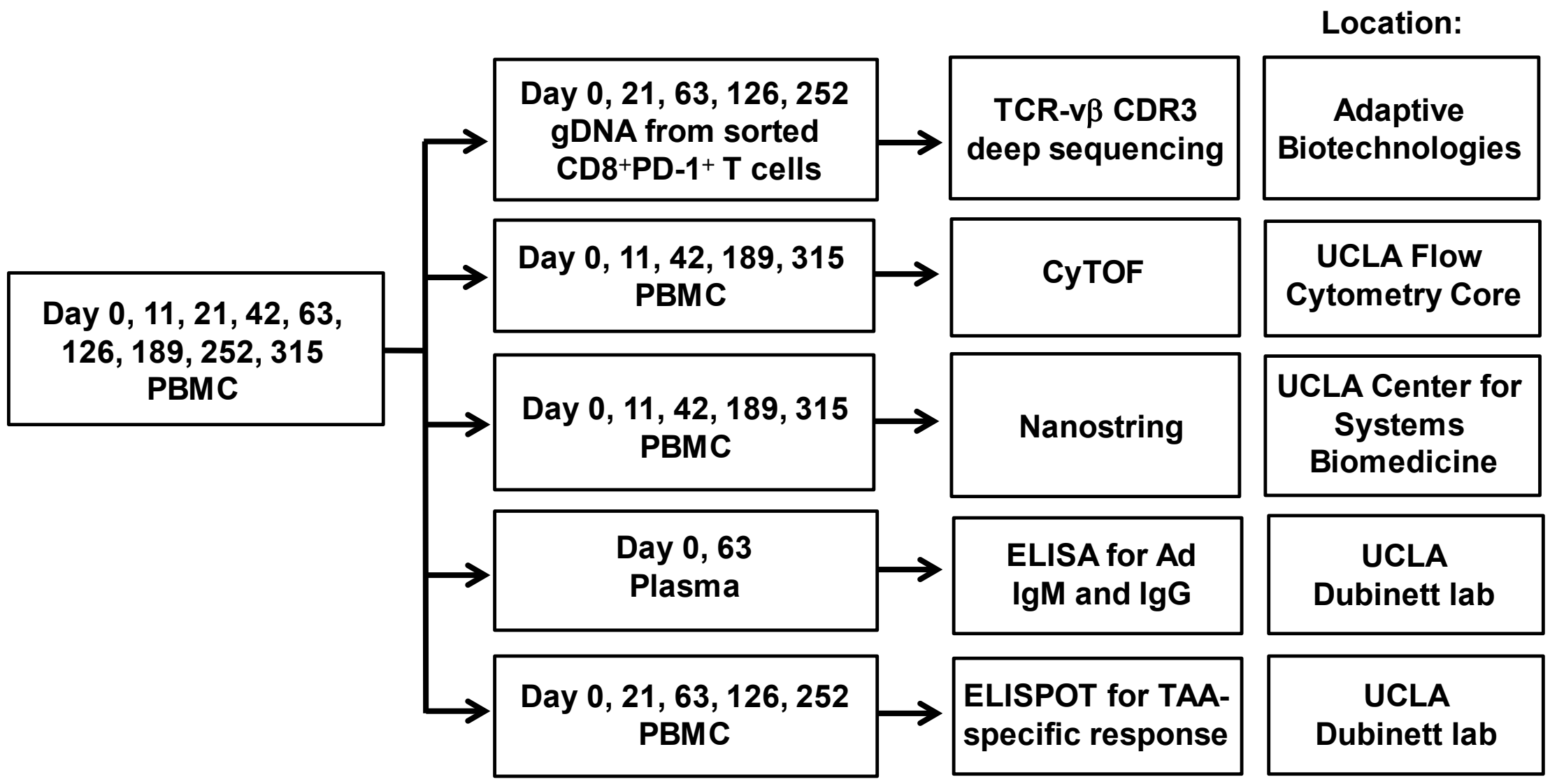

3.2.3 Clinical Responses: Screening, day 63, Q3mo until progression of disease or withdrawal from study

1. CT scans for evaluation of tumor burden (irRECIST Criteria for patient management, RECIST v1.1 for final analysis).
2. Toxicity by NCI Common Toxicity Criteria v 4.0 to define adverse event (AE) associated with the combination therapy of Ad-CCL21-DC and pembrolizumab.

4 PATIENT SELECTION

Sample Size:

Up to 24 patients. During dose escalation, up to 12 patients will be evaluated, 3 or 6 patients at each dose level, depending on the presence or absence of a dose-limiting toxicity (DLT). To confirm safety of the dose established during dose escalation (ExD), if less than 6 patients were evaluated at this dose during dose escalation, the cohort will be expanded to a total of six patients. During dose expansion, 12 patients will be evaluated at the dose established during dose escalation (ExD). From enrollment of the first patient onto the study until the last dose administered to the last subject, this study is anticipated to take approximately 4 years to complete.

4.1 Inclusion Criteria

1. Male/female participants who are at least 18 years of age on the day of signing informed consent with histologically confirmed diagnosis of NSCLC will be enrolled in this study.
2. Stage IV pathologically proven NSCLC.
3. The participant (or legally acceptable representative if applicable) provides written informed consent for the trial.
4. Must have received prior systemic anti-cancer therapy for advanced disease that includes either:
    i. a PD-1 and/or PD-L1 inhibitor in patients without a sensitizing EGFR mutation and/or ALK gene rearrangement or
    ii. Have received prior tyrosine kinase inhibitor therapy in patients with a

sensitizing EGFR mutation and/or ALK gene rearrangement. For patients with sensitizing EGFR mutations, prior therapy must include osimertinib if a T790M mutation in the EGFR gene has been documented, and for patients with ALK gene rearrangements, prior therapy must include a second generation ALK inhibitor (e.g. alectinib, brigatinib, ceritinib). Note: For this group, prior cytotoxic chemotherapy is permitted, but prior therapy with a PD-1 or PD-L1 inhibitor is not permitted.

5. Measurable disease by RECIST v1.1 Guidelines (see **Appendix B**).
6. ECOG performance status of 0, 1(see **Appendix A**).
7. Have adequate organ function as defined in the following table (Table 1).

Table 2 Adequate Organ Function Laboratory Values

System	Laboratory Value
Hematological	
Absolute neutrophil count (ANC)	≥1500/µL
Platelets	≥100 000/µL
Hemoglobin	≥9.0 g/dL or ≥5.6 mmol/L[a]
Renal	
Creatinine OR<br>Measured or calculated[b] creatinine clearance (GFR can also be used in place of creatinine or CrCl)	≤1.5 × ULN OR<br>≥30 mL/min for participant with creatinine levels >1.5 × institutional ULN
Hepatic	
Total bilirubin	≤1.5 ×ULN OR direct bilirubin ≤ULN for participants with total bilirubin levels >1.5 × ULN
AST (SGOT) and ALT (SGPT)	≤2.5 × ULN (≤5 × ULN for participants with liver metastases)
Coagulation	
International normalized ratio (INR) OR prothrombin time (PT)<br>Activated partial thromboplastin time (aPTT)	≤1.5 × ULN unless participant is receiving anticoagulant therapy as long as PT or aPTT is within therapeutic range of intended use of anticoagulants

ALT (SGPT)=alanine aminotransferase (serum glutamic pyruvic transaminase); AST (SGOT)=aspartate aminotransferase (serum glutamic oxaloacetic transaminase); GFR=glomerular filtration rate; ULN=upper limit of normal.

[a] Criteria must be met without erythropoietin dependency and without packed red blood cell (pRBC) transfusion within last 2 weeks.

[b] Creatinine clearance (CrCl) should be calculated per institutional standard.

Note: This table includes eligibility-defining laboratory value requirements for treatment; laboratory value requirements should be adapted according to local regulations and guidelines for the administration of specific chemotherapies.

8. A lesion that either:
    i. Is intended to be accessed bronchoscopically.
       OR
    ii. Is intended to be accessed with CT guided transthoracic injection and in the estimation of the radiologist performing the procedure will not require transversing a bullae that significantly increases the risk of pneumothorax

9. Criteria for known Hepatitis B and C positive subjects

   Hepatitis B and C screening tests are not required unless:

   •Known history of HBV or HCV infection
   •As mandated by local health authority

   9.2 Hepatitis B positive subjects

   Participants who are HBsAg positive are eligible if they have received HBV antiviral therapy for at least 4 weeks and have undetectable HBV viral load prior to randomization.

   Participants should remain on anti-viral therapy throughout study intervention and follow local guidelines for HBV anti-viral therapy post completion of study intervention.

   9.2 Participants with history of HCV infection are eligible if HCV viral load is undetectable at screening.

   Participants must have completed curative anti-viral therapy at least 4 weeks prior to randomization.

10. Male participants:
    i. A male participant must agree to use a contraception as detailed in Appendix C of this protocol during the treatment period and for at least 4 months after the last dose of study treatment and refrain from donating sperm during this period.
11. Female participants:
    i. A female participant is eligible to participate if she is not pregnant (see Appendix C), not breastfeeding, and at least one of the following conditions applies:
        a. Not a woman of childbearing potential (WOCBP) as defined in Appendix 3.
           OR
        b. A WOCBP who agrees to follow the contraceptive guidance in Appendix C during the treatment period and for at least 4 months after the last dose of study treatment.

9.3 <u>Exclusion Criteria</u>

1. Comorbid disease or a medical or psychiatric condition that would impair the ability of the patient to receive or comply with the study protocol.
2. Any use of systemic corticosteroids within 10 days of treatment initiation.
3. Evidence of respiratory failure or inability to tolerate biopsy procedure in the

investigator's opinion.

4. Acute viral, bacterial, or fungal infection, which requires specific therapy. Acute therapy must have been completed at least 7 days prior to study treatment.
5. HIV infected patients (defined as HIV-1/HIV-2 Antibody positive).
6. Concurrent active Hepatitis B (defined as HBsAg positive and/or detectable HBV DNA) and Hepatitis C virus (defined as anti-HCV Ab positive and detectable HCV RNA) infection.
   Note: Hepatitis B and C screening tests are not required unless:
   --Known history of HBV and HCV infection
   --As mandated by local health authority
7. Has a known history of active tuberculosis.
8. Hypersensitivity to any reagents used in the study
9. Pregnancy or inadequate contraception.
10. Lactating females.
11. Clinically active brain metastases, defined as untreated and symptomatic, or requiring therapy with steroids or anticonvulsants to control associated symptoms. In patients with brain metastases that have been treated with radiation therapy, treatment must end at least 14 days prior to starting study treatment. History of leptomeningeal disease is exclusionary regardless of prior therapy.
12. Subjects with organ allografts.
13. Previous or concurrent evidence of autoimmune disease requiring systemic steroids or immunosuppressive agents. Subjects with vitiligo or resolved childhood asthma/atopy would be an exception to this rule. Subjects that require inhaled steroid or local steroid injections will not be excluded from the study. Subjects with hypothyroidism who are managed by hormone replacement will not be excluded from the study.
14. Patients with a history or current evidence of any condition, therapy, or laboratory abnormality that might confound the results of the trial, interfere with the subject's participation for the full duration of the trial, or is not in the best interest of the subject to participate, in the opinion of the treating investigator.

9.4 <u>Concomitant Medications/Vaccinations (Allowed and Prohibited)</u>

All allowed medications or treatments should be kept to a minimum.

Medications or vaccinations specifically prohibited in the exclusion criteria are not allowed during the ongoing trial. If there is a clinical indication for one of these or other medications or vaccinations specifically prohibited during the trial, discontinuation from trial therapy or vaccination may be required. The final decision on any supportive therapy or vaccination rests with the investigator and/or the participant's primary physician.
Acceptable Concomitant Medications

All treatments that the investigator considers necessary for a participant's welfare may be administered at the discretion of the investigator in keeping with the community standards of medical care. All concomitant medication will be recorded on the case report form (CRF) including all prescription, over-the-counter (OTC), herbal supplements, and IV medications and fluids. If changes occur during the trial period, documentation of drug dosage, frequency, route, and date may also be included on the CRF.

All concomitant medications received within 28 days before the first dose of trial treatment and 30 days after the last dose of trial treatment should be recorded. Concomitant medications administered after 30 days after the last dose of trial treatment should be recorded for SAEs and ECIs as defined in Section 11.4-11.5.

Prohibited Concomitant Medications

Participants are prohibited from receiving the following therapies 14 days prior to initiation of therapy and during Treatment Phase (including retreatment for post-complete response relapse) of this trial:

- Antineoplastic systemic chemotherapy or biological therapy
- Immunotherapy not specified in this protocol
- Chemotherapy not specified in this protocol
- Investigational agents other than pembrolizumab
- Radiation therapy
    - Note: Radiation therapy to a symptomatic solitary lesion or to the brain may be allowed at the investigator's discretion.
- Live vaccines within 30 days prior to the first dose of study treatment and while participating in the study. Examples of live vaccines include, but are not limited to, the following: measles, mumps, rubella, varicella/zoster, yellow fever, rabies, BCG, and typhoid vaccine. Seasonal influenza vaccines for injection are generally killed virus vaccines and are allowed; however, intranasal influenza vaccines (eg, FluMist®) are live attenuated vaccines and are not allowed.
- Systemic glucocorticoids for any purpose other than to modulate symptoms from an event of clinical interest of suspected immunologic etiology. The use of physiologic doses of corticosteroids may be approved after consultation with the Sponsor.

Participants who, in the assessment by the investigator, require the use of any of the aforementioned treatments for clinical management should be removed from the study. All treatments that the Investigator considers necessary for a participant's welfare may be administered at the discretion of the Investigator in keeping with the community standards of medical care.

Medications or vaccinations specifically prohibited in the exclusion criteria are not allowed during the ongoing study. If there is a clinical indication for any medication or vaccination specifically prohibited during the study, discontinuation from study therapy or vaccination may be required. The final decision on any supportive therapy or vaccination rests with the investigator and/or the participant's primary physician. However, the decision to continue the participant on study treatment requires the mutual agreement of the investigator, the Sponsor and the participant.
There are no prohibited therapies during the Post-Treatment Follow-up Phase.

5 INFORMATION ON DRUGS AND BIOLOGIC AGENTS

5.1 Pembrolizumab

5.1.1 Chemical Properties: Pembrolizumab is a humanized anti–programmed cell death 1 (anti–PD-1) monoclonal antibody (mAb) of the immunoglobulin G4 (IgG4)/kappa isotype with a stabilizing S228P sequence alteration in the fragment crystallizable (Fc) region. Pembrolizumab binds to human PD-1 and blocks the interaction between PD-1 and its ligands. The theoretical molecular weight of the polypeptide is 146,288 daltons (Da) and its theoretical isoelectric point (pI) is 7.5. The parental murine anti–human PD-1 antibody (hPD-1.09Å) was produced by immunizing mice with hPD-1 DNA. The pembrolizumab antibody was generated by humanization of the parental antibody by the Medical Research Council (Cambridge, UK) using complementarity determining region grafting technology (U.S. Patent No. 5,225,539). The gene segments encoding the variable heavy and light chains of pembrolizumab, as well as human IgG4, were codon-optimized, synthesized, and ligated into a vector. A single expression plasmid, pAPD11V1-GA, was constructed for the expression of both the heavy and light antibody chains of pembrolizumab. The nucleotide sequences encoding the heavy and light chains, along with their respective promoters and poly A signal sequences, have been confirmed by DNA sequence analysis. The pAPD11V1-GA expression vector was subsequently used to transfect CHO-DXB-11 cells for the development of the pembrolizumab-producing cell line.

The nomenclature of pembrolizumab drug substance is provided below:

Code Name	MK-3475 (Anti-PD-1)
Other Code Name	02P106, ORG 307448-0, SCH 900475 (Anti–PD1)
Chemical Name	Humanized X PD-1_mAb (H409A11) IgG4
CAS Number	1374853-91-4
CAS Name	Anti-(human protein PDCD1 (programmed cell death 1)) immunoglobulin G4 (human-Mus musculus monoclonal heavy chain) disulfide with human-Mus musculus monoclonal light chain, dimer
USAN and INN	Pembrolizumab
Trade Name	KEYTRUDA®

5.1.2 Structure: Pembrolizumab is a highly selective humanized mAb designed to block the interaction between PD-1 and its ligands, programmed cell death ligand 1 (PD-L1) and programmed cell death ligand 2 (PD-L2). Pembrolizumab is an IgG4/kappa isotype with a stabilizing sequence alteration in the Fc region. The theoretical molecular weights of the heavy and light chains derived from the amino acid sequences, excluding glycosylation, are 49.4 kiloDaltons (KDa) and 23.7 KDa, respectively. The antibody is heterogeneously glycosylated at asparagine 297 within the Fc domain of each heavy chain, yielding molecular weights typically ranging between 148.9 and 149.5 KDa, depending on the attached glycan chains.

5.1.3 Physiochemical Properties: Pembrolizumab drug substance (DS) is produced at several locations to yield: a partially formulated aqueous solution stored under refrigerated (2° to 8°C) conditions at a concentration of 40.0 to 50.0 mg/mL in 10 millimolar (mM) histidine buffer, pH 5.2 to 5.8, and a fully formulated aqueous solution stored frozen (–40°C) at a concentration of 22.5 to 27.5 mg/mL in 10 mM histidine buffer, pH 5.2 to 5.8, containing 7% sucrose and 0.02% polysorbate 80. The drug substance solution from both sources is a clear to opalescent liquid. The manufacturing process for pembrolizumab is

a suspension cell culture process that uses commercially available animal component-free medium. The contents of the production bioreactor are depth filtered to remove intact cells and cell debris and then 0.2 micron (µm) filtered into presterilized bags. The purification process for pembrolizumab drug substance consists of the following steps: 3 chromatography steps, 1 viral inactivation step, 1 viral filtration step, 1 ultrafiltration/diafiltration step, 1 formulation step (only for the fully formulated DS stored frozen), and 1 final 0.2 µm filtration step.

5.1.4 Pharmaceutical Formulation: Two drug product (DP) dosage forms are available for pembrolizumab: a white to off-white lyophilized powder, 50 mg/vial, and a liquid, DP 100 mg/vial, both in Type I glass vials intended for single use only.

- Pembrolizumab Powder for Solution for Infusion, 50 mg/vial (manufactured using the partially formulated DS), is reconstituted with sterile water for injection prior to use. Pembrolizumab DP is formulated with L-histidine as a buffering agent, polysorbate 80 as surfactant, and sucrose as a stabilizer/tonicity modifier and may contain hydrochloric acid and/or sodium hydroxide for pH adjustment (if necessary).
- Pembrolizumab Solution for Infusion 100 mg/vial is a liquid DP (manufactured using the fully formulated DS with L-histidine as buffering agent, polysorbate 80 as surfactant, and sucrose as stabilizer/tonicity modifier).

Both DP dosage forms are stored under refrigerated conditions (2° to 8°C).
The lyophilized DP after reconstitution with sterile water for injection and the liquid DP are clear to opalescent solutions, essentially free of visible particles. The reconstituted lyophilized product and the liquid product are intended for intravenous (IV) administration. The reconstituted DP solution or the liquid DP can be further diluted with normal saline or 5% dextrose in the concentration range of 1 to 10 mg/mL in IV containers made of polyvinyl chloride (PVC) or non-PVC material. Reconstituted vials should be used immediately to prepare the infusion solution in the IV bag, and the infusion solution should be administered immediately. Diluted pembrolizumab solutions may be stored at room temperature for a cumulative time of up to 4 hours. This includes room temperature storage of admixture solutions in the IV bags and the duration of infusion. In addition, IV bags can be stored at 2° to 8°C for up to a cumulative time of 20 hours. This recommendation is based on up to 24 hours of room temperature and up to 24 hours of refrigerated stability data of diluted pembrolizumab solutions in the IV bags.

5.2 Ad-CCL21-DC

5.2.1 Facilities, infrastructure, and oversight (UCLA Human Gene and Cell Therapy Facility (HGCTF) for Gene Therapy Cell Product Manufacture):

UCLA has a dedicated Human Gene and Cell Therapy Facility (HGCTF) specifically designed for cellular and gene transfer studies (BioSafety level 2; 7 HEPA-filtered rooms). This secured area has 24/7 centralized monitoring of air and equipment. Access is restricted to investigators and staff with certified training and is supervised byHGCTF quality assurance personnel. The quality system for the facility includes the appropriate management of out-of-specification (OOS) lab results and investigations, non-conforming materials and products, equipment and process deviations, change control, corrective and preventive actions, product review and release, compliance audits and audit programs (internal/external). There have been

at least 20 investigator-initiated clinical trials in which the cell or gene transfer product has been manufactured in this facility.
This trial will be overseen by the Jonsson Comprehensive Cancer Center Data and Safety Monitoring Board (JCCC DSMB). Primary JCCC DSMB functions include: developing a plan for monitoring adverse events, setting initial criteria for termination of the study for toxicity, and enforcing these set criteria. Oncology trials overseen by the JCCC DSMB are subject to real time monitoring and routine audits by the JCCC Office of Regulatory Compliance (ORC). The Compliance Officer also monitors all aspects of the trial and reports to the JCCC DSMB on a continua basis. Monitoring and audit reports are also forwarded to the UCLA IRB.

Recombinant Human GM-CSF and IL-4

5.2.2 Supplier: Recombinant human GM-CSF (leukine) will be obtained from UCLA Pharmacy, which is manufactured and tested for clinical use under the Current Good Manufacturing Practices (cGMP) guidelines by Sanofi. Recombinant human IL-4 will be supplied by Miltenyi under cGMP guidelines.

5.2.3 Description: GM-CSF is a highly purified recombinant human glycoprotein expressed in *S. cervis, which can stimulate the proliferation and differentiation of granulocyte-macrophage precursor cells,* and, in combination with IL-4, results in the differentiation of monocytes into DC.

5.2.4 Clinical Activity: Leukine™ (rHuGM-CSF) is FDA-approved for intravenous or subcutaneous injection into patients for the purposes of stem cell mobilization, myeloid reconstitution following myeloablative chemotherapy, and bone marrow transplantation (Paglia, Chiodoni et al. 1996; Porgador, Snyder et al. 1996; Reeves, Royal et al. 1996). In this protocol, GM-CSF will be used in combination with IL-4 for the *ex vivo* preparation of autologous DC as previously described (Riedl, Baratelli et al. 2003).

5.2.5 Route of Administration: GM-CSF and IL-4 will only be used to grow DC *in vitro* at a concentration of 800 U/ml and 400 U/ml, respectively.

5.2.6 Storage and Stability: GM-CSF and IL-4 will be stored as a lyophilized powder at - 80°C and protected from light, and reconstituted aseptically using sterile RPMI and stored at -80°C for *in vitro* use.

5.3 cGMP-Ad-CCL21 Virus Derivative

5.3.1 Supplier: Our proposal of IT Ad-CCL21-DC combined with IV anti-PD-1 for NSCLC patients has recently been accepted into NCI Experimental Therapeutics (NExT) Program. Dr. Jason Yovandich has been assigned as the NCI project leader for our proposal. We have received a lot of fully tested and quantified GMP-grade replication-deficient adenovirus expressing CCL21 (cGMP-Ad-CCL21) from NExT (Lot L0901002) prior to the initiation of this trial. In addition, we have obtained cGMP-Ad-CCL21 (Lot L0604006) manufactured by BDP/SAIC-Frederick, Inc. SAIC-Frederick, Inc. is a contractor for the National Cancer Institute (NCI-Frederick) (Frederick, Maryland), which is a government-owned, contractor operated facility.

5.3.2 Description: The cGMP-Ad-CCL21 is a purified first generation recombinant replication-defective (E1/E3-deleted), serotype 5 adenovirus derivative. From the materials transferred to *BDP/SAIC-Frederick, Inc*, the plaque purified virus seed lot VL20041117A was produced in the Virus Production Facility at the BDP. This virus was then plaque purified 2 additional times, and amplified following the third plaque purification. In process testing did not reveal contamination. Ad-CCL21 (***Final Vialed Product LotL0604006***) was

generated in a cGMP-validated HEK293 Host Cell System. The HEK293 cell line is an immortalized line of primary human embryonal kidney cells transformed by sheared human adenovirus type 5 (Ad 5) DNA. The HEK293 Master Cell Bank (MCB) Lot 2003-0025 was manufactured and tested at Magenta Corporation (Rockville, MD). Magenta Corporation obtained the HEK293 cells from American Type Culture Collection (ATCC®, ATCC® Number: CRL-1573™). The virus production utilized HEK293 Master Cell Bank (MCB) Lot 31872, which was derived from MCB Lot 2003-0025 (Magenta Corporation, Rockville, MD) as the starting material at SAICFrederick,Inc. in the Cell Culture Production Laboratory/BDP. HEK293 cells were expanded through seven passages (3/21/2006 –4/14/2006). The HEK293 cells produced in the final passage (passage 7) were transferred to the Virus Production Facility and placed into incubators set at 37°C, 5% CO2, and 80% humidity, for use in virus production. The 250 mL conical tubes were removed from ≤ -70°C storage and the resuspended virus was thawed at room temperature for approximately 4 hours. The virus was centrifuged at a setting of 4°C and 3500 x g for 20 minutes. The supernatant (clarified viral lysate) was transferred into three PETG bottles (Bottle 1: 120 mL, Bottle 2: 120 mL, Bottle 3: 80 mL) and placed in MMIC ≤ -70°C controlled storage. This batch was purified by 2 CsCl-density gradients, and dialyzed. After in process testing was negative, the final biological substance was sterile filtered and bottled 6/6/2006. The Ad-CCL21 Biological Substance Lot L0604006 was manually vialed in a Class 100 biological safety cabinet (BSC) by aliquotting 1.1 mL ± 3.6% per container (2.87 x 1011 vp/mL) into 1.5 mL cryovials (Nalgene Catalog Number 5000-1020). A total of 579 vials were filled. Lot L0604006 Ad-CCL21 has a titer of 2.46 x 10^{10} pfu/mL, was tested to indicate a viral particle (vp) to plaque-forming unit (pfu) ratio of 11.67, and there was no detectable Replication Competent Adenovirus (RCA) in 3 x 10^{10} vp. Lot L0901002 Ad-CCL21 was tested at 120 months post production on 07/25/19 and found to have a titer of 2.00 x 10^{10} pfu/mL, a viral particle concentration of 3.65 x 10^{11} vp/ml, and a viral particle (vp) to plaque-forming unit (pfu) ratio of 18.3.

5.3.3 <u>Ad-CCL21 Construct Schematic:</u>

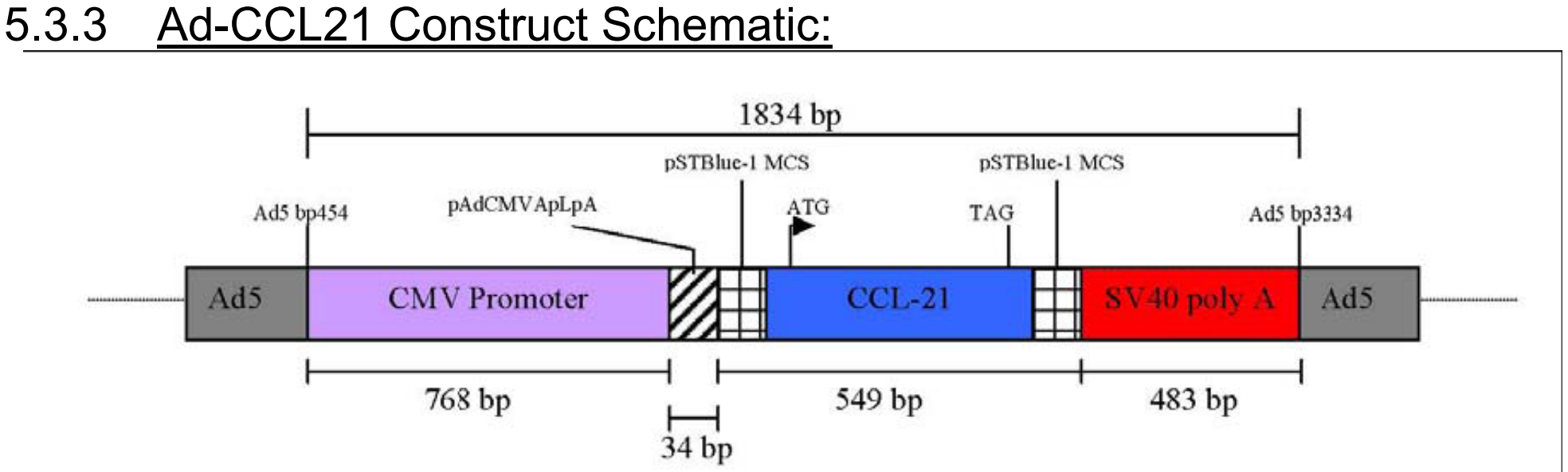

5.3.4 <u>Route of Administration</u>: Ad-CCL21 will only be used to transduce DC *in vitro* and will not be directly administered to patients.

5.3.5 <u>Storage and Stability</u>: The final cGMP Ad-CCL21 product was manufactured by the NeXT program; the bulk was 0.22 µm filtered and vialed (1.1 mL fill volume) for single use. The final product is stored at ≤ −70°C in single vials containing 1.1 ml Ad-CCL21-suspension at a concentration of (3.65 x 10^{11} vp/mL) in GST buffer (20 mM Tris, 25 mM NaCl, 25% glycerol, pH 8.0). The stability of Ad-CCL21 is verified annually by Leidos Biomedical Research, Inc. coordinated by NCI. These analyses will be ongoing alongside the

implementation of the clinical trial.

5.4 Preparation of autologous DC

5.4.1 Collection of PBMC from leukapheresis and cryopreservation: At least 9 days prior to the first Ad-CCL21-DC injection, a single four-hour leukapheresis procedure will be performed in the Hemapheresis Unit. If not enough autologous dendritic cells are generated from the first leukapheresis procedure, a second leukapheresis procedure would be done after the first injection of Ad-CCL21-DC. Whole blood (100mL) will also be drawn at the time of leukapheresis for autologous serum preparation. If a patient has poor venous access, catheter placement may be performed. This will be done on a case by case basis. Samples from the leukapheresis product prior to Ficoll centrifugation will be sent for sterility testing, as described in section 5.6 Biosafety Testing. Peripheral blood mononuclear cells (PBMC) will be isolated from the leukapheresis product by density-gradient centrifugation on Ficoll-Paque™ PLUS (GE Healthcare). PBMC will be proceeded to DC production, or cryopreserved at 0.25-1 x 10^8 cells/ml of DMSO containing freezing media (10% DMSO + 20% autologous serum + 70% RPMI-1640) in controlled rate freezing cryo-containers, then stored at –80°C.

5.4.2 DC culture/Differentiation of monocytes into DC: All cell processing will be conducted in UCLA HGCTF,. At least 9 days prior to the first Ad-CCL21-DC administration into the patient, fresh PBMC will be used for DC production. Cells will be plated at 5-20 x 10^6 cells/ml (200-400 x 10^6 cells per 20-30 ml per T175 flask) in DC culture media (Cellgenix). After allowing adherence for 1-2 hours at 37°C, nonadherent cells are gently removed by washing with normal saline. Adherent cells are cultured in DC culture media for 6-7 days in a 37°C, 5% $C0_2$ incubator in the presence of rhGM-CSF (800 U/ml) and rhIL-4 (400 U/ml). After 4 days of DC culturing (Protocol Day –2, +19, or +40), fresh DC culture media containing rhGM-CSF (800 U/ml) and rhIL-4 (400 U/ml) will be added to each flaskand 1 ml of supernatant from each flask, will be harvested for retention in case an investigation is needed. .

5.5 Preparation of Ad-CCL21-DC

5.5.1 DC transduction with Ad-CCL21: Following 6-7 days of culturing, DC will be harvested from the T175 flasks by gently pipetting from culture, washed in serum free RPMI, and resuspended in serum free RPMI to make 2-6 x 10^6 cells/ml. Ad-CCL21 will be added to the DC at MOI=100:1. The cell suspension will be centrifuged at 2000 x *g* at RT for two hours (Nishimura, Nishioka et al. 2001). Following centrifugation, the Ad-CCL21-DC will be washed with normal saline and resuspended in 5% culture media (RPMI+5% autologous serum). The cells are counted in trypan blue and the appropriate numbers ($5x10^6$, $1x10^7$, or $3x10^7$ Ad-CCL21-DC per injection depending on dose cohort; enough cells for one injection per vial) are resuspended and frozen in vaporized liquid nitrogen utilizing CryoStor CS10, which is a pre-formulated, serum and protein-free and cGMP manufactured freezing medium designed to prepare and preserve cells in ultra-low temperature environments (-70ºC to -196ºC). Ad-CCL21-DC cells will also be frozen in 1-2 quality control (QC) vials, and all QC tests will be performed on a QC vial thawed 1-5 days post freezing as detailed in Section 5.6. We will also freeze a retention sample in CS10 in case a future investigation is needed. If not enough Ad-CCL21-DC cells are obtained for all 3 injections, then second leukapheresis will be performed on Day 11 or 32, and the same Ad-

CCL21-DC manufacturing will be performed as detailed above.

5.5.2 Preparation of Ad-CCL21-DC for intratumoral injection: On the day of patient injection (Day 0, +21, or +42), the frozen Ad-CCL21-DC will be thawed from liquid nitrogen, washed in 5% culture media, counted by trypan blue dye exclusion and the cell number to be injected per cohort will be brought to a standard volume of 1.2 ml in 5% culture media. Ad-CCL21-DC will be loaded in a sterile syringe and injected into the patient's lung cancer by CT-guided or bronchoscopic delivery.

5.6 Biosafety Testing

5.6.1 Ad-CCL21 Testing: cGMP Ad-CCL21 vector was provided through RAID by SAIC-Frederick, Inc. Yearly recertification will be provided by Leidos Biomedical Research , Inc. coordinated by NCI.

5.6.2 Leukapheresis Testing: An aliquot of the leukapheresis product will be tested for sterility testing (negative up to 14 days).

5.6.3 Autologous Serum Testing: Autologous patient serum will be collected at the time of leukapheresis and heat inactivated (56˚C, 30 minutes). After heat inactivation, a 0.1-0.2 ml aliquot will be sent for sterility testing. The remaining autologous serum will be used for preparing 5% RPMI autologous culture media and freezing media, or aliquoted into tubes and placed into long-term storage at –80°C.

5.6.4 Tests to be performed with QC aliquot for product release:

5.6.4.1 DC Phenotypic Analysis: Ad-CCL21-DC will be phenotypically characterized using flow cytometry. They will be examined for MHC class II (HLA-DR) and CD86 costimulatory molecule expression, and DC will be identified by gating on the large granular cells (LGC) that are positive for both MHC class II and CD86 (≥ 30% for releasing)..

5.6.4.2 Culture for sterility: Acceptance Criteria for our final product will be a negative sterility culture after 14 days. However, these results may be available after infusion of the final product. 5.6.4.3 Endotoxin assay: Evaluation of endotoxin contamination by Limulus Amebocyte Lysate (LAL, Quantitative Chromogenic Procedure): Properly diluted sample are tested using a Endosafe-PTS kit to quantitatively determine endotoxin (Gram -LPS) chromogenically. Samples are diluted withRPMI media. Positive and negative controls (with endotoxin spiked samples) are jointly analyzed on an Endosafe-PTS reader (Charles River Laboratories) with a sensitivity of 0.01 endotoxin units (EU)/ml. The Acceptance Criteria will be <5.0 EU/Kg weight of the study subject.

5.6.4.4 DC Viability Analysis: Acceptance Criteria for Ad-CCL21-DC will be ≥ 70% viability by trypan blue exclusion.

5.6.4.5 MycoAlert assay: Mycoplasma detection will be performed using the Lonza MycoAlert Mycoplasma detection kit. A ratio in the luminescence readings before and after the addition of the MycoAlert substrate is used to determine if the cell culture is contaminated by Mycoplasma. A ratio < 1 is considered negative with absent mycoplasma contamination.

5.6.4.6 Reserve DC samples: Cryopreserve 0.5-1x10^6 Ad-CCL21-DC cells in CS10 freezing medium as retention sample. Untransduced DC (2-10 x 10^6) and the remaining Ad-CCL21 exceeding the cells for a given

dose and needed for release criteria tests - will be used for functional assays to ensure proper DC production.

5.6.5 Product Labeling: The final product will be labeled with the Patient Trial Number, Patient Medical Record Number (MRN), Vaccine Number, manufacturing Date and "expiration" date and time (24 hours after preparation), and "Not Tested for Biohazards."

5.6.6 Lot Release Criteria:

The Criteria for Lot Release of Ad-CCL21-DC:

Sample	Testing	Lot Release Criteria
0 Leukapheresis	Sterility	Negative up to 14 days
0 Serum	Sterility	Negative up to 14 days
Ad-CCL21-DC QC tube	Mycoplasma test	Negative
Ad-CCL21-DC QC tube	DC Viability	≥ 70% Viability
Ad-CCL21-DC QC tube	DC Phenotype	CD86 + MHC Class II ≥ 30% LGC
Ad-CCL21-DC QC tube	Sterility	Submitted
Ad-CCL21-DC QC tube	DC Endotoxin Assay	≤ 5 EU/Kg

Release criteria on the day on infusion (Day 0, 21, 42):
DC viability: ≥ 70%
DC Endotoxin assay: ≤ 5 EU/Kg
DC sterility: submitted (negative up to 14 days, which will be obtained at a later date)

6. TREATMENT PLAN

At least nine days before the first DC injection (Protocol Day -9), blood is collected from the patient and leukapheresis is performed. If a patient has poor venous access, catheter placement may be performed. This will be done on a case by case basis. PBMC are obtained from Ficoll-Hypaque centrifugation, and the cells are cryopreserved or proceed to the first DC production. PBMC are cultured in DC media with rGM-CSF and rIL-4 for 6-7 days to allow monocyte differentiation to DC DC are then transduced with Ad-CCL21 and frozen in CS10 in aliquots enough for one IT injection in addition to QC aliquots, and store in -80 followed by transfer to liquid nitrogen. The QC vial is tested for DC phenotyping, sterility, endotoxin, mycoplasma and viability by trypan blue staining as the criteria of lot release of Ad-CCL21-DC vaccines. On the days of infusion, frozen AD-CCL21-DC will be washed with 5% culture media, and cells will be counted and subjected to viability, sterility and endotoxin assays. Up to 100mL of blood is drawn from patients for safety and immunologic studies, and the lung tumor is injected with Ad-CCL21-DC by CT-guidance or bronchoscopy (Protocol Day 0, +21, and +42). The optimal approach for delivery of Ad-CCL21-DC

will be determined on an individual basis by the medical oncology investigator, generally in consultation with investigators from thoracic surgery, pulmonary, and/or radiology. Ad-CCL21-DC injection is preceded by treatment with 200 mg intravenous pembrolizumab. Needle aspirate or core biopsy is performed just prior to injection on the same day (Protocol Day 0, +21, and +42) to allow collection of samples from the tumor. Peripheral blood samples are drawn for safety monitoring (on Days 0, 63) and immunologic studies (on Days 0, 11, 21, 42, 63, 126, 189, 252, and 315). Radiologic imaging is obtained to assess for treatment-related changes in tumor burden at Screening, Day 63, and every 3 months until progression of disease or patient withdrawal from the study. If a patient demonstrates progression of disease on evaluation of the tumor burden on Day 63, then further CT scans or bronchoscopies are no longer obtained. Eligible patients will be assigned to a cohort and will receive intratumoral injections of autologous Ad-CCL21-DC and intravenous pembrolizumab in conjunction with tumor sampling and patient monitoring on the days as detailed in **Table 2**. All enrolled patients will continue to receive intravenous pembrolizumab every 3 weeks until progressive disease (PD) or withdrawal from the study for up to 1 year of treatment.

In the event that the first leukapheresis procedure generates an inadequate number of Ad-CCL21-DC for injection and as a result does not meet the intended dose defined by the cohort the patient was assigned to the patient will still receive the intratumoral injection, at the investigator's discretion, but at a dose lower than the cohort dose. Patients who receive IT Ad-CCL21-DC at lower than the cohort dose on Day 0, as well as those patients in whom it is anticipated that not enough cells have been generated from the first leukapheresis procedure to administer the cohort defined dose on Days 21 and 42, the patient will undergo a second leukapheresis procedure at Day 11 or 32. If a patient receives less than 2 of 3 IT injections (Day 0, 21, 42) at their cohort defined dose, he or she will not be included in the cohort analysis for MTD determination, but will be included in the secondary analyses of clinical and biological responses. During dose escalation, an additional patient will be enrolled to replace the patient that did not receive the appropriate dose in order to allow complete enrolment into each cohort and to allow every patient in a cohort to receive the same dose of Ad-CCL21-DC.

Fig. 5. Protocol Summary

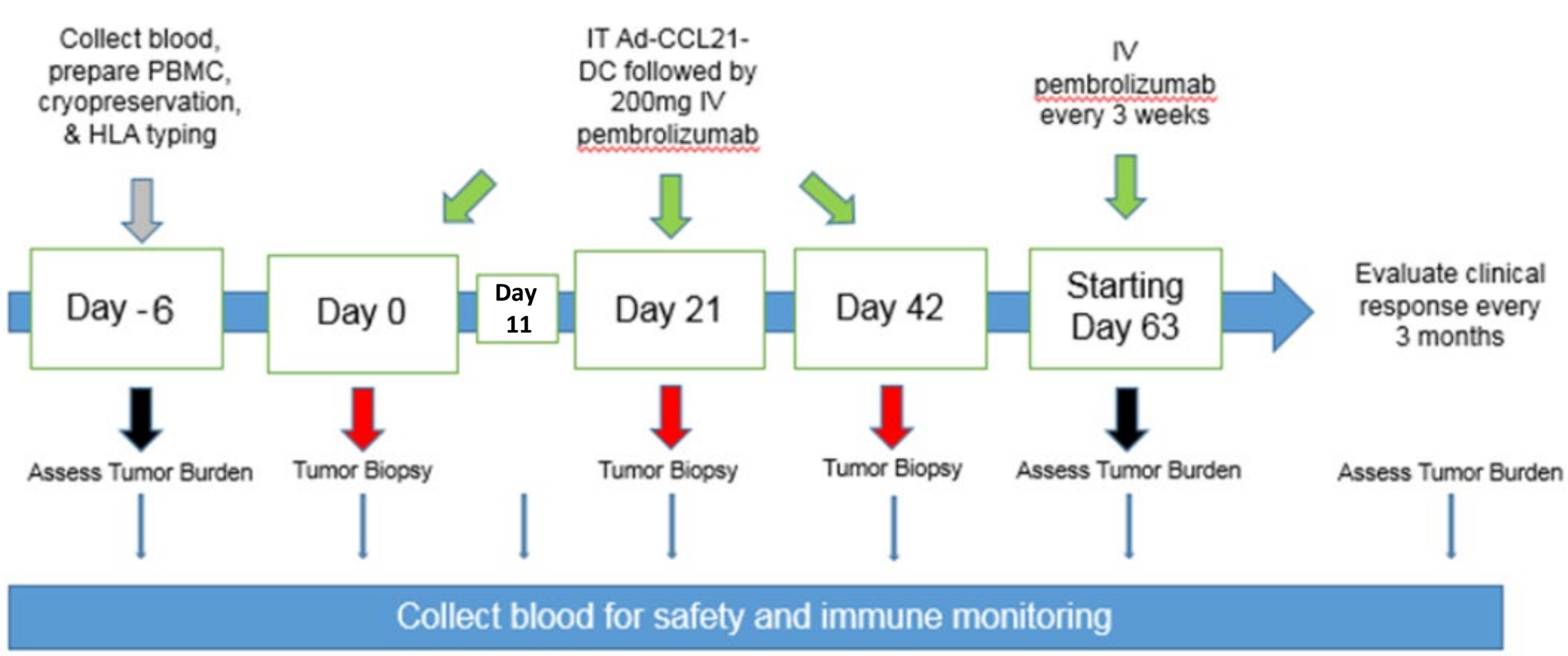

6.1 Bronchoscopy and intratumoral injection of DC

6.1.1 Preparation for bronchoscopy: Bronchoscopy will be performed as an outpatient procedure in a state of the art bronchoscopy suite. The rooms are fully staffed with personnel and equipped for interventional bronchoscopy. Patients will have complete screening laboratory work verifying that they have adequate renal function, liver function, white blood cell counts, platelet counts, PT/PTT, and pulmonary function. Patients will have fasted for at least 6 hours prior to the study. A trained bronchoscopy nurse will check patients in and confirm that all vital signs are normal (systolic blood pressure >90 and <150 mmHg, diastolic blood pressure <90mm Hg, heart rate <100/min, temperature <38°C, respiratory rate <25/min, and oxygen saturation >90% on room air). Patients are attached to continuous monitoring (ECG, non-invasive blood pressure monitoring, and pulse oximetry) and provided with oxygen at 2-6 liters/min via nasal cannula. To ablate the gag-reflex, patients are treated with a hand held nebulizer containing 3 ml of 2 or 4% lidocaine followed by topical spraying with 20% benzocaine or 4% lidocaine as needed. Due to their smoking histories, some patients may be treated with albuterol 2.5 mg and atrovent 1.0 mg by hand-held nebulizer as indicated by the clinical situation at the time of the procedure. Conscious sedation will be administered in accordance with institutional Conscious Sedation Guidelines using intravenous anxiolytics (e.g. midazolam in 0.5-1 mg aliquots) and analgesics (e.g. fentanyl 25 mg aliquots).

6.1.2 Visual inspection: A fiberoptic video-bronchoscope will be introduced orally or transnasally by physicians trained and certified in the procedure. The larynx, trachea, and bilateral airways (mainstem, lobar, and segmental bronchi) will be systematically visualized. Two ml aliquots of 2% lidocaine will be applied to the bronchial mucosa at selected sites if additional topical anesthesia is required. The amount of lidocaine that is administered in this manner is recorded and will not exceed a 4 mg/kg or total dose of 280 mg, in order to prevent lidocaine toxicity.

6.1.3 Needle biopsy and intratumoral injection of Ad-CCL21-DC: Only patients with endobronchial lung cancers may be candidates for bronchoscopic injections. Patients with a major endobronchial lesion in the segmental, lobar, or mainstem bronchus with complete obstruction of the airway may be eligible for bronchoscopic injection if there is no evidence of respiratory failure or inability to tolerate biopsy procedure in the investigator's opinion.Patients with an endobronchial lesion in the segmental bronchus with variable stenosis (not completely obstructed) may be eligible for bronchoscopic injection if there is no evidence of respiratory failure, as previously described. In addition, central tumors with extrinsic airway compression with no endobronchial involvement will not be approached with a bronchoscopic delivery but rather a CT-guided trans-thoracic technique. Bronchoscopy will entail complete visual inspection of bilateral airways. If these anatomic inclusion criteria are met, the bronchoscope will be advanced to the endobronchial tumor. The exact site and orientation of the mass with respect to the bronchoscope will be captured on videotape. Still color prints will be stored as a computerized image on an overlying grid. By matching visual airway landmarks and by comparing live images with stored images using an overlying grid, the operator will assure that the same tumor location is inspected, biopsied, and injected on subsequent bronchoscopy. Prior to injecting Ad-CCL21-DC, four needle biopsies will be performed using a 18 or 19 -gauge needle depending on the tumor size to obtain core biopsies of the tumor for *in vitro* monitoring studies. The mass will be visually divided into 4 quadrants and at least 2 needle biopsies will be obtained from each of these

quadrants depending on tumor size and location. Patients will receive 4 injections of Ad-CCL21-DC, one in each quadrant, using a 22-gauge transbronchial cytology needle (13 mm needle length and 144 cm working length and diameter of 1.8 mm). The dead space of the cytology needle assembly will be carefully filled with the Ad-CCL21-DC suspension and 0.25 ml of cell suspension injected into each site. Prior to injecting Ad-CCL21-DC, gentle aspiration will be applied to ensure that the needle tip has not been placed into a vascular structure. The day 0, 21, and 42 bronchoscopies will be performed by the same physician when possible in order to reduce inter-bronchoscopist variability and assure repeat sampling at the same site. Alternatively, instead of using the Wang transbronchial needle, biopsies may be obtained with the use of bronchoscopic biopsy forceps or by endobronchial ultrasound guided needle. If a patient has a complication such as pneumothorax or bleeding after the pre-treatment biopsy, the Ad-CCL21-DC will still be injected unless the risk is considered inappropriate by the study investigator and/or proceduralist. If the risk is determined to be too high for the patient to receive the injection of Ad-CCL21-DC, the patient will remain on pembrolizumab alone. Patients who cannot complete the entire Ad-CCL21-DC dose schedule will be excluded from the MTD/MAD determination, and will be replaced with another participant. The vaccine may be delivered by direct bronchoscopy or endobronchial ultrasound guidance.

6.1.4 Post-bronchoscopy monitoring: At the completion of the procedure, the bronchoscope will be removed and the patient will be placed with the head of the bed elevated. Vital signs will be monitored every 15 minutes in conjunction with continuous ECG and pulse oximetry, for at least 2 hours or until the patient is awake, alert, and stable. Prior to discharge, all patients will undergo a post-injection chest x-ray. Patients will be discharged home with a complete list of follow-up instructions and contact numbers in case an emergency should arise.

6.2 CT-guided intratumoral injection of Ad-CCL21-DC

6.2.1 Preparation for CT-guided procedure: CT-guided procedures will be performed as an outpatient procedure in the state of the art, interventional radiology suite. A board-certified interventional radiologist of UCLA's Department of Radiology will perform all trans-thoracic CT-guided injections of Ad-CCL21-DC. The feasibility of CT-guided injection of immunotherapy has recently been demonstrated at our institution. Patients will have complete screening laboratory work verifying that they have adequate renal function, liver function, white blood cell counts, platelet counts, PT/PTT, and pulmonary function. All CT scans will be performed on a GE High Speed Advantage scanner (GE Medical Systems, Milwaukee, WI). Limited axial scans will be performed through the target lesion at 3mm, 5mm, or 10mm collimation, depending on the lesion size. Scans will be done before, during, and after needle placement to plan needle placement approach and confirm successful and uncomplicated injection. All patients will be prepped and draped in the usual sterile fashion. 1% lidocaine will be used to achieve local anesthesia. No sedation is planned. Patients will be monitored with respect to temperature, pulse, and blood pressure pre- and post-procedure. Oxygen saturation and blood pressure will be monitored throughout the procedure.

6.2.2 Needle biopsy and intratumoral injection: For lung tumors >3 cm in size, the mass will be divided into 4 equal quadrants, and each quadrant will undergo biopsy and Ad-CCL21-DC injection. For tumors <3 cm in size, the mass will undergo biopsy

and injection at only a single site. Depending on size and location of the tumor, either a set of 17 G needle with 18 G gun or 19 G needle with 20 G gun will be used to obtain a core biopsy specimen. Following insertion of the needle tip into a selected target lesion, aspiration will be applied to the syringe to confirm no entry into vascular structures. At least two core biopsies of the lung tumor from each quadrant will be obtained depending on tumor size and location. A 22-gauge Chiba spinal needle inserted through the 19-gauge outer needle will be used to deliver Ad-CCL21-DC. A total volume of 1 ml of Ad-CCL21-DC will be injected equally into 4 separate quadrants or at a single site depending on the size of the tumor, followed by a 1 ml of normal saline flush administration. With all transpleural intrapulmonary injections, 2-5 ml of autologous blood will be administered along the parenchymal needle tract as a blood patch with withdrawal of the needle. If a pneumothorax occurs due to the transthoracic injection, when feasible the pneumothorax will be evacuated as the injection needle is withdrawn. In the case of a pneumothorax or bleeding after biopsies, Ad-CCL21-DC will still be injected unless the risk is considered inappropriate by the study investigator and/or proceduralist. If the risk is too high, the patient will remain on pembrolizumab alone. Patients who cannot complete the entire Ad-CCL21-DC dose schedule will be excluded from the MTD/MAD determination, and will be replaced with another participant.

6.2.3 Post-procedure monitoring: At the completion of the procedure, the patient's vital signs will be monitored every 15 minutes after injection for at least 1 hour and up to 4 hours. Prior to discharge, all patients will undergo a post-injection chest x-ray. Patients will be discharged home with a complete list of follow-up instructions and contact numbers in case an emergency should arise.

7 TREATMENT MODIFICATION AND DISCONTINUATION ACCORDING TO LEVEL OF TOXICITY

The National Cancer Institute (NCI) Common Toxicity Criteria Scale v 4.0 will be used to define grades (severity) of toxicity (see **Appendix C**).

7.1 Treatment Modification and General Management of Toxicities

7.1.1 For any Grade 1 toxicity, there will be no dose modification.

7.1.2 If Grade 2 toxicity develops, the investigator may elect to continue the intratumoral treatment with careful monitoring, or to withhold the treatment until values return to Grade 1 or less, then restart the treatment. One of the expected grade 2 toxicities associated with lung tumor injections is the iatrogenic introduction of a pneumothorax. If a pneumothorax occurs, the patient may be monitored, or have the pneumothorax evacuated by needle thoracostomy. A recalcitrant or enlarging pneumothorax may necessitate chest tube placement. This is an expected complication, which we do not anticipate will necessitate halting further injections. Accordingly, if the pneumothorax resolves with thoracostomy evacuation within 72 hrs of the injection, and is not radiographically apparent at the time of the next injection, the protocol will proceed as prescribed.

7.1.3 If Grade 3 toxicity is observed that is likely attributable to the biological effects of DC, further administration will be withheld until the toxicity is reduced to less than grade 2, and the investigator evaluates the subject to determine clinical acceptability for continuing the protocol. Treatment can resume at the discretion of the investigators, but with a reduction in the dose of intratumoral DC by 50%. If the observed grade 3

toxicity is an expected procedural complication (e.g.: a persistent air leak that has not responded to thoracostomy drainage at 7 days), intratumoral treatment will be withheld until the complication resolves. If the complication resolves between days 7-14 following the initial vaccine administration, and investigator evaluates the subject to be clinically acceptable for continuing with the protocol, then the vaccine administration will resume at full dose.

7.1.4 Delays of administration > 2 weeks due to toxicity, or repeat Grade 3 toxicity despite dose reduction will result in subject discontinuation of the protocol. Subjects who have been discontinued due to delays of administration will be replaced until the necessary number of patients to be evaluated is met.

7.1.5 Any Grade 4 toxicity will result in removal of the subject from the protocol, continued observation and treatment of the subject as indicated by the clinical situation. The development of Grade 4 toxicity in any one subject in a cohort, thought to be related to the protocol treatment, will result in termination of treatment at that dosing level, and definition of the MTD/MAD at the dose administered in the prior cohort.

7.1.6 Any subject death within 30 days of study drug administration will result in the study to be held, until evaluation of the cause is determined. If the death is attributed to the protocol treatment, further treatment in all subjects will cease, and the study will be discontinued.

7.2 Symptomatic Therapy for Toxicity

Symptomatic therapy such as analgesics or other helpful therapy may be administered if deemed necessary by the investigators. All medications administered will be recorded in the appropriate Case Report Form.

7.3 Pembrolizumab Dose Modification and Toxicity Management due to Immunogenic Adverse Events

Adverse events associated with pembrolizumab exposure may represent an immunologic etiology. These immune-related AEs (irAEs) may occur shortly after the first dose or several months after the last dose of pembrolizumab treatment and may affect more than on body system simultaneously. Therefore, early recognition and initiation of treatment is critical to reduce complications. Based on existing clinical study data, most irAEs were reversible and could be managed with interruptions of pembrolizumab, administration of corticosteroids and/or other supportive care. For suspected irAEs, ensure adequate evaluation to confirm etiology or exclude other causes. Additional procedures or tests such as bronchoscopy, endoscopy, skin biopsy may be included as part of the evaluation. Based on the severity of irAEs, withhold or permanently discontinue pembrolizumab and administer corticosteroids. Dose modification and toxicity management guidelines for irAEs associated with pembrolizumab are provided in Table 5. See Section 7.3.1 for supportive care guidelines, including use of corticosteroids.

Table 5: Dose Modification and Toxicity Management Guidelines for Immune-Related Adverse Events Associated with Pembrolizumab

General instructions:

1. Corticosteroid taper should be initiated upon AE improving to Grade 1 or less and continue to taper over at least 4 weeks.
2. For situations where pembrolizumab has been withheld, pembrolizumab can be resumed after AE has been reduced to Grade 1 or 0 and corticosteroid has been tapered. Pembrolizumab should be permanently discontinued if AE does not resolve within 12 weeks of last dose or corticosteroids cannot be reduced to ≤10 mg prednisone or equivalent per day within 12 weeks.
3. For severe and life-threatening irAEs, IV corticosteroid should be initiated first followed by oral steroid. Other immunosuppressive treatment should be initiated if irAEs cannot be controlled by corticosteroids.

Immune-related AEs	Toxicity grade or conditions (CTCAEv5.0)	Action taken to pembrolizumab	irAE management with corticosteroid and/or other therapies	Monitor and follow-up
Pneumonitis	Grade 2	Withhold	• Administer corticosteroids (initial dose of 1-2 mg/kg prednisone or equivalent) followed by taper	• Monitor participants for signs and symptoms of pneumonitis<br>• Evaluate participants with suspected pneumonitis with radiographic imaging and initiate corticosteroid treatment<br>• Add prophylactic antibiotics for opportunistic infections
	Grade 3 or 4, or recurrent Grade 2	Permanently discontinue		
Diarrhea / Colitis	Grade 2 or 3	Withhold	• Administer corticosteroids (initial dose of 1-2 mg/kg prednisone or	• Monitor participants for signs and symptoms of enterocolitis

	Grade 4	Permanently discontinue	equivalent) followed by taper	(ie, diarrhea, abdominal pain, blood or mucus in stool with or without fever) and of bowel perforation (ie, peritoneal signs and ileus).<br>• Participants with ≥ Grade 2 diarrhea suspecting colitis should consider GI consultation and performing endoscopy to rule out colitis.<br>• Participants with diarrhea/colitis should be advised to drink liberal quantities of clear fluids. If sufficient oral fluid intake is not feasible, fluid and electrolytes should be substituted via IV infusion.
AST / ALT elevation or Increased bilirubin	Grade 2	Withhold	• Administer corticosteroids (initial dose of 0.5- 1 mg/kg prednisone or equivalent) followed by taper	• Monitor with liver function tests (consider weekly or more frequently until liver enzyme value returned to baseline or is stable
	Grade 3 or 4	Permanently discontinue	• Administer corticosteroids (initial dose of 1-2 mg/kg prednisone or	

			equivalent) followed by taper	
Type 1 diabetes mellitus (T1DM) or Hyperglycemia	Newly onset T1DM or Grade 3 or 4 hyperglycemia associated with evidence of β-cell failure	Withhold	• Initiate insulin replacement therapy for participants with T1DM<br>• Administer anti-hyperglycemic in participants with hyperglycemia	• Monitor participants for hyperglycemia or other signs and symptoms of diabetes.
Hypophysitis	Grade 2	Withhold	• Administer corticosteroids and initiate hormonal replacements as clinically indicated.	• Monitor for signs and symptoms of hypophysitis (including hypopituitarism and adrenal insufficiency)
	Grade 3 or 4	Withhold or permanently discontinue[1]		
Hyperthyroidism	Grade 2	Continue	• Treat with non-selective beta-blockers (eg, propranolol) or thionamides as appropriate	• Monitor for signs and symptoms of thyroid disorders.
	Grade 3 or 4	Withhold or permanently discontinue[1]		
Hypothyroidism	Grade 2-4	Continue	• Initiate thyroid replacement hormones (eg, levothyroxine or liothyroinine) per standard of care	• Monitor for signs and symptoms of thyroid disorders.
Nephritis and Renal dysfunction	Grade 2	Withhold	• Administer corticosteroids (prednisone 1-2 mg/kg or equivalent) followed by taper.	• Monitor changes of renal function
	Grade 3 or 4	Permanently discontinue		
Myocarditis	Grade 1 or 2	Withhold	• Based on severity of AE administer corticosteroids	• Ensure adequate evaluation to confirm etiology and/or
	Grade 3 or 4	Permanently discontinue		

				exclude other causes
All other immune-related AEs	Intolerable/ persistent Grade 2	Withhold	• Based on type and severity of AE administer corticosteroids	• Ensure adequate evaluation to confirm etiology and/or exclude other causes
	Grade 3	Withhold or discontinue based on the type of event. Events that require discontinuation include and not limited to: Gullain-Barre Syndrome, encephalitis		
	Grade 4 or recurrent Grade 3	Permanently discontinue		

1. Withhold or permanently discontinue pembrolizumab is at the discretion of the investigator or treating physician.

NOTE:

For participants with Grade 3 or 4 immune-related endocrinopathy where withhold of pembrolizumab is required, pembrolizumab may be resumed when AE resolves to ≤ Grade 2 and is controlled with hormonal replacement therapy or achieved metabolic control (in case of T1DM).

7.3.1 Supportive Care Guidelines

Subjects should receive appropriate supportive care measures as deemed necessary by the treating investigator. Suggested supportive care measures for the management of adverse events with potential immunologic etiology are outlined below. Where appropriate, these guidelines include the use of oral or intravenous treatment with corticosteroids as well as additional anti-inflammatory agents if symptoms do not improve with administration of corticosteroids. Note that several courses of steroid tapering may be necessary as symptoms may worsen when the steroid dose is decreased. For each disorder, attempts should be made to rule out other causes such as metastatic disease or bacterial or viral infection, which might require additional supportive care. The treatment guidelines are intended to be applied when the investigator determines the events to be related to pembrolizumab.

Note: if after the evaluation the event is determined not to be related, the investigator is instructed to follow the ECI reporting guidance but does not need to follow the treatment guidance. Refer to Table 3 for dose modification.

It may be necessary to perform conditional procedures such as bronchoscopy, endoscopy, or skin photography as part of evaluation of the event.

- Pneumonitis:
    - For Grade 2 events, treat with systemic corticosteroids. When symptoms improve to Grade 1 or less, steroid taper should be started and continued over no less than 4 weeks.

- For Grade 3-4 events, immediately treat with intravenous steroids. Administer additional anti-inflammatory measures, as needed.
  - Add prophylactic antibiotics for opportunistic infections in the case of prolonged steroid administration.
- Diarrhea/Colitis:

  Subjects should be carefully monitored for signs and symptoms of enterocolitis (such as diarrhea, abdominal pain, blood or mucus in stool, with or without fever) and of bowel perforation (such as peritoneal signs and ileus).
  - All subjects who experience diarrhea/colitis should be advised to drink liberal quantities of clear fluids. If sufficient oral fluid intake is not feasible, fluid and electrolytes should be substituted via IV infusion. For Grade 2 or higher diarrhea, consider GI consultation and endoscopy to confirm or rule out colitis.
  - For Grade 2 diarrhea/colitis that persists greater than 3 days, administer oral corticosteroids.
  - For Grade 3 or 4 diarrhea/colitis that persists > 1 week, treat with intravenous steroids followed by high dose oral steroids.
  - When symptoms improve to Grade 1 or less, steroid taper should be started and continued over no less than 4 weeks.
- Type 1 diabetes mellitus (if new onset, including diabetic ketoacidosis [DKA]) or ≥ Grade 3 Hyperglycemia, if associated with ketosis (ketonuria) or metabolic acidosis (DKA)
  - For T1DM or Grade 3-4 Hyperglycemia
    - Insulin replacement therapy is recommended for Type I diabetes mellitus and for Grade 3-4 hyperglycemia associated with metabolic acidosis or ketonuria.
    - Evaluate patients with serum glucose and a metabolic panel, urine ketones, glycosylated hemoglobin, and C-peptide.
- Hypophysitis:
  - For Grade 2 events, treat with corticosteroids. When symptoms improve to Grade 1 or less, steroid taper should be started and continued over no less than 4 weeks. Replacement of appropriate hormones may be required as the steroid dose is tapered.
  - For Grade 3-4 events, treat with an initial dose of IV corticosteroids followed by oral corticosteroids. When symptoms improve to Grade 1 or less, steroid taper should be started and continued over no less than 4 weeks. Replacement of appropriate hormones may be required as the steroid dose is tapered.
- Hyperthyroidism or Hypothyroidism:

  Thyroid disorders can occur at any time during treatment. Monitor patients for changes in thyroid function (at the start of treatment, periodically during treatment, and as indicated based on clinical evaluation) and for clinical signs and symptoms of thyroid disorders.
  - Grade 2 hyperthyroidism events (and Grade 2-4 hypothyroidism):
    - In hyperthyroidism, non-selective beta-blockers (e.g. propranolol) are suggested as initial therapy.
    - In hypothyroidism, thyroid hormone replacement therapy, with levothyroxine or liothyroinine, is indicated per standard of care.
  - Grade 3-4 hyperthyroidism

        - Treat with an initial dose of IV corticosteroid followed by oral corticosteroids. When symptoms improve to Grade 1 or less, steroid taper should be started and continued over no less than 4 weeks. Replacement of appropriate hormones may be required as the steroid dose is tapered.
- Hepatic:
    - For Grade 2 events, monitor liver function tests more frequently until returned to baseline values (consider weekly).
        - Treat with IV or oral corticosteroids
    - For Grade 3-4 events, treat with intravenous corticosteroids for 24 to 48 hours.
    - When symptoms improve to Grade 1 or less, a steroid taper should be started and continued over no less than 4 weeks.
- Renal Failure or Nephritis:
    - For Grade 2 events, treat with corticosteroids.
    - For Grade 3-4 events, treat with systemic corticosteroids.
    - When symptoms improve to Grade 1 or less, steroid taper should be started and continued over no less than 4 weeks.

7.4 Dose Modification and Toxicity Management of Infusion-Reactions Related to Pembrolizumab

Pembrolizumab may cause severe or life threatening infusion-reactions including severe hypersensitivity or anaphylaxis. Signs and symptoms usually develop during or shortly after drug infusion and generally resolve completely within 24 hours of completion of infusion. Dose modification and toxicity management guidelines on pembrolizumab associated infusion reaction are provided in Table 6.

Table 6: Pembrolizumab Infusion Reaction Dose Modification and Treatment Guidelines

**NCI CTCAE Grade**	**Treatment**	**Premedication at Subsequent Dosing**
**Grade 1**<br>Mild reaction; infusion interruption not indicated; intervention not indicated	Increase monitoring of vital signs as medically indicated until the participant is deemed medically stable in the opinion of the investigator.	None
**Grade 2**<br>Requires therapy or infusion interruption but responds promptly to symptomatic treatment (e.g., antihistamines, NSAIDs, narcotics, IV fluids); prophylactic medications indicated for ≤24 hrs	**Stop Infusion.**<br>Additional appropriate medical therapy may include but is not limited to:<br>IV fluids<br>Antihistamines<br>NSAIDs<br>Acetaminophen<br>Narcotics<br>Increase monitoring of vital signs as medically indicated until the participant is deemed medically stable in the opinion of the investigator.<br>If symptoms resolve within 1 hour of stopping drug infusion, the infusion may be restarted at 50% of the original infusion rate (e.g. from 100 mL/hr to 50 mL/hr). Otherwise dosing will be held until symptoms resolve and the participant should be premedicated for the next scheduled dose.<br>**Participants who develop Grade 2 toxicity despite adequate premedication should be permanently discontinued from further study drug treatment**	Participant may be premedicated 1.5h (± 30 minutes) prior to infusion of _____ with:<br>Diphenhydramine 50 mg po (or equivalent dose of antihistamine).<br>Acetaminophen 500-1000 mg po (or equivalent dose of analgesic).

**Grades 3 or 4**<br>Grade 3:<br>Prolonged (i.e., not rapidly responsive to symptomatic medication and/or brief interruption of infusion); recurrence of symptoms following initial improvement; hospitalization indicated for other clinical sequelae (e.g., renal impairment, pulmonary infiltrates)<br>Grade 4:<br>Life-threatening; pressor or ventilatory support indicated	**Stop Infusion.**<br>Additional appropriate medical therapy may include but is not limited to:<br>Epinephrine**<br>IV fluids<br>Antihistamines<br>NSAIDs<br>Acetaminophen<br>Narcotics<br>Oxygen<br>Pressors<br>Corticosteroids<br>Increase monitoring of vital signs as medically indicated until the participant is deemed medically stable in the opinion of the investigator.<br>Hospitalization may be indicated.<br>**In cases of anaphylaxis, epinephrine should be used immediately.<br>**Participant is permanently discontinued from further study drug treatment.**	No subsequent dosing
Appropriate resuscitation equipment should be available at the bedside and a physician readily available during the period of drug administration.<br>For further information, please refer to the Common Terminology Criteria for Adverse Events v5.0 (CTCAE) at http://ctep.cancer.gov		

8 CLINICAL, LABORATORY, & BIOLOGIC/IMMUNOLOGIC EVALUATIONS

All exams, tests, and procedures may occur ± 4 business days and are outlined in Table 5.

Table 3—Schedule of Events

Schedule of Events (all events have a ±7 day window)									
Day	Screen	-6	0	11	21	42	Starting Day 63, and Q 3 weeks f/u until treatment discontinuation (up to 1 year)	Discontinuation Visit	Follow up (Q3 months)
Informed Consent	X								
Disease Status									X
Physician Visit	X	X	X	X	X	X	X	X	
History and Physical	X	X	X	X	X	X	X	X	
ECOG	X		X	X	X	X	X	X	
Placement of catheter (if clinically indicated on a case by case basis)	X								
ECG (repeated if symptoms suggest)	X		X		X	X			
Pulse oximetry	X		X		X	X			
Leukapheresis[3]		X[2]		X[2]					
Blood for Safety Testing (post-injections)			X				X[1]		
Blood for CBC	X		X	X	X	X	X	X	
Blood for Chemistries/Creatinine	X		X	X	X	X	X	X	
Blood for LFTs	X		X	X	X	X	X	X	
Blood for PT, PTT	X		X	X	X	X	X	X	
Blood for thyroid testing	X		X	X	X	X	X	X	
Pregnancy test (female only)	X								
Urinalysis	X								
HIV, Hepatitis B and Hepatitis C sAg	X								
Blood for biologic/ immunologic tests			X	X	X	X	X[1]		
Tumor aspirate or biopsy			X		X	X			
Imaging to assess tumor burden & response	X						X[1]		
CT or MRI of brain (if symptoms suggestive of CNS metastasis)	X								
DC treatment			X		X	X			
Pembrolizumab treatment			X		X	X	X		

[1]Imaging done every 3 months to assess tumor burden and response. Safety Monitoring blood draws done on Day 63; and Immune Monitoring blood draws done on Day 63, 126, 189, 252, 315.
[2]The first leukapheresis will be performed prior to day -9. A second leukapheresis is to be done only if it is anticipated that not enough cells have been generated from the first leukapheresis procedure or if a patient receives IT Ad-CCL21-DC at lower than the cohort dose on Day 0. Of note, the second leukapheresis can occur on Day 11 or 32.
[3]100mL blood will be drawn at the time of leukapheresis for autologous serum preparation.

8.1 <u>Screening and Baseline Evaluations (see Table 3)</u>

Screening for eligibility will take place within 45 days prior to Day 0. The baseline evaluation will take place between day –14 and day 0 of the study, prior to intratumoral administration of DC. All subjects will be thoroughly informed about the required evaluations, testing, procedures, and the

follow-up schedule. Written informed consent will be obtained from all subjects prior to screening.

8.1.1 History and Physical Examination: A complete history and physical exam will be performed at initial screening and a targeted physical exam will be repeated at the baseline evaluation.
8.1.2 ECOG Performance Status (see **Appendix A**): screening and baseline
8.1.3 Tumor Burden Evaluation: Evaluation of tumor burden with appropriate imaging of measurable disease (see **Appendix B**).
8.1.4 Urinalysis: Routine urinalysis.
8.1.5 Serum Chemistries: Serum chemistries including uric acid, calcium, phosphorus, magnesium, amylase, transaminases (SGOT/SGPT), alkaline phosphatase, LDH, total bilirubin, BUN, creatinine, electrolytes, and glucose.
8.1.6 Hematology: Complete blood count (CBC), differential, platelets, and coagulation tests, prothrombin time and partial thromboplastin time (PT and PTT).
8.1.7 CT or MRI of the brain: If symptoms suggest CNS metastasis.
8.1.8 Safety and Immunologic testing: Peripheral blood for baseline measurements.
8.1.9 HIV1/HIV2 antibody testing
8.1.10 Hepatitis B surface antigen testing
8.1.11 Hepatitis C antibody testing and quantitative HCV RNA
8.1.12 Pregnancy test: Child bearing age females only. Serum pregnancy test must be negative at screening.

8.2 Interval Clinical Evaluations (see Table 3)

Evaluations will take place during screening, on days 0, 11, 21, 42, 63, and every three weeks thereafter. There will be additional repeating evaluations every 3 months until progressive disease (PD) or withdrawal from the study.

8.2.1 History and Physical Examination: Interval history and targeted physical exam will be performed when indicated.
8.2.2 ECOG Performance Status: Documented at each study visit
8.2.3 Serum Chemistries: include uric acid, calcium, phosphorus, magnesium, amylase, transaminases, alkaline phosphatase, LDH, total bilirubin, BUN, creatinine, electrolytes, and glucose.
8.2.4 Hematology: CBC, differential and platelets, PT and PTT.
8.2.5 Thyroid Testing: TSH, Free T3, and T4.
8.2.6 Safety and Immunologic testing: Peripheral blood for interval measurements, taken on days 0, 11, 21, 42, 63, 126, 189, 252, and 315.

8.3 Tumor Burden Evaluation

Potential studies to assess tumor burden include CT scan of the chest, abdomen, and pelvis, conventional chest or bone radiograph, bone scan, and/or PET scan. For patients undergoing DC treatment, scans will be performed at screening, Day 63 and every three months thereafter until disease progression or withdrawal from the protocol. Additional studies will be performed as indicated for each patient depending upon the state and extent of their disease.

8.4 Post-Treatment Evaluation

After treatment discontinuation, patients will be monitored for study follow up by telephone every

3 months until withdrawal from the study or death. Procedures are outlined in Table 3.

9 EVALUATION OF RESPONSE

9.1 Clinical Response

Subjects will have serial measurements of evaluable target lesions and non-target lesions that will be graded according to both the immune related and standard RECIST v1.1 guidelines prior to Day 0, on Day 63, and every 3 months thereafter until disease progression or withdrawal from the protocol (Therasse, Arbuck et al. 2000)(see **Appendix B**).

Target Lesions

9.1.1 Complete Response (CR): Disappearance of all target lesions

9.1.2 Partial Response (PR): At least a 30% decrease in the sum of the longest diameter (LD) of target lesions, taking as reference the baseline sum LD

9.1.3 Progressive Disease (PD): At least a 20% increase in the sum of the LD of target lesions, taking as reference the smallest sum LD recorded since the treatment started or the appearance of one or more new lesions.

9.1.4 Stable Disease (SD): Neither sufficient shrinkage to qualify for PR nor sufficient increase to qualify for PD, taking as reference the smallest sum LD since the treatment started.

Non-target Lesions

9.1.5 Complete Response (CR): Disappearance of all non-target lesions and normalization of tumor marker level.

9.1.6 Incomplete Response/Stable Disease (SD): Persistence of one or more non-target lesion(s) or/and maintenance of tumor marker level above the normal limits.

9.1.7 Progressive Disease (PD): Appearance of one or more new lesions and/or unequivocal progression of existing non-target lesion. Although a clear progression of "non-target" lesions only is exceptional, in such circumstances, the opinion of the treating physician should prevail and the progression status should be confirmed later on by the review panel (or study chair).

10 CRITERIA FOR DISCONTINUATION OF STUDY PATIENTS

10.1 Criteria For Treatment Discontinuation

Participation in this study will be discontinued in the events described in section 7, and may be discontinued for any of the following reasons listed below*:

10.1.1 At the subject's request: Any patient who wishes to withdraw from the study may do so at any time. They will also be counseled that long-term follow-up for the effects of immunotherapy are strongly desired by the medical facility and federal regulatory groups.**

10.1.2 A major, unexpected, or life-threatening toxicity requiring that treatment be discontinued.

10.1.3 Generalized impairment or mental incompetence, which would render the patient unable to understand his/her participation in the study.**

10.1.4 If, in the Investigator's medical judgment, further participation would be injurious to the subject's health or well-being.**

10.1.5 Administrative reasons, such as subject noncompliance or a major protocol violation.**

*An explanation will be recorded for any patient that is taken off treatment and the appropriate Case Report Form completed.
**Subjects discontinued for these reasons will be replaced, until the necessary number of patients for evaluation of study treatment is met.

10.2 Criteria for Study Discontinuation

The study can be discontinued by the investigators, sponsor, RAC, or the FDA.

11 ADVERSE EXPERIENCES

11.1 Adverse Experience Reporting

The investigator or qualified designee will assess each subject to evaluate for potential new or worsening AEs as specified in the Trial Flow Chart and more frequently if clinically indicated. Adverse experiences will be graded and recorded throughout the study and during the follow-up period according to NCI CTCAE Version 5.0 (see Section 14.2). Toxicities will be characterized in terms regarding seriousness, causality, toxicity grading, and action taken with regard to trial treatment.

For subjects receiving treatment with pembrolizumab all AEs of unknown etiology associated with pembrolizumab exposure should be evaluated to determine if it is possibly an event of clinical interest (ECI) of a potentially immunologic etiology (termed immune-related adverse events, or irAEs).

HHS Guidelines for Research Involving Recombinant DNA Molecules: It is the responsibility of the institution and those associated with it to adhere to the intent of the *Guidelines* as well as to their specifics.

11.2 Assessing and Recording Adverse Events

An AE is defined as any unfavorable medical event experienced by a clinical investigation subject who has been administered a pharmaceutical product or has undergone a procedure. AE does not necessarily have to have a causal relationship with the treatment. An AE can therefore be any unfavorable and unintended sign (including an abnormal laboratory finding, for example), symptom, or disease temporally associated with the use of a medicinal product or protocol-specified procedure, whether or not considered related to the product or procedure. In the context of this study, we will include any worsening (i.e., any clinically significant adverse change in frequency and/or intensity) of a preexisting condition that is temporally associated with the administration of Ad-CCL21-DC or pembrolizumab as an AE.
We will monitor AE in two phases. Phase one will constitute safety monitoring during the treatment, where ELISA for Adenovirus-specific IgM and IgG antibodies will be performed with patient plasma, using the blood samples collected on day 0, 7, and 28. The second phase of AE monitoring will occur during clinical response evaluations, which will be performed on screening day, day 63 and once every 3 months thereafter until progression of the disease or patient withdrawal from the study. Toxicity by NCI Common Toxicity Criteria will be applied to define AEs associated with the combination therapy of Ad-CCL21-DC and pembrolizumab. All AEs will be

monitored and reported to regulatory authorities and IRB/IECs in accordance with all applicable global laws and regulations.
Changes resulting from normal growth and development that do not vary significantly in frequency or severity from expected levels are not to be considered adverse events. Examples of this may include, but are not limited to, teething, typical crying in infants and children, and onset of menses or menopause occurring at a physiologically appropriate time.
Pembrolizumab (Merck) refers to the investigational biological product marketed, manufactured by, licensed by, provided by or distributed by Merck for human use.
Adverse events may occur during the course of the use of either product in clinical trials or within the follow-up period specified by the protocol, or prescribed in clinical practice, from overdose (whether accidental or intentional), from abuse, and from withdrawal.
Adverse events may also occur in screened subjects during any pre-allocation baseline period as a result of a protocol-specified intervention, including washout or discontinuation of usual therapy, diet, placebo treatment, or a procedure.
Progression of the cancer under study is not considered an adverse event unless it is considered to be drug related by the investigator.
All adverse events will be recorded from the time the consent form is signed through 30 days following cessation of treatment and at each examination on the Adverse Event case report forms/worksheets, regardless of attribution to study drug. The reporting timeframe for adverse events meeting any serious criteria is described in section 11.5.1.

11.3 Definition of an Overdose for This Protocol and Reporting of Overdose to the UCLA DSMB and to Merck

For purposes of this trial, an overdose of pembrolizumab will be defined as any dose of 1,000 mg or greater (≥5 times the indicated dose). No specific information is available on the treatment of overdose of pembrolizumab. Appropriate supportive treatment should be provided if clinically indicated. In the event of overdose, the subject should be observed closely for signs of toxicity. Appropriate supportive treatment should be provided if clinically indicated.
If an adverse event(s) is associated with (“results from”) the overdose of pembrolizumab, the adverse event(s) is reported as a serious adverse event, even if no other seriousness criteria are met.
If a dose of pembrolizumab meeting the protocol definition of overdose is taken without any associated clinical symptoms or abnormal laboratory results, the overdose is reported as a non-serious Event of Clinical Interest (ECI), using the terminology “accidental or intentional overdose without adverse effect.”
All reports of overdose with and without an adverse event must be reported within 24 hours to the UCLA DSMB and within 2 working days to Merck Global Safety. (Attn: Worldwide Product Safety; FAX 215 661-6229)

11.4 Reporting of Pregnancy and Lactation to UCLA DSMB and to Merck

If a participant inadvertently becomes pregnant while on treatment with pembrolizumab, the participant will be immediately discontinued from study treatment. Although pregnancy and infant exposure during breast feeding are not considered adverse events, it is the responsibility of investigators or their designees to report any pregnancy or lactation in a participant (spontaneously reported to them) that occurs during the study.

Pregnancies and infant exposures during breastfeeding that occur after the consent form is signed but before treatment must be reported by the investigator if they cause the participant to be excluded from the trial, or are the result of a protocol-specified intervention, including but not limited to washout or discontinuation of usual therapy, diet, placebo treatment or a procedure.

Pregnancies and infant exposures during breastfeeding that occur from the time of treatment through 120 days following cessation of Sponsor's product, or 30 days following cessation of treatment if the participant initiates new anticancer therapy, whichever is earlier, must be reported by the investigator. All reported pregnancies must be followed to the completion/termination of the pregnancy. Pregnancy outcomes of spontaneous abortion, missed abortion, benign hydatidiform mole, blighted ovum, fetal death, intrauterine death, miscarriage and stillbirth must be reported as serious events (Important Medical Events). If the pregnancy continues to term, the outcome (health of infant) must also be reported.

Such events must be reported within 24 hours to the UCLA DSMB and within 2 working days to Merck Global Safety. (Attn: Worldwide Product Safety; FAX 215-661-6229)

11.5 Immediate Reporting of Adverse Events to UCLA DSMB and Merck

11.5.1 Serious Adverse Events

A serious adverse event is any adverse event occurring at any dose or during any use of either AD-CCL21-DC or pembrolizumab that:

- Results in death;
- Is life threatening;
- Results in persistent or significant disability/incapacity;
- Results in or prolongs an existing inpatient hospitalization;
- Is a congenital anomaly/birth defect;
- Is a new cancer (that is not a condition of the study);
- Is associated with an overdose;
- Is any other important medical event

Refer to Table 7

for additional details regarding each of the above criteria.
Any serious adverse event, or follow up to a serious adverse event, including death due to any cause other than progression of the cancer under study that occurs to any subject from the time the consent is signed through 90 days following cessation of treatment, or the initiation of new anti-cancer therapy, whichever is earlier, whether or not related to AD-CCL21-DC, pembrolizumab, or both, must be reported within 24 hours to UCLA DSMB and within 2 working days to Merck Global Safety.

Additionally, any serious adverse event, considered by an investigator who is a qualified physician to be related to AD-CCL21-DC, pembrolizumab, or both that is brought to the attention of the investigator at any time outside of the time period specified in the previous paragraph also must be reported immediately to the UCLA DSMB.

11.5.2 Events of Clinical Interest

Selected non-serious and serious adverse events are also known as Events of Clinical Interest (ECI) and must be recorded as such on the Adverse Event case report forms/worksheets and reported to the UCLA DSMB. Events of clinical interest for this trial include:
1. An overdose of AD-CCL21-DC, pembrolizumab, or both, as defined in Section 14.2 - Definition of an Overdose for This Protocol and Reporting of Overdose, that is not associated with clinical symptoms or abnormal laboratory results.

2. An elevated AST or ALT lab value that is greater than or equal to 3X the upper limit of normal and an elevated total bilirubin lab value that is greater than or equal to 2X the upper limit of normal and, at the same time, an alkaline phosphatase lab value that is less than 2X the upper limit of normal, as determined by way of protocol-specified laboratory testing or unscheduled laboratory testing.*

*Note: These criteria are based upon available regulatory guidance documents. The purpose of the criteria is to specify a threshold of abnormal hepatic tests that may require an additional evaluation for an underlying etiology. The trial site guidance for assessment and follow up of these criteria can be found in the Investigator Trial File Binder (or equivalent).

1. Additional adverse events:

ECIs (both non-serious and serious adverse events) from the date of first dose through 90 days following cessation of treatment, or 30 days after the initiation of a new anticancer therapy, whichever is earlier, need to be reported within 24 hours to the UCLA DSMB and within 2 working days to Merck Global Safety, regardless of attribution to study treatment, consistent with standard SAE reporting guidelines.

Subjects should be assessed for possible ECIs prior to each dose. Lab results should be evaluated and subjects should be asked for signs and symptoms suggestive of an immune-related event. Subjects who develop an ECI thought to be immune-related should have additional testing to rule out other etiologic causes. If lab results or symptoms indicate a possible immune-related ECI, then additional testing should be performed to rule out other etiologic causes. If no other cause is found, then it is assumed to be immune-related.

11.5.3 Protocol-Specific Exceptions to Serious Adverse Event Reporting

There are no protocol-specific exceptions to serious adverse event reporting.

11.6 Evaluating Adverse Events

An investigator who is a qualified physician will evaluate all adverse events according to the NCI Common Terminology for Adverse Events (CTCAE), version 5.0. Any adverse event which changes CTCAE grade over the course of a given episode will have each change of grade recorded on the adverse event case report forms/worksheets.

All adverse events regardless of CTCAE grade must also be evaluated for seriousness.

11.7 Sponsor Responsibility for Reporting Adverse Events

All Adverse events will be reported to regulatory authorities, IRB/IECs, in accordance with all applicable global laws and regulations.

Table 7 Evaluating Adverse Events

An investigator who is a qualified physician, will evaluate all adverse events as to:

**V5.0 CTCAE Grading**	**Grade 1**	**Mild; asymptomatic or mid symptoms; clinical or diagnostic observations only; intervention not indicated.**
	**Grade 2**	**Moderate; minimal, local or noninvasive intervention indicated; limiting age-appropriate instrumental ADL.**
	**Grade 3**	**Severe or medically significant but not immediately life-threatening; hospitalization or prolongation or hospitalization indicated; disabling; limiting self-care ADL.**
	**Grade 4**	**Life threatening consequences; urgent intervention indicated.**

	**Grade 5**	**Death related to AE**
**Seriousness**	A serious adverse event is any adverse event occurring at any dose or during any use of AD-CCL21-DC, pembrolizumab, or both that:	
	†**Results in death**; or	
	†**Is life threatening; or** places the subject, in the view of the investigator, at immediate risk of death from the event as it occurred (Note: This does not include an adverse event that, had it occurred in a more severe form, might have caused death.); or	
	†**Results in a persistent or significant disability/incapacity** (substantial disruption of one's ability to conduct normal life functions); or	
	†**Results in or prolongs an existing inpatient hospitalization** (hospitalization is defined as an inpatient admission, regardless of length of stay, even if the hospitalization is a precautionary measure for continued observation. (Note: Hospitalization [including hospitalization for an elective procedure] for a preexisting condition which has not worsened does not constitute a serious adverse event.); or	
	†**Is a congenital anomaly/birth defect** (in offspring of subject taking either product regardless of time to diagnosis);or	
	**Is a** new **cancer;** (that is not a condition of the study) **or**	
	**Is an overdose** (whether accidental or intentional). Any adverse event associated with an overdose is considered a serious adverse event. An overdose that is not associated with an adverse event is considered a non-serious event of clinical interest and must be reported within 24 hours.	
	**Other important medical events** that may not result in death, not be life threatening, or not require hospitalization may be considered a serious adverse event when, based upon appropriate medical judgment, the event may jeopardize the subject and may require medical or surgical intervention to prevent one of the outcomes listed previously (designated above by a †).	
**Duration**	Record the start and stop dates of the adverse event. If less than 1 day, indicate the appropriate length of time and units	
**Action taken**	Did the adverse event cause AD-CCL21-DC, pembrolizumab, or both to be discontinued?	
**Relationship to test drug**	Did AD-CCL21-DC, pembrolizumab, or both cause the adverse event? The determination of the likelihood that AD-CCL21-DC, pembrolizumab, or both caused the adverse event will be provided by an investigator who is a qualified physician. The investigator's signed/dated initials on the source document or worksheet that supports the causality noted on the AE form, ensures that a medically qualified assessment of causality was done. This initialed document must be retained for the required regulatory time frame. The criteria below are intended as reference guidelines to assist the investigator in assessing the likelihood of a relationship between the test treatments and the adverse event based upon the available information.<br>**The following components are to be used to assess the relationship between AD-CCL21-DC, pembrolizumab, or both, and the AE**; the greater the correlation with the components and their respective elements (in number and/or intensity), the more likely the product in question caused the adverse event (AE):	
	**Exposure**	Is there evidence that the subject was actually exposed to AD-CCL21-

		DC, pembrolizumab, or both such as: reliable history, acceptable compliance assessment (pill count, diary, etc.), expected pharmacologic effect, or measurement of drug/metabolite in bodily specimen?
	**Time Course**	Did the AE follow in a reasonable temporal sequence from administration of AD-CCL221-DC, pembrolizumab, or both?<br>Is the time of onset of the AE compatible with a treatment-induced effect (applies to trials with investigational medicinal product)?
	**Likely Cause**	Is the AE not reasonably explained by another etiology such as underlying disease, other drug(s)/vaccine(s), or other host or environmental factors

11.8 Responsibility for Reporting Adverse Events

All Adverse Events will be reported to regulatory authorities, IRB/IECs and investigators in accordance with all applicable global laws and regulations.

12 DOCUMENTATION, RECORD KEEPING, CRFs

The investigator will maintain adequate records so that the conduct of the study can be fully documented and monitored.

Copies of protocols, case report forms (CRFs), test result originals, all product accountability records, correspondence, patient informed consent, and any other documents relevant to the conduct of the study will be kept on file by the investigator for five years after all investigational use of product is discontinued and the FDA is so notified or until five years after a Product Licensing Application is approved. Study documents will not be destroyed. For FDA inspections, it will be necessary to have access to complete study patient records, provided that patient confidentially is maintained.

The investigator will obtain a separate release of medical information form to be signed by the study patient in order to facilitate access to the patient's medical records should the patient be hospitalized at an institution with which the study investigator is not associated. A record will be kept of all patients considered (and screened) for the study and subsequently deemed ineligible. The reason for ineligibility must be recorded.

13 DATA COLLECTION AND STUDY MONITORING

13.1 <u>Introduction</u>

13.1.1 Data Coordinating Center

The UCLA Department of Medicine Statistics Core (DOMStat) is appointed as the DCC for "A phase 1 Trial of IT administration of Ad-C CL21-DC combined with IV pembrolizumab for advanced NSCLC." DCC will provide data and statistical support for data collection, entry, and management. These responsibilities further broken down include: overseeing the electronic data capture and monitoring systems, implementing staff data collection and entry training, participating in investigator meetings, preparing reports for the principal investigator and relevant parties, conducting quality controls, as well as performing data cleaning and analysis (particularly for primary endpoints). To conduct data quality checks and cleaning, DCC will deploy its sophisticated in-house query system and tracking system to identify and address data irregularities, thereby ensuring an accurate and complete electronic data capture database for final analysis.

13.1.1.1 Oversee the Data Collection and Monitoring Systems

Three tightly integrated systems make up the DCC data collection and management plan for this study: (1) the secure electronic data capture system, (2) the data management query system, (3) the tracking system. DCC will deploy these already developed data systems, monitor progress of data collection and transmission, as well as provide corrective feedback based on quality control checks. Amazon Web Services will be the encrypted cloud storage at the junction of these data systems. Please refer to Section 2 *Electronic Data Capture and Monitoring Systems* section for further explanation.

13.1.1.2 Participate in all investigator meetings

During these meetings, changes to the protocol and subsequent changes to the database will be discussed and new training will be provided if necessary. Study data collection progress reports will be provided by DCC to the rest of the team, and any data issues will be discussed and resolved. Additionally, group conversations led by DCC will develop solutions for systematic data acquisition and data entry problems which may be encountered at the collection site.

13.1.1.3 Prepare Reports

The DCC will generate reports periodically and by request throughout the study. Reports will cover information concerning participant accrual, primary endpoints evaluation, study compliance, data quality reports, review of out-of-range laboratory values, protocol deviation and violation reports, as well as AE/SAE summary and detail reports (especially with regards to DLT). DSBM reports will contain recruitment information, basic demographic information, and unblinded AE and SAE data.

13.1.1.4 Quality Control

The DCC will provide training to relevant personnel on data collection policies, proper data entry protocol and systems usage. Repeated data entry errors or deviations from data protocol will result in retraining overseen by DCC staff. Real-time logic checks coded into the EDC will minimize data entry errors and the need for corrective feedback. Scheduled checking of collected data will enable the DCC to provide corrective feedback to study staff, ensuring a complete and accurate database for subsequent analysis. Please refer to Section *5.2 Data Quality Control Procedures* for further explanation.

13.1.1.5 Data Analysis

Please see Section *14 Statistical Considerations* section in the Main Protocol.

13.1.2 Clinical Data Management Plan (CDMP) Overview

This CDMP makes clear to all participants the data management practices needed to ensure appropriate handling of data at all steps of the project. The principal investigator, the DCC, and project and study staff have all reviewed and signed off on the CDMP for this proposal. Further reviews will be initiated prior to and during the study to ensure familiarity and compliance. Under the direction of the DCC, any CDMP changes needed to meet to the evolving demands of the study will be reviewed and signed off by the appropriate parties.

13.2 Description of Electronic Data Capture (EDC), Monitoring, and Storage Systems

Three tightly integrated systems make up the DCC supervised data collection and management plan for this study: (1) the secure electronic data capture (EDC) system, (2) the data management query system (QS), and (3) the tracking system (TS). The EDC will enable

data capture, while the QS and TS will monitor the quality, completeness, and timeliness of study data. Amazon Web Services (AWS) will serve as the encrypted cloud storage at the junction of the EDC, QS, and TS data systems. Please refer to **Figure 6** below for an overview of the data flow.

Figure 6: Overview of Data Flow

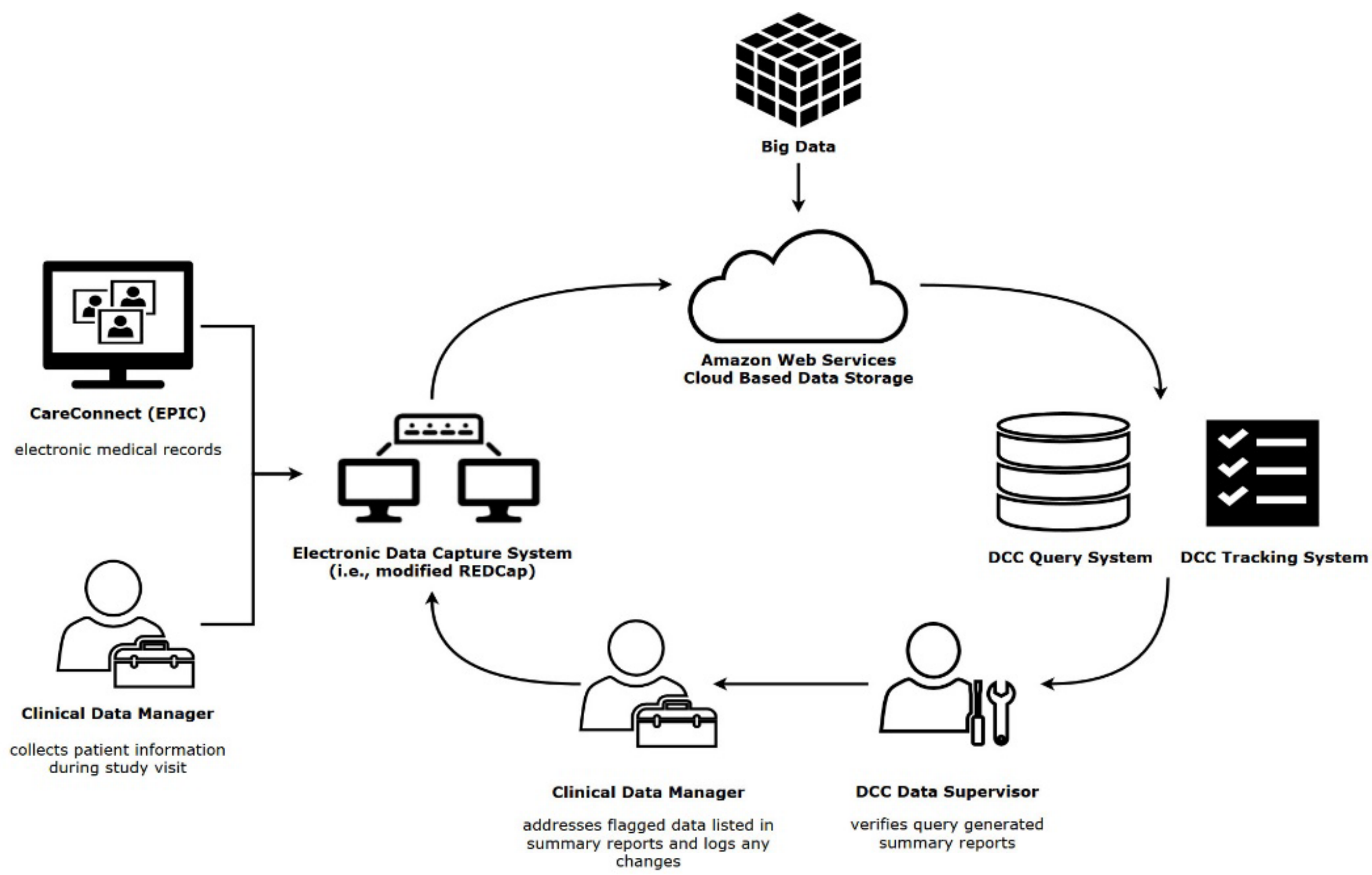

13.2.1 **Electronic Data Capture (EDC) Overview**

13.2.1.1 <u>Design and Protection</u>

The EDC used by the DCC is a modified version of Research Electronic Data Capture (**REDCap**) system [version 6.15.15]. REDCap is a secure, HIPAA compliant web application designed by the Vanderbilt University Medical Center to support data capture for biomedical research studies. With an intuitive user interface for database design and data entry, REDCap is flexible enough to be used for a variety of research models including surveys. Research teams can design surveys in a web browser and engage potential respondents using a variety of notification methods, including text messages and email. REDCap also provides real-time data entry validation (e.g. for data types and range checks), audit trails, a de-identified data export mechanism to common statistical packages (SPSS, SAS, Stata, R/S-Plus), and the ability to easily create real-time reports that preliminarily verify completeness and quality of data. Another important feature is the study-specific data dictionary generated in an iterative self-documenting process by all members of the research team. This iterative development and testing process promotes a well-planned data collection strategy for individual studies.

Based on extensive expertise and experience, DCC has modified REDCap to ensure that it performs at a higher and more secure level as required by clinical trials standards for EDC systems. These standards include Health Insurance Portability and Accountability Act (**HIPAA**) and the Good Clinical Data Management Practices (**GCDMP**) guidelines published by the Society for Clinical Data Management (**SCDM**). DCC modifications have also enabled automatic notification when AEs are marked as grade 3 or above, as well as reminder notifications for study staff of all upcoming treatment and visit dates after baseline.

REDCap electronic clinical research forms (**eCRFs**) (i.e., clinical research forms and surveys) have been personalized for the clinical trial's data collection needs. Sophisticated programming of the eCRFs by the DCC permits specific branching logic that enhances efficiency and accuracy of data entry. Please refer to Section 13.*6.3.2 Fields Revealed Based on Prior Selection* for further explanation of branching logic. Specified value ranges and character types for data entry fields, as well as automatic computation of derived variables and summary scores (e.g., age calculated from date of birth and date of baseline) will minimize data entry errors. Marking input for all fields as mandatory will assure a complete database. Please refer to Section 13.*6 Data Validation Processes for EDC Data* for further explanation of automated data validation checks and mandatory fields in the EDC.

The status of each eCRFs of the planned data collection will be viewed as an event grid with each time point displayed as a column and each eCRF displayed as a row, where completed, unverified, and incomplete form status are indicated respectively by the colors green, yellow, and red in the grid (Figure 7). This user friendly interface will enable the DCC appointed data manager to track the status of eCRFs and assure the completion of on-site participant data collection by study staff.

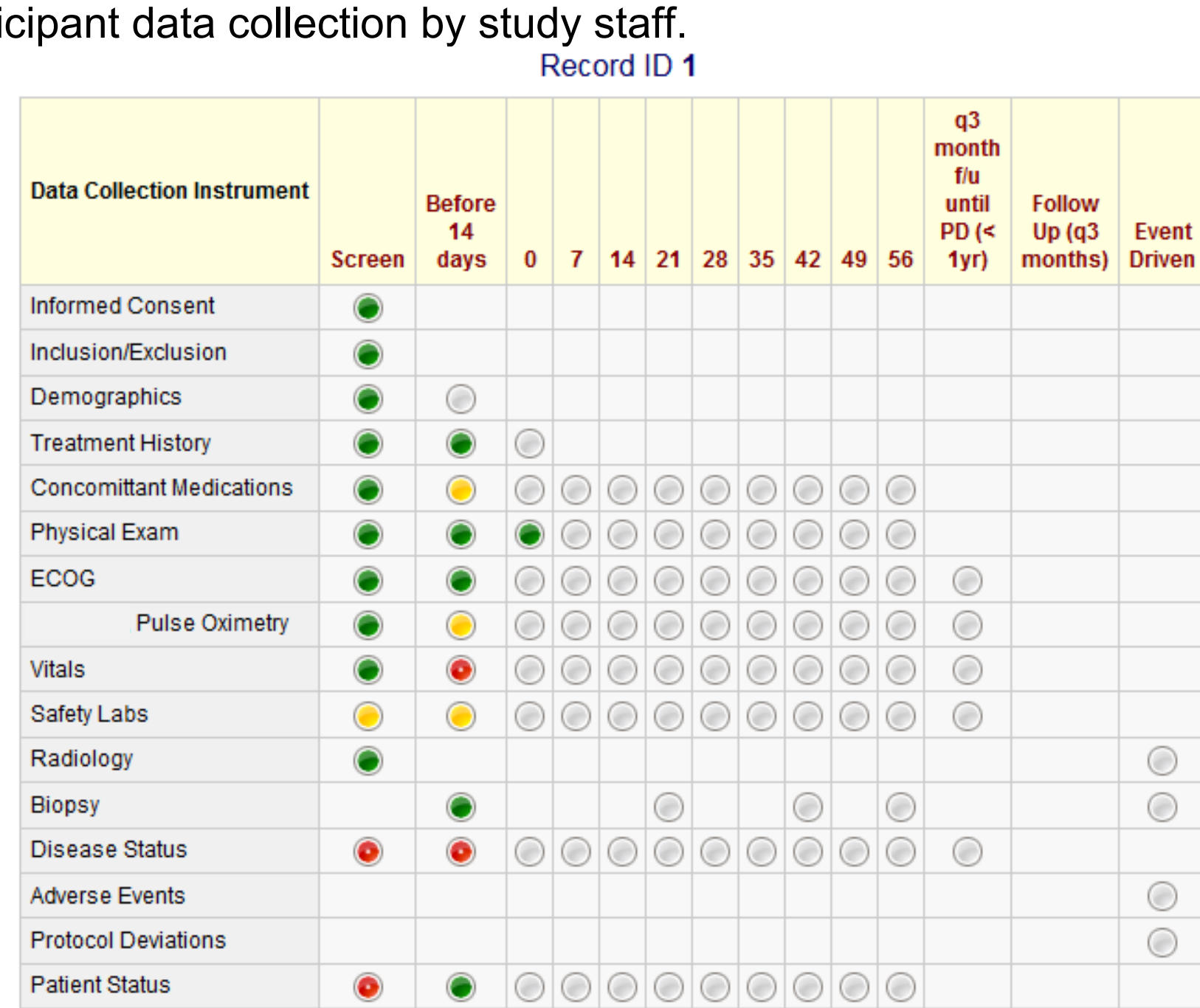

FIGURE 7: Sample REDCap eCRF status grid

Based on the study data collection needs, the following eCRFs (**Figure 8**) have been developed in REDCap to be administered during the study:

Figure 8: REDCap eCRF

	Description
Informed Consent	The informed consent document reviewed with and signed by participants will be uploaded to this REDCap eCRF. The original source documentation will be kept securely at the clinic site.
Inclusion/Exclusion	Inclusion and exclusion criteria will be administered to screen for trial eligibility.
Demographics	Age, gender, ethnicity, performance status, prior therapies, other baseline characteristics.
Treatment History	Any treatment(s) undergone by the participant specific to advanced NSCLC.
Concomitant Medications	Any medications taken by participants (before or during the trial) that are not administered as part of the protocol.
Physical Exam	Physical exam evaluating participant health before and during the treatment.
ECOG	Participant's ECOG performance results.
Vitals	Results for participant vitals (e.g., blood pressure).
Labs	Results for study labs.
Radiology	Radiology results, including imaging to assess tumor burden and response as well as CT or MRI of brain (if symptoms suggestive of CNS metastasis).
Biopsy	Results for biopsies.
Disease Status	Monitoring of cancer status (Stage I – IV).
Adverse Events	Information regarding serious and other adverse events.
Protocol Deviations	Information concerning protocol deviances will be documented.
Patient Status	Patient status throughout the study will be monitored. Status may be designated as "failed screening," "consented at screen," "active," "withdrawn," or "completed." Circumstances affecting participant involvement in the study will be documented.

eCRFs may be reviewed, eliminated, or added before initiation or during the study. Please refer to Section 13.10 Changes to Production EDC for further information.

13.2.1.2 <u>Security</u>
Please refer to Section 13.3.1 Network Directories for explanation of REDCap Security.

13.2.1.3 <u>Role of Study Staff</u>
DCC will supervise training for and deployment of the EDC (i.e., REDCap) at the clinical site. All study staff will be trained in proper methods of data collection during a group initiation meeting at the start of the trial. During the data collection period, the study staff will be required to enter the data into EDC during or soon after each participant's study visit. Any serious deviations from proper data collection and entry will require re-education of individual staff by the members of DCC.

Reports listing any missing or erroneously entered eCRF data will be sent to the clinic site on a weekly basis. The DCC will produce these reports using a query system developed to automate the process of identifying errors from erroneous data collection or data entry. Please refer to Section 13*.2.2 Query System* for further explanation. Study staff will correct identified data irregularities in the REDCap eCRFs and log their corrections. This correction log will help DCC data supervisor keep track of the progress and timeliness of error resolution in the study of REDCap data.

Study staff will have no role in managing CareConnect uploads into REDCap. Please refer to Section 13*.5.1.2 Care Connect* for further information regarding CareConnect uploads.

13.2.2 **Query System (QS) Monitoring System Overview**

13.2.2.1 <u>Design and Protection</u>

The Query System (**QS**) is intended to be used in conjunction with the DCC's modified REDCap (i.e., EDC). The QS serves as a comprehensive data quality check, essential for ensuring the integrity of future data analysis. The QS is developed as a standardized program that is flexible and has been tailored for the specifics of this particular clinical trial. Big Data collected in this study will be screened by the Tracking System, not by the query system.

Errors in the EDC data may arise due to manual entry and be left uncaptured by the EDC system's programmed automatic data validation checks. The QS enables a higher sophistication and complexity in programming checks that match the nature of the clinical trial's collected data. Hence the first job of the QS is to detect, locate, and label potential data anomalies such as missing information and values that are out of range or implausible. Inclusion and exclusion criteria will also be checked using the QS to serve as a secondary verification of participant's satisfaction of study criteria after the enrollment visit.

AWS permits the exporting of uploaded REDCap data in a variety of formats (e.g., SAS, R) that match the query system's formatting needs. The QS can also take the REDCap generated data dictionary and detect the branching logic for every eCRF, which is then used to identify the logic patterns governing the data. For example, if sex is selected as male, the QS is able to determine based on processing the data dictionary that questions concerning menopause are appropriately left blank (and if these questions are filled, QS flags those data entries). Based on logic analysis, the QS generates an output of flagged data entries. The QS then checks for any resolutions or changes made since the last query system analysis. If any flagged data entries have been modified, the QS is designed to track these changes, update the QS database of errors accordingly, and keep a historical log of all changes that occurred for any data entry field. The final step of the QS is to create overall reports about data errors and progress for DCC data supervisor. Data supervisor will verify these reports and forward them to site personnel if further follow-up for flagged entries is needed.

In addition to generating this database of errors, the QS also composes a list of variables that have been marked as having errors more than 33% of the time. This list functions as signal to the data manager that the self-generating logic should be revised. Please refer to Section 13.10 Changes to Production EDC for further explanation of how changes to the EDC (including logic manipulations) are implemented.

The extensive expertise and experience of the DCC from managing numerous complex data management projects needs reveals itself in this REDCap and QS set up. Each component is meant to complement and enhance the function of its counterpart for the end goal of rapid, accurate, and clear data processing. In summary, the QS is meant to be a periodic systematic check for errors in the site collected participant data. This error tracking software enhances communication regarding data cleaning between clinical study staffs and data supervisor, thereby assuring a complete and accurate database of participants' outcomes for final analyses. The associated corrections log is intended for the DCC appointed manager to easily view identified irregularities and monitor the type and timeliness of changes implemented.

13.2.2.2 Security

Please refer to Section 13.*3.1 Network Directories* for explanation of Query System Security.

13.2.2.3 Role of Study Staff

The DCC will be responsible for running and maintaining the QS. Study staff will have no interaction with the QS except for receiving and responding to the weekly quality reports.

13.2.3 **Big Data Tracking System**

13.2.3.1 Design and Protection

The DCC designed Tracking System will monitor the timeliness and completeness of processed Big Data uploads to the AWS Cloud. Before upload to AWS, Big Data will have already been processed by the Hoffman2 cluster platform. The DCC will alert study staff to any Big Data irregularities or incomplete uploads.

13.2.3.2 Security

Please refer to Section 13.*3.2 Network Directories* for explanation of Tracking System security.

13.2.3.3 Role of Study Staff

Study staff will have no interaction with Tracking System other than receiving reports listing Big Data uploads flagged for being incomplete or missing.

13.2.4 **Amazon Web Services Storage Cloud**

13.2.4.1 Design and Protection

*Amazon Web Services (**AWS**)* offers a reliable, scalable, and inexpensive cloud computing service with a pay for what you use model. AWS is trusted by a variety of enterprises to power a range of workloads including: web and mobile applications, game development, data processing and warehousing, storage, archive, and many others. Rich controls, auditing, and broad security accreditations ensures that AWS meets local and global laws and regulations concerning participant privacy, including HIPAA. For this study, Hoffman2 cluster platform processed Big Data and REDCap data will be systematically backed up into the AWS cloud. This backup serves as an additional guarantee for data integrity, as well as provides the DCC another secure means by which to access participant data for quality controls and analysis. Clinical and lab data will be merged within the cloud for analysis. The DCC is working closely with others within and outside the UCLA community to implement AWS services for numerous studies. This is being done in order to better align present and future research with NIH initiatives for

cloud-based research projects, an initiative developed in response to the challenges of increasingly complex data (i.e., Big Data).

13.2.4.2 Security
Please refer to Section 13.*3.3 Network Directories* for explanation of AWS Security.

13.2.4.3 Role of Study Staff
Study staff will have no interaction with AWS. The DCC will work in conjunction with the UCLA Research Computing Services Team (https://dgit.healthsciences.ucla.edu/pages/Research-Computing) to maintain the AWS cloud based server.

13.3 Security of Electronic Data Capture (EDC), Monitoring, and Storage Systems

13.3.1 EDC (i.e., REDCap) and Query System (QS)

The EDC and QS are both supported by the UCLA Clinical and Translational Science Institute (**CTSI**) program (https://www.ctsi.ucla.edu/) and include high level data security, access logs, data storage, and onsite and offsite backup. For this study, data shared between the EDC and QS will be stored on UCLA's secure network drive and backed up on the AWS cloud based server maintained by the Research Computing Services Team (https://dgit.healthsciences.ucla.edu/pages/Research-Computing). UCLA CTSI supports the local data center that hosts the EDC servers, as well as backs up the host UCLA network drive nightly. All web-based REDCap information transmission is encrypted with TLS (Transport Layer Security) which provides improved security over SSL (Secured Socket Layer). As a further precaution, the DCC will regularly download and store all data on external hard drives that will be locked in the office of the principal biostatistician.

All staff, including data managers and investigators, must request a REDCap account and be granted project-specific access by the data supervisor in order access any database. Assigning each study staff their own REDCap account enables computer-generated, time-stamped audit trails that independently record the date and time of operator entries and actions that create, modify, or delete electronic records. Data access rights will be tailored for each person's role on the project to limit data editing and exporting. REDCap permits two forms of access for data entry: the public facing Internet REDCap allows the collection of limited data set while the internal facing Intranet REDCap allows the collection of PHI data. The Intranet REDCap is only accessible within the Mednet network or through VPN access from the outside. This study will use the Intranet REDCap. Therefore, access to this study's Intranet REDCap database is limited to people with a REDCap account who are approved by the investigator and using computers within the Mednet network or through VPN access.

13.3.2 Tracking System (TS)

The TS will be protected by UCLA's protected network and the DCC's sever protections. Any Big Data screened by the TS will be securely backed up on the Amazon Web Services Cloud. Only approved members of the DCC will be able to access the TS using their assigned UCLA VPN protected accounts, which enables audit trails.

13.3.3 Amazon Web Services

Key features of the encrypted, HIPAA-accommodating Amazon Web Services cloud server include (1) a secured computer space that limits access to the associated virtual machines (VMs); (2) a 24/7/365 uptime for data collection and access to the web portal,

with means for system redundancy and backups; (3) a scalable amount of storage for the anticipated mHealth datasets; and 4) an on-demand capacity for computational power (via the Elastic Compute Cloud, EC2). Security will be further bolstered by UCLA institutional security measures (e.g., VM access is limited to UCLA's authentication methods) and access restriction to on-campus AWS-trained software engineers.

13.4 Verification of Setup and Implementation of Electronic Data Capture (EDC), Monitoring, and Storage Systems

13.4.1 **Electronic Data Capture (e.g., REDCap)**

13.4.1.1 Site Data

The EDC database and eCRFs for this study have already been developed and tested with sample data according to established DCC protocol. The protocol reflects years of accumulated expertise and experience from numerous clinical studies. Study systems functioning will again be evaluated and documented prior to participant enrollment by the DCC director (Dr. Elashoff). Please refer to Section 13.6 Data Validation Process for EDC Data for further explanation of the data validation plan for site-collected participant data. Please refer to Section 13.2 Electronic Data Capture (EDC) System and the Underlying Clinical Database for further explanation of the study systems' specifics.

13.4.1.2 CareConnect Data

CareConnect data will be propagated into the REDCap eCRFs using an automatic query program deployed in previous DCC moderated clinical trials. The CareConnect to REDCap pipeline will be tested prior to study start using sample data in a joint effort by the DCC and UCLA's CTSI Biomedical Informatics Group. Please refer to Section 13*.5.1.2 Care Connect* for further information about this automatic CareConnect-to-REDCap query program.

13.4.2 **Query System and Tracking System**

The QS functioning has been tested in other studies and adjusted accordingly to meet the complexity and data burden of clinical trials. The TS was specifically created for this study, and will therefore undergo rigorous function testing based on DCC protocol for deploying new data validation systems. The QS and TS will both be tested using sample data prior to study start. Please refer to Section 13.2 Electronic Data Capture (EDC) System and the Underlying Clinical Database for further explanation of these study systems' specifics.

13.4.3 **Amazon Web Services**

The UCLA Research Computing Services Team (https://dgit.healthsciences.ucla.edu/pages/Research-Computing) will be responsible for implementing and maintaining the AWS cloud. Prior to the study, DCC will confirm sample data uploads into the AWS to ensure that data flow (diagramed in **Figure 6**) is working appropriately. During the study, DCC will verify study data uploads into the AWS cloud using the QS and TS.

13.5 Data Entry, Downloads, and Cleaning

13.5.1 **Data Input into the EDC**

13.5.1.1 Study Staff Data Entry

Study staff will manually enter all gathered clinical information into eCRFs (e.g., demographics, inclusion/exclusion criteria satisfaction). The DCC will check data entry for plausibility and consistency using the query system. Any data that is flagged by the query system will be reviewed by the DCC appointed data manager and (if appropriate)

corrected by study staff. The EDC will serve as the initial data record for data except for consent forms, data automatically pulled from CareConnect, and Big Data.

13.5.1.1.1 Pre-requisites for Study Staff Data Entry into EDC

The DCC will supervise deployment of and training for the EDC (i.e., REDCap) at the clinical site. All study staff will be trained in proper methods of data collection during a group initiation meeting at the start of the trial. Prior to the data collection period, the study staff must enter provided test data into the eCRFs. These data entries must be reviewed and approved for accuracy by the DCC data supervisor in order to certify the study staff in data entry for the trial.

13.5.1.1.2 Study Staff access to EDC

The DCC data supervisor of the EDC can grant data entry privileges to the study staff once the training meeting is completed, competence in data entry is demonstrated (refer to 6.1), and compliance with the privacy training mandated by relevant regulatory boards of the specific clinic and UCLA is documented (e.g., HIPAA). These data access privileges are tailored for each person's role on data editing and exporting. For study staff, access will generally be restricted to data entry into eCRFS and, should the need for corrections arise, data editing. Other functions will be granted only to qualified personnel who need such access.

13.5.1.1.3 Data Entry Guidelines

The DCC has developed a manual of operations describing all necessary study procedures which need to be entered onto the EDC system (i.e., REDCap). The manual outlines how to enter data into and navigate the eCRFs using step by step diagram based instructions. The DCC will distribute physical and digital copies to the study staff.

13.5.1.2 <u>CareConnect</u>

The following information will be pulled from CareConnect and uploaded into REDCap: labs, adverse events, procedures, diagnoses, concomitant medications, vitals, radiology tests reports, pathology reports. A mix of standardized lists and free text fields will be used to collect participant information in CareConnect. Using standardized list permits rapid analysis and side steps the statistical analysis problem of linking varied descriptions or spellings for the same lab or medication. Standardized lists will be developed with the expertise of the principal investigator and adjusted as necessary based on the needs of the trial.

The UCLA's CTSI Biomedical Informatics Group has developed a query program that can automatically propagate REDCap eCRFs with data pulled from participant medical records within CareConnect. This query program has been used in previous clinical trials, but has been reworked for the unique needs of this clinical trial. Please refer Section 13.4.1.2 CareConnect Data for further information about query program function testing.

13.5.2 **Data Quality Control Procedures**

The quality of study data will be monitored using multiple query and monitoring systems. Generally, based on query and monitoring system results, reports will be generated and sent to study staff to initiate the approval or corrections process. Please refer to Section 13.*8 Reports* for further information about reports.

13.5.2.1 EDC

During data collection period, the study staff will be required to enter the data into EDC during the participant's study visit or shortly after the visit. Built-in automatic checks of the EDC system ensure manually entered data follows the logic parameters required for responses to the questions. The EDC permits the programming of popup windows (i.e., queries) to automatically appear on a conditional basis. Study staffs inputting information into the EDC eCRFs are alerted with popup windows if their data entry inputs are implausible or illogical. Please refer to Section 13.6 Data Validation Process for EDC Data for the triggering criteria.

The kinds of correction alerts that will appear are as follows:

- If mandatory data fields are left blank, then study staffs will be informed via popup that they are not allowed to close out or switch between eCRFs before filling out the required field. This correction alert will continue to appear until the mandatory data fields are completed.

- For both mandatory and non-mandatory fields, if the data entry input fails the parameters established for the question, either the study staff will be prompted to change their response or to confirm their response. The correction alert will continue to appear until the parameters for the question are met or the entry has been confirmed.

When necessary, the study staffs will be able to contact the DCC to troubleshoot any data entry issues. Members of the DCC will assist in helping to resolve the issue. All overrides of correction alerts that permit data entries that fail study parameters will be logged by the DCC data manager into the corrections log. No overrides are anticipated.

The data entered into the EDC will be further filtered for errors with the QS. This QS has been developed by the DCC to automate identification of logic and consistency errors, such as the wrong number of entries present for a given data domain. Please refer to Section 13.4 Data Validation Processes for further explanation of automatic EDC and QS data checks. Any serious deviations from proper data collection and entry identified via the QS will require re-education of individual staff by the members of DCC.

13.5.2.1.1 EDC Laboratory Values

REDCap eCRFs will be programmed with range restrictions that are permissive enough to allow unusual lab results, yet still filter out implausible values. Range boundaries will be defined using the expertise of the principal investigator as well as UCLA's database of standardized lab results. A weekly report of out-of-range or unusual lab values will be sent to the clinical core for review and approval. Please refer to Section 13.8 Reports for further information about the report.

13.5.2.2 Big Data

Big Data will be checked using the DCC developed TS for completeness and timeliness of uploads. Please refer to Section 13.*2.3 Big Data Tracking System* for further information regarding the tracking system. A weekly report will be sent to the study staff for review and corrections. Please refer to Section 13.*8 Reports* for further information about this report.

13.6 Data Validation Processes for EDC Data

Sophisticated programming of the EDC (i.e., REDCap) and QS by the DCC programming staff automates data validation at the data entry and data processing stage. The eCRFs used in REDCap permits branching logic and skip patterns that enhance efficiency and accuracy of data entry. Univariate, multivariate, cross-module, and cross-visits alerts in the EDC will minimize data entry errors at the site, while in the QS these alerts will enable the DCC to create automated weekly corrective feedback reports for site coordinators.

In combination, the validation protocols of EDC and QS will assure a complete and accurate database for analysis by the DCC. Please refer to Section 13.2 Electronic Data Capture and Monitoring Systems for further explanation of integration between these systems. The following subsections detail the automatic validation programing used for EDC or QS.

13.6.1 **Univariate Alerts**

Automated univariate alerts in the EDC and QS serve as part of the first line of defense against data entry and processing errors in site collected data. These alerts enable rapid identification and correction of inconsistencies, thereby assuring the accuracy and completeness of the database for analysis.

13.6.1.1 Valid Character Type

Data entry fields are programmed to only accept inputs that match the required character type (e.g., questions seeking numerical responses will only allow numerical inputs). If the wrong type of character is inputted into the EDC, a pop up window will indicate to the study staff the correct character type. Should the QS identify data entries that do not match the expected character type, the DCC appointed data manager will be alerted by the system automatically and will be able to initiate a documented correction process.

13.6.1.2 Valid Range

Pre-set ranges informed by previous research and clinical standards will minimize errors in data entry (e.g., 400 BMI). The ranges are permissive enough to allow reasonable but rare values, yet restrictive enough to filter out implausible measures generated by mistypes. Previous research and clinical standards have been selected via the expertise of the principal *investigator*. In the EDC system, a pop up window will serve as an alert for an out of range value and contain the information for the DCC data manager should the study staff need to seek permission to input unusual values. The QS serves as a secondary and more complex filter, allowing for categorization of data based on more nuanced criteria. The DCC data manager will review the categorized data and, based on study specific research and clinical standards, initiate the documented correction process as needed. Any unusual values will be noted and stored with explanations derived from participant information or study staff, thereby assuring the integrity of future DCC database analysis.

13.6.1.3 Missing Value

If data entry fields within the EDC are not set as mandatory, study staffs may skip those questions. The QS processes the data collected by the EDC and generates a missing values list. The DDC appointed data manager will review this list and follow up with the study staff if any missing values seems to have occurred in error. These missing values will be documented together with the reason for its absence.

13.6.1.4 Mandatory Fields

The EDC system can be set to designate specific data entry fields as mandatory, ensuring that study staffs may not close out or switch between eCRFs before filling out required information. A pop up window within the EDC system will alert the study staff to which data entry fields need to be completed. The QS then works in conjunction with the EDC to filter this mandatory data for plausibility (e.g. valid range, consistency across eCRFs and visits). The DCC appointed data manager will review a list created by the QS of flagged values, follow up with the study staff, and document any corrections to the data.

13.6.1.5 Automatic Computation of Summary Scores and Other Derived Variables

The EDC is capable of automatically calculating summary scores and other derived variables (e.g., Age calculated from date of birth and date of baseline). This automation minimizes chances for errors in calculations or data entry to occur on the side of the study staff. The QS assesses the plausibility of these summary scores and other derived variables, but does not participate in the computation.

13.6.2 Valid Value Alerts for Multiple-Choice Fields (Select only 1)

The variety of multiple-choice field types in the EDC enable the DCC to tailor the eCRFs to the needs of the study.

13.6.2.1 "Select One" Checklist

The eCRFs of the EDC incorporate data entry fields requiring the selection of one checkbox from a series of different choices. These specific "check one" checklist entry fields only permit the selection of one response, meaning two responses cannot be selected for the same question. If the option "Other" is selected, another data entry field can be coded to appear and collect an explanation (e.g., "Please explain other"). Other data entry fields can be coded to conditionally appear based on the response given to these "Select One" checklist questions. Please refer to Section 13.6.1.4 Mandatory Fields for when the question is designated as mandatory. Please refer to Section 13.6.1.3 Missing Value for when the question is designated as non-mandatory and left blank.

13.6.2.2 "Select Multiple" Checklist

The EDC contains a data entry field option allowing the selection of one or more checkbox from a series of different choices. If other choices are selected alongside the option "None," a pop up window appears alerting the study staff to their inconsistency error. Should this error remain ignored, the QS is programmed to flag such incongruences, enabling the DCC appointed data manager to initiate the documented corrective process. If the option "Other" is selected, another data entry field can be coded to appear and collect an explanation (e.g., "Please explain other"). Other data entry fields can be coded to conditionally appear based on the response given to these "Select Multiple" checklist questions. Please refer to Section 13.6.1.4 Mandatory Fields for when the question is designated as mandatory. Please refer to Section 13.6.1.3 Missing Value for when the question is designated as non-mandatory and left blank.

13.6.2.3 Yes or No

The EDC allows for the creation of yes or no data entry fields. Other data entry fields can be coded to conditionally appear based on the response given to these yes or no questions. Please refer to Section 13.6.1.4 Mandatory Fields for when the question is designated as mandatory. Please refer to Section 13.6.1.3 Missing Value for when the question is designated as non-mandatory and left blank.

13.6.2.4 Drop Down

Questions in the EDC may be programmed with pre-set answers that can be selected from a drop down menu. Drop down questions only permit the selection of one response, meaning two responses cannot be selected for the same question. If the option "Other" is selected, another data entry field can be coded to appear and collect an explanation (e.g., "Please explain other"). Other data entry fields can be coded to conditionally appear based on the response given to these drop down questions. Please refer to Section 13.6.1.4 Mandatory Fields for when the question is designated as mandatory. Please refer to Section 13.6.1.3 Missing Value for when the question is designated as non-mandatory and left blank.

13.6.3 **Multivariate and Cross-Module Alerts**

Multivariate alerts are programmed to identify incongruent responses within one eCRF or between eCRFs during the same assessment time (e.g., Screen) or between two different assessment times (e.g., between Screen and Baseline). Multivariate alerts identify logical inconsistencies within eCRFS from the same assessment time. Cross-module alerts identify data mismatches between eCRFS from different assessment times. For example, for multivariate consistency, if an eCRF collecting information about birth control indicates that a female participant is using birth control, then any positive responses to male birth control use (e.g., vasectomy) will be flagged. For cross-module consistency, if the participant is designated as male during Screen, any responses to questions pertaining to only female subjects will be flagged across all subsequent visits. The EDC permit branching logic and skip patterns within the eCRFs that minimize the potential for both multivariate and cross-module data errors.

13.6.3.1 Related Fields serving as Reference Values

Within and between eCRFs in the EDC, specific data entry fields can be designated to serve as reference for judging the validity of other data entry fields. The QS alerts the DCC appointed data manager to data entries that violate the parameters established by the reference value. The QS also automates identification of data entries that violate pre-established patterns. For example, if a participant's BMI fluctuates between visits Baseline to next visit from 21 to 27, the QS can extract this data entry for further evaluation. The data manager is likely to find the source of the mismatch and a corrective process can be initiated and corrections logged.

13.6.3.2 Fields Revealed Based on Prior Selection

The EDC permits conditional linkage between data entry fields, meaning the answer to a prior question can determine the subsequent questions revealed on the eCRF. This programmed conditional reveal enhances the accuracy of data collection by study staff and can be done between or within eCRFS as well as during the same or between visits. For example, if a participant is indicated to be male, then questions regarding pregnancy and lactation will remain hidden during the visit and during subsequent visits. Should the participant be identified as female, questions regarding pregnancy and lactation will appear at the baseline visit.

13.7 Electronic Files

13.7.1 **Electronic Data Capture System**

13.7.1.1 Consent Documents

The EDC system will not serve as source documentation for consent documents. Signed consent forms will be uploaded as pdfs to the EDC (i.e., REDCap) by study staff after every participant screening visit. The physical copy of the signed consent forms will remain on-site as source documentation.

13.7.1.2 CareConnect Medical Files
CareConnect medical records will be scanned using a query program created by UCLA's CTSI Biomedical Informatics Group. Participant data will be pulled from Care Connect by this query program and automatically transferred into REDCap eCRFs.

13.7.2 **Big Data**
Big Data files will be processed using the Hoffman2 cluster platform and then uploaded into the AWS cloud.

13.8 Reports
The following reports will be prepared by DCC throughout the study data collection period.

List of Data Management Reports

**Report Name**	**Description/Purpose**	**Frequency**	**Recipients/ Users**	**Method for Provision**
Study Accrual Report	Reports up to date summary of newly enrolled, completed, and lost to follow up participants	Weekly	DCC Data Managers and executive committee	Word document
Screen Fail Report	Report detailing reasons why participants failed screening.	Periodically throughout study enrollment.	DCC, Principal Investigator	PDF
Progress Report	Reports on up to date enrollment as well as baseline demographics and characteristics. Also include reasons for screen fail and screen drop out.	Semi-Annual	DCC Data Managers, Executive Committee, and NIDCR	PDF
Query System Flagged EDC Data Entries	List all data entries that have been flagged for logic or value inconsistencies.	Updated weekly, starting at study commencement	DCC Data Managers and Study staffs	Automated report stored on local network.
Out of Range Lab Values	Reports listing out of range lab values for review by principal investigator.	Weekly	DCC, Principal Investigator	Automated report stored on local network.
Tracking System Reports	Report identifying Big Data uploads to AWS that are incomplete or not on time.	Weekly	DCC, Study Staff, Principal Investigator	Automated report stored on local network.
Correction Log for EDC	List all data entries that have been flagged in REDCap and any follow up that occurred (left alone or modified from initial input).	Updated continuously, starting at study commencement.	DCC Data Managers, Study Staff	Database on the EDC (i.e., REDCap), propagated with eCRF

	Includes reference to all personnel involved and at what stages involvement occurred.			surveys collecting information about errors
Protocol Deviations and Violations	Report detailing protocol deviations and violations.	Periodically throughout study.	DCC, Principal Investigator	PDF
AE/SAEs	Report listing AEs/SAEs that are marked as grade 3	According to safety and clinical standards	DCC, Principal Investigator, Relevant Parties (i.e., DSMB, IRB)	PDF
DSMB	Report containing recruitment and basic demographic information, as well as unblended AE and SAE data.	According to previously established DSBM timeline and by request	DCC, Principal Investigator, DSMB	PDF
IRB enrollment Report	General enrollment demographics	Annual	DCC Data Managers, Executive Committee and IRB	Electronic transmission to IRB portal
Study Analysis	Reports analyzing collected data (e.g., participant characteristics, outcomes, etc.).	At End	DCC, Principal Investigator	Word Document

13.9 S/AE Reconciliation

The DCC has developed an eCRF to record the event of serious and other adverse events. Events that are unexpected, related or possibly related to the study, and place subjects at greater risk than originally anticipated will be documented on an AE/SAE REDCap eCRF. To satisfy the requirement for prompt reporting, related and unexpected adverse events will be reported within ten days and serious adverse events within two. All adverse events will be reported to regulatory authorities (including CIRM and the Jonsson Comprehensive Cancer Center's DSMB), IRB/IECs, and investigators in accordance with all applicable global laws and regulations. The Office for Human Research Protections (**OHRP**) recommends that investigators include the following information when reporting an adverse event, or any other incident, experience, or outcome:

- appropriate identifying information for the research protocol, such as the title, investigator's name, and the IRB project number;
- a detailed description of the AE, incident, experience, or outcome;
- an explanation of the basis for determining that the AE, incident, experience, or outcome represents an unanticipated problem;
- a description of any changes to the protocol or other corrective actions that have been taken or are proposed in response to the unanticipated problem.

AEs and SAEs will be categorized based on their relationship to study drug, grade, severity, and expectedness. AE eCRFs will be programmed to evaluate protocol defined drug limiting toxicity (**DLT**). REDCap automatically generates summary AE reports by AE type and by participant ID, enabling immediate AE/SAE review at all study time points. The DCC will conduct more sophisticated analysis on SAE and AE throughout and at the end of the study period in compliance with the demands of the study. The DCC will also generate a cumulative AE report for quarterly, biannual, or annual review (as determined necessary by the DSBM).

13.10 Changes to Production EDC

Should the DCC data supervisor be alerted to the fact that changes are needed to the EDC system (i.e., REDCap) while the project is in production, the data supervisor will initiate a meeting with the director of the DCC (i.e., David Elashoff). Dr. Elashoff will review proposed changes, provide feedback, communicate with the principal investigator, and sign off as needed. The data supervisor will then back up the project specific EDC database, implement the changes to the project, and submit the changes for approval by the DCC's EDC systems manager. The systems manager then reviews these changes to ensure no prior data collection will be compromised by implemented changes. If necessary, the data supervisor will have the ability to restore the project specific EDC database from the DCC backup data stored on the server and external hard drives. The details of the change to the project will be automatically logged by the EDC system. The DCC will initiate additional training for study staffs if required by the changes to the EDC system.

13.11 Database Closure

After study data collection end, the final data validation using the query system (for REDCap) is run. If no discrepancies in the data are identified, the datasets are finalized in consultation between the data manager and the principal statistician. All data management activities will be completed prior to database lock. Completion of pre-lock data management activities will be monitored, approved, and documented by the principal statistician. Careful execution of pre-lock activities is essential, as the database cannot be changed after locking except for in case of a critical issue or for other important operational reasons. Please refer to Section 13.11.3 Database Unlock for further explanation of criteria for database unlocking. Once the approval for locking is obtained from all relevant parties, the database is locked and clean data is extracted for statistical analysis by the DCC. Data analysis will be followed by data archival according to best-practice standards. Please refer to Section 13.12 Data Archiving and Provision of Final Materials to Sponsors for further information about archival of study data.

Subsections here include an overview of final pre-closure data checks; quality assurance review and actual database lock; and procedures for unlocking a database in the event that data must be corrected after lock.

13.11.1 Closure Checks

Closure procedures prior to database lock will verify the integrity and completion of the database. In some cases, these may be the same data checks described in the above sections, repeated until all queries are resolved and the data is determined to be clean. Procedures also include:

- Check that all expected eCRFs have been entered in REDCap
- Determine the status of each subject entered (i.e., excluded, ongoing, completed, withdrawn, lost to follow-up, etc.)
- Check for value formatting problems in database exports
- Confirm that all expected site signatures have been applied

- Review and confirm explanations for irregularities in data with study staffs and study managers (e.g., participant goes from 21 to 27 for BMI between first and second visit) during the period between enrollment visit and randomization visit)
- Final approval of the principal biostatistician and principal investigator

13.11.2 Quality Assurance Audit and Database Lock

Quality of collected data will be assured through the built-in EDC data validity checks, the Query system, REDCap errors database record data corrections and explanations. Built-in EDC validity checks will serve as the first line of defense for data errors. The Query System will generate summary reports identifying data flagged for violating study parameters for REDCap. The DCC will then contact the study staffs to initiate corrections or collect explanations for any irregularities in REDCap data. All corrections and explanations for REDCap data will be stored on a separate EDC errors database.
After the final data cleaning using the query system, the DCC will perform an audit on data exported from REDCap. For example, a sample of completed eCRFs in REDCap may be compared against exported SAS databases to ensure the integrity of the final study data. Upon the study completion and data cleaning, a soft lock will occur to freeze out all data collection personnel accounts (study staffs and study managers). After data analysis is completed, the hard lock will be initiated and all REDCap accounts other than those belonging to the DCC appointed EDC manager and principal biostatistician will have their access privileges revoked and accounts closed. Databases will be archived and shared according to relevant standards. Please refer to Section 13.12 Data Archiving and Provision of Final Materials to Sponsor for further explanation of the archiving process.
Any modifications to datasets after the implementation of the soft lock will require approval by the principal investigator, biostatistician, and EDC manager. Necessary changes to databases after the hard lock will require a more extensive review, sufficient justification, and accompanying audit trail. Please refer to Section 13.11.3 Database Unlock for further explanations of modifications to databases after hard lock.

13.11.3 Database Unlock

Generally, no modification in the database is possible after database lock. In case of a critical issue or for other important operational reasons, privileged users (i.e., principal biostatistician) can modify the data even after the database is locked. Critical issues and operational reasons will be jointly assessed by the principal investigator and biostatistician through the lens of their considerable background in clinical trials. An audit trail and proper documentation detailing sufficient justification for updating the locked database will be maintained. Any changes to the database will require further approval by the EDC manager to ensure the integrity of other pre-existing data remains intact. Before changes are implemented, the databases are downloaded to ensure an unmodified copy of the database exists should the need arise for the database to be restored.

13.12 Data Archiving and Provision of Final Materials to Sponsor

Principal Investigators and Grantees will share intellectual property generated by this CIRM-funded research including research results in scientific articles, publication-related biomedical materials, and patented inventions for research use in California as required by CIRM regulations duly adopted by the ICOC. The entire EDC database of the study will remain stored in the cloud and also on site with the DCC in an external hard drive for 5 years.

13.13 Jonsson Comprehensive Cancer Center Data and Safety Monitoring Plan

<u>JCCC DSMB</u>:

The Jonsson Comprehensive Cancer Center (JCCC) Data and Safety Monitoring Board (DSMB) was constituted in January 2001. The DSMB membership consists of representatives of the Cancer Center clinical research community. The Director of the Cancer Center appoints members to a term of 2 years and each member is selected for their professional expertise in oncology practice and research.

The JCCC DSMB meets monthly to review all SAE reports for trials overseen by the JCCC DSMB. All SAE reports, which have been filed since the previous meeting, are presented to the committee for review.

For trials overseen by the JCCC DSMB, the DSMB reviews all dose-limiting toxicities (DLTs) for dose-escalation studies. Protocol suspensions and re-opening of accrual to the next cohort based on DLT evaluation fall under the purview of the DSMB.

For all JCCC oncology trials where the JCCC DSMB has primary oversight, all SAEs shall be reported to the JCCC DSMB in a timely manner [ten days, two days for a death] regardless of relationship and expectedness. The JCCC ORC will review all submissions and the ORC staff will enter the information into the JCCC Clinical Trials database. Reports are generated for full JCCC DSMB review. For trials where the JCCC DSMB has primary DSMB review responsibility, the DSMB requires that the PI generate cumulative adverse event reports for quarterly, biannual or annual review.

The DSMB reviews each SAE report and determines whether or not protocol modifications are warranted to ensure subject safety. In this review, prior occurrences of similar toxicity with the therapy under study are taken into consideration, as well as the severity of the event and the likelihood that it was related to a study drug. The DSMB may recommend no changes to the study if the event is expected or related to other causes such as the subject's underlying condition. The DSMB may request an expert's advice of another non-Principal Investigator with national experience to support their deliberations and decisions.

The JCCC DSMB has the authority to recommend to the UCLA IRB the immediate halt to a study (i.e., discontinuation of any further treatment of enrolled subjects and discontinuation of enrollment of new subjects) should there be any serious unexpected toxicity that warrants further investigation.

14 STATISTICAL CONSIDERATIONS

14.1 <u>Study Design and Objective(s)</u>

This is a Phase I dose escalation trial to evaluate the safety and efficacy of intratumoral injection of CCL21 gene-modified DC (Ad-CCL21-DC) when combined with intravenous pembrolizumab in 1) patients with advanced non-small cell lung cancer (NSCLC) without a sensitizing epidermal growth factor receptor (EGFR) mutation and/or anaplastic lymphoma kinase (ALK) gene rearrangement who have received prior systemic anti-cancer therapy for advanced disease that includes a PD -1 and/or PD-L1 inhibitor or 2) who have sensitizing EGFR mutations and/or ALK gene rearrangements and have received prior tyrosine kinase inhibitor therapy.

During dose escalation, a modified 3+3 design will be used in the first dose escalation cohort

and six patients will be enrolled in the second dose escalation cohort. Patients enrolled into a given cohort ((1), (2), (-1)) will receive the same Ad-CCL21-DC dose by CT-guided or bronchoscopic intratumoral injection preceded by intravenous pembrolizumab 200 mg on days 0, 21, and 42 (each +/- 7d), and intravenous pembrolizumab 200 mg every three weeks thereafter for up to a year. The Ad-CCL21-DC dose is 1 x 10^7 cells/injection in the first cohort (1), and will be increased to 3 x 10^7 cells/injection (2) pending tolerability in the earlier cohort. Dose escalation may proceed only if all 3 patients enrolled in the lower dose cohort experience no DLT or 1 of 6 patients in a cohort has a DLT. If a patient dies within 30 days of receiving investigational treatment, the study will be held until further evaluation, and potential risks vs. benefits of continuing the study will be discussed with the UCLA JCCC DSMB. If the dose regimen in cohort 1 (Ad-CCL21-DC 1 x 10^7 cells/injection) is not well tolerated, de-escalation to Ad-CCL21-DC 5 x 10^6 cells/injection will be allowed (-1). If the dose regimen specified for Cohort (2) (Ad-CCL21-DC 3 x 10^7 cells/ injection) is not the maximum tolerated dose (MTD), no further dose escalation will be conducted, and this dose level will be defined as maximum administered dose (MAD).

Following the completion of the dose-escalation phase, a dose expansion cohort of 12 patients will be enrolled and treated at ExD for up to a year (note: only intravenous pembrolizumab is given after day 42). All enrolled patients will continue to be followed by a physician, and undergo a history and physical examination every 3 months until progressive disease (PD) or withdrawal from the study. Eligible patients will be assigned to a cohort and will receive intratumoral injections of autologous Ad-CCL21-DC and intravenous pembrolizumab in conjunction with tumor sampling and patient monitoring on the days as detailed in **Table 2**.

In determining the Maximum Tolerable Dose (MTD)/Maximum Administered Dose (MAD), no more than 6 patients will be treated at a given dose. The MTD/MAD is defined as the maximum dose level at which fewer than 2 of 6 patients experience a DLT.

The primary objectives of this trial are to determine the safety and efficacy of DC + pembrolizumab. The primary end-points of this phase I study are MTD/MAD and objective response rate (ORR), and the secondary end-points include immunologic and clinical responses.

14.2 <u>Sample Size Determination</u>

In this trial, a modified 3 + 3 dose escalation design will be used for the first dose escalation cohort and six patients will be enrolled in the second dose escalation cohort. Dose escalation will be followed by an expansion cohort at the MTD/MAD will be used. A total sample size of up to 24 (up to 12 in Dose-Escalation Phase, 12 in Expansion Phase) patients is proposed.

In the Dose-Escalation Phase, the number of patients will depend on the number of dose cohorts that will be enrolled before reaching the MTD. Based on the modified 3 + 3 design that will be used for the first dose escalation cohort and the six patients to be enrolled in the second dose escalation cohort, dose escalation is estimated to include approximately 9-12 patients to evaluate safety and tolerability. Escalation to the next dose cohort will depend on the background toxicity rate (i.e., probability of DLT at a given dose). For example, when 1 of 3 patients develops a DLT and the cohort is expanded to 6 subjects, the proposed plan for dose escalation provides a 91% probability that dose escalation will proceed at doses associated with DLT probability of <10%. The table below shows the probability of escalation from cohort to cohort with various toxicity rates.

Background	1%	5%	10%	20%	30%	40%	50%	60%	70%	80%	90%

Toxicity Rate											
Probability of dose escalation	99.9%	97%	91%	71%	49%	31%	17%	8%	3%	1%	0.1%

In the Expansion Phase, 12 additional patients will be evaluated at the dose established during dose escalation (ExD). Therefore, a total of 16 evaluable patients (6 in the dose escalation cohort +12 in the expansion cohort, with an assumed 10% dropout rate) will be used to further assess toxicity, ORR, immunologic and clinical responses. A sample size of 16 evaluable patients achieves 82.9% power to detect a 25% difference in ORR between the null hypothesis: ORR=8% and the alternative hypothesis: ORR=33%, using a two-sided one-sample exact Binomial test, at a 0.10 significance level. The rationale for combining these two patient populations for statistical analyses is that both have similar ORRs to single agent PD-1 inhibitor therapy, with the ORR for treatment with a PD-1 or PD-L1 inhibitor beyond progression is 8% and the ORR of pembrolizumab in patients with sensitizing EGFR mutations on the KEYNOTE-001 trial was 7.7% (Kazandjian et al. 2017; Hellmann 2015).

14.3 Definition of Analysis Populations

The Safety Population will include all subjects who receive at least 1 dose of CCL21 gene-modified DC (Ad-CCL21-DC) + pembrolizumab. The Response Population will include all evaluable subjects. The Response Population will be used for the analysis of biologic and clinical response. During escalation, for a patient to be considered evaluable for the determination of the dose for expansion, a patient must have received at least 2 of 3 injections at the prescribed number of cells (with allowance of cell numbers up to 10% below the prescribed dose to accommodate minor variability). For a patient to be considered evaluable for efficacy, a patient must have received at least 2/3 of the prescribed dose density (number of injections x the average number of cells injected at each injection) of the dose that is selected for expansion (regardless of whether the patient was enrolled during escalation or expansion). So, a patient enrolled during escalation at the dose selected for expansion who receives at least 2/3 (but less than 90%) of the prescribed dose density can be used to assess efficacy.

14.4 Patient Disposition

A detailed description of patient disposition will be provided. It will include a summary of the number and percentage of patients, per Ad-CCL21-DC dose cohort:

- Enrolled
- Received treatment
- Evaluable for safety consideration of dose escalation
- Who violate the protocol
- Who complete the protocol
- Who withdraw because of:
    - AE
    - Disease progression
    - Physician's recommendation
    - Withdrew consent

14.5 Patient Characteristics

Demographic characteristics of patients including age, gender ethnicity, performance status, prior therapies and other baseline characteristics as collected on the case report form will be

summarized by Ad-CCL21-DC dose cohort using descriptive statistics. For continuous variables, descriptive statistics (n, mean, standard deviation, standard error, median, minimum, and maximum) will be provided. For categorical variables, patient counts and percentages will be provided. Categories for missing data will be presented if necessary.

14.6 Planned Methods of Analysis

14.6.1 Safety Analysis

All patients who receive any amount of CCL21 gene-modified DC (Ad-CCL21-DC) + pembrolizumab will be included in the final summaries and listings of safety data. All safety analyses will be performed by dose level, treatment cycle and week, where appropriate.

Summary tables will present the number of patients observed with treatment-emergent adverse events and corresponding percentages by dose cohort. The denominator used to calculate incidence percentages consists of patients receiving at least one dose of CCL21 gene-modified DC (Ad-CCL21-DC) + pembrolizumab in that cohort. Within each summary table, the AEs will be reported and categorized using a CTCAE v5.0 terminology and severity.

Deaths and SAEs will be tabulated on a per-patient basis.

Laboratory data will be presented by dose cohort and visit. Abnormal laboratory values will be assessed according to NCI CTCAE v5.0 where possible.

14.6.2 Efficacy Analysis

The primary efficacy endpoint for this study is the objective response defined using RECIST v1.1 criteria. The ORR will be summarized using descriptive statistics by dose level. The ORR will be analyzed using one-sample exact Binomial test for the patients treated at MTD or MAD in both the dose escalation phase and dose expansion phase. The 95% exact confidence interval (CI) will be provided.

14.6.3 Analysis of Immunologic Data

All immunologic parameters will be summarized using mean, standard deviation, median, percentile for continuous variables, and frequency and percentage for categorical variables. The summary statistics will be tabulated by time and dose level. Several plots, such as stem-and-leaf plot and normal probability plot, will be constructed to check the distribution of each variable. Transformation will be applied if the distribution of the variable is not normal.

For the tumor tissue immune parameters measured at different time points, the post-treatment values will be compared with the pre-treatment values using either paired t-test or Wilcoxon signed-rank test. Box plots for pre-treatment and post-treatment data will be presented.

For the repeatedly measured peripheral blood immune parameters, the immune parameter versus time scatter plot with Loess smooth regression line will be constructed to investigate the time trend. A spline regression model will be developed to characterize the change of immune parameter over time. Comparison between immune parameters will be carried out using the permutation method. Dose level will

be included as a covariate.

Since the analyses of all immune parameters are considered as exploratory, significant level will not be adjusted for multiple outcomes and multiple comparisons.

15 ETHICAL CONSIDERATIONS

The investigator will ensure that the study is conducted in full conformance with the FDA standards for human research as specified in 21 CFR 312.

15.1 Informed Consent

All study participants must sign an informed consent. The investigators will inform all subjects as to the nature, aims, duration, potential hazards, and procedures to be performed during the study and that his or her medical records may be reviewed by the FDA. This protocol must receive approval by the Institutional Review Board prior to implementation. The investigator must also explain that the patients are completely free to refuse to enter the study or to withdraw from it at any time. The protocol will be discussed in detail with all potentially eligible patients. All revisions of the protocol must be reflected in the consent form and reviewed by the IRB. Separate informed consent must be obtained for all surgical procedures.

15.2 Patient Confidentiality

All reports and patient samples will be identified only by a coded number to maintain patient confidentiality. All records will be kept confidential to the extent permitted by law. The investigator should keep a separate log of patients' codes, names, and addresses. Documents which identify the patient by name (informed consent) should be kept in strict confidence.

15.3 Patient's Financial Responsibilities during the Study

Patients will not be financially responsible for any procedures or tests of an investigational nature.

16 CRITERIA AND PROCEDURES FOR PROTOCOL MODIFICATION AND STUDY TERMINATION

Modifications which may affect the safety of the study patient, or which may alter the scope of the investigation, the scientific quality of the study, the study design, dosages, duration of therapy, patient assessments (added evaluation that poses potential risk or inconvenience to the patient), number of patients, and patient eligibility criteria, may be made only after appropriate consultation between the sponsor and the investigator.

If the consensus is to revise the current protocol, a formal List of Changes will accompany the amended protocol and these will be submitted to the FDA, the site's IRB, and other committees as required.

Both the collaborating company and the investigator reserve the right to terminate the study at any time. If this becomes necessary, appropriate procedures for continuing the long-term follow-up requested by the regulatory agencies will be arranged after review and approval by both parties.

17 References

Anagnostou, V. K. and J. R. Brahmer (2015). "Cancer immunotherapy: a future paradigm shift in the treatment of non-small cell lung cancer." Clin Cancer Res **21**(5): 976-984.

Andersson, A., et al. (2009). "IL-7 promotes CXCR3 ligand-dependent T cell antitumor reactivity in lung cancer." J Immunol **182**(11): 6951-6958.

Banchereau, J. and R. M. Steinman (1998). "Dendritic cells and the control of immunity." Nature **392**(6673): 245-252.

Baratelli, F., et al. (2010). "PGE(2) contributes to TGF-beta induced T regulatory cell function in human non-small cell lung cancer." Am J Transl Res **2**(4): 356-367.

Baratelli, F., et al. (2008). "Pre-clinical characterization of GMP grade CCL21-gene modified dendritic cells for application in a phase I trial in non-small cell lung cancer." J Transl Med **6**: 38.

Brahmer, J. R., et al. Clinical activity and biomarkers of MEDI4736, an anti-PD-LI antibody, in patients with NSCLC. 2014 ASCO Annual Meeting, J Clin Oncol 32:5s, 2014 (suppl: abstr 8021).

Bunn, P. A., Jr., et al. (2000). "New therapeutic strategies for lung cancer: biology and molecular biology come of age." Chest **117**(4 Suppl 1): 163S-168S.

Carbone, D. P., et al. (2017). "First-Line Nivolumab in Stage IV or Recurrent Non-Small-Cell Lung Cancer." N Engl J Med **376**(25): 2415-2426.

Carmi, Y., et al. (2015). "Allogeneic IgG combined with dendritic cell stimuli induce antitumour T-cell immunity." Nature **521**(7550): 99-104.

Cerundolo, V., et al. (2004). "Dendritic cells: a journey from laboratory to clinic." Nat Immunol **5**(1): 7-10.

Chan, V. W., et al. (1999). "Secondary lymphoid-tissue chemokine (SLC) is chemotactic for mature dendritic cells." Blood **93**(11): 3610-3616.

Constantino, J., et al. (2017). "Dendritic cell-based immunotherapy: a basic review and recent advances." Immunol Res **65**(4): 798-810.

Cyster, J. G. (1999). "Chemokines and the homing of dendritic cells to the T cell areas of lymphoid organs." J Exp Med **189**(3): 447-450.

Dubinett, S. M., et al. (2000). "Tumor antigens in thoracic malignancy." Am J Respir Cell Mol Biol **22**(5): 524-527.

Dubinett, S. M., et al. (1991). "Cytokine administration alters the distribution of activated lymphocytes to the lung." Pathobiology **59**(6): 372-377.

Dubinett, S. M., et al. (1995). "Down-regulation of murine fibrosarcoma transforming growth factor-beta 1 expression by interleukin 7." J Natl Cancer Inst **87**(8): 593-597.

Dubinett, S. M., et al. (1989). "Adoptive immunotherapy of murine pulmonary metastases with interleukin 2 and interferon-gamma." Am J Respir Cell Mol Biol **1**(5): 361-369.

Dubinett, S. M., et al. (2010). "Chemokines: can effector cells be redirected to the site of the tumor?" Cancer J **16**(4): 325-335.

Dubinett, S. M., et al. (1993). "Intratumoral interleukin-2 immunotherapy: activation of tumor-infiltrating and splenic lymphocytes in vivo." Cancer Immunol Immunother **36**(3): 156-162.

Fong, K. M., et al. (1999). "Molecular pathogenesis of lung cancer." J Thorac Cardiovasc Surg **118**(6): 1136-1152.

Gainor, J. F., et al. (2016). "EGFR Mutations and ALK Rearrangements Are Associated with Low Response Rates to PD-1 Pathway Blockade in Non–Small Cell Lung Cancer: A Retrospective Analysis." Clinical Cancer Research **22**(18): 4585-4593.

Gandhi, L., et al. (2018). "Pembrolizumab plus Chemotherapy in Metastatic Non–Small-Cell Lung Cancer." New England Journal of Medicine **378**(22): 2078-2092.

Garg, A. D., et al. (2017). "Trial watch: Dendritic cell-based anticancer immunotherapy." Oncoimmunology **6**(7): e1328341.

Garon, E. B., et al. (2015). "Pembrolizumab for the treatment of non-small-cell lung cancer." N Engl J Med **372**(21): 2018-2028.

Gettinger, S. N., et al. (2015). "Overall Survival and Long-Term Safety of Nivolumab (Anti-Programmed Death 1 Antibody, BMS-936558, ONO-4538) in Patients With Previously Treated Advanced Non-Small-Cell Lung Cancer." J Clin Oncol **33**: 2004-2012.

Hagenbaugh, A., et al. (1997). "Altered immune responses in interleukin 10 transgenic mice." J Exp Med **185**(12): 2101-2110.

Hellmann MD, G. E., Gandhi L, et al (2015). "MINI03.05 Efficacy of Pembrolizumab in Key Subgroups of Patients with Advanced NSCLC." Journal of Thoracic Oncology **10:S261-S406**.

Herbst, R. S., et al. (2016). "Pembrolizumab versus docetaxel for previously treated, PD-L1-positive, advanced non-small-cell lung cancer (KEYNOTE-010): a randomised controlled trial." Lancet **387**(10027): 1540-1550.

Herbst, R. S., et al. (2014). "Predictive correlates of response to the anti-PD-L1 antibody MPDL3280A in cancer patients." Nature **515**(7528): 563-567.

Hillinger, S., et al. (2003). "EBV-induced molecule 1 ligand chemokine (ELC/CCL19) promotes IFN-gamma-dependent antitumor responses in a lung cancer model." Journal of Immunology **171**(12): 6457-6465.

Huang, M., et al. (1998). "Non-small cell lung cancer cyclooxygenase-2-dependent regulation of cytokine balance in lymphocytes and macrophages: up-regulation of interleukin 10 and down-regulation of interleukin 12 production." Cancer Res **58**(6): 1208-1216.

Kazandjian, D., et al. (2017). "Characterization of outcomes in patients with metastatic non-small cell lung cancer treated with programmed cell death protein 1 inhibitors past RECIST version 1.1–defined disease progression in clinical trials." Seminars in Oncology **44**(1): 3-7.

Keir, M. E., et al. (2008). "PD-1 and its ligands in tolerance and immunity." Annu Rev Immunol **26**: 677-704.

Kim, J., et al. (1995). "IL-10 production in cutaneous basal and squamous cell carcinomas. A mechanism for evading the local T cell immune response." J Immunol **155**(4): 2240-2247.

Kirk, C. J., et al. (2001). "The dynamics of the T-cell antitumor response: chemokine-secreting dendritic cells can prime tumor-reactive T cells extranodally." Cancer Res **61**(24): 8794-8802.

Lee, C. K., et al. (2018). "Clinical and Molecular Characteristics Associated With Survival Among Patients Treated With Checkpoint Inhibitors for Advanced Non–Small Cell Lung Carcinoma: A Systematic Review and Meta-analysisAssociation of Checkpoint Inhibitors With Survival in Advanced Non–Small Cell Lung

CarcinomaAssociation of Checkpoint Inhibitors With Survival in Advanced Non–Small Cell Lung Carcinoma." JAMA Oncology **4**(2): 210-216.

Lee, J. M., et al. (2014). PD-L1 expression correlates with immune response in a Phase I trial of CCL21 gene modified dendritic cell therapy in lung cancer. Journal for ImmunoTherapy of Cancer. **2:** O20.

Lee, J. M., et al. (2017). "Phase I Trial of Intratumoral Injection of CCL21 Gene-Modified Dendritic Cells in Lung Cancer Elicits Tumor-Specific Immune Responses and CD8+ T-cell Infiltration." Clin Cancer Res **23**: 4556-4568.

Liang, C. M., et al. (2007). "Local expression of secondary lymphoid tissue chemokine delivered by adeno-associated virus within the tumor bed stimulates strong anti-liver tumor immunity." J Virol **81**(17): 9502-9511.

Lisberg, A., et al. (2018). "A Phase II Study of Pembrolizumab in EGFR-Mutant, PD-L1+, Tyrosine Kinase Inhibitor Naïve Patients With Advanced NSCLC." Journal of Thoracic Oncology **13**(8): 1138-1145.

Lisberg, A. and E. B. Garon (2019). "Does Platinum-Based Chemotherapy Still Have a Role in First-Line Treatment of Advanced Non–Small-Cell Lung Cancer?" Journal of Clinical Oncology **37**(7): 529-536.

McBride, W. H., et al. (1992). "Genetic modification of a murine fibrosarcoma to produce interleukin 7 stimulates host cell infiltration and tumor immunity." Cancer Res **52**(14): 3931-3937.

McGranahan, N., et al. (2016). "Clonal neoantigens elicit T cell immunoreactivity and sensitivity to immune checkpoint blockade." Science **351**(6280): 1463-1469.

Miller, P. W., et al. (2000). "Intratumoral administration of adenoviral interleukin 7 gene-modified dendritic cells augments specific antitumor immunity and achieves tumor eradication." Human Gene Therapy **11**(1): 53-65.

Nagarsheth, N., et al. (2017). "Chemokines in the cancer microenvironment and their relevance in cancer immunotherapy." Nat Rev Immunol **17**(9): 559-572.

Newman, A. M., et al. (2015). "Robust enumeration of cell subsets from tissue expression profiles." Nat Methods **12**(5): 453-457.

Nishimura, N., et al. (2001). "Enhanced efficiency by centrifugal manipulation of adenovirus-mediated interleukin 12 gene transduction into human monocyte-derived dendritic cells." Hum Gene Ther **12**(4): 333-346.

Novak, L., et al. (2007). "Characterization of the CCL21-mediated melanoma-specific immune responses and in situ melanoma eradication." Mol Cancer Ther **6**(6): 1755-1764.

Okazaki, T., et al. (2001). "PD-1 immunoreceptor inhibits B cell receptor-mediated signaling by recruiting src homology 2-domain-containing tyrosine phosphatase 2 to phosphotyrosine." Proc Natl Acad Sci U S A **98**(24): 13866-13871.

Paglia, P., et al. (1996). "Murine dendritic cells loaded in vitro with soluble protein prime cytotoxic T lymphocytes against tumor antigen in vivo." J Exp Med **183**(1): 317-322.

Pan, J., et al. (2006). "CXCR3/CXCR3 ligand biological axis impairs RENCA tumor growth by a mechanism of immunoangiostasis." J Immunol **176**(3): 1456-1464.

Parra, E. R., et al. (2017). "Validation of multiplex immunofluorescence panels using multispectral microscopy for immune-profiling of formalin-fixed and paraffin-embedded human tumor tissues." Sci Rep **7**(1): 13380.

Paz-Ares, L., et al. (2018). "Pembrolizumab plus Chemotherapy for Squamous Non–Small-Cell Lung Cancer." New England Journal of Medicine **379**(21): 2040-2051.

Politi, K. and R. S. Herbst (2015). "Lung cancer in the era of precision medicine." Clin Cancer Res **21**(10): 2213-2220.

Porgador, A., et al. (1996). "Induction of antitumor immunity using bone marrow-generated dendritic cells." J Immunol **156**(8): 2918-2926.

Reck, M., et al. (2016). "Pembrolizumab versus Chemotherapy for PD-L1-Positive Non-Small-Cell Lung Cancer." N Engl J Med **375**(19): 1823-1833.

Reeves, M. E., et al. (1996). "Retroviral transduction of human dendritic cells with a tumor-associated antigen gene." Cancer Res **56**(24): 5672-5677.

Restifo, N. P., et al. (1993). "Identification of human cancers deficient in antigen processing." J Exp Med **177**(2): 265-272.

Riedl, K., et al. (2003). "Overexpression of CCL-21/secondary lymphoid tissue chemokine in human dendritic cells augments chemotactic activities for lymphocytes and antigen presenting cells." Mol Cancer **2**: 35.

Riley, J. L. (2009). "PD-1 signaling in primary T cells." Immunol Rev **229**(1): 114-125.

Rizvi, N. A., et al. (2015). "Cancer immunology. Mutational landscape determines sensitivity to PD-1 blockade in non-small cell lung cancer." Science **348**(6230): 124-128.

Roberts, E. W., et al. (2016). "Critical Role for CD103(+)/CD141(+) Dendritic Cells Bearing CCR7 for Tumor Antigen Trafficking and Priming of T Cell Immunity in Melanoma." Cancer Cell **30**(2): 324-336.

Sabado, R. L., et al. (2016). "Dendritic cell-based immunotherapy." Cell Res **27**(1): 74-95.

Sharma, P., et al. (2017). "Primary, Adaptive, and Acquired Resistance to Cancer Immunotherapy." Cell **168**(4): 707-723.

Sharma, S., et al. (2003). "Interleukin-7 gene-modified dendritic cells reduce pulmonary tumor burden in spontaneous murine bronchoalveolar cell carcinoma." Human Gene Therapy **14**(16): 1511-1524.

Sharma, S., et al. (1999). "T cell-derived IL-10 promotes lung cancer growth by suppressing both T cell and APC function." Journal of Immunology **163**(9): 5020-5028.

Sharma, S., et al. (2000). "Secondary lymphoid tissue chemokine mediates T cell-dependent antitumor responses in vivo." J Immunol **164**(9): 4558-4563.

Sharma, S., et al. (2001). "Secondary lymphoid organ chemokine reduces pulmonary tumor burden in spontaneous murine bronchoalveolar cell carcinoma." Cancer Res **61**(17): 6406-6412.

Sharma, S., et al. (2003). "SLC/CCL21-mediated anti-tumor responses require IFNgamma, MIG/CXCL9 and IP-10/CXCL10." Mol Cancer **2**: 22.

Sharma, S., et al. (2005). "Tumor cyclooxygenase-2/prostaglandin E2-dependent promotion of FOXP3 expression and CD4+ CD25+ T regulatory cell activities in lung cancer." Cancer Res **65**(12): 5211-5220.

Shepherd, F. A. (2003). "Second-line chemotherapy for non-small cell lung cancer." Expert Review of Anticancer Therapy **3**(4): 435-442.

Sheppard, K. A., et al. (2004). "PD-1 inhibits T-cell receptor induced phosphorylation of the ZAP70/CD3zeta signalosome and downstream signaling to PKCtheta." FEBS Lett **574**(1-3): 37-41.

Socinski, M. A., et al. (2018). "Atezolizumab for First-Line Treatment of Metastatic Nonsquamous NSCLC." New England Journal of Medicine **378**(24): 2288-2301.

Spranger, S., et al. (2017). "Tumor-Residing Batf3 Dendritic Cells Are Required for Effector T Cell Trafficking and Adoptive T Cell Therapy." Cancer Cell **31**(5): 711-723 e714.

Srivastava, M. K., et al. (2012). "Myeloid suppressor cells and immune modulation in lung cancer." Immunotherapy **4**(3): 291-304.

Steinman, R. M. (2012). "Decisions about dendritic cells: past, present, and future." Annu Rev Immunol **30**: 1-22.

Therasse, P., et al. (2000). "New guidelines to evaluate the response to treatment in solid tumors. European Organization for Research and Treatment of Cancer, National Cancer Institute of the United States, National Cancer Institute of Canada." J Natl Cancer Inst **92**(3): 205-216.

Tumeh, P. C., et al. (2014). "PD-1 blockade induces responses by inhibiting adaptive immune resistance." Nature **515**(7528): 568-571.

Vicari, A. P., et al. (2000). "Antitumor effects of the mouse chemokine 6Ckine/SLC through angiostatic and immunological mechanisms." J Immunol **165**(4): 1992-2000.

Wu, S., et al. (2008). "Tumor transfected with CCL21 enhanced reactivity and apoptosis resistance of human monocyte-derived dendritic cells." Immunobiology **213**(5): 417-426.

Yang, S. C., et al. (2006). "Intrapulmonary administration of CCL21 gene-modified dendritic cells reduces tumor burden in spontaneous murine bronchoalveolar cell carcinoma." Cancer Res **66**(6): 3205-3213.

Yang, S. C., et al. (2004). "Intratumoral administration of dendritic cells overexpressing CCL21 generates systemic antitumor responses and confers tumor immunity." Clin Cancer Res **10**(8): 2891-2901.

Yoshino, I., et al. (1994). "HER2/neu-derived peptides are shared antigens among human non-small cell lung cancer and ovarian cancer." Cancer Res **54**(13): 3387-3390.

Yousefieh, N., et al. (2009). "Regulated expression of CCL21 in the prostate tumor microenvironment inhibits tumor growth and metastasis in an orthotopic model of prostate cancer." Cancer Microenviron **2**(1): 59-67.

Zeng, G., et al. (2006). "Dendritic cell surface calreticulin is a receptor for NY-ESO-1: direct interactions between tumor-associated antigen and the innate immune system." J Immunol **177**(6): 3582-3589.

Zhu, L. X., et al. (2003). "IL-10 mediates sigma 1 receptor-dependent suppression of antitumor immunity." J Immunol **170**(7): 3585-3591.

Zou, W., et al. (2016). "PD-L1 (B7-H1) and PD-1 pathway blockade for cancer therapy: Mechanisms, response biomarkers, and combinations." Sci Transl Med **8**(328): 328rv324.

Appendix A: ECOG Performance Status

Grade	Description
0	Normal activity. Fully active, able to carry on all pre-disease performance without restriction.
1	Symptoms, but ambulatory. Restricted in physically strenuous activity, but ambulatory and able to carry out work of a light or sedentary nature (e.g., light housework, office work).
2	In bed <50% of the time. Ambulatory and capable of all self-care, but unable to carry out any work activities. Up and about more than 50% of waking hours.
3	In bed >50% of the time. Capable of only limited self-care, confined to bed or chair more than 50% of waking hours.
4	100% bedridden. Completely disabled. Cannot carry on any self-care. Totally confined to bed or chair.
5	Dead.
* As published in Am. J. Clin. Oncol.: *Oken, M.M., Creech, R.H., Tormey, D.C., Horton, J., Davis, T.E., McFadden, E.T., Carbone, P.P.: Toxicity And Response Criteria Of The Eastern Cooperative Oncology Group. Am J Clin Oncol 5:649-655, 1982.* The Eastern Cooperative Oncology Group, Robert Comis M.D., Group Chair.	

1.1 Common Terminology Criteria for Adverse Events V5.0 (CTCAE)

The descriptions and grading scales found in the revised NCI Common Terminology Criteria for Adverse Events (CTCAE) version 5.0 will be utilized for adverse event reporting. (http://ctep.cancer.gov/reporting/ctc.html)

Appendix B: Response Evaluation Criteria in Solid Tumors (RECIST) v1.1 Criteria for Evaluating Response in Solid Tumors and Patient Withdrawal/ Discontinuation Criteria

RECIST v1.1 Tumor Assessment Worksheet Instructions

1. Refer to the protocol for specific tumor assessment requirements noting:
   a. any protocol specific modifications to RECIST v1.1 criteria – be sure to incorporate these into your assessment (i.e., imaging modality)
   b. screening window for tumor assessment
   c. time-points/windows/frequency of tumor assessments during treatment and follow-up when applicable
      - determine time-point that first assessment is to be done, i.e.,: 6 weeks from randomization date or 6 weeks from C1D1 (+/- 7days)
2. Standard method of assessment for RECIST v1.1 remains grounded in the anatomical assessment of disease by contrast CT of Chest, Abdomen, and Pelvis, and CT or MRI of brain when clinically indicated. If contrast CT is contraindicated, then a non-contrast CT of Chest and MRI of Abdomen and Pelvis should be done. Functional imaging or FDG-PET is not an acceptable method of tumor assessment per RECIST v1.1 criteria; rather it is used as an adjunct to determination of disease progression. Bone scan or plain films are not considered adequate imaging techniques to measure bone lesions; however these techniques can be used to confirm the presence or disappearance of bone lesions. Imaging based evaluation should always be done rather than clinical exam unless the lesion(s) being followed cannot be imaged but are assessable by clinical exam.
3. Radiologist should provide a thorough baseline assessment of all sites of disease, non-nodal and nodal, along with the longest diameter in mm of non-nodal disease and the short axis measurement in mm of nodal disease. Non-disease findings should also be documented in the radiology report and noted in the investigator's note (i.e.: simple cyst, fatty tissue).
4. Using the radiologist's assessment, the investigator should select a total of ten (10) target lesions (TL) with a maximum of five (5) lesions per organ on the basis of their size, be representative of all involved organs, but in addition should be those that lend themselves to reproducible repeated measurements. All other sites of disease should be identified as non-target lesions (NTL). The investigator should document his/her choice and rationale for selection of lesions for baseline assessment of overall tumor burden to be assessed for response. ***Note:*** You may record multiple non-target lesions involving the same organ as a single NTL on the form, i.e.: multiple bone mets, multiple liver mets; multiple enlarged pelvic lymph nodes.
   a. **Target Lesions (TLs)** are defined as non-nodal lesions with a minimum ***longest diameter (LD)*** of≥10mm by CT/MRI; 10mm caliper measurement by clinical exam (documented with photo and ruler); ≥20mm by chest X-ray (CT is preferred; however, lesions on chest X-ray may be considered if clearly defined and surrounded by aerated lung); and lymph nodes ≥15mm in the ***short axis (SA)***. **Note:** Measure the longest diameter of the node first. Then determine the longest dimension perpendicular to the longest diameter and report that measurement as the short axis. (For example: a cervical lymph node is reported as being 17mm x 22mm. The SA measurement would be 17mm). ***Note:*** Lytic or mixed lytic-blastic bone lesions with identifiable soft tissue components that can be evaluated by CT/MRI and meet the definition of measurability can be considered as measurable lesions
   b. **Non-Target Lesions (NTLs)** are defined as non-measurable and include: any measurable lesions that were not selected as target lesions; non-nodal lesions <10mm in ***longest diameter***; lymph nodes that have a ***short axis*** ≥10mm and <15mm ***Note:*** SA nodal non-target lesion must have a reduction in SA to <10mm to be considered a CR; therefore, documenting nodal non-target lesions SA is recommended. ; blastic bone lesions, lytic or mixed lytic-blastic lesions without an identifiable soft tissue component that meets the definition of measurable, and lesions considered truly non-measurable which include: leptomeningeal disease, ascites, pleural/pericardial effusions, inflammatory breast disease, lymphangitic involvement of skin/lung, abdominal

masses/abdominal organomegaly identified by physical exam that is not measurable by reproducible imaging techniques. ***Note:*** lesions situated in a previously irradiated area are considered non-measurable lesions (non-target) unless there has been demonstrated progression in the lesion and then it may be considered a target lesion.

   c. **Non-Pathological Lesions** include nodes that have a short axis of <10mm and simple cysts. These should not be recorded as target or non-target lesions.

5. List each selected baseline target lesion under the column "Lesion Description" and record the LD/SA (Largest Diameter(non-nodal)/Short Axis(nodal)) for each lesion in the corresponding Baseline LD/SA mm column. Baseline assessment of overall tumor burden is calculated by adding the longest diameter measurement of non-nodal target lesions and the short axis measurement for target nodal lesions in mm and reporting this as the baseline Sum of the Diameters (SOD). Add the baseline measurements and record the SOD in the corresponding space.
6. List all non-target sites of disease under the column "Lesion Description" and record as present in the corresponding Baseline Present/Absent column. Measurements are not required and should be followed as "present", "absent", or in rare cases "unequivocal progression" (write this in space when applicable). Note***:*** There are not a maximum number of non-target lesions; therefore, you may need to use an additional assessment form to document all non-target lesions. However, keep in mind that multiple non-target lesions involving the same organ may be recorded as a single item, i.e., multiple liver mets
7. Document the time-point response of Target Lesions in the row labeled "**TL Response"** that corresponds to the specific assessment time-point. Target lesion response is determined by the SOD of subsequent tumor assessments. **Note**: All Target Lesions (nodal and non-nodal) recorded at baseline should have their actual measurements recorded at each subsequent evaluation, even when very small. If it is the opinion of the radiologist that the lesion has likely disappeared, the measurement should be recorded as "0"mm. If the lesion is believed to be present and is faintly seen but too small to measure, a default value of 5mm should be recorded.
   a. **Complete Response (CR)**: Disappearance of all target lesions (each pathological lymph nodes must have a reduction in short axis to <10mm)
   b. **Partial Response (PR)**: $\geq$30% decrease in the **baseline SOD**. To find the PR threshold, at baseline, multiple the baseline SOD x 0.7 and record this in the corresponding spaces for each tumor assessment time-point. *Note: This calculation only needs to be calculated at baseline.* For example: Baseline SOD = 32mm x 0.7 = 22.4mm; therefore, on subsequent assessments, the SOD would have to be $\leq$22.4mm to be documented and recorded as a PR.
   c. **Progressive Disease (PD):** $\geq$20% increase in the **Nadir SOD** (this includes the Baseline SD if it is the smallest on study) ***+ an absolute increase of 5mm over the nadir SOD***. The appearance of one or more new lesions representing disease is also considered PD. To find the PD threshold, determine the smallest SD recorded and multiple it by 1.20 and record this calculation in the corresponding space for each tumor assessment time-point. *Note: This calculation must be done at each assessment*. Example: Baseline SOD = 43mm PD threshold = 43 x 1.20 = 51.6mm. Assessment #2 SOD = 35mm, = 1.2 x 35 = new PD threshold = 42mm Assessment #3 SOD = 37mm. PD threshold = 42mm (Nadir SOD of 35mm). Assessment #4 SOD = 43mm = >20% ↑ over nadir SOD (Assessment #2) and the SOD of 43mm is an absolute increase of 8mm over the nadir SOD of 35mm.
   d. **Stable Disease (SD):** There is neither sufficient shrinkage (compared to baseline) to qualify for PR nor sufficient increase (taking as reference the smallest SOD while on study) to qualify for PD.
   e. **Not All Evaluated (NE)**: When no imaging/measurement is done at all at a particular assessment time point or only a subset of lesion measurements are made at an assessment (unless documentation is made that the contribution of the individual missing lesion(s) measurements would not change the assigned time point response. This would most likely happen in the case of PD). ***Note:*** Make all attempts at obtaining/clarifying measurements for all baseline sites of disease at each subsequent assessment time-point.

8. Document the time-point response of Non-Target Lesions in the row labeled **"NTL Response"** that corresponds to the specific assessment time-point. Non Target Lesion response is defined as:
    a. **Complete Response (CR)**: Disappearance of all non-target lesions and normalization of tumor marker level. All lymph nodes must be non-pathological in size (<10mm short axis)
    b. **Non CR/Non PD**: Persistence of one or more non-target lesions and/or maintenance of tumor marker level above normal limits
    c. **Progressive Disease (PD):** Unequivocal progression of existing non-target lesions. The appearance of one or more new lesions is also considered progression. ***Note:*** When there is measurable disease, to achieve unequivocal progression, there must be an overall level of substantial worsening in non-target disease, such that even in presence of SD or PR of target disease, the overall tumor burden has increased sufficiently to merit discontinuation of therapy. When there is only non-measurable disease, there needs to be an increase in tumor burden representing an additional 73% increase in volume (example: increase in pleural effusion from trace to large)
    d. **Not All Evaluated (NE)**: When none or only some imaging is done at a particular assessment time point.
9. Document the presence or absence of any new lesion (Target or Non-Target) in the row labeled **"NEW LESION"** at each assessment time point after baseline. This represents disease progression; however, this finding should be unequivocal and not attributable to differences in scanning technique, change in imaging modality or findings thought to represent something other than tumor (Example: a new bone lesion may represent healing or flare of pre-existing lesions).
    a. A lesion identified on a follow-up study in an anatomical location that **was not scanned at baseline is considered a new lesion** and will indicate disease progression
    b. If a new lesion is equivocal (i.e. because of small size), continued therapy and follow up evaluation may clarify if it truly represents new disease. Discuss and obtain approval from medical monitor to continue treatment and re-evaluate. If re-eval confirms it as a new lesion, then PD should be recorded as the date of initial scan.
10. Document the overall response in the row labeled **"OVERALL RESPONSE"** at each assessment time point after baseline.
11. Document the review and confirmation date of the Clinical Investigator in the row labeled **"Investigator Signature/Date"** at baseline and each subsequent assessment time point. ***Important:*** This is source documentation that disease burden and response have been confirmed by the investigator prior to continued study treatment after each assessment time point. In addition, the investigator's documentation should reflect any clarifications related to imaging/treatment discussed with radiologist and/or study monitor.

Additional RECIST v1.1 Guidance:

1. **Special Notes on Response Assessment:**

    Complete Response:

    - When nodal disease is included in the Sum of Largest Diameter of target lesions and the nodes decrease to "normal" size (<10mm), measurements may still be reported on scans. The measurements should be recorded even though the nodes are normal to prevent the overstatement of progression should progression be based on increase in the size of the nodes. This means that responses with CR may not have a total sum of "zero".
    - When it is difficult to distinguish residual disease from normal tissue and the evaluation of CR depends upon this determination, it is recommended that the residual lesion be investigated (FNA/Biopsy) before assigning a status of CR. FDG-PET may be used to upgrade a response to CR in cases where a residual radiographic abnormality is thought to represent fibrosis or scarring. *Note: both approaches may lead to false positive CR due to limitations of FDG-PET and biopsy resolution/sensitivity.*

    Progression:

- For equivocal findings of progression (i.e., very small and uncertain new lesions, cystic changes or necrosis in existing lesions), treatment may continue until the next scheduled assessment. If at the next scheduled assessment, progression is confirmed, the date of progression should be the earlier date when progression was suspected. ***Note:*** The investigator's documentation should reflect the equivocal findings with decision to continue to treat until the next scheduled assessment.
- Patients with global deterioration of health status requiring discontinuation of treatment *without objective evidence of disease progression* at that time should be reported as **"symptomatic deterioration"**. Every effort should be made to document objective progression even after discontinuation of treatment. Symptomatic deterioration is a reason for discontinuing study therapy, not an objective response which is determined by evaluation of target and non-target disease.

Target Lesions that Split or Coalesce:

- When non-nodal lesions split, the longest diameters of the split portions should be added together to calculate the target lesion sum
- When a non-nodal lesion has coalesced (no longer separable), the vector of the longest diameter should be the maximal longest diameter for the coalesced lesion

Target Lesions that become "too small to measure":

- Actual measurements of all baseline nodal and non-nodal lesions should be recorded at each subsequent assessment, even when very small (i.e., 2mm). If the radiologist is not comfortable with assigning an exact measurement and the radiologist feels
    - that the lesion has likely disappeared, the measurement should be recorded as "0"mm
    - that the lesion is believed to be present and is faintly seen but too small to measure, a default value of "5"mm should be recorded

2. **Confirmatory Measurement of Response**
    a. In non-randomized trials where response is the primary endpoint, confirmation of CR and PR is required to ensure responses identified are not due to measurement error.
    b. In randomized (phase II or III) trials where SD or PD are the primary endpoints, confirmation of response is not required
    c. Stable Disease measurements must have met the SD criteria at least once after study entry at a minimum interval (in general $\geq$ 6-8 weeks) that is defined in the study protocol.

Modified RECIST Definitions:

- Definition for SD, PR, and CR are the same as RECIST v1.1.
- PD by modified RECIST: Although the same rules for progression apply for modified RECIST, if the subject is clinically stable, at the discretion of the PI, they may continue treatment until the next imaging time points ≥4 weeks later. If PD is confirmed, the date of PD is the same as the initial date of PD by RECIST. If PD is not confirmed, the date of PD is based on the second event for PD by imaging.

- The following scenarios defined confirmation of PD or lack thereof at the repeat imaging time point. Note that if PD is not confirmed, imaging should resume the regular interval per the schedule. Also, if a second PD event occurs, no further confirmation of PD is required. Note; if PD is confirmed and the subject is achieving a clinically meaningful benefit, the site should speak with DSMB to discuss the potential to continue on treatment.

PD IS CONFIRMED:

1. If initial PD is due to increase in tumor burden ≥ 20% compared to nadir, PD is Confirmed if ANY of the following are met:
    - At follow-up scan tumor burden remains ≥ 20% compared to nadir + 5mm absolute increase if tumor burden is small
    - New unequivocal PD of non target lesion (NTL) (qualitative)
    - One or more new lesion(s)
    - Clinical instability

2. If initial PD is due to unequivocal PD of NTL, PD is CONFIRMED at follow up if ANY of the following conditions are met:
    - NT disease resulting in initial PD is worse (qualitative)
    - New additional unequivocal PD of NTL
        - (qualitatively assessed)
    - Additional new lesion (s)
    - New PD due to tumor burden (SOD) ≥ 20% compared to nadir+ 5 mm absolute increase if tumor burden is small

3. If PD is due to New Lesion, PD is CONFIRMED at follow up time point if ANY of the following conditions are met:
    - New lesion resulting in initial PD is worse (qualitative)
    - Additional new lesion (s)
    - New unequivocal PD of non target (qualitative)
    - New PD due to tumor burden (SOD) ≥ 20% compared to nadir + 5 mm absolute increase if tumor burden is small

PD IS NOT CONFIRMED AT FOLLOW UP IMAGING FOR THE FOLLOWING SCENARIOS :

4. If PD is due to increase in tumor burden by 20% compared to nadir, PD is NOT CONFIRMED at follow up if ALL of the following conditions are met
    - Tumor burden is <20% compared to nadir
    - No unequivocal PD of non target lesion (qualitative)
    - No new lesion(s)
    - Continued clinical stability

5. If PD is due to New Lesion(s), PD is NOT CONFIRMED at follow up time point if ALL of the following conditions are met:
    - Previous new lesion (that was initially called PD) is not worse (qualitative)
    - No additional new lesion(s)
    - No unequivocal PD of non target (qualitative)
    - No new PD due to tumor burden (SOD) ≥ 20% compared to nadir
    - Continued clinical stability

6. If PD is due to unequivocal PD of non target, PD is NOT CONFIRMED at follow up if ALL of the following conditions are met:
    - Initial PD of non target disease is not worse (qualitative)
    - No additional new unequivocal PD of non target qualitative)
    - No new lesion(s)
    - No new PD due to tumor burden (SOD) ≥ 20% compared to nadir

- Continued clinical stability

Time point response of Measurable Disease (Target Lesions) with or without Non-Measurable Disease (Non Target Lesions)

Target Lesion Response	Non Target Lesion	New Lesions	Overall Response
CR	CR	No	CR
CR	Non-CR/Non-PD	No	PR
CR	NE	No	PR
PR	Non-PD or NE	No	PR
SD	Non-PD or NE	No	SD
NE	Non-PD	No	NE
PD	Any	Yes or No	PD
Any	PD	Yes or No	PD
Any	Any	Yes	PD
CR=Complete Response, PR=Partial Response, SD=Stable Disease, PD=Progressive Disease, and NE=Not All Evaluated			

Time point response of Non-Measurable Disease Only (Non Target Lesions)

Non Target Lesion Response	New Lesions	Overall Response
CR	No	CR
Non-CR/Non-PD	No	Non-CR/Con-PD
NE	No	NE
Unequivocal Progression	Yes or No	PD
Any	Yes	PD
CR=Complete Response, PD=Progressive Disease, and NE=Not All Evaluated<br>Non-CR/Non-PD is preferred over "Stable Disease" for non-target disease since SD is increasingly used as endpoint for assessment of efficacy in some trials so to assign this category when no lesions can be measured is not advised		

Note: If a CR is truly met at a time point, then any disease seen at a subsequent time point, even disease meeting PR criteria relative to baseline, makes the disease PD at that time point since disease must have reappeared after CR.

Participant Withdrawal/Discontinuation Criteria

Participants may discontinue study treatment at any time for any reason or be dropped from the study treatment at the discretion of the investigator should any untoward effect occur. In addition, a participant may be discontinued from study treatment by the investigator or the Sponsor if study treatment is

inappropriate, the trial plan is violated, or for administrative and/or other safety reasons.

A participant must be discontinued from study treatment but continue to be monitored in the study for any of the following reasons:

- The participant or participant's legally acceptable representative requests to discontinue study treatment
- Confirmed radiographic disease progression, as outlined in the protocol
- Any progression or recurrence of any malignancy that requires active treatment, or any occurrence of another malignancy that requires active treatment
- Unacceptable adverse experiences as described in the protocol
- The participant has a medical condition or personal circumstance which, in the opinion of the investigator and/or sponsor, placed the participant at unnecessary risk from continued administration of study treatment.
- The participant has a confirmed positive serum pregnancy test
- Noncompliance with study treatment or procedure requirements
- Recurrent Grade 2 pneumonitis
- The participant is lost to follow-up

Appendix C: Contraceptive Guidance and Pregnancy Testing

Woman of Childbearing Potential (WOCBP)

A woman is considered fertile following menarche and until becoming post-menopausal unless permanently sterile (see below)

Women in the following categories are not considered WOCBP:

- Premenarchal
- Premenopausal female with 1 of the following:
  - Documented hysterectomy
  - Documented bilateral salpingectomy
  - Documented bilateral oophorectomy

  Note: Documentation can come from the site personnel's review of the participant's medical records, medical examination, or medical history interview.

- Postmenopausal female
  - A postmenopausal state is defined as no menses for 12 months without an alternative medical cause.
    - A high follicle stimulating hormone (FSH) level in the postmenopausal range may be used to confirm a postmenopausal state in women not using hormonal contraception or hormonal replacement therapy (HRT). However, in the absence of 12 months of amenorrhea, confirmation with two FSH measurements in the postmenopausal range is required.
  - Females on HRT and whose menopausal status is in doubt will be required to use one of the non-hormonal highly effective contraception methods if they wish to continue their HRT during the study. Otherwise, they must discontinue HRT to allow confirmation of postmenopausal status before study enrollment.

Contraception Requirements

Male Participants:

Male participants with female partners of childbearing potential are eligible to participate if they agree to one of the following during the protocol defined time frame in section 4:

- Be abstinent from penile-vaginal intercourse as their usual and preferred lifestyle (abstinent on a long term and persistent basis) and agree to remain abstinent
- Use a male condom plus partner use of a contraceptive method with a failure rate of <1% per year as described in Table 8 when having penile-vaginal intercourse with a woman of childbearing potential who is not currently pregnant.
    - Note: Men with a pregnant or breastfeeding partner must agree to remain abstinent from penile-vaginal intercourse or use a male condom during each episode of penile penetration.

Female Participants:

Female participants of childbearing potential are eligible to participate if they agree to use a highly effective method of contraception that has a low user dependency consistently and correctly as described in Table 8 during the protocol-defined time frame in Section 4.

Table 8 Highly Effective Contraceptive Methods That Have Low User Dependency

**Highly Effective Methods That Have Low User Dependency**<br>*Failure rate of <1% per year when used consistently and correctly.*
● Progestogen- only contraceptive implant [a, b]<br>● Intrauterine hormone-releasing system (IUS) [b]<br>● Intrauterine device (IUD)<br>● Bilateral tubal occlusion
● **Vasectomized partner**<br>A vasectomized partner is a highly effective contraception method provided that the partner is the sole male sexual partner of the WOCBP and the absence of sperm has been confirmed. If not, an additional highly effective method of contraception should be used.
● **Sexual abstinence**<br>Sexual abstinence is considered a highly effective method only if defined as refraining from heterosexual intercourse during the entire period of risk associated with the study treatment. The reliability of sexual abstinence needs to be evaluated in relation to the duration of the study and the preferred and usual lifestyle of the participant.
Notes:<br>Use should be consistent with local regulations regarding the use of contraceptive methods for participants of clinical studies.<br><br>a) If locally required, in accordance with Clinical Trial Facilitation Group (CTFG) guidelines, acceptable contraceptive implants are limited to those which inhibit ovulation.<br>b) Condoms must be used in addition to the hormonal contraception during the treatment period and for at least 4 months after the last dose of study treatment.

Pregnancy Testing

WOCBP should only be included after a negative highly sensitive urine or serum pregnancy test.

Pregnancy testing will be performed whenever an expected menstrual cycle is missed or when pregnancy is otherwise suspected.
