# Supplementary material for: A CCL21 gene-modified dendritic cell vaccine combined with pembrolizumab in advanced non-small cell lung cancer: a phase I trial
Source: Nat Commun. 2026 Oct 4;17:11321. doi: 10.1038/s41467-026-78130-7 (PMC13724721; doi:10.1038/s41467-026-78130-7)
Supplement: Supplementary file 2 [file 41467_2026_78130_MOESM2_ESM.pdf]

Description of Additional Supplementary Files

Supplementary Data 1: Differentially expressed genes enriched in novel versus pre-existing T cell clones in tumor biopsies.

Supplementary Data 2: Genes with increased expression over time in peripheral blood mononuclear cell samples.
