# Supplementary material for: A CCL21 gene-modified dendritic cell vaccine combined with pembrolizumab in advanced non-small cell lung cancer: a phase I trial
Source: Nat Commun. 2026 Oct 4;17:11321. doi: 10.1038/s41467-026-78130-7 (PMC13724721; doi:10.1038/s41467-026-78130-7)
Supplement: Supplementary file 3 [file 41467_2026_78130_MOESM4_ESM.pdf]

nature portfolio

Corresponding author(s): Edward Garon, Bin Liu, Steven Dubinett

Last updated by author(s): May 13, 2026

Reporting Summary

Nature Portfolio wishes to improve the reproducibility of the work that we publish. This form provides structure for consistency and transparency in reporting. For further information on Nature Portfolio policies, see our Editorial Policies and the Editorial Policy Checklist.

Statistics

For all statistical analyses, confirm that the following items are present in the figure legend, table legend, main text, or Methods section.

n/a	Confirmed	
☐	☒	The exact sample size (*n*) for each experimental group/condition, given as a discrete number and unit of measurement
☐	☒	A statement on whether measurements were taken from distinct samples or whether the same sample was measured repeatedly
☐	☒	The statistical test(s) used AND whether they are one- or two-sided<br>*Only common tests should be described solely by name; describe more complex techniques in the Methods section.*
☐	☒	A description of all covariates tested
☐	☒	A description of any assumptions or corrections, such as tests of normality and adjustment for multiple comparisons
☐	☒	A full description of the statistical parameters including central tendency (e.g. means) or other basic estimates (e.g. regression coefficient) AND variation (e.g. standard deviation) or associated estimates of uncertainty (e.g. confidence intervals)
☐	☒	For null hypothesis testing, the test statistic (e.g. *F*, *t*, *r*) with confidence intervals, effect sizes, degrees of freedom and *P* value noted<br>*Give P values as exact values whenever suitable.*
☒	☐	For Bayesian analysis, information on the choice of priors and Markov chain Monte Carlo settings
☒	☐	For hierarchical and complex designs, identification of the appropriate level for tests and full reporting of outcomes
☐	☒	Estimates of effect sizes (e.g. Cohen's *d*, Pearson's *r*), indicating how they were calculated

Our web collection on statistics for biologists contains articles on many of the points above.

Software and code

Policy information about availability of computer code

Data collection	MIF images were obtained using the Vectra Polaris (v1.0, Akoya Biosciences). Slide scans were then imported into HALO (v3.6, Indica Labs) for stitching, selecting regions of interest, cell segmentation, and signal quantification.<br>CellRanger 9.0 (10X Genomics) was used to align raw reads from single cell RNA sequencing data.<br>TCR-β complementarity determining region 3 (CDR3) sequencing was performed with the immunoSEQ platform (Adaptive Biotechnologies).
Data analysis	Statistical analyses were performed in R (v4.4.2) for scRNA/TCR-seq and otherwise in Prism 9 (GraphPad).<br><br>Sequencing analyses were performed in R using established algorithms and open source code that we have cited. These packages include:<br>- Strelka2 (v2.9.10), Mutect2 (v4.1), and ABRA for whole exome sequencing<br>- cfSNV (v0.99.0) for ctDNA analysis<br>- DropletUtils (v1.26.0), scran (v1.34.0), scater (v1.34.1), and harmony (v1.2.4) for basic processing of scRNA-seq data<br>- scRepertoire (v2.2.1) and immunarch (v0.10.3) for analyzing TCR-seq data<br>- DESeq2 (v1.46.0) for DEG analysis<br>- clusterProfiler (v4.14.6) and AUCell (v1.28.0) for gene set enrichment and signature scoring<br>- CellChat (v2.2.0) for cell-cell interaction inference<br>- lme4 package (v1.1.37) for modeling linear mixed effects

For manuscripts utilizing custom algorithms or software that are central to the research but not yet described in published literature, software must be made available to editors and reviewers. We strongly encourage code deposition in a community repository (e.g. GitHub). See the Nature Portfolio guidelines for submitting code & software for further information.

Data

Policy information about availability of data

All manuscripts must include a data availability statement. This statement should provide the following information, where applicable:

- Accession codes, unique identifiers, or web links for publicly available datasets
- A description of any restrictions on data availability
- For clinical datasets or third party data, please ensure that the statement adheres to our policy

The study protocol is available in the Supplementary Information file. Raw single cell RNA sequencing data is deposited in the Sequence Read Archive under BioProject accession code PRJNA1348488 (https://www.ncbi.nlm.nih.gov/bioproject/?term=PRJNA1348488). Processed data including count matrices are deposited in the Gene Expression Omnibus under accession codes GSE317309 (https://www.ncbi.nlm.nih.gov/geo/query/acc.cgi?acc=GSE317309) and GSE345124 (https://www.ncbi.nlm.nih.gov/geo/query/acc.cgi?acc=GSE345124). Tumor and peripheral blood WES data generated in this study have been deposited in the European Genome-phenome Archive (EGA) under accession code EGAS50000002127 (https://ega-archive.org/studies/EGAS50000002127). This data can be made available through an approved Data Use Agreement for research purposes only. WES data cannot be made publicly available for three of the patients as they did not sign a consent that explicitly allowed WES deposition. Additional data, including de-identified participant data, will be shared on reasonable request for non-commercial research purposes. Requests for de-identified individual participant data can be made to the corresponding author, and this data will be shared up to 5 years after publication through an approved Data Use Agreement. Source data are provided with this paper.

Research involving human participants, their data, or biological material

Policy information about studies with human participants or human data. See also policy information about sex, gender (identity/presentation), and sexual orientation and race, ethnicity and racism.

Reporting on sex and gender	Both male (n=10) and female (n=13) participants were included in this study. Sex was self-reported and recorded at enrollment. Due to the small sample size, the study was not powered to detect sex-specific effects, and formal sex-stratified analyses were not performed.
Reporting on race, ethnicity, or other socially relevant groupings	Race and ethnicity were self-reported at enrollment. To preserve participant confidentiality and avoid over-interpretation, only race categories with more than one participant were reported individually; all other categories were grouped as "Other." Ethnicity was reported as either "Hispanic or Latino" or "Non-Hispanic or Latino" in accordance with standard clinical research conventions. Given the limited sample size, the study was not powered to evaluate race- or ethnicity-specific differences, and no subgroup analyses were performed.
Population characteristics	Clinical and demographic data collected at baseline included age, sex, race/ethnicity, smoking status, prior lines of therapy, and tumor pathology. This data was reported in the aggregate to preserve patient privacy. Patients were stratified based on their treatment response (stable disease vs progressive disease).
Recruitment	Participants were recruited from the UCLA patient population and through physician referrals. As a single-center study conducted at an academic medical center, the cohort may reflect local and regional demographic characteristics. Selection biases historically associated with clinical trial enrollment may have affected recruitment as well.
Ethics oversight	The study was conducted in accordance with the protocol, the International Council for Harmonization Good Clinical Practice guidelines, and the Declaration of Helsinki. The study protocol received approval from the UCLA Institutional Review Board, and all patients provided written informed consent.

Note that full information on the approval of the study protocol must also be provided in the manuscript.

Field-specific reporting

Please select the one below that is the best fit for your research. If you are not sure, read the appropriate sections before making your selection.

[x] Life sciences [ ] Behavioural & social sciences [ ] Ecological, evolutionary & environmental sciences

For a reference copy of the document with all sections, see nature.com/documents/nr-reporting-summary-flat.pdf

Life sciences study design

All studies must disclose on these points even when the disclosure is negative.

Sample size	A sample size of 16 patients was estimated to provide 82.9% power to detect an absolute difference of 25% in objective response rate from the null hypothesis of 8% to the alternative hypothesis of 33%, using a two-sided one-sample exact binomial test at a 10% significance level.
Data exclusions	Efficacy was evaluated only for patients who received at least 2 doses at the maximum administered dose of the CCL21-DC vaccine, which was pre-specified in the study protocol, resulting in 4 patients excluded from the efficacy cohort.
Replication	Unable to replicate due to all data being derived from a clinical trial.
Randomization	No randomization due to being a single-arm clinical trial. Samples were analyzed according to clinical outcomes.
Blinding	Blinding was not possible due to being a single-arm clinical trial.

Reporting for specific materials, systems and methods

We require information from authors about some types of materials, experimental systems and methods used in many studies. Here, indicate whether each material, system or method listed is relevant to your study. If you are not sure if a list item applies to your research, read the appropriate section before selecting a response.

Materials & experimental systems

n/a	Involved in the study
☐	☒ Antibodies
☐	☒ Eukaryotic cell lines
☒	☐ Palaeontology and archaeology
☒	☐ Animals and other organisms
☐	☒ Clinical data
☒	☐ Dual use research of concern
☒	☐ Plants

Methods

n/a	Involved in the study
☒	☐ ChIP-seq
☒	☐ Flow cytometry
☒	☐ MRI-based neuroimaging

Antibodies

Antibodies used

MIF analysis was performed using the following antibodies:

- Primary: pan-cytokeratin (PanCK, 1:100 dilution, Agilent #M351501–2), CD3 (no dilution, Ventana Medical Systems #790–4341), CD8 (1:200, Leica Biosystems #CD8–4B11-L-CE), FOXP3 (1:200, Cell Signaling Technology #98377), granzyme B (1:200, Leica Biosystems #NCL-L-GRAN-B), and Ki67 (1:200, Ventana Medical Systems #790–4286)
- Secondary: Opal 480, 520, 570, 690, 780, or TSA-DIG (Akoya Biosciences)

Validation

Validation of all antibodies were performed by the manufacturers. Information can be found on their websites listed below:

- PanCK: https://www.agilent.com/store/productDetail.jsp?catalogId=M351501-2
- CD3: https://diagnostics.roche.com/us/en/products/lab/cd3-2gv6-confirm-rtd000768.html
- CD8: https://shop.leicabiosystems.com/us/ihc-ish/ihc-primary-antibodies/pid-cd8
- FOXP3: https://www.cellsignal.com/products/primary-antibodies/foxp3-d2w8e-rabbit-monoclonal-antibody/98377
- Granzyme B: https://shop.leicabiosystems.com/us/ihc-ish/ihc-primary-antibodies/pid-granzyme-b
- Ki67: https://diagnostics.roche.com/us/en/products/lab/ki-67-30-9-confirm-rtd000792.html
- Opal 480: https://www.quanterix.com/spatial-assay-kits-and-reagents/opal-480-reagent-pack/
- Opal 520: https://www.quanterix.com/spatial-assay-kits-and-reagents/opal-520-reagent-pack/
- Opal 570: https://www.quanterix.com/spatial-assay-kits-and-reagents/opal-570-reagent-pack/
- Opal 690: https://www.quanterix.com/spatial-assay-kits-and-reagents/opal-690-reagent-pack/
- Opal 780: https://www.quanterix.com/spatial-assay-kits-and-reagents/opal-780-reagent-pack/
- Opal TSA-DIG: https://www.quanterix.com/spatial-assay-kits-and-reagents/tsa-plus-dig/

Eukaryotic cell lines

Policy information about cell lines and Sex and Gender in Research

Cell line source(s)	The KPL cell line was derived from a genetically-engineered mouse primary lung tumor.
Authentication	None of the cell lines used were authenticated.
Mycoplasma contamination	All cell lines tested negative for Mycoplasma contamination.
Commonly misidentified lines (See ICLAC register)	No commonly misidentified lines were used.

Clinical data

Policy information about clinical studies

All manuscripts should comply with the ICMJE guidelines for publication of clinical research and a completed CONSORT checklist must be included with all submissions.

Clinical trial registration	NCT03546361
Study protocol	Available on reasonable request
Data collection	Study occurred at UCLA hospitals and oncology clinics. Patient recruitment and enrollment occurred from July 2019 to December 2023. Data collection occurred from July 2019 until the data cutoff date of June 1, 2024.

Outcomes

The primary outcomes of the study were the maximum administered dose (MAD), predefined as the maximum dose tolerated during the 3+3 dose escalation phase, and the objective response rate (ORR), predefined as the percentage of patients with an objective response according to RECIST v1.1 guidelines. The secondary outcomes were the AE profile as well as drug target activity as determined by analyzing serial pre- and post-treatment biopsies and blood specimens. AEs were graded according to the National Cancer Institute Common Terminology Criteria for Adverse Events (CTCAE) v5.0. trAEs were defined as AEs possibly related to either the CCL21-DC vaccine and/or pembrolizumab. Additional efficacy endpoints included progression-free survival (PFS, defined as time from date of enrollment to date of progression per RECIST v1.1 guidelines), and overall survival (OS, defined as time from date of enrollment to date of death).

Plants

Seed stocks

Report on the source of all seed stocks or other plant material used. If applicable, state the seed stock centre and catalogue number. If plant specimens were collected from the field, describe the collection location, date and sampling procedures.

Novel plant genotypes

Describe the methods by which all novel plant genotypes were produced. This includes those generated by transgenic approaches, gene editing, chemical/radiation-based mutagenesis and hybridization. For transgenic lines, describe the transformation method, the number of independent lines analyzed and the generation upon which experiments were performed. For gene-edited lines, describe the editor used, the endogenous sequence targeted for editing, the targeting guide RNA sequence (if applicable) and how the editor was applied.

Authentication

Describe any authentication procedures for each seed stock used or novel genotype generated. Describe any experiments used to assess the effect of a mutation and, where applicable, how potential secondary effects (e.g. second site T-DNA insertions, mosiacism, off-target gene editing) were examined.
