# Supplementary material for: A CCL21 gene-modified dendritic cell vaccine combined with pembrolizumab in advanced non-small cell lung cancer: a phase I trial
Source: Nat Commun. 2026 Oct 4;17:11321. doi: 10.1038/s41467-026-78130-7 (PMC13724721; doi:10.1038/s41467-026-78130-7)
Supplement: Supplementary file 4 [file 41467_2026_78130_MOESM5_ESM.pdf]

nature portfolio

Peer Review File

A CCL21-gene modified dendritic cell vaccine combined with pembrolizumab in advanced non-small cell lung cancer: a phase I trial

Corresponding Author: Professor Steven Dubinett

This file contains all reviewer reports in order by version, followed by all author rebuttals in order by version.

Version 0:

Reviewer comments:

Reviewer #1

(Remarks to the Author)
This study is a rigorously designed and in-depth Phase I clinical trial, which for the first time evaluated the safety, preliminary efficacy, and immunological effects of intratumoral injection of CCL21 gene-modified dendritic cell (DC) vaccine combined with pembrolizumab in patients with advanced non-small cell lung cancer (NSCLC) in humans. Its key highlights are as follows:
1. Innovative Combination Therapy Strategy
The study aims to recruit T cells into the tumor microenvironment (TME) via DC vaccines expressing the chemokine CCL21, and combine this with PD-1 inhibitor to block immunosuppressive signals. Theoretically, this strategy is expected to synergistically enhance the anti-tumor immune response, especially in patients who have developed resistance to previous immunotherapy.
2. Comprehensive and In-depth Immune Monitoring
The study design includes systematic collection of biological samples and analysis using multiple high-dimensional technologies (such as single-cell RNA/TCR sequencing, multiplex immunofluorescence [MIF], whole-exome sequencing [WES], cell-free DNA [cfDNA] sequencing, and cytokine profile analysis), which provides a detailed map of local tumor and systemic immune responses.
3. Revelation of Potential Immune Response Mechanisms
It was confirmed that the combination therapy can induce increased infiltration of CD4+ T cells into tumors, and this effect is associated with the CCL21 secretion level of DC vaccines.
It was found that patients with stable disease (SD) showed increased intratumoral TCR clonal diversity, and the newly emerged T cell clones exhibited stronger proliferative capacity.
Systemic immune characteristics related to clinical outcomes were identified (e.g., baseline CXCL8 level, dynamic changes of cytokines such as IL-3 and XCL1 during treatment).
Immunosuppressive myeloid cell signals (e.g., SPP1 pathway) were observed in the tumor microenvironment, suggesting potential mechanisms of drug resistance.
4. Clear Positioning of the Therapeutic Status Quo
The study enrolled mostly patients who had previously failed immune checkpoint inhibitor (ICI) treatment, and clarified the exploratory value of this therapy in such refractory populations. Although the objective response rate (ORR) was low, a certain disease control rate (DCR) and regression of intracranial metastases in individual cases were still observed, indicating potential biological activity of the therapy.

Major concerns:
1. In the protocol, the CT imaging was done at patient screening, day 63, and every 3 months following DC injection until progression of disease or withdrawal from study. Isn't the interval too long for patients receiving later-line treatment? Even in the first-line treatment setting, the first efficacy assessment is usually performed on Day 42 in other trials.
2. Citations of supplementary figures have some problems, as some figures did not appear in the supplementary material. Please screen all the supplementary figures and the citations in the manuscript. "This subset showed greater similarity between novel and pre-existing clones, but there was still relative overrepresentation of naive/memory and cytotoxic CD4+ cells as well as Tregs (Supplementary Fig. 4A)."
"Differentially expressed gene (DEG) analysis confirmed that novel clones were enriched in memory markers such as IL7R

and ribosomal genes, which are typical in scRNA-seq of naive/memory CD4+ cells (Fig. 3G, Supplementary Fig. 4B-C, Table 3). There were several genes linked to clonal expansion, including JUNB and FXYD5, and genes related to CD4+ cell activation and Treg development, such as ICOS and CTLA4. There was de-enrichment of genes related to cytotoxic and terminal effector T cell phenotypes, including GZMA, GZMB, CCL5, and NKG7. Gene ontology analysis confirmed enrichment for protein synthesis processes in novel clones as well as de-enrichment for signaling- and cytotoxicity-related processes (Fig. 3H). When comparing cluster distribution of these clones based on clinical response, SD patients were found to have greater proportion of pre-existing clones in the GZMB+ CD8+ cluster at baseline (Supplementary Fig. 4D). There were, however, no differences between PD and SD groups in regard to the cluster distribution of novel versus pre-existing clones (Supplementary Fig. 4E). "
"There was significant relative reduction of CCL20, IL-10, and TGFa in the SD group with opposing trends in the PD group (Supplementary Fig. 5A-E)."
3. No objective response was observed in this study (objective response rate, ORR = 0), with only disease stabilization achieved. The core reasons for the limited efficacy require further in-depth discussion. It is recommended to supplement the comparative analysis with historical data, including the ORR (approximately 8%) and disease control rate (DCR) in patients with similar drug-resistant non-small cell lung cancer (NSCLC), so as to clarify whether the combination therapy has advantages in terms of immune response or survival benefits. Meanwhile, combined with mechanisms such as poor persistence of T cell clones and immunosuppression in the tumor microenvironment, explanations should be provided for why the immune response did not translate into objective tumor regression.
4. The study found that many newly emerged T cell clones failed to persist, and it is hypothesized that these clones may be non-tumor-specific "bystander" T cells. While this is a plausible explanation, direct evidence supporting it is lacking. It is therefore recommended that the discussion section be expanded to include considerations on how to distinguish tumor-reactive from non-reactive T cell clones using existing or future technologies—such as the combination of tumor neoantigen prediction and TCR clone tracking.
5. The TCR diversity in the periphery is inconsistent with that in the tumor, and the mechanistic explanation for this discrepancy is insufficient.
6. Please discuss the future directions for the improvement of CCL21-DCs in this study; please also discuss the feasibility of CXCL9/10-engineered DCs for the treatment of the study's enrolled population.
Minor concerns:
1. A patient has a maximum disease control time of 12 months. After that, was the patient censored or did the disease progress?
2. Why did two patients need to be replaced? As stated in the manuscript: "Per protocol, six patients needed to be treated at the selected dose and two patients needed to be replaced."
3. Just out of curiosity, what is the approximate cost of DC vaccine for one patient in US dollars? Are there any mature marketed products, and what are their market prices?

Reviewer #2

(Remarks to the Author)
AUTHORS:

Summary of the manuscript
The manuscript by Oh et al reports the results of a phase I trial of an autologous monocyte-derived dendritic cell (DC) vaccine transduced ex vivo with a CCL21-expressing adenoviral vector (AdCCL21). The vaccine was administered intratumorally in patients with advanced NSCLC who had progressed on standard therapies, including patients with EGFR-driven tumors after appropriate targeted therapy.

The DC vaccine was injected directly into a lung tumor either bronchoscopically or by CT-guided transthoracic injection on days 0, 21, and 42. At each time point, patients received four injections per lesion, one into each quadrant of the tumor, and simultaneous core biopsies of the injected lesion were obtained. In parallel, all patients received pembrolizumab. Four patients were treated with fresh DC vaccine, whereas subsequent patients received cryopreserved DCs, after the authors had confirmed minimal impact of a freeze thaw cycle on DC phenotype. Dose escalation was performed in two cohorts (1×10^7 and 3×10^7 cells per injection), and 3×10^7 cells was selected for expansion.

Clinically, no objective radiologic responses were observed. Seven patients achieved stable disease (SD) by RECIST 1.1, the majority lasting less than 6 months, with one patient maintaining SD for 12 months. The regimen was generally well tolerated: treatment-related AEs occurred in 4 patients (17%), including one grade 1 flank pain related to the IT injection and three immune-related events, among them one grade 3 immune-related diabetes mellitus.

The authors performed an extensive set of correlative immune analyses on serial tumor biopsies and blood samples, comparing patients with SD versus progressive disease (PD). They report:
1. A trend toward decreased mutant-allele tumor heterogeneity (MATH scores) over time in tumors from SD patients, but not PD patients, which they interpret as evidence of immunoediting.
2. scRNA/TCR-seq on tumor samples from 17 patients, showing that intratumoral TCR diversity increased over time in SD tumors but not PD tumors, driven largely by three patients (one with intracranial response, one with more than 12 months SD, and another with SD -no more data about this case-).
3. Multiplex immunofluorescence showing an initial increase in intratumoral $CD4^+$ T-cell infiltration (but not $CD8^+$) after treatment; however, in several patients this increase at day 21 waned by day 42, resulting in no significant overall trend across all time points and no clear difference between SD and PD. The authors therefore argue that T-cell infiltration alone

does not explain the association between TCR diversity and clinical outcome.
4. At the clonal level, they show that most novel post-treatment T-cell clones in the tumor are CD4⁺, particularly naive/memory CD4⁺ cells expressing CCR7, consistent with recruitment via CCL21. Even after restricting analyses to larger clones (≥0.2% of the intratumoral TCR repertoire), novel clones remain enriched for CD4 naive/memory, CD4 cytotoxic, and Treg phenotypes. Differential gene expression analyses further demonstrate that these novel intratumoral clones display a naive/memory CD4⁺ transcriptional profile (IL7R, ribosomal genes), with signatures of early clonal expansion (JUNB, FXYD5) and activation/regulatory markers (ICOS, CTLA4), but are depleted of cytotoxic effector genes (GZMA, GZMB, CCL5, NKG7). Gene ontology analyses confirm enrichment for protein synthesis and de-enrichment of cytotoxic/signaling pathways. Clinically, patients with SD have a higher baseline proportion of pre-existing GZMB⁺ CD8⁺ clones, but the distribution of novel versus pre-existing clones after therapy does not differ between SD and PD. Temporal analyses show that many novel clones appearing on day 21 are no longer detectable at day 42. Among persistent novel clones, there is on average a 5.2-fold clonal expansion, more pronounced in SD than in PD (7.1-fold vs 4.6-fold, p=0.0001). In SD patients, persistent clones expand particularly within CD4 naive/memory, CD4 cytotoxic, Treg, and CD8 GZMB⁺ clusters, whereas PD tumors show a trend toward contraction, notably a reduction in CD8 GNLY⁺ clones.
5. The myeloid compartment is dominated by SPP1⁺ and IL1B⁺ macrophages, expressing additional pro-tumor markers such as MARCO and TREM2, with SPP1-mediated signaling being the most frequent macrophage–T-cell interaction. In contrast, the CXCL9/10–CXCR3 axis, associated with favorable ICI responses, is infrequently observed. Thus, the vaccine appears to recruit T cells into a persistently immunosuppressive myeloid microenvironment that is not effectively reprogrammed.
6. A positive correlation between CCL21 production by individual DC vaccine batches and increases in intratumoral T-cell infiltration after injection of those batches, supporting the intended chemoattractant function of the vaccine. The lack of increase in Ki-67 in CD4⁺ or CD8⁺ T cells suggests that enhanced CD4⁺ infiltration is driven by recruitment rather than in situ proliferation.
7. In blood, cfDNA and liquid biopsy analyses are relatively uninformative; the authors provide cfDNA fraction but do not detail variant allele frequency (VAF) changes in driver mutations. scRNA-seq of PBMCs at multiple time points (days 0, 21, 42, 63) reveals no major shifts in peripheral immune cell subsets in relation to treatment or outcome. However, MHC-II–related genes become progressively upregulated in PBMCs from SD, but not PD, patients, suggesting enhanced systemic antigen-presenting capacity.
8. Bulk TCR-seq of PBMCs shows no significant systemic changes in TCR diversity between SD and PD patients, in contrast with the dynamic intratumoral repertoire. Approximately 46% of novel intratumoral TCR clones are detectable in peripheral blood, and over one-third are already present at baseline, indicating that many “novel” tumor clones likely reflect recruited circulating T cells rather than de novo intratumoral generation. When restricting to clones absent at baseline in both tumor and blood, only 16% are ever detected in peripheral blood, and these show declining CD4⁺ fractions over time, suggesting poor systemic persistence of locally generated clones. A notable exception is a patient with local stability of the injected lesion and systemic PD, in whom a PD-1⁺CD103⁺CD39⁺ exhausted, tumor-specific T-cell clone expands intratumorally post-therapy, transiently appears in the peripheral TCR repertoire, and then disappears.
Overall, the study concludes that, while the CCL21-DC vaccine plus pembrolizumab is well tolerated and can have immune activity, particularly by recruiting CD4⁺ naive/memory T cells, the clinical efficacy is poor, and immune activation is transient and constrained by persistent immunosuppressive myeloid programs and limited tumor specificity of the T-cell response.

Overall assessment
This is a technically and methodologically ambitious phase I trial. The authors go well beyond conventional DC vaccination, combining an engineered CCL21-transduced DC product with intratumoral delivery via bronchoscopy or CT-guided injection, serial tumor biopsies, and an extensive battery of correlative assays, including WES, scRNA/TCR-seq, cfDNA, and cytokine profiling. The logistical and technical effort is substantial and commendable.

However, from a clinical standpoint, this is essentially a negative trial: although it is well tolerated, there are no objective responses and almost all SD events are short-lived (less than 6 months, with a single 12-month SD). As a consequence, the immunologic findings are difficult to clearly attribute to the vaccine, as opposed to natural history in a heterogeneous, heavily pretreated population. Many of the changes described (e.g., transient CD4⁺ infiltration, increased TCR diversity, transient generation of novel clones) illustrate challenges of cancer vaccines, namely, the difficulty of turning initial immune activation into durable, tumor-specific, cytotoxic immunity.

That said, some observations are indeed of interest for the field, especially the detailed clonal analyses and the characterization of an immunosuppressive SPP1⁺/IL1B⁺ macrophage niche that persists despite vaccination and the correlation of immune cell clones in tumor and in blood.

I believe the manuscript can be suitable for publication if the authors more clearly acknowledge the negative clinical nature of the study in the abstract, avoid overinterpreting correlative data, and better explore alternative definitions of clinical benefit for more accurate translational analyses.

Major comments

1. I recommend explicitly stating in the abstract that the clinical efficacy of this study was limited. While the authors clearly discuss the negative clinical results in the Discussion section, this is not fully reflected in the abstract. In addition, the concluding statement that the regimen “can promote antitumor responses” should be revised to clarify that this trial did not demonstrate meaningful clinical activity or clear vaccine-attributable antitumor immune responses.

2. Clinical efficacy and definition of benefit (SD as endpoint):
The clinical results are negative: no objective responses, short SD in most cases, and only one patient with SD lasting 12 months. It was a heterogeneous, heavily pretreated NSCLC population—including EGFR-driven tumors that are resistant to immunotherapy, and different to other NSCLC patients.

SD by RECIST 1.1, especially when less than six months, is a weak indicator of antitumoral efficacy as it can reflect natural history of the disease.
I strongly recommend the authors re-examine clinical benefit using different criteria, for example: incorporating ctDNA dynamics (e.g., driver mutation VAF reduction rather than cfDNA fraction alone) to identify patients with molecular response. As currently presented, it is difficult to link the extensive immunologic data to a vaccine related clinical effect.

3. Dose escalation rationale for DC vaccination:
The manuscript describes a formal dose-escalation phase up to 3×10^7 DCs per injection, despite decades of experience suggesting that DC vaccines are generally safe and unlikely to reach a conventional MTD. Please justify more clearly why a 3+3 dose escalation was necessary in this context and how the chosen doses relate to prior DC- or CCL21-DC–based trials.

4. Details on injected lesions and local responses:
The study design relies critically on intratumoral injection. However, it remains unclear which patients and which lesions (location) were injected and if they were injected using bronchoscopy or CT-guided. Was the same lesion injected at each time point?

It is also unclear whether the local responses of injected lesions had volume reduction or durable stabilization, independent of systemic progression. The text mentions at least one patient with stability of the CCL21-DC injected lesion but systemic PD, and a brain metastasis with apparent radiologic resolution. These cases deserve a clearer, more structured description (previous treatments, molecular subtype, lesion size, measurability, follow-up).
A per-lesion analysis (injected vs non-injected) would significantly strengthen the argument for a local vaccine effect, even in the absence of systemic responses.

Was there any pathological response? Including data of pathological response and % of tumor cells in each rebiopsy would be helpful.

5. Brain metastasis response:
Supplementary Fig. 1C illustrates an "untreated brain metastasis" that appears to resolve after therapy. However, the lesion is small, less than 1 cm. I suggest the authors provide more detailed clinical information to support a true intracranial CR. Also, clarify why this patient did not receive previous radiotherapy of the brain lesion (inclusion criteria state that brain metastases should be previously treated with radiotherapy).

6. Attribution of immunologic changes to the vaccine versus natural history:
The manuscript frequently implies that observed changes (e.g., increased $CD4^+$ infiltration, emergence of novel intratumoral clones, increased TCR diversity, MHC-II upregulation in PBMCs) are attributable to the CCL21-DC vaccine. However, in a single-arm, small-cohort trial with a highly heterogeneous population and no control arm, it is very difficult to separate vaccine effects from the behavior of indolent tumors or from pembrolizumab alone. The authors should explicitly acknowledge this limitation and be more cautious in assigning causality. In particular, the observation that SD patients often had more favorable baseline features suggests that intrinsic biology, rather than the vaccine, may largely drive outcomes.

7. Interpretation of T-cell clonality and dominance of $CD4^+$ naive/memory recruitment:
The TCR and scRNA-seq data are a central strength of the manuscript. They show clearly the emergence of novel intratumoral TCR clones, but most do not persist. Only about half of novel clones are detectable in peripheral blood, and clones likely generated de novo within the TME have minimal and transient peripheral representation. Novel clones are predominantly $CD4^+$ naive/memory, enriched for IL7R and ribosomal genes, with limited cytotoxic effector programs and frequent expression of regulatory/activation markers (ICOS, CTLA4). SD patients have a higher baseline burden of pre-existing $GZMB^+$ $CD8^+$ effector clones, whereas the distribution of novel versus pre-existing clones post-treatment does not differ between SD and PD.
Collectively, these findings strongly support the notion that CCL21-DC vaccination could recruit $CD4^+$ naive/memory and regulatory phenotypes to a persistently immunosuppressive TME, with limited induction of durable, tumor-specific $CD8^+$ cytotoxic responses. This aligns with prior literature showing that robust and sustained antitumor immunity generally requires high-avidity, antigen-specific $CD8^+$ T cells.

I would encourage the authors to more explicitly frame this as a key mechanistic conclusion and to discuss potential next steps (e.g., strategies to bias recruitment toward $CD8^+$ cytotoxic clones, modify the cytokine payload, or target myeloid suppressive pathways).

8. Myeloid compartment and lack of microenvironmental reprogramming:
The identification of $SPP1^+$ and $IL1B^+$ macrophages expressing MARCO and TREM2, and the dominance of SPP1-mediated macrophage–T-cell signaling, is compelling and consistent with recent literature on immunosuppressive myeloid niches. This pattern offers a plausible explanation for the limited durability of the vaccine-induced T-cell activation. However, the authors stop short of proposing how their therapy could be rationally combined with myeloid-targeting agents or altered

to reprogram this macrophage compartment. Adding a more explicit discussion of potential combinatorial strategies (e.g., MARCO/TREM2 blockade, SPP1 axis targeting) would increase the translational value of the work.

9. Heterogeneity of the patient population and molecular characterization:
The cohort includes EGFR-mutated and ALK-positive tumors and presumably other molecular subsets, but molecular profiles are not clearly presented in the main Table of baseline characteristics. I recommend the authors add more granular molecular data (e.g., EGFR, ALK, KRAS, including mutations in genes that usually preclude immune response as STK11, KEAP1, etc.) and discuss whether any of these features are associated with the limited clinical benefit observed. The case with 12 months of SD should be described in more detail (tumor genotype, prior treatments, tumor burden), as it is the single most clinically relevant outcome.

Minor comments

1. Ethnicity/race variable spelling:
In Table 1, the category “None-Hispanic of Latino” is incorrect. This should be corrected to standard terminology such as “Non-Hispanic or Latino” or “Not Hispanic or Latino”.

2. MTD wording:
The text currently states that “19 patients received at least 2 injections at the maximum tolerated dose (MTD)” although the MTD was not reached. This should be changed to “highest planned dose” or “selected dose for expansion” to avoid confusion.

3. Accrual period:
The accrual/reference period appears to extend from July 2019 to January 2024 for only 23 patients. While this may be realistic given the complexity of intratumoral vaccination, it would be helpful if the authors comment on the reasons for the relatively slow accrual.

4. Supplementary AEs table:
It would be helpful if the supplementary AEs table included all-grade AEs in more detail, including attribution (DC vs pembrolizumab vs DC vaccine vs injection), given the novelty of the intratumoral procedure.

Additional comments:

The study is of great interest despite the scarcity of clinical responses. There is a multitude of factors that could impair the benefit of dendritic vaccination in the current study. It could be of interest if the authors could, or have already, determined signatures for predicting the response to dendritic cell vaccines and immunotherapy. Any determination of the tumor microenvironment—whether a hot, T-cell–inflamed environment with high PD-L1 or a cold, T-cell–excluded tumor microenvironment—would be of benefit.

STK11 and KEAP1 co-mutations negate the effect of immunotherapy. Although the number of patients is small, the NGS information is of interest.

Among the markers examined, bear in mind the expression of DNASE1L3 in dendritic cells, which promotes CD8+ T-cell functon(see Chen et al., DNASE1L3-expressing dendritic cells promote CD8+ T cell function and anti-PD-(L)1 therapy efficacy by degrading neutrophil extracellular traps, Cancer Cell, 2025).

Ectonucleotide pyrophosphatase/phosphodiesterase 1 (ENPP1), a hydrolase of extracellular cGAMP, causes immune suppression and can jeopardize the immunotherapeutic approach. Immunotherapy resistance often occurs through overexpression of COL3A1 and COL6A1. M2 polarization, driven by disturbances in cGAS-STING signaling or alterations in dsRNA resolution, often driven by elevation of protein arginine methyltransferase 1 (PRMT1), are some of the markers that should be taken into account.

I suggest re-addressing the discussion, trimming it a bit, and re-focusing on the limitations you have cited. It is of interest but somewhat reiterative and therefore requires more concretion. Keep the above comments in consideration.

Concluding remarks
In summary, this manuscript presents a sophisticated and carefully executed immunologic study of a clinically negative trial. The major value of the work lies in its multi-omics dissection of the limits of DC-based vaccination in a highly immunotherapy-resistant setting, rather than in demonstrating clinical efficacy. With more cautious interpretation, clearer presentation of the clinical context and limitations, and additional refinement of the analyses and discussion, as suggested above, the manuscript could make a useful contribution to the design of next-generation, microenvironment-aware DC vaccine strategies.

Reviewer #3

(Remarks to the Author)

In this manuscript, Oh and colleagues report results from a phase I trial combining intratumoral injection of a CCL21-expressing dendritic cell (CCL21-DC) vaccine with pembrolizumab in patients with advanced NSCLC. Although the combination therapy was well tolerated, there were no objective responses observed. The best response was stable disease, which was observed in 36.8% of patients. Analyses of longitudinal biopsies revealed a therapy-associated increase in tumor infiltration by CD4+ T cells and novel T cell clones. Disease stability was associated with increased T cell receptor (TCR) diversity and greater expansion of novel clones following therapy. However, many novel clones failed to persist over time, while immunosuppressive myeloid signaling signatures were identified across time points. The authors concluded that “CCL21-DC vaccination combined with pembrolizumab can promote antitumor immune responses”, but efficacy may be constrained due to limited tumor specificity of the T cell response and an inability to fully reprogram the tumor microenvironment.

I commend the authors on a well-designed phase I trial evaluating a CCL21-DC vaccine combined with PD-1 ICB (pembrolizumab) in advanced, pretreated NSCLC patients. I also commend them on overcoming the logistical hurdles facing obtaining longitudinal pre- and on-therapy tissue samples for their correlative studies. The authors report (1) increased CD4 infiltration and (2) increased intratumoral TCR diversity in patients with stable disease. Unfortunately, there was minimal clinical efficacy and no real objective responses.

I have a few comments:

1- The authors report greater expansion of novel clones after therapy. However the majority of expanded clones after treatment were $CD4^+$ clones ($CCR7^+$ naïve/memory), and there is no phenotypic or functional validation. The data seem consistent with bystander recruitment with the vaccine, rather than tumor-specific priming, and although this is acknowledged in the discussion, the framing in the abstract and results still implies antitumor relevance. For example, “CCL21-DC vaccination with pembrolizumab can promote antitumor immune responses” is stated in the Aabstract, although there is no objective response, limited CD8 engagement, and no validated tumor specificity. What appears to be observed is that the combination therapy induces transient clonal perturbations in the TME, although evidence for antitumor immunity remains limited.

2- The TCR diversity claim need to carefully consider bystander effects, as the authors repeatedly state that increased TCR diversity is favorable because it is mostly found in patients with stable disease. However, as noted above, the recruitment is largely of naïve-like CD4 cells that do not have demonstrated antitumor functon(or at least are not shown to have antitumor function by the authors). These data may also be confounded by repeated spatial sampling. The authors use a 0.2% clonal frequency cutoff to show clonal expansion, but this threshold seems arbitrary and requires further explanation. Although several groups using TCR/scRNA sequencing to identify tumor-relevant clones provide strict thresholds (e.g., >5 or >10 cells per clone to define expansion), remove bystanders using published viral-associated TCR databases (e.g., VDJdb), and validate findings with published transcriptional signatures of tumor reactivity (Caushi et al., Lowery et al. Nature 596.7870 (2021): 126-132), such approaches were not applied here. Further computational analysis of this type would greatly strengthen the validity of the TCR diversity and cell-state results.

3- Reprogramming of the suppressive TME is also explored by the authors, showing enrichment of suppressive macrophages with MARCO and TREM2 expression, which is important given preclinical evidence of CCL21-DC–mediated reprogramming that was not observed in this trial. CXCL9/10 signaling was also observed in only three patients with stable disease. In the discussion, this finding may be overstated as promoting an inflammatory state in stable disease patients, given the limited number of cases.

4- MIF analysis showed increased CD4 T-cell infiltration. A suggestion would be to assess spatial features within the MIF data, if possible, including proximity to tumor cells, immune exclusion, and relationships with macrophages, given the importance of immunosuppressive macrophages highlighted by the authors.

In conclusion, this study provides value in characterizing the immunologic consequences of intratumoral CCL21-DC vaccination in combination with pembrolizumab in advanced-stage NSCLC. The data demonstrate immune cell recruitment and transient clonal dynamics but fails to show tumor-specific immunity or meaningful reprogramming of the TME. Several conclusions should be restated to reflect these findings more accurately.

Reviewer #4

(Remarks to the Author)

This is a phase I trial evaluating a vaccine composed of autologous dendritic cells (DCs) engineered to overexpress the chemokine CCL-21 (via an adenoviral vector), administered in combination with the anti-PD-1 antibody pembrolizumab. CCL-21 promotes the colocalization of T cells and DCs, while pembrolizumab is an immune checkpoint inhibitor; together, these agents may enhance antitumor immunity within the tumor microenvironment. The rationale for combining these therapies also stems from a prior phase I trial of the CCL-21 DC vaccine, in which PD-L1 (the ligand for PD-1) overexpression was observed.

The clinical trial uses a two-step design with a modified dose escalation (applied only to the CCL-21 DC vaccine; pembrolizumab was administered at a fixed dose) following a 3+3 scheme, followed by a dose-expansion cohort. In the dose-escalation phase, the primary endpoint is the Maximum Tolerated Dose (MTD), defined as the dose with unacceptable toxicity in strictly fewer than 33% of treated patients. In the dose-expansion phase, the primary endpoint is the overall response rate according to RECIST v1.1. Several translational analyses were performed in parallel using tumor biopsies and blood samples, and these constitute the majority of the results presented in the article.

No dose-limiting toxicities were observed, and therefore the MTD was not reached. In terms of efficacy, no patient experienced an objective response to treatment.

As a statistician, I focus my comments on the clinical trial design and general considerations related to the statistical analyses in this study.

Transparency: The protocol and clinical trial registration number are provided, and there were no deviations from the

submitted protocol. I have no concerns on this point.

Design: This is a standard statistical design for a phase I study, following a 3+3 approach. However, the cohort size at each dose level was not consistently three patients as usual; in some cases it was six, while still adhering to the targeted 33% unacceptable toxicity rate. The sample size justification for the dose-expansion cohort, based on a two-sided binomial proportion test, is reproducible.
Comments:
- Could you provide the median follow-up time for patients in the study? Some censoring appears to have occurred early.
- Could you also specify the number of patients enrolled at each dose-escalation stage in the flow diagram and give the reasons for exclusion (+replacement if applicable)? According to the protocol, six patients should have been treated at the maximum dose level, whereas the text refers to eight patients. It is also unclear how 19 patients were included in the efficacy set.

General considerations: The sample size in this study is very small (as the authors acknowledge in the discussion). Given this, it is unlikely that assumptions for parametric tests such as Student's t-tests (Figures 4 and 6) are satisfied such as normality. I recommend using non-parametric alternatives such as the Wilcoxon rank-sum test. n addition, with respect to the translational analyses, the cohort size is so limited that it is highly unlikely that robust or reliable inferences can be drawn from these data.

Despite these limitations, I found it valuable that the authors explored mechanistic explanations for why the combination of the DC vaccine with pembrolizumab failed to elicit a clinical response. Such investigations might help identify ineffective candidates early and prevent unnecessary research burden.

Reviewer #5

(Remarks to the Author)
I co-reviewed this manuscript with one of the reviewers who provided the listed reports. This is part of the Nature Communications initiative to facilitate training in peer review and to provide appropriate recognition for Early Career Researchers who co-review manuscripts.

Version 1:

Reviewer comments:

Reviewer #1

(Remarks to the Author)
After careful review, I am pleased to recommend acceptance of this manuscript for publication.

Reviewer #2

(Remarks to the Author)
The authors have adequately replied to all my queries. I now feel that the manuscript can proceed to publication if all other reviewers are in agreement.

Reviewer #3

(Remarks to the Author)
The authors have adequately addressed all concerns raised in the prior review. They have appropriately reframed their conclusions to remove overstatements regarding antitumor immune specificity, tempered claims around TCR diversity, and clarified the threshold used for defining clonal expansion. The addition of VDJdb cross-referencing and tumor-reactive transcriptional signatures, including those from Lowery et al. and Caushi et al., strengthens the TCR analysis, and the new spatial neighborhood analysis using MIF data adds meaningful context to the T cell infiltration findings. Interpretations of the CXCL9/10 signaling data have been appropriately qualified given the small sample size. Overall, the revisions reflect a more accurate and measured characterization of the trial findings, and the manuscript is suitable for acceptance.

Reviewer #4

(Remarks to the Author)
I read the revised version of the manuscript. The authors answered all my previous comments, I have no further remark from the statistical perspective.

Open Access This Peer Review File is licensed under a Creative Commons Attribution 4.0 International License, which permits use, sharing, adaptation, distribution and reproduction in any medium or format, as long as you give appropriate credit to the original author(s) and the source, provide a link to the Creative Commons license, and indicate if changes were made.
In cases where reviewers are anonymous, credit should be given to 'Anonymous Referee' and the source.
The images or other third party material in this Peer Review File are included in the article's Creative Commons license, unless indicated otherwise in a credit line to the material. If material is not included in the article's Creative Commons license and your intended use is not permitted by statutory regulation or exceeds the permitted use, you will need to obtain permission directly from the copyright holder.
To view a copy of this license, visit https://creativecommons.org/licenses/by/4.0/

RESPONSE TO REVIEWERS

Reviewer #1 (Remarks to the Author):

This study is a rigorously designed and in-depth Phase I clinical trial, which for the first time evaluated the safety, preliminary efficacy, and immunological effects of intratumoral injection of CCL21 gene-modified dendritic cell (DC) vaccine combined with pembrolizumab in patients with advanced non-small cell lung cancer (NSCLC) in humans. Its key highlights are as follows:

1. Innovative Combination Therapy Strategy
The study aims to recruit T cells into the tumor microenvironment (TME) via DC vaccines expressing the chemokine CCL21, and combine this with PD-1 inhibitor to block immunosuppressive signals. Theoretically, this strategy is expected to synergistically enhance the anti-tumor immune response, especially in patients who have developed resistance to previous immunotherapy.
2. Comprehensive and In-depth Immune Monitoring
The study design includes systematic collection of biological samples and analysis using multiple high-dimensional technologies (such as single-cell RNA/TCR sequencing, multiplex immunofluorescence [MIF], whole-exome sequencing [WES], cell-free DNA [cfDNA] sequencing, and cytokine profile analysis), which provides a detailed map of local tumor and systemic immune responses.
3. Revelation of Potential Immune Response Mechanisms
It was confirmed that the combination therapy can induce increased infiltration of CD4+ T cells into tumors, and this effect is associated with the CCL21 secretion level of DC vaccines. It was found that patients with stable disease (SD) showed increased intratumoral TCR clonal diversity, and the newly emerged T cell clones exhibited stronger proliferative capacity. Systemic immune characteristics related to clinical outcomes were identified (e.g., baseline CXCL8 level, dynamic changes of cytokines such as IL-3 and XCL1 during treatment). Immunosuppressive myeloid cell signals (e.g., SPP1 pathway) were observed in the tumor microenvironment, suggesting potential mechanisms of drug resistance.
4. Clear Positioning of the Therapeutic Status Quo
The study enrolled mostly patients who had previously failed immune checkpoint inhibitor (ICI) treatment, and clarified the exploratory value of this therapy in such refractory populations. Although the objective response rate (ORR) was low, a certain disease control rate (DCR) and regression of intracranial metastases in individual cases were still observed, indicating potential biological activity of the therapy.

Major concerns:
1. In the protocol, the CT imaging was done at patient screening, day 63, and every 3 months following DC injection until progression of disease or withdrawal from study. Isn't the interval too long for patients receiving later-line treatment? Even in the first-line treatment setting, the first efficacy assessment is usually performed on Day 42 in other trials.

We acknowledge that our scans occurred on the later side of typical intervals. In our experience, imaging in both trials and real-world practice is generally performed every 8-12 weeks, with perhaps a first assessment at 6-8 weeks. However, we felt it would be most appropriate to complete the DC vaccination schedule prior to restaging. For immunotherapies, there is also some concern that imaging too early may demonstrate pseudo-progression or not provide sufficient time for a robust immune response. We have detailed this rationale in the Methods section (page 19, paragraph 2). In regard to our reported findings, it is possible that more frequent imaging could have affected time-based metrics such as PFS, but this ultimately would not have changed the final conclusion of a negative trial by ORR or denotation of SD vs PD patients.

2. Citations of supplementary figures have some problems, as some figures did not appear in the supplementary material. Please screen all the supplementary figures and the citations in the manuscript. “This subset showed greater similarity between novel and pre-existing clones, but there was still relative overrepresentation of naive/memory and cytotoxic CD4+ cells as well as Tregs (Supplementary Fig. 4A).”
“Differentially expressed gene (DEG) analysis confirmed that novel clones were enriched in memory markers such as IL7R and ribosomal genes, which are typical in scRNA-seq of naive/memory CD4+ cells (Fig. 3G, Supplementary Fig. 4B-C, Table 3). There were several genes linked to clonal expansion, including JUNB and FXYD5, and genes related to CD4+ cell activation and Treg development, such as ICOS and CTLA4. There was de-enrichment of genes related to cytotoxic and terminal effector T cell phenotypes, including GZMA, GZMB, CCL5, and NKG7. Gene ontology analysis confirmed enrichment for protein synthesis processes in novel clones as well as de-enrichment for signaling- and cytotoxicity-related processes (Fig. 3H). When comparing cluster distribution of these clones based on clinical response, SD patients were found to have greater proportion of pre-existing clones in the GZMB+ CD8+ cluster at baseline (Supplementary Fig. 4D). There were, however, no differences between PD and SD groups in regard to the cluster distribution of novel versus pre-existing clones (Supplementary Fig. 4E).”
“There was significant relative reduction of CCL20, IL-10, and TGFa in the SD group with opposing trends in the PD group (Supplementary Fig. 5A-E).”

Thank you for pointing out these inconsistencies. We reviewed all figures and ensured that they are correctly ordered and referenced in the text.

3. No objective response was observed in this study (objective response rate, ORR = 0), with only disease stabilization achieved. The core reasons for the limited efficacy require further in-depth discussion. It is recommended to supplement the comparative analysis with historical data, including the ORR (approximately 8%) and disease control rate (DCR) in patients with similar drug-resistant non-small cell lung cancer (NSCLC), so as to clarify whether the combination therapy has advantages in terms of immune response or survival benefits. Meanwhile, combined with mechanisms such as poor persistence of T cell clones and immunosuppression in the tumor microenvironment, explanations should be provided for why the immune response did not translate into objective tumor regression.

We have provided a more detailed discussion of the trial results and context. We have included references for historical controls of immune checkpoint inhibition beyond initial progression (Discussion, page 13, paragraph 2). We now concretely state that there is no evidence for clinical advantage with the combination therapy. We also have significantly expanded on potential mechanistic reasons for the limited clinical benefit (Discussion, page 15, paragraph 1 and paragraph 3; and page 16, paragraph 2).

4. The study found that many newly emerged T cell clones failed to persist, and it is hypothesized that these clones may be non-tumor-specific "bystander" T cells. While this is a plausible explanation, direct evidence supporting it is lacking. It is therefore recommended that the discussion section be expanded to include considerations on how to distinguish tumor-reactive from non-reactive T cell clones using existing or future technologies—such as the combination of tumor neoantigen prediction and TCR clone tracking.

We agree that we have not validated the tumor specificity of the T cells directly against potential neoantigens. We have clarified this in the discussion and reviewed strategies for direct validation as a future direction (Discussion, page 17, paragraph 1).

5. The TCR diversity in the periphery is inconsistent with that in the tumor, and the mechanistic explanation for this discrepancy is insufficient.

We have expanded our proposed explanations (Discussion, page 16, paragraph 2), though acknowledge that direct mechanistic evidence is lacking in our study. Importantly, trafficking to local lymph nodes has been shown to be a key component of this process, but unfortunately we were unable to evaluate this due to tissue constraints.

6. Please discuss the future directions for the improvement of CCL21-DCs in this study; please also discuss the feasibility of CXCL9/10-engineered DCs for the treatment of the study's enrolled population.

We have expanded upon our discussion of the cytokine-modified DC vaccine platform, including for CXCL9/10 (Discussion, page 16, paragraph 1). We also discuss potential combination immunotherapies that may act synergistically with DC vaccines, including myeloid-targeting therapies given our data showing immunosuppressive myeloid signaling in many tumor samples.

Minor concerns:

1. A patient has a maximum disease control time of 12 months. After that, was the patient censored or did the disease progress?

We have clarified in the text that this patient was censored at 12 months per protocol, but they did not experience disease progression at that time (Results, page 5, paragraph 3).

2. Why did two patients need to be replaced? As stated in the manuscript: “Per protocol, six patients needed to be treated at the selected dose and two patients needed to be replaced.”

We apologize for the vagueness here: we have edited this sentence to clarify that these 2 patients did not receive all 3 DC injections, which was required by protocol to complete the dose escalation phase (Results, page 4, paragraph 3). One of these patients went off study due to clinical deterioration and the other stopped treatment due to an adverse event. We have better detailed exclusion events in an updated CONSORT flow diagram in Supplementary Fig. 1.

3. Just out of curiosity, what is the approximate cost of DC vaccine for one patient in US dollars? Are there any mature marketed products, and what are their market prices?

Within the UCLA GMP laboratory, the cost for vaccine preparation and preservation for each patient (all 3 injections) was approximately $15,000. This number does not include personnel costs. The only approved dendritic cell therapy on the market is sipuleucel-T for prostate cancer, though this is not an intratumoral therapy. The price tag for a full three-dose treatment course at time of market entry in 2010 was $93,000.

<u>Reviewer #2 (Remarks to the Author):</u>

Summary of the manuscript
The manuscript by Oh et al reports the results of a phase I trial of an autologous monocyte-derived dendritic cell (DC) vaccine transduced ex vivo with a CCL21-expressing adenoviral vector (AdCCL21). The vaccine was administered intratumorally in patients with advanced NSCLC who had progressed on standard therapies, including patients with EGFR-driven tumors after appropriate targeted therapy.

The DC vaccine was injected directly into a lung tumor either bronchoscopically or by CT-guided transthoracic injection on days 0, 21, and 42. At each time point, patients received four injections per lesion, one into each quadrant of the tumor, and simultaneous core biopsies of the injected lesion were obtained. In parallel, all patients received pembrolizumab. Four patients were treated with fresh DC vaccine, whereas subsequent patients received cryopreserved DCs, after the authors had confirmed minimal impact of a freeze thaw cycle on DC phenotype. Dose escalation was performed in two cohorts (1×10^7 and 3×10^7 cells per injection), and 3×10^7 cells was selected for expansion.

Clinically, no objective radiologic responses were observed. Seven patients achieved stable disease (SD) by RECIST 1.1, the majority lasting less than 6 months, with one patient maintaining SD for 12 months. The regimen was generally well tolerated: treatment-related

AEs occurred in 4 patients (17%), including one grade 1 flank pain related to the IT injection and three immune-related events, among them one grade 3 immune-related diabetes mellitus.

The authors performed an extensive set of correlative immune analyses on serial tumor biopsies and blood samples, comparing patients with SD versus progressive disease (PD). They report:

1. A trend toward decreased mutant-allele tumor heterogeneity (MATH scores) over time in tumors from SD patients, but not PD patients, which they interpret as evidence of immunoediting.
2. scRNA/TCR-seq on tumor samples from 17 patients, showing that intratumoral TCR diversity increased over time in SD tumors but not PD tumors, driven largely by three patients (one with intracranial response, one with more than 12 months SD, and another with SD -no more data about this case-).
3. Multiplex immunofluorescence showing an initial increase in intratumoral $CD4^+$ T-cell infiltration (but not $CD8^+$) after treatment; however, in several patients this increase at day 21 waned by day 42, resulting in no significant overall trend across all time points and no clear difference between SD and PD. The authors therefore argue that T-cell infiltration alone does not explain the association between TCR diversity and clinical outcome.
4. At the clonal level, they show that most novel post-treatment T-cell clones in the tumor are $CD4^+$, particularly naive/memory $CD4^+$ cells expressing CCR7, consistent with recruitment via CCL21. Even after restricting analyses to larger clones (≥0.2% of the intratumoral TCR repertoire), novel clones remain enriched for CD4 naive/memory, CD4 cytotoxic, and Treg phenotypes. Differential gene expression analyses further demonstrate that these novel intratumoral clones display a naive/memory $CD4^+$ transcriptional profile (IL7R, ribosomal genes), with signatures of early clonal expansion (JUNB, FXYD5) and activation/regulatory markers (ICOS, CTLA4), but are depleted of cytotoxic effector genes (GZMA, GZMB, CCL5, NKG7). Gene ontology analyses confirm enrichment for protein synthesis and de-enrichment of cytotoxic/signaling pathways. Clinically, patients with SD have a higher baseline proportion of pre-existing $GZMB^+$ $CD8^+$ clones, but the distribution of novel versus pre-existing clones after therapy does not differ between SD and PD. Temporal analyses show that many novel clones appearing on day 21 are no longer detectable at day 42. Among persistent novel clones, there is on average a 5.2-fold clonal expansion, more pronounced in SD than in PD (7.1-fold vs 4.6-fold, p=0.0001). In SD patients, persistent clones expand particularly within CD4 naive/memory, CD4 cytotoxic, Treg, and CD8 $GZMB^+$ clusters, whereas PD tumors show a trend toward contraction, notably a reduction in CD8 $GNLY^+$ clones.
5. The myeloid compartment is dominated by $SPP1^+$ and $IL1B^+$ macrophages, expressing additional pro-tumor markers such as MARCO and TREM2, with SPP1-mediated signaling being the most frequent macrophage–T-cell interaction. In contrast, the CXCL9/10–CXCR3 axis, associated with favorable ICI responses, is infrequently observed. Thus, the vaccine appears to recruit T cells into a persistently immunosuppressive myeloid microenvironment that is not effectively reprogrammed.

6. A positive correlation between CCL21 production by individual DC vaccine batches and increases in intratumoral T-cell infiltration after injection of those batches, supporting the intended chemoattractant function of the vaccine. The lack of increase in Ki-67 in $CD4^+$ or $CD8^+$ T cells suggests that enhanced $CD4^+$ infiltration is driven by recruitment rather than in situ proliferation.
7. In blood, cfDNA and liquid biopsy analyses are relatively uninformative; the authors provide cfDNA fraction but do not detail variant allele frequency (VAF) changes in driver mutations. scRNA-seq of PBMCs at multiple time points (days 0, 21, 42, 63) reveals no major shifts in peripheral immune cell subsets in relation to treatment or outcome. However, MHC-II–related genes become progressively upregulated in PBMCs from SD, but not PD, patients, suggesting enhanced systemic antigen-presenting capacity.
8. Bulk TCR-seq of PBMCs shows no significant systemic changes in TCR diversity between SD and PD patients, in contrast with the dynamic intratumoral repertoire. Approximately 46% of novel intratumoral TCR clones are detectable in peripheral blood, and over one-third are already present at baseline, indicating that many "novel" tumor clones likely reflect recruited circulating T cells rather than de novo intratumoral generation. When restricting to clones absent at baseline in both tumor and blood, only 16% are ever detected in peripheral blood, and these show declining $CD4^+$ fractions over time, suggesting poor systemic persistence of locally generated clones. A notable exception is a patient with local stability of the injected lesion and systemic PD, in whom a $PD\text{-}1^+CD103^+CD39^+$ exhausted, tumor-specific T-cell clone expands intratumorally post-therapy, transiently appears in the peripheral TCR repertoire, and then disappears.
Overall, the study concludes that, while the CCL21-DC vaccine plus pembrolizumab is well tolerated and can have immune activity, particularly by recruiting $CD4^+$ naive/memory T cells, the clinical efficacy is poor, and immune activation is transient and constrained by persistent immunosuppressive myeloid programs and limited tumor specificity of the T-cell response.

Overall assessment

This is a technically and methodologically ambitious phase I trial. The authors go well beyond conventional DC vaccination, combining an engineered CCL21-transduced DC product with intratumoral delivery via bronchoscopy or CT-guided injection, serial tumor biopsies, and an extensive battery of correlative assays, including WES, scRNA/TCR-seq, cfDNA, and cytokine profiling. The logistical and technical effort is substantial and commendable.

However, from a clinical standpoint, this is essentially a negative trial: although it is well tolerated, there are no objective responses and almost all SD events are short-lived (less than 6 months, with a single 12-month SD). As a consequence, the immunologic findings are difficult to clearly attribute to the vaccine, as opposed to natural history in a heterogeneous, heavily pretreated population. Many of the changes described (e.g., transient $CD4^+$ infiltration, increased TCR diversity, transient generation of novel clones) illustrate challenges of cancer vaccines, namely, the difficulty of turning initial immune activation into durable, tumor-specific, cytotoxic immunity.

That said, some observations are indeed of interest for the field, especially the detailed clonal analyses and the characterization of an immunosuppressive $SPP1^+$/$IL1B^+$ macrophage niche that persists despite vaccination and the correlation of immune cell clones in tumor and in blood.

I believe the manuscript can be suitable for publication if the authors more clearly acknowledge the negative clinical nature of the study in the abstract, avoid overinterpreting correlative data, and better explore alternative definitions of clinical benefit for more accurate translational analyses.

We thank the reviewer for their comprehensive assessment of the manuscript and appreciate their detailed feedback as expounded below.

Major comments

1. I recommend explicitly stating in the abstract that the clinical efficacy of this study was limited. While the authors clearly discuss the negative clinical results in the Discussion section, this is not fully reflected in the abstract. In addition, the concluding statement that the regimen "can promote antitumor responses" should be revised to clarify that this trial did not demonstrate meaningful clinical activity or clear vaccine-attributable antitumor immune responses.

We agree with the reviewer in being more explicit with the clinical implication of our study and have edited the abstract and other sections throughout the manuscript to reflect the limited clinical efficacy (for example, Discussion, page 14, paragraph 3 and page 15, paragraph 1).

2. Clinical efficacy and definition of benefit (SD as endpoint):
The clinical results are negative: no objective responses, short SD in most cases, and only one patient with SD lasting 12 months. It was a heterogeneous, heavily pretreated NSCLC population—including EGFR-driven tumors that are resistant to immunotherapy, and different to other NSCLC patients.

SD by RECIST 1.1, especially when less than six months, is a weak indicator of antitumoral efficacy as it can reflect natural history of the disease.
I strongly recommend the authors re-examine clinical benefit using different criteria, for example: incorporating ctDNA dynamics (e.g., driver mutation VAF reduction rather than cfDNA fraction alone) to identify patients with molecular response. As currently presented, it is difficult to link the extensive immunologic data to a vaccine related clinical effect.

We agree that it is difficult to parse out the relative contributions of underlying disease biology and short-term SD. We thought it was a good idea to use ctDNA as a proxy but were left with a very low sample number, as some patients did not have clear and/or

detectable driver mutations. Ultimately, we felt that rather than finding a different clinical readout, it was most appropriate to more clearly frame the data in the context of a negative trial that did not yield objective responses. We have acknowledged this limitation of using SD/PD denotations (Discussion, page 14, paragraph 1).

We have meanwhile expanded on clinical descriptions for cases of interest to provide greater context to select cases where we do believe there is more reasonable evidence of efficacy, which were also the cases with the starkest change in immune parameters such as TCR diversity (Results, page 5, paragraphs 3-4). We also tried to emphasize data that illustrate per-patient changes to establish the immune perturbations produced by the CCL21-DC vaccine, rather than overly focusing on SD vs PD differences.

3. Dose escalation rationale for DC vaccination:
The manuscript describes a formal dose-escalation phase up to 3×10^7 DCs per injection, despite decades of experience suggesting that DC vaccines are generally safe and unlikely to reach a conventional MTD. Please justify more clearly why a 3+3 dose escalation was necessary in this context and how the chosen doses relate to prior DC- or CCL21-DC–based trials.

The dose escalation was based on our prior phase I trial of the CCL21-DC vaccine; we used the 2 highest dose levels from that trial. Though the reviewer's question is very reasonable, we did feel that the novel combination of the DC vaccine with immune checkpoint inhibition could potentiate immune-related adverse effects. We have detailed this rationale in the manuscript (Methods, page 18, paragraph 1).

4. Details on injected lesions and local responses:
The study design relies critically on intratumoral injection. However, it remains unclear which patients and which lesions (location) were injected and if they were injected using bronchoscopy or CT-guided. Was the same lesion injected at each time point?

We have clarified in the manuscript that the same lesion was injected at each time point (Results, page 4, paragraph 2). We have also provided the frequency of bronchoscopic vs CT-guided injection and modified the swimmer's plot to denote which patients received each modality (Fig. 1).

It is also unclear whether the local responses of injected lesions had volume reduction or durable stabilization, independent of systemic progression. The text mentions at least one patient with stability of the CCL21-DC injected lesion but systemic PD, and a brain metastasis with apparent radiologic resolution. These cases deserve a clearer, more structured description (previous treatments, molecular subtype, lesion size, measurability, follow-up).
A per-lesion analysis (injected vs non-injected) would significantly strengthen the argument for a local vaccine effect, even in the absence of systemic responses.

We have significantly expanded our descriptions of the 2 cases of interest to provide greater clinical context (Results, page 5, paragraphs 3-4). In addition, we have provided waterfall plots depicting injected vs non-injected lesions (Supplementary Fig. 2). These data show that although there was overall greater stability among injected tumors, there were still no significant reductions in tumor size.

Was there any pathological response? Including data of pathological response and % of tumor cells in each rebiopsy would be helpful.

Due to the limited size of the biopsies and potential for sampling bias, we felt that a raw denotation of tumor percentage would not be insufficient to determine pathologic response, which typically is applied to resected samples. However, we did analyze our MIF data to assess changes in tumor cell metrics to indirectly evaluate the local response (Results, page 9, paragraph 1 and Supplementary Fig. 5E-H). Though there was an interesting trend showing decreased tumor density and proliferation in SD samples, these were not statistically significant.

5. Brain metastasis response:
Supplementary Fig. 1C illustrates an "untreated brain metastasis" that appears to resolve after therapy. However, the lesion is small, less than 1 cm. I suggest the authors provide more detailed clinical information to support a true intracranial CR. Also, clarify why this patient did not receive previous radiotherapy of the brain lesion (inclusion criteria state that brain metastases should be previously treated with radiotherapy).

The protocol specified that patients would be excluded for clinically active brain metastases, defined as "untreated and symptomatic." Due to it being asymptomatic and quite small (4 mm), this was not considered to be exclusionary. We have also included additional details on this case (Results, page 5, paragraph 4).

6. Attribution of immunologic changes to the vaccine versus natural history:
The manuscript frequently implies that observed changes (e.g., increased $CD4^+$ infiltration, emergence of novel intratumoral clones, increased TCR diversity, MHC-II upregulation in PBMCs) are attributable to the CCL21-DC vaccine. However, in a single-arm, small-cohort trial with a highly heterogeneous population and no control arm, it is very difficult to separate vaccine effects from the behavior of indolent tumors or from pembrolizumab alone. The authors should explicitly acknowledge this limitation and be more cautious in assigning causality. In particular, the observation that SD patients often had more favorable baseline features suggests that intrinsic biology, rather than the vaccine, may largely drive outcomes.

We agree that there are major limitations due to sample size and lack of comparator arm, especially in regard to the clinical implications. We have toned down our conclusions and comments particularly where we correlate findings to clinical outcomes (for example, in Abstract; Discussion, page 13, paragraph 3; and Discussion, page 15, paragraph 3). However, we do believe that the study design of having

sequential biopsies from the same tumor pre- and post-DC injection allows us to make observations (such as increased CD4+ T cell infiltration or TCR diversity) that could reasonably be attributable to the vaccine, especially since the vast majority of patients had previously been treated with ICIs.

7. Interpretation of T-cell clonality and dominance of $CD4^+$ naive/memory recruitment: The TCR and scRNA-seq data are a central strength of the manuscript. They show clearly the emergence of novel intratumoral TCR clones, but most do not persist. Only about half of novel clones are detectable in peripheral blood, and clones likely generated de novo within the TME have minimal and transient peripheral representation. Novel clones are predominantly $CD4^+$ naive/memory, enriched for IL7R and ribosomal genes, with limited cytotoxic effector programs and frequent expression of regulatory/activation markers (ICOS, CTLA4). SD patients have a higher baseline burden of pre-existing $GZMB^+$ $CD8^+$ effector clones, whereas the distribution of novel versus pre-existing clones post-treatment does not differ between SD and PD.

Collectively, these findings strongly support the notion that CCL21-DC vaccination could recruit $CD4^+$ naive/memory and regulatory phenotypes to a persistently immunosuppressive TME, with limited induction of durable, tumor-specific $CD8^+$ cytotoxic responses. This aligns with prior literature showing that robust and sustained antitumor immunity generally requires high-avidity, antigen-specific $CD8^+$ T cells.

I would encourage the authors to more explicitly frame this as a key mechanistic conclusion and to discuss potential next steps (e.g., strategies to bias recruitment toward $CD8^+$ cytotoxic clones, modify the cytokine payload, or target myeloid suppressive pathways).

Thank you for this feedback, and we overall agree with framing the data as a story of limited clinical efficacy due to immunosuppressive mechanisms. We have included thoughts on potential next steps in regard to modifying the DC vaccine or additional combination treatment approaches (Discussion, page 16, paragraph 1).

8. Myeloid compartment and lack of microenvironmental reprogramming: The identification of $SPP1^+$ and $IL1B^+$ macrophages expressing MARCO and TREM2, and the dominance of SPP1-mediated macrophage–T-cell signaling, is compelling and consistent with recent literature on immunosuppressive myeloid niches. This pattern offers a plausible explanation for the limited durability of the vaccine-induced T-cell activation. However, the authors stop short of proposing how their therapy could be rationally combined with myeloid-targeting agents or altered to reprogram this macrophage compartment. Adding a more explicit discussion of potential combinatorial strategies (e.g., MARCO/TREM2 blockade, SPP1 axis targeting) would increase the translational value of the work.

Thank you for this suggestion, we have added more concrete statements regarding the potential role of myeloid therapies in combination with a DC vaccine and immune checkpoint inhibition (Discussion, page 16, paragraph 1).

9. Heterogeneity of the patient population and molecular characterization: The cohort includes EGFR-mutated and ALK-positive tumors and presumably other molecular subsets, but molecular profiles are not clearly presented in the main Table of baseline characteristics. I recommend the authors add more granular molecular data (e.g., EGFR, ALK, KRAS, including mutations in genes that usually preclude immune response as STK11, KEAP1, etc.) and discuss whether any of these features are associated with the limited clinical benefit observed. The case with 12 months of SD should be described in more detail (tumor genotype, prior treatments, tumor burden), as it is the single most clinically relevant outcome.

We have collated all the available molecular profiles for these patients and provided it as a separate oncoplot (Supplementary Fig. 2G and Results, page 6, paragraph 2). Of interest, there was stark division between SD and PD patients in regard to KRAS and TP53 mutation status. This could reflect underlying biology, though patients with KRAS mutations had better outcomes even with aggressive co-mutations such as STK11 and KEAP1. In addition, we have provided a more thorough case report of patients of interest to allow readers to evaluate the possibility of a therapy-induced immune response (Results, page 5, paragraphs 3-4).

Minor comments

1. Ethnicity/race variable spelling:
In Table 1, the category “None-Hispanic of Latino” is incorrect. This should be corrected to standard terminology such as “Non-Hispanic or Latino” or “Not Hispanic or Latino”.

Thank you for catching this error; this has been corrected.

2. MTD wording:
The text currently states that “19 patients received at least 2 injections at the maximum tolerated dose (MTD)” although the MTD was not reached. This should be changed to “highest planned dose” or “selected dose for expansion” to avoid confusion.

We have changed references to MTD to maximum administered dose (MAD) which is the term used in our protocol.

3. Accrual period:
The accrual/reference period appears to extend from July 2019 to January 2024 for only 23 patients. While this may be realistic given the complexity of intratumoral vaccination, it would be helpful if the authors comment on the reasons for the relatively slow accrual.

As the reviewer suggests, this is in part due to logistical considerations as well as availability of numerous competing trials at an academic center. We also ran into challenges early on logistically related to the COVID pandemic. However, we were consistently able to complete steps such as DC manufacturing and delivery in a reasonable turnaround time without major issues, which we believe supports the feasibility of this approach. We have commented on these challenges in the Discussion, page 13, paragraph 1.

4. Supplementary AEs table:
It would be helpful if the supplementary AEs table included all-grade AEs in more detail, including attribution (DC vs pembrolizumab vs DC vaccine vs injection), given the novelty of the intratumoral procedure.

We have clarified that the flank pain treatment AE was attributed to the injection procedure (Results, page 5, paragraph 1 and Supplementary Table 1). The remaining treatment AEs were attributed to immunotherapy, though it is not clear clinically to what extent the DC component may have potentiated irAEs associated with pembrolizumab.

Additional comments:

The study is of great interest despite the scarcity of clinical responses. There is a multitude of factors that could impair the benefit of dendritic vaccination in the current study. It could be of interest if the authors could, or have already, determined signatures for predicting the response to dendritic cell vaccines and immunotherapy. Any determination of the tumor microenvironment—whether a hot, T-cell–inflamed environment with high PD-L1 or a cold, T-cell–excluded tumor microenvironment—would be of benefit.

We have provided additional detail including NGS and PD-L1 data by patient and associated response category (Supplementary Fig. 2G and Results, page 6, paragraph 2). There was no clear association with PD-L1 status though there was enrichment of KRAS mutations in the SD group. We have clarified some of the resistance mechanisms we have identified in our analyses (Discussion, page 15, paragraph 1 and paragraph 3), though ultimately we were unable to elucidate a single signature that would predict clinical outcomes.

STK11 and KEAP1 co-mutations negate the effect of immunotherapy. Although the number of patients is small, the NGS information is of interest.

We have provided additional information on the mutation profiles of these patients as derived from NGS testing (Supplementary Fig. 2G), including co-mutations such as STK11 and KEAP1.

Among the markers examined, bear in mind the expression of DNASE1L3 in dendritic cells,

which promotes CD8+ T-cell function(see Chen et al., DNASE1L3-expressing dendritic cells promote CD8+ T cell function and anti-PD-(L)1 therapy efficacy by degrading neutrophil extracellular traps, Cancer Cell, 2025).

Thank you for this suggestion. We did observe DNASE1L3 expression concentrated within the cDC1 cluster, but unfortunately due to the very low numbers of DCs captured by our scRNA-seq data (n=902 across all patients), we are unable to correlate their phenotypes to our clinical or other immune findings. We have however included evaluation of these markers as a potential future direction and cited this and other relevant articles (Discussion, page 15, end of paragraph 3).

Ectonucleotide pyrophosphatase/phosphodiesterase 1 (ENPP1), a hydrolase of extracellular cGAMP, causes immune suppression and can jeopardize the immunotherapeutic approach. Immunotherapy resistance often occurs through overexpression of COL3A1 and COL6A1. M2 polarization, driven by disturbances in cGAS-STING signaling or alterations in dsRNA resolution, often driven by elevation of protein arginine methyltransferase 1 (PRMT1), are some of the markers that should be taken into account.

We have explored various immune biomarkers as suggested by the reviewer. We specifically applied M1/M2 polarization signatures to our macrophage subsets to show a heavy bias towards M2 phenotypes (Fig. 5C and Results, page 10, paragraph 3). We unfortunately did not capture many stromal cells (n=894 across all patients), which are known to be susceptible during the single cell dissociation process, and thus do not have robust data on stromal markers such as COL3A1 and COL6A1.

I suggest re-addressing the discussion, trimming it a bit, and re-focusing on the limitations you have cited. It is of interest but somewhat reiterative and therefore requires more concretion. Keep the above comments in consideration.

Concluding remarks

In summary, this manuscript presents a sophisticated and carefully executed immunologic study of a clinically negative trial. The major value of the work lies in its multi-omics dissection of the limits of DC-based vaccination in a highly immunotherapy-resistant setting, rather than in demonstrating clinical efficacy. With more cautious interpretation, clearer presentation of the clinical context and limitations, and additional refinement of the analyses and discussion, as suggested above, the manuscript could make a useful contribution to the design of next-generation, microenvironment-aware DC vaccine strategies.

Thank you for the very comprehensive feedback. We have made edits throughout the manuscript such that the clinical message is not overstated, and we focus on therapeutic limitations identified in our analysis that could be useful for future iterations of DC vaccines.

Reviewer #3 (Remarks to the Author):

In this manuscript, Oh and colleagues report results from a phase I trial combining intratumoral injection of a CCL21-expressing dendritic cell (CCL21-DC) vaccine with pembrolizumab in patients with advanced NSCLC. Although the combination therapy was well tolerated, there were no objective responses observed. The best response was stable disease, which was observed in 36.8% of patients. Analyses of longitudinal biopsies revealed a therapy-associated increase in tumor infiltration by CD4+ T cells and novel T cell clones. Disease stability was associated with increased T cell receptor (TCR) diversity and greater expansion of novel clones following therapy. However, many novel clones failed to persist over time, while immunosuppressive myeloid signaling signatures were identified across time points. The authors concluded that "CCL21-DC vaccination combined with pembrolizumab can promote antitumor immune responses", but efficacy may be constrained due to limited tumor specificity of the T cell response and an inability to fully reprogram the tumor microenvironment.

I commend the authors on a well-designed phase I trial evaluating a CCL21-DC vaccine combined with PD-1 ICB (pembrolizumab) in advanced, pretreated NSCLC patients. I also commend them on overcoming the logistical hurdles facing obtaining longitudinal pre- and on-therapy tissue samples for their correlative studies. The authors report (1) increased CD4 infiltration and (2) increased intratumoral TCR diversity in patients with stable disease. Unfortunately, there was minimal clinical efficacy and no real objective responses.

I have a few comments:

1- The authors report greater expansion of novel clones after therapy. However the majority of expanded clones after treatment were $CD4^+$ clones ($CCR7^+$ naïve/memory), and there is no phenotypic or functional validation. The data seem consistent with bystander recruitment with the vaccine, rather than tumor-specific priming, and although this is acknowledged in the discussion, the framing in the abstract and results still implies antitumor relevance. For example, "CCL21-DC vaccination with pembrolizumab can promote antitumor immune responses" is stated in the Abstract, although there is no objective response, limited CD8 engagement, and no validated tumor specificity. What appears to be observed is that the combination therapy induces transient clonal perturbations in the TME, although evidence for antitumor immunity remains limited.

Thank you for these comments. We agree with this feedback and have edited the abstract and other sections to better reflect this sentiment. We believe that our studies support possible immune effects of the DC vaccine via CCL21-mediated recruitment, for example, but agree that references to "antitumor" specificity in particular are imprecise. These have been removed from the Abstract and elsewhere. We more explicitly state that the immune response appears to be largely non-specific (Discussion, page 15, paragraph 1) and comment on potential future directions that would be required to validate tumor specificity (Discussion, page 17, paragraph 1).

2- The TCR diversity claim need to carefully consider bystander effects, as the authors repeatedly state that increased TCR diversity is favorable because it is mostly found in patients with stable disease. However, as noted above, the recruitment is largely of naïve-like CD4 cells that do not have demonstrated antitumor function(or at least are not shown to have antitumor function by the authors). These data may also be confounded by repeated spatial sampling. The authors use a 0.2% clonal frequency cutoff to show clonal expansion, but this threshold seems arbitrary and requires further explanation. Although several groups using TCR/scRNA sequencing to identify tumor-relevant clones provide strict thresholds (e.g., >5 or >10 cells per clone to define expansion), remove bystanders using published viral-associated TCR databases (e.g., VDJdb), and validate findings with published transcriptional signatures of tumor reactivity (Caushi et al., Lowery et al. Nature 596.7870 (2021): 126-132), such approaches were not applied here. Further computational analysis of this type would greatly strengthen the validity of the TCR diversity and cell-state results.

We agree that a direct mechanistic connection between TCR diversity and clinical benefit is not demonstrated by our data. We have included thoughts in the Discussion and have tempered statements regarding the importance of TCR diversity (page 13, paragraph 3 and page 15, paragraph 1). We instead propose that the TCR diversity may reflect initial immune infiltration achieved by the therapy without leading to robust CD8+ responses that would be expected had any patients experienced an objective response. We still find it intriguing that the greatest increases in TCR diversity were observed in patients with the greatest clinical benefit, but have tried to more clearly state that this does not represent mechanistic evidence for antitumor immunity (Discussion, page 14, paragraph 1 and page 15, paragraph 1).

For clonal expansion, we have clarified that we have chosen the threshold due to it representing the 95th percentile in clonal proportion (Results, page 9, paragraph 2). Due to having tumor samples from multiple patients with varying sampling characteristics, we felt that having a percentage would be more applicable across the dataset, rather than absolute numbers. The literature does report several other thresholds to define expanded clones, but we feel these are also somewhat arbitrary in nature as there has been no standard established for this purpose.

We agree that cross referencing viral TCR databases can be helpful for identifying bystanders. However, the primary limitation with this method is that it is not exhaustive – in our dataset, only 4.3% of clones were identified as viral through VDJdb. However, this analysis did provide some supporting insights into T cell phenotypes (Results, page 10, paragraph 2 and Supplementary Fig. 6D).

In regard to tumor reactive signatures, we had previously applied these to characterize the T cell scRNA clusters (Supplementary Fig. 3C). This analysis included signatures from the Lowery et al paper as well as an updated scoring method from Dr. Kellie Smith's group who had published Caushi et al. We have now further applied these

signatures to help support our assertion that "novel persistent" clones are more likely to represent tumor-relevant T cells (Supplementary Fig. 6E). We however acknowledge that these cells are few in number and likely insufficient to mount a truly effective immune response (Discussion, page 15, paragraph 1).

3- Reprogramming of the suppressive TME is also explored by the authors, showing enrichment of suppressive macrophages with MARCO and TREM2 expression, which is important given preclinical evidence of CCL21-DC–mediated reprogramming that was not observed in this trial. CXCL9/10 signaling was also observed in only three patients with stable disease. In the discussion, this finding may be overstated as promoting an inflammatory state in stable disease patients, given the limited number of cases.

We have restated our interpretation of this section to more simply provide the data and minimize the clinical association given the small numbers of samples (Results, page 10, paragraph 3 and Discussion, page 15, paragraph 3).

4- MIF analysis showed increased CD4 T-cell infiltration. A suggestion would be to assess spatial features within the MIF data, if possible, including proximity to tumor cells, immune exclusion, and relationships with macrophages, given the importance of immunosuppressive macrophages highlighted by the authors.

We have performed a spatial neighborhood analysis using tumor centroids, and we show that the T cell infiltration seen broadly in the tumor leads to greater CD4+ T cell proximity to tumor cells (Supplementary Fig. 5A-D). Due to a 7-color limit to our MIF, we have thus far been able to only apply a T cell-specific panel. We agree that a myeloid panel would be of particular interest, but unfortunately we now have limited residual tissue to perform further imaging analyses.

In conclusion, this study provides value in characterizing the immunologic consequences of intratumoral CCL21-DC vaccination in combination with pembrolizumab in advanced-stage NSCLC. The data demonstrate immune cell recruitment and transient clonal dynamics but fails to show tumor-specific immunity or meaningful reprogramming of the TME. Several conclusions should be restated to reflect these findings more accurately.

Thank you for your thorough feedback and suggestions. In addition to the specific changes above, we have reframed our conclusions to more concretely report the limited clinical efficacy seen in this trial, as well as to highlight the immunosuppressive mechanisms revealed in the correlative studies.

Reviewer #4 (Remarks to the Author):

This is a phase I trial evaluating a vaccine composed of autologous dendritic cells (DCs) engineered to overexpress the chemokine CCL-21 (via an adenoviral vector), administered in combination with the anti-PD-1 antibody pembrolizumab. CCL-21 promotes the

colocalization of T cells and DCs, while pembrolizumab is an immune checkpoint inhibitor; together, these agents may enhance antitumor immunity within the tumor microenvironment. The rationale for combining these therapies also stems from a prior phase I trial of the CCL-21 DC vaccine, in which PD-L1 (the ligand for PD-1) overexpression was observed.

The clinical trial uses a two-step design with a modified dose escalation (applied only to the CCL-21 DC vaccine; pembrolizumab was administered at a fixed dose) following a 3+3 scheme, followed by a dose-expansion cohort. In the dose-escalation phase, the primary endpoint is the Maximum Tolerated Dose (MTD), defined as the dose with unacceptable toxicity in strictly fewer than 33% of treated patients. In the dose-expansion phase, the primary endpoint is the overall response rate according to RECIST v1.1. Several translational analyses were performed in parallel using tumor biopsies and blood samples, and these constitute the majority of the results presented in the article.

No dose-limiting toxicities were observed, and therefore the MTD was not reached. In terms of efficacy, no patient experienced an objective response to treatment.

As a statistician, I focus my comments on the clinical trial design and general considerations related to the statistical analyses in this study.

Transparency: The protocol and clinical trial registration number are provided, and there were no deviations from the submitted protocol. I have no concerns on this point.

Design: This is a standard statistical design for a phase I study, following a 3+3 approach. However, the cohort size at each dose level was not consistently three patients as usual; in some cases it was six, while still adhering to the targeted 33% unacceptable toxicity rate. The sample size justification for the dose-expansion cohort, based on a two-sided binomial proportion test, is reproducible.

Comments:
- Could you provide the median follow-up time for patients in the study? Some censoring appears to have occurred early.

The median follow-up time by reverse Kaplan-Meier was 32.3 months (Results, page 5, paragraph 2) . We apologize for the oversight, but on review one of the early censoring events was labeled in error and the KM plots have been corrected (Supplementary Fig. 2D-E). There were 2 patients censored relatively early (1 in SD group and 1 in PD group) who were lost to follow-up shortly after coming off trial. We have added these details to the Supplementary Fig. 2 legend.

- Could you also specify the number of patients enrolled at each dose-escalation stage in the flow diagram and give the reasons for exclusion (+replacement if applicable)? According to the protocol, six patients should have been treated at the maximum dose level, whereas

the text refers to eight patients. It is also unclear how 19 patients were included in the efficacy set.

We have amended the flow diagram (now shown separately in Supplementary Fig. 1) to better reflect the patient enrollment and treatment flow. Specifically, we have made note of 2 patients who did not complete the 3rd DC vaccine injection in the dose escalation cohort. In addition, we have clarified that 4 among the 23 treated patients were not efficacy evaluable due not meeting the definition of "2 injections at the maximum dose level." We hope this provides greater clarity regarding the exclusions.

General considerations: The sample size in this study is very small (as the authors acknowledge in the discussion). Given this, it is unlikely that assumptions for parametric tests such as Student's t-tests (Figures 4 and 6) are satisfied such as normality. I recommend using non-parametric alternatives such as the Wilcoxon rank-sum test. In addition, with respect to the translational analyses, the cohort size is so limited that it is highly unlikely that robust or reliable inferences can be drawn from these data.

We have changed statistical analyses to non-parametric tests where applicable (including Figs. 4 and 6). We also have tempered our language regarding the strength of statistical conclusions based on the small sample size.

Despite these limitations, I found it valuable that the authors explored mechanistic explanations for why the combination of the DC vaccine with pembrolizumab failed to elicit a clinical response. Such investigations might help identify ineffective candidates early and prevent unnecessary research burden.

Thank you for your thoughtful feedback for this manuscript.

<u>Reviewer #5 (Remarks to the Author):</u>

I co-reviewed this manuscript with one of the reviewers who provided the listed reports. This is part of the Nature Communications initiative to facilitate training in peer review and to provide appropriate recognition for Early Career Researchers who co-review manuscripts.

Thank you for your contributions to the review of our manuscript.
